# Navigating the complexities of digital health technology implementation: a scoping review of barriers and facilitators

**DOI:** 10.1186/s43058-026-00892-4

**Published:** 2026-03-04

**Authors:** Laura Wittich, Hendrikje Rödiger, Tanja Rombey, Anna-Lena Brecher, Lena Kraft, Sophia Sgraja, Viktoria Stein, Cornelia Henschke

**Affiliations:** 1 Department of Health Care Management, Faculty of Economics and Management, Berlin University of Technology, Straße Des 17. Juni 135, Berlin, BE 10623 Germany; 2 Hannover Medical School, Institute of Epidemiology, Social Medicine and Health Systems Research, Carl-Neuberg-Str. 1, Hannover, 30625 Germany; 3 Department of Public Health and Primary Care, Leiden University Medical Centre, Health Campus The Hague, Wijnhaven Building, Turfmarkt 99, The Hague, DP 2511 The Netherlands; 4 Institute of General Practice and Interprofessional Care, University Hospital Tübingen, Tübingen, Germany

**Keywords:** Digital Health Technologies, Implementation Barriers, Implementation Facilitators, Human and Social Dynamics, Organisational Readiness, Infrastructure and Data Security, Adoption Strategies, Digital Literacy

## Abstract

**Background:**

The implementation of digital health technologies (DHTs) is a strategic priority for many health systems, yet integrating them into routine clinical use remains challenging. While numerous studies explore DHT adoption, few provide a comprehensive perspective across technologies and stakeholder groups. This review synthesises the most prevalent barriers and facilitators to DHT implementation in high-income healthcare settings.

**Methods:**

A scoping review was conducted following Joanna Briggs Institute and PRISMA-ScR guidelines. Publications from 2019 to 2024 reporting barriers or facilitators to DHT implementation in upper-middle and high-income countries were identified through systematic searches in PubMed and Scopus. An inductive approach guided iterative coding and thematic categorisation. Findings were synthesised based on frequency, overlap, and variation across technologies and stakeholder groups.

**Results:**

From 15,327 unique records screened, 238 publications were included. In total, 2538 barriers and 1433 facilitators were identified, grouped into three overarching dimensions: human and social dynamics, organisational structure and management, and infrastructure and data security. Human and social factors such as resistance to change, scepticism, and limited digital literacy were the most frequently reported across the majority of technologies and stakeholder groups. Organisational barriers, including funding constraints, workflow misalignment, and limited leadership engagement, along with infrastructure-related challenges such as poor usability, data privacy concerns, and interoperability issues, were also substantial but were comparatively less frequent. Patterns varied by technology type (e.g., telehealth, mobile health apps, AI tools) and stakeholder group (e.g., healthcare professionals, health system managers, users of health services), highlighting the complex, context-dependent nature of DHT implementation.

**Conclusions:**

Successful DHT implementation demands more than technical readiness. It requires organisational leadership, robust infrastructure, and system-wide alignment. While human and social dynamics remain central, leadership, resource allocation, and robust infrastructures are equally critical. Current evidence often underemphasises structural barriers such as governance gaps, misaligned incentives, and technical limitations. Sustainable digital transformation requires a balanced approach combining top-down strategic guidance for regulatory clarity with bottom-up engagement to foster cultural change. Future research should operationalise governance strategies, leadership practices, and monitoring indicators that support long-term digital health integration.

**Registration:**

A prospective protocol was uploaded to the Open Science Framework: https://osf.io/vr7d9/ (10.17605/OSF.IO/VR7D9).

**Supplementary Information:**

The online version contains supplementary material available at 10.1186/s43058-026-00892-4.

Contributions to the literature

- This review shows that the successful implementation of digital health technologies depends on the dynamic interplay of human, organisational, and infrastructural conditions.
- Based on a comprehensive, inductively derived categorisation, we present key implementation factors across human, organisational, and infrastructural domains.
- The paper distinguishes between adoption and implementation, showing that each stage demands tailored strategies across user, organisational, and system levels.
- It highlights underexplored barriers at the organisational and system level, such as misaligned incentives, limited leadership capacity, and governance gaps.
- By synthesising findings across technologies and stakeholder groups, the review identifies cross-cutting factors that support sustainable, system-wide digital transformation

## Background

The digitalisation of healthcare is advancing globally, albeit at varying speeds across the European Union and OECD countries [1]. A central element of this transformation is the adoption of digital health technologies (DHTs), which encompass a wide range of innovations, including electronic health records, telehealth, mHealth (mobile health) applications, and artificial intelligence [2]. These technologies have the potential to enhance healthcare quality, improve patient safety, and reduce costs [3].

The digitalisation of healthcare is advancing globally, albeit at varying speeds across the European Union and OECD countries.1 A central element of this transformation is the adoption of digital health technologies (DHTs), which encompass a wide range of innovations, including electronic health records, telehealth, mHealth (mobile health) applications, and artificial intelligence.2 These technologies have the potential to enhance healthcare quality, improve patient safety, and reduce costs [3].

Recognising the importance of digital transformation, various global initiatives have been established to support the digitalisation of healthcare. For instance, the Global Digital Health Partnership and the World Health Organization Digital Health Strategy highlight the need for strong leadership, sustainable funding, regulatory alignment, and robust governance frameworks to facilitate the implementation of digital health technologies [3, 4]. Additionally, key enablers such as a digitally skilled workforce, interoperable systems, and secure infrastructure have been identified as essential to advancing digital healthcare [5].

Implementation can be pursued through different strategies. A top-down approach relies on strong governance, with policymakers and institutions driving digital transformation through regulation, financial incentives, and mandates [3]. In contrast, bottom-up initiatives, shaped by private-sector companies, healthcare providers, and patients [6], often lead to higher user engagement and acceptance [7]. While top-down policies provide regulatory alignment and funding, bottom-up approaches enable innovation and adaptability to local needs. In practice, a balanced strategy that integrates elements of both is often key to the successful adoption of digital innovations in healthcare [8].

Despite these efforts, healthcare systems continue to face major challenges in implementing DHTs [9]. The transition from technology development to full integration into routine care is a complex process. It requires regulatory approval or certification in accordance with applicable regulations, reimbursement decisions from public health insurers, and broad adoption by both healthcare professionals and patients [10]. Governance and legal frameworks must keep pace in alignment with the lifecycle of technologies [11] while maintaining patient safety, data privacy, and security [12]. Nevertheless, implementation efforts often face resistance, technical and organisational constraints, or insufficient support structures [13].

While extensive research has explored DHT implementation, many existing studies focus on specific stakeholder groups (e.g., healthcare professionals [14]), medical conditions (e.g., diseases like chronic obstructive pulmonary disease [15]), or technologies (e.g., mHealth [16]), thereby limiting their overall scope.

To address this gap, this review provides a comprehensive synthesis of barriers and facilitators shaping the implementation of DHTs across different stakeholder groups and technologies. By systematically identifying and categorising the most prevalent factors, this work aims to inform strategies that accelerate DHT adoption in high-income healthcare systems. Examining diverse implementation experiences across contexts will provide insights for developing more effective, scalable, and sustainable digital transformation strategies.

## Methods

This scoping review followed the recommendations of the Joanna Briggs Institute Manual for Evidence Synthesis for conducting scoping reviews [17] and adhered to the *PRISMA-ScR* (Preferred Reporting Items for Systematic Reviews and Meta-Analyses extension for Scoping Reviews) *Checklist* [18] (see *Additional file 1*). A prospective protocol was uploaded to the Open Science Framework and can be accessed at https://osf.io/vr7d9/.

### Searches

A comprehensive search strategy was developed in collaboration with experienced librarians and applied to the electronic databases PubMed (including MEDLINE and PubMed Central) and Scopus. The search was supplemented by citation tracking, in which the reference lists of included systematic reviews were screened using the Citationchaser web application to capture additional relevant publications [19].

The search combined MeSH terms, free-text keywords, and synonyms related to digital health technologies, barriers and facilitators, and implementation. The whole search strategy, including the search strings, is detailed in *Additional file 2*.

All identified references were imported into Covidence software [20] for deduplication and screening. A 20% sample of records was independently screened by two reviewers (LW, HR) to assess interrater agreement. If agreement exceeded 80%, the remaining records were screened by a single reviewer (LW); otherwise, an additional 20% sample was reassessed. Any discrepancies were resolved by consensus. After title and abstract screening, full-text review was conducted by one reviewer (LW), with a second reviewer (HR) cross-checking excluded publications to ensure consistency. Reference lists of included systematic reviews were manually reviewed to identify additional publications.

### Study inclusion and exclusion criteria

Publications were included if they were published in English or German between January 2019 and March 2024 and conducted in upper-middle-income and high-income countries (HMICs), as classified by the World Bank Country and Lending Groups, [20]*.* Studies from low- and middle-income countries (LMICs) were excluded to ensure comparability, given that their infrastructural and systemic conditions differ markedly. While LMICs may rely on digital tools to address gaps in physical infrastructure and workforce shortages, HMICs face challenges related to system complexity, interoperability, and regulation.

Eligible publications examined barriers and facilitators to DHT implementation. Barriers were defined as factors, conditions, or characteristics reported to impede, delay, or adversely affect DHT implementation in routine practice; conversely, facilitators were defined as factors that enable, accelerate, or positively influence implementation. All empirical study designs were included, such as qualitative, quantitative, and mixed-methods approaches. Non-empirical publications (e.g., editorials, protocols) and condition-specific studies (e.g., a blood sugar monitoring app) were excluded, as they provide limited insight into broader system-level issues.

Existing DHT classifications, such as those developed by the World Health Organization (WHO) [20], were deemed insufficiently granular and precise for the scope of this review. Therefore, a refined six-group classification was developed (detailed in *Additional file Table A1*):

- Health Data Infrastructure – Systems for managing, storing, and integrating electronic health data, with a focus on accessibility, interoperability, and security (e.g., electronic health records, large-scale data platforms).
- Telehealth – Remote healthcare delivery, including services such as video consultations, telemonitoring, and telerehabilitation.
- Digital Health Applications (DiHA) – Patient-centred tools such as mobile apps for disease management, self-monitoring, and medication adherence, sometimes incorporating provider-facing features.
- Artificial Intelligence (AI) – AI-enabled solutions in healthcare, including predictive analytics, clinical decision support, and process automation based on machine learning and Internet of Things technologies.
- General Digital Health Technologies – Broad or cross-cutting digital innovations not classified elsewhere, such as digital health policy platforms or digital inclusion initiatives.
- Conceptual or Niche DHTs – Emerging or specialised technologies, including Health 4.0 frameworks, augmented reality applications, and novel wearable devices.

The stakeholder populations were classified according to the WHO’s digital health taxonomy [20], with the addition of Health Data Service Providers to capture the perspectives of technical industries involved in digital health innovation. The final classification included four stakeholder groups (detailed in *Additional file Table 2*):

- Users of Health Services – Individuals who are potential or current users of health services, including patients, caregivers, and general citizens.
- Healthcare Professionals – Members of the health workforce delivering health interventions, such as hospitals, rehabilitation centres, and clinicians.
- Health System Managers – Stakeholders involved in the administration and oversight of health systems, including governance bodies, insurers, and research institutions.
- Health Data Service Providers – Technical entities involved in digital health innovation, such as software developers, start-ups, and medical device manufacturers.

### Data extraction strategy

Data extraction was carried out in Covidence software, beginning with a pilot of 10% of included publications independently reviewed by two researchers (LW, HR) to refine the approach. This pilot calibrated operational definitions of “barriers” and “facilitators”, refined procedures for extracting verbatim text segments (i.e., only passages directly reporting on implementation factors), and generated over 40 preliminary codes through open coding. These codes were iteratively refined and grouped into subcategories and, ultimately, into the three overarching dimensions (Human & Social Dynamics, Organisational Structure & Management, Infrastructure & Data Security). Complex cases were discussed within the core review team (LW, HR, AB, LK, SS) to ensure shared understanding. After resolving discrepancies, the remaining publications were divided among the reviewers (LW: 150 publications; HR: 64 publications). Extracted variables included study characteristics (authors, year, design, country), stakeholder group, healthcare setting, DHT type, and key findings on implementation barriers and facilitators.

### Data synthesis and presentation

Thematic analysis was conducted using ATLAS.ti Windows version 25.0.1.32924 [21]. An inductive approach guided the development of categories and subcategories for barriers and facilitators. Using a data-driven, iterative coding process, factors were identified without imposing predefined theoretical constructs. A coding guideline was developed to ensure consistency across publications. Each code contained a definition and illustrative examples, clarified distinctions between reportable implementation factors and contextual information, and included decision rules for complex cases, such as multi-causal resistance to change, spanning human, organisational, and infrastructural levels. Established frameworks, such as the Consolidated Framework for Implementation Research (CFIR), were not applied because their domains may overlook emerging or technology-specific factors.

A narrative synthesis was conducted in line with best practices for scoping reviews [17]. Publication characteristics and key themes were analysed based on frequency and thematic overlap to identify commonalities, divergences, and overarching categories. Internal validity was supported through dual screening, iterative coding, and team consensus. Data-driven categories were grouped into overarching dimensions and ranked by frequency, with percentages indicating their relative prominence.

## Results

### Review statistics

A total of 19,760 publications were identified through systematic searches in PubMed and Scopus. An additional 15 publications were retrieved via reference screening of included publications. Following deduplication, 15,327 unique records remained. After title and abstract screening, 463 publications were selected for full-text review, of which 238 met the inclusion criteria. *Additional file Fig. 1* illustrates the selection process in a PRISMA Flow-Chart, and *Additional file Table 3* provides a list of excluded publications along with the reasons for exclusion.

### Characteristics of publications

In terms of methodological design, 98 publications were reviews, 86 qualitative publications, 32 quantitative publications, and 22 employing mixed methods. The majority of publications were released in 2023. The publication period ranged from 2019 to 2024, with the annual number of publications tripling from 2019 (n = 24) to 2023 (n = 80). Regional analysis revealed that most publications focused on Europe and Central Asia (n = 58), followed country-wise by the United States (n = 31), Germany (n = 14), and Saudi Arabia (n = 11). Overall, 30 publications were non-region-specific, while 38 studies covered multiple countries or regions. The characteristics of the included studies are presented in *Additional file Table 4*.

### Barriers and facilitators

A total of 2538 barriers and 1433 facilitators were identified, grouped into three dimensions: *Human & Social Dynamics*, which included social, psychological, and cultural factors; *Organisational Structure & Management,* encompassing structural, regulatory, and leadership issues; and *Infrastructure & Data Security,* covering technical, security, and interoperability needs. Detailed results for all included publications are provided in *Additional file Table 5*.

There were 1160 barriers and 638 facilitators relating to the dimension *Human & Social Dynamics* (accounting for approximately 45.7% of all identified barriers and 44.5% of all facilitators. The remaining proportions were divided between *Organisational Structure & Management* (24.8% barriers, 26.3% facilitators) and *Infrastructure & Data Security* (29.5% barriers, 29.2% facilitators). Some categories appeared in both the barrier and facilitator columns because inductive coding indicated that similar underlying factors were reported to hinder or enable implementation, depending on the specific context described in the included publications.

A total of five categories were assigned to the *Human and Social Dynamics* dimension (see Table 1).

**Table 1:** Categories and frequency of barriers and facilitators in human and social dynamics

HUMAN & SOCIAL DYNAMICS					
**Frequency of times coded**		**in %**	**Frequency of times coded**		**in %**
**Barriers (sum)**	**1160**	**100%**	**Facilitators (sum)**	**636**	**100%**
**Acceptance & Engagement**	**495**	**42.7%**	**Perceived Effectiveness & Value**	**242**	**37.9%**
Resistance to Change	155	13.4%			
Lack of Interest & Motivation	127	10.9%			
Fear & Scepticism	97	8.4%	**Acceptance & Engagement**	**159**	**24.9%**
*Lack of Trust and Transparency*	*47*	*4.1%*	Readiness to Embrace Technology	101	15.8%
*Negative Impact on Provider-Patient Relationship*	*29*	*2.5%*	Strengthened Patient-Provider Relationship	38	6.0%
*Concerns about Autonomy & Increased Responsibilities*	*29*	*2.5%*	*Promoting Trust & Transparency*	*20*	*3.1%*
*Preference for Traditional Care & Established Social Norms*	*11*	*0.9%*			
**Literacy & Skills**	**303**	**26.1%**	**Literacy & Skills**	**149**	**23.4%**
Low Digital Literacy & Skills Deficits	167	14.4%	Strong Social Network & System Support	83	13.0%
Insufficient Training & Technical Support	108	9.3%	Robust Skills & Capacity Building	44	6.9%
*Inadequate Information & Lack of Awareness*	*28*	*4.4%*	*Education & Awareness*	*22*	*3.4%*
**Sociocultural and Economic Aspects**	**193**	**16.6%**	**Sociocultural and Economic Aspects**	**83**	**12.9%**
Cultural Differences & Language Barriers	152	13.1%	Improved Access to Care	69	10.8%
*Limited Affordability & Financial Constraints*	*41*	*3.5%*	*Financial Support*	*14*	*2.2%*
**Scepticism about Effectiveness and Value**	**115**	**9.9%**			
***Ethics***	***54***	***4.7%***	***Ethics & Transparency***	***5***	***0.8%***
*Ethical Concerns*	*26*	*2.2%*			
*Bias and Discrimination*	*23*	*2.0%*			

Legend Table 1: Categories accounting for less than 5% within the respective dimension are presented in italics to highlight their potentially lower importance

The largest barrier category was *Acceptance & Engagement* (42.7%), driven by resistance to change, lack of interest and motivation, fear, and scepticism. Barriers under 5% included trust issues, negative provider-patient relationships, autonomy concerns, and adherence to traditional care norms. *Literacy & Skills* (26.1%) encompassed limited digital literacy, insufficient training, and a lack of technical support, ranging from basic device use to unfamiliarity with underlying technologies. Barriers related to information and awareness were less common and indicated difficulties, conveying the benefits and functionalities of digital technologies to target audiences. *Sociocultural and Economic Aspects* (16.6%) reflected cultural differences and language barriers, particularly affecting low-income and less-educated populations, as well as individuals with age-related, cognitive, or physical impairments. *Scepticism About Effectiveness and Value* (9.9%) captured doubts regarding clinical benefit or cost-effectiveness. Finally, *Ethics* (4.7%) addressed concerns about bias, discrimination, and environmental impact.

Facilitators largely reflected the same categories. The most prominent was *Perceived Effectiveness & Value* (37.9%), associated with improved care quality, operational efficiency, and added value through convenience, empowerment, and personalised treatment options. *Acceptance & Engagement* (24.9%) were characterised by openness to technology, supported by positive attitudes, service satisfaction, and both intrinsic and extrinsic motivation to adopt digital technologies. *Literacy & Skills* facilitators (23.4%) included informal support from families, caregivers, and peers, along with targeted education and training for users and professionals. *Sociocultural and Economic Aspects* (13.0%) included improved access, as reflected in reduced travel times and greater specialist availability. Consistent with the barriers, *Ethics & Transparency* (0.8%) were rarely reported as key enablers of implementation.

A total of four categories were assigned to the Organisational Structure & Management dimension (see Table 2).

**Table 2:** Categories and frequency of barriers and facilitators in organisational structure and management

ORGANISATIONAL STRUCTURE & MANAGEMENT					
**Frequency of times coded**		**in %**	**Frequency of times coded**		**in %**
**Barriers (sum)**	**629**	**100%**	**Facilitators (sum)**	**377**	**100%**
**Resource Allocation**	**207**	**32.9%**	**Work Culture**	**159**	**41.8%**
Insufficient Funding	139	22.1%	Active Leadership Engagement	87	22.8%
Shortage of Skilled Workforce	49	7.8%	Interdisciplinary Collaboration among Stakeholders	44	11.8%
*Inadequate Reimbursement Models*	*19*	*3.0%*	Technical & Administrative Support	25	6.7%
			*Defined Roles & Transparent Processes*	*3*	*0.8%*
**Workflow Compatibility**	**160**	**25.4%**	**Resource Allocation**	**76**	**20.4%**
Competing Priorities & Provider Overload	78	12.4%	Sufficient Financial Investment & Funding	42	11.1%
Workflow Integration Challenges	67	10.7%	Project Management & Resource Allocation	21	5.6%
*Lack of Standardised Protocols*	*15*	*2.4%*	*Insurance & Reimbursement Models*	*14*	*3.7%*
**Regulatory Challenges & Policy Gaps**	**132**	**21.0%**	**Workflow Compatibility**	**72**	**19.3%**
			Smooth Workflow Integration	26	6.9%
**Work Culture**	**130**	**20.7%**	Work-Life Balance and Flexibility	25	6.6%
Lack of Strategy, Resistance to Change & Bureaucracy	80	12.6%	Standard-Setting	21	5.6%
Lack of Leadership Support	31	4.9%			
*Structural Resistance to Innovation*	*10*	*1.6%*	**Government Policy & Regulatory Engagement**	**69**	**18.3%**
*Commitment & Role Clarity*	*9*	*1.4%*			

Legend Table 2: Categories accounting for less than 5% within the respective dimension are presented in italics to highlight their potentially lower importance

*Resource Allocation* (32.9%) emerged as the most frequently reported barrier, reflecting funding shortages and a lack of skilled workforce. Inadequate reimbursement models further impeded adoption, as misaligned financial incentives resulted in undercompensation for virtual care. *Workflow Compatibility* (25.4%) encompassed competing clinical priorities, administrative burdens, and documentation demands. Fragmented practices and siloed partnerships contributed to poor workflow integration and operational inefficiencies. *Regulatory Challenges & Policy Gaps* (21.0%) captured legal and governance issues, including non-uniform data exchange rules, ambiguous legal frameworks, and unclear accountability, which hindered compliance efforts. *Work Culture* (20.7%) involved resistance to change, weak digital leadership, risk aversion, and centralised decision-making, all of which limited capacity for innovation. Lack of leadership support further constrained implementation efforts.

Facilitators within *Work Culture* (42.2%) highlighted active leadership, clear strategic direction, and proactive engagement of local champions and ‘super-users’ to drive innovation and support change management. Interdisciplinary collaboration among healthcare professionals, policymakers, and technology developers enhanced coordination through cross-sector partnerships and technical working groups. Structured training, including 'train-the-trainer' models, supported system-level integration. *Resource Allocation* (20.4%) functioned as a facilitator when adequate financial investment, especially in rural areas, was paired with effective project management and targeted resource distribution. Expanded reimbursement frameworks contributed to covering a broader range of digital services. *Workflow Compatibility* (19.1%) facilitators included technologies that aligned well with existing routines, supported gradual introduction in non-critical tasks, and helped relieve provider workload. Standard-setting enabled streamlined data exchange and regulatory clarity. *Government Policy & Regulatory Engagement* (18.3%) promoted broader implementation through supportive legislation, financial incentives, and alignment with national health agendas.

Barriers within the *Infrastructure & Data Security* dimension were evenly distributed across four categories (see Table 3).

**Table 3:** Categories and frequency of barriers and facilitators in infrastructure and data security

INFRASTRUCTURE & DATA SECURITY					
**Frequency of times coded**		**in %**	**Frequency of times coded**		**in %**
**Barriers (sum)**	**749**	**100%**	**Facilitators (sum)**	**416**	**100%**
**Usability**	**191**	**25.5%**	**Usability**	**239**	**57.7%**
Poor Usability & Interface Design	158	21.1%	Ease of Use	166	39.9%
*Therapeutic Functionality Limitations*	*33*	*4.4%*	User Engagement	46	11.1%
			Intuitive Design & Functionality	28	6.7%
**Privacy, Security & Liability Challenges**	**187**	**25.0%**	**Accessiblity & Quality**	**76**	**18.2%**
Privacy & Confidentiality Concerns	160	21.4%	Data Quality & Reliability	36	8.6%
*Legal Liability*	*27*	*3.6%*	Interoperability	27	6.5%
			*Enhanced Data Accessibility*	*13*	*3.1%*
**Hardware & Software Requirements**	**186**	**24.8%**	**Robust Data Security & Privacy Measures**	**57**	**13.6%**
Inadequate Digital Infrastructure & Equipment	90	12.0%	Clear and Supportive Legal Frameworks	1	0.2%
Insufficient Internet Connectivity & Bandwidth	89	11.9%			
*Lack of IT Support & Training*	*7*	*0.9%*	**Hardware & Software Requirements**	**45**	**10.8%**
			Reliable IT Infrastructure & Equipment	29	7.0%
**Accessibility & Quality**	**185**	**24.7%**	*Reliable Network Connectivity*	*9*	*2.2%*
Data Fragmentation, Lack of Interoperability & Standards	103	13.8%	*Comprehensive Training & Support*	*7*	*1.7%*
Poor Data Quality & Accuracy	65	8.7%			
*Accessibility & Technical Challenges*	*17*	*2.3%*			

Legend Table 3: Categories accounting for less than 5% within the respective dimension are presented in italics to highlight their potentially lower importance

*Usability* (25.5%) was the most frequently reported barrier. Contributing factors included non-intuitive layouts, limited adaptability, and poor interface design that failed to meet user needs. Limitations in therapeutic functionality also hindered adoption, particularly when virtual formats restricted diagnostic processes or weakened provider-patient rapport. *Privacy, Security & Liability Challenges* (25.0%) involved concerns about data ownership, confidentiality, cybersecurity threats, and re-identification risks. Legal uncertainty regarding medico-legal accountability added further complexity. *Hardware & Software Requirements* (24.8%) posed major obstacles, especially in settings with inadequate infrastructure, outdated hardware, low processing capacity, or unreliable internet connectivity. These issues were particularly pronounced in underserved areas, where network interruptions disrupted service delivery. *Accessibility & Quality* (24.7%) issues were linked to data fragmentation, interoperability issues, and inconsistent standards, which complicated data exchange between institutions. Poor data quality and system obsolescence also limited reliability and undermined implementation efforts.

Facilitators mirrored these categories. *Usability* (57.4%) was most commonly cited, supported by intuitive design, clear instructions, and engaging interfaces that enhanced user satisfaction and efficiency. Early and sustained user involvement, including patients and professionals, further improved adoption and long-term use. Tailored design features addressed the needs of specific groups, such as older adults or individuals requiring personalised therapy. *Accessibility & Quality* (18.2%) was strengthened by improved data reliability, interpretability, and validation processes. Local pilot testing supported real-world implementation and scalability. Interoperability was enhanced through standardised platforms, robust exchange pathways, and phased integration, thereby reducing fragmentation and aligning systems. *Robust Data Security & Privacy Measures* (13.6%) included strong encryption, secure transmission, and privacy-by-design principles, aligned with standards such as the General Data Protection Regulation (GDPR) and the Health Insurance Portability and Accountability Act (HIPAA). Additional safeguards, including regular audits and privacy-enhancing techniques like anonymisation or federated learning, supported regulatory compliance and stakeholder trust. Finally, *Hardware & Software Requirements* (10.8%) were cited as facilitators when sufficient IT infrastructure—reliable servers, storage, and processing capacity—was in place.

### Barriers and facilitators by technology

**Fig. 1:**
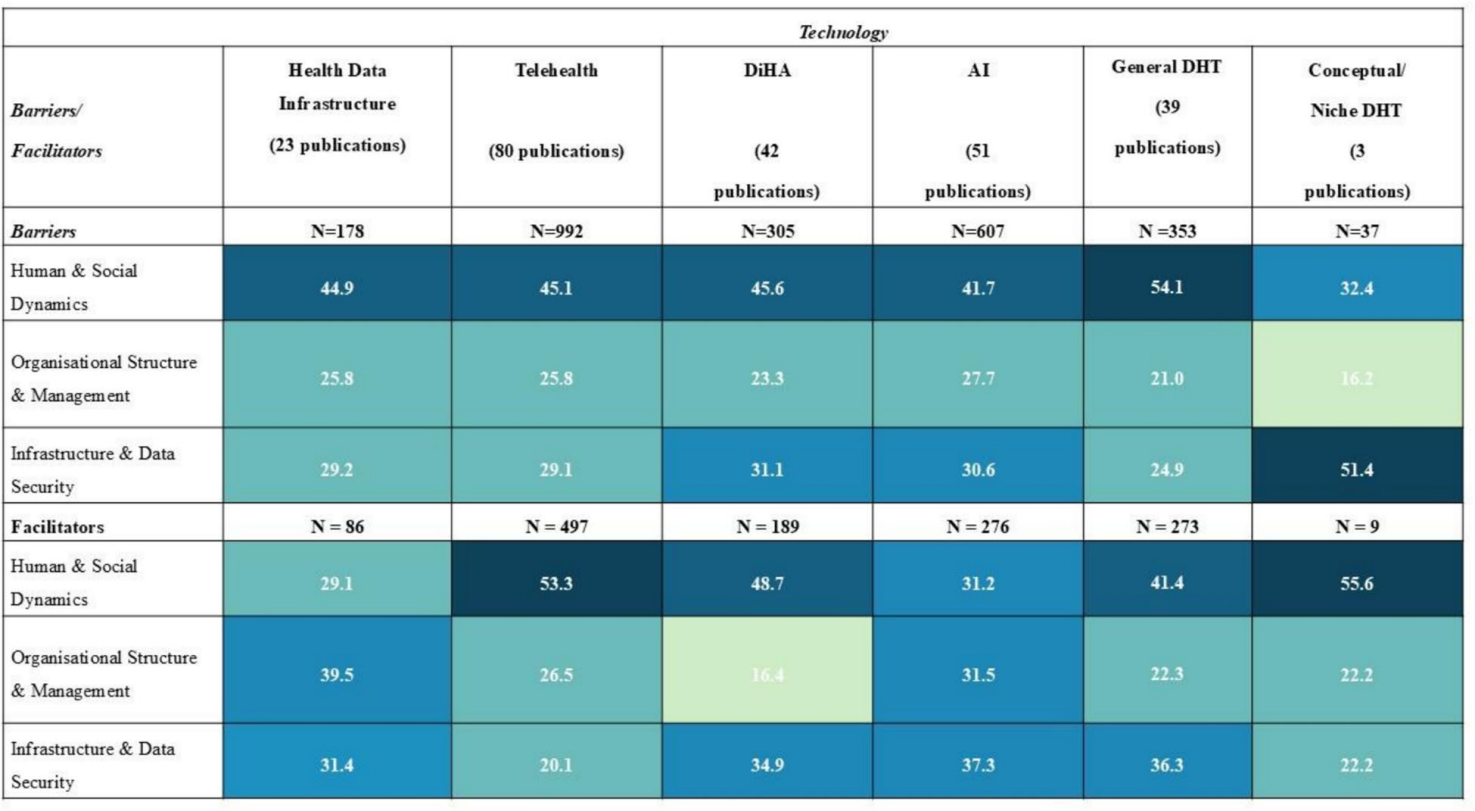
Distribution of barriers and facilitators by dimension (in %) for technology groups

Barriers and facilitators were categorised by six technology groups (see Fig. 1), with each publication assigned based on its primary focus. Telehealth was the most researched group among the publications (n = 80 publications), followed by AI (n = 51), DiHA (n = 42), and General DHT (n = 39). Health Data Infrastructure (n = 23) and Conceptual/Niche DHT (n = 3) were studied less frequently.

Human & Social Dynamics emerged as the most frequently reported barrier category across technology groups, accounting for roughly half of all barriers, except in Conceptual/Niche DHT (32.4%). Facilitators in this domain were similarly prominent, ranging from 41.4% to 55.6%, though slightly lower in Health Data Infrastructure (29.1%) and AI (31.2%), reflecting their more technical and less user-facing nature.

Organisational Structure & Management barriers appeared more evenly, ranging from 21.0% to 27.7%, but were less frequent in Conceptual/Niche DHTs (16.2%). In contrast, facilitators in this dimension were particularly pronounced for Health Data Infrastructure (39.5%) and AI (31.5%), underscoring the importance of organisational alignment for scaling these technologies.

Infrastructure & Data Security barriers were most pronounced for Conceptual/Niche technologies (51.4%), highlighting the technical maturity gaps these innovations face. Among other groups, this dimension accounted for approximately one-quarter to one-third of reported barriers (24.9% to 31.1%). Facilitators in this area were relatively consistent across technologies (31.4% to 37.3%), except for Telehealth (20.1%) and Conceptual/Niche DHTs (22.2%), where infrastructure facilitators were less commonly reported. The lower proportion of reported technical barriers in Telehealth likely reflects its more established role in healthcare delivery. In contrast, the limited use and early-stage development of Conceptual/Niche DHTs may explain the lower reporting frequency observed for these technologies.

### Barriers and facilitators by stakeholder group

**Fig. 2:**
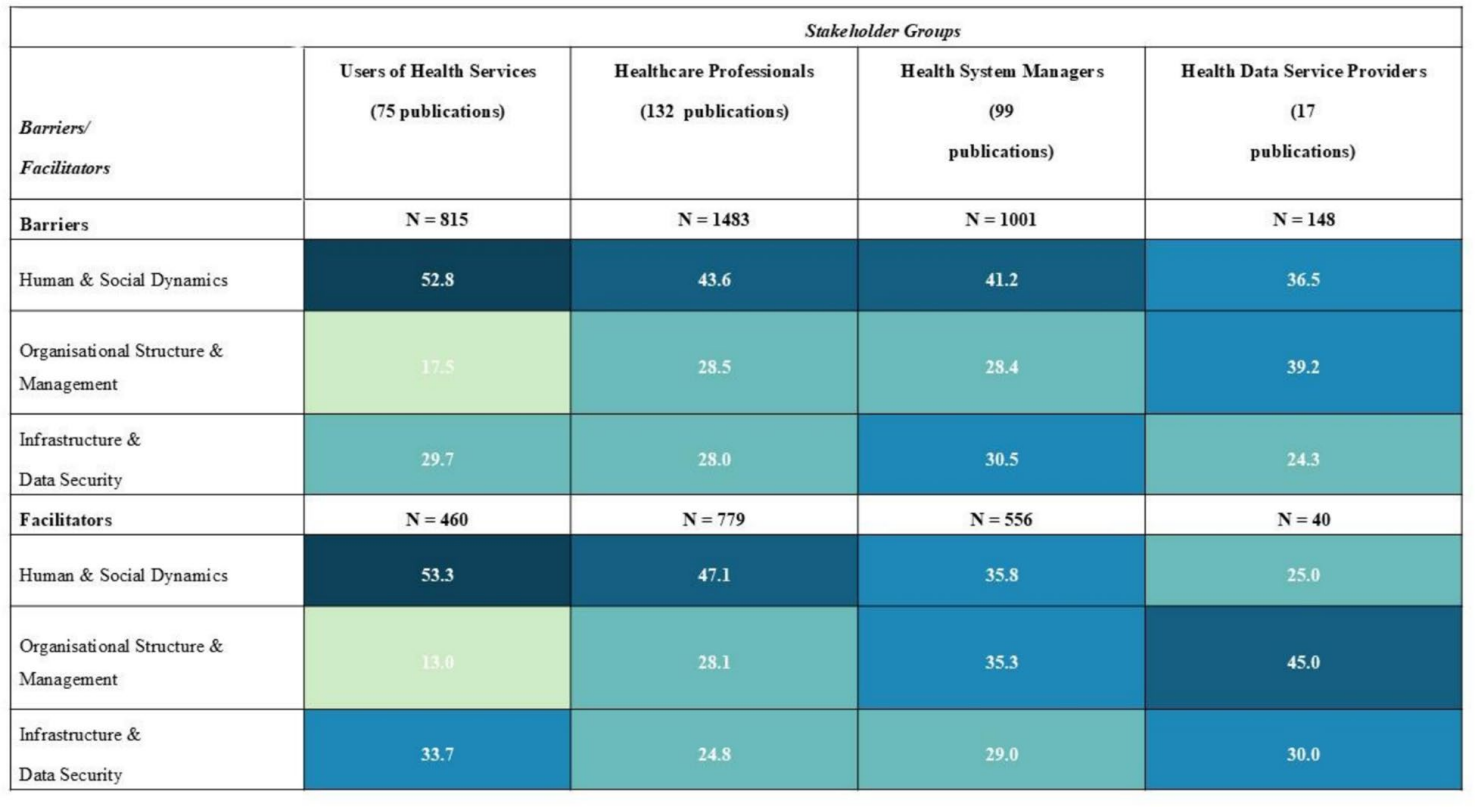
Distribution of barriers and facilitators by dimension (in %) for different stakeholder groups

Barriers and facilitators were further analysed across four stakeholder groups (see Fig. 2), with each publication assigned to one or more groups based on its research focus. Healthcare Professionals were the most studied group (n = 132), followed by Health System Managers (n = 99), Users of Health Services (n = 75), and Health Data Service Providers (n = 17).

Human & Social Dynamics barriers were the most prevalent across all groups, particularly among Users of Health Services (52.8%), decreasing to 36.5% for Health Data Service Providers. A similar trend was observed for facilitators in this dimension, with Users at 53.3% and Data Service Providers at 25.0%, underscoring the strong influence of behavioural factors on technology adoption.

Organisational Structure & Management barriers varied more widely, ranging from 17.5% among Users to 39.2% among Data Service Providers. Facilitators in this dimension followed the same pattern, wit*h* the highest share for Data Service Providers (45.0%) and the lowest for Users (13.0%), reflecting the differing relevance of governance and structural alignment across roles.

In the Infrastructure & Data Security dimension, the distribution of barriers and facilitators was more uniform across groups, with each group reporting approximately one-quarter to one-third of the total number of reported factors.

Overall, Human & Social Dynamics remained the most influential dimension in shaping the implementation of DHTs across stakeholder populations, particularly among end-users.

## Discussion

This scoping review synthesised findings from 238 publications reporting on the implementation of DHTs in high-income healthcare settings. Barriers and facilitators were categorised into three overarching dimensions: human and social dynamics, organisational structure and management, and infrastructure and data security. While each dimension reflects a distinct aspect of the implementation process, their interplay was evident across diverse technologies and stakeholder groups. Human and social factors emerged as dominant, but implementation success was also influenced by leadership, strategic resource management, and robust infrastructure.

Within human and social dynamics, common barriers included resistance to change, lack of motivation, and scepticism regarding the effectiveness of digital interventions. These findings are consistent with theoretical models such as the Unified Theory of Acceptance and Use of Technology (UTAUT) [22] the Diffusion of Innovation Theory (DOI) [23], as well as behavioural models that highlight the role of perceived usefulness, complexity, and attitudes in uptake [24, 25]. Conversely, enabling factors included trust in the technology [26], user-centred design [27], and training initiatives for patients and professionals [28, 29].

Organisational conditions played a central role, as reflected in the substantial share of identified barriers. Frequently reported challenges included limited financial resources, misaligned reimbursement structures, and skilled workforce shortages, reflecting broader systemic misalignments. Workflow incompatibility and fragmented service delivery further impeded progress. These dynamics align with the productivity paradox, whereby adoption may initially increase workloads before improving efficiency [30, 31]. High upfront costs, complex integration requirements, and the need for extensive training can delay the anticipated benefits of digitalisation [32]. Workforce shortages, burnout, and competing priorities also constrain capacity, even in well-resourced systems [33, 34]. In contrast, strong leadership supported success through strategic vision, active management, and the mobilisation of local champions and ‘super users’ [35, 36]. These findings highlight the need for organisations that are not only receptive to innovation but also structurally equipped to manage the additional demands of digital transformation.

Although infrastructure and data-related issues were reported less frequently, they remain essential to implementation. Usability challenges, including poor interface design and limited clinical functionality, continue to hinder uptake, particularly among users with accessibility needs [33]. Privacy concerns, cybersecurity risks, and legal uncertainties limit trust, particularly in the absence of harmonised governance [34]. While interoperability is often framed as a technical challenge, it also reflects deeper systemic fragmentation that impedes integration across care settings [37]. These challenges are more prominent in policy and industry literature than in academic publications, indicating an underrepresentation of regulatory complexity in research [38]. Technology-specific findings showed that user-facing tools, such as telehealth and DiHA, were shaped primarily by social factors, whereas technical and organisational challenges were more salient for AI and Health Data Infrastructure [26]. Telehealth, the most studied group, benefited from established clinical pathways [39], and widespread uptake during the COVID-19 pandemic [40]. In contrast, AI adoption remains slower due to algorithmic opacity, ethical concerns, and organisational readiness gaps. AI, despite its potential in diagnostics [41, 42], predictive analytics [43], and personalised medicine [43], faces persistent barriers related to complexity and regulation [11, 44]. Conceptual and niche DHTs, still in early development, face infrastructural limitations and limited investment, delaying integration [45].

Stakeholder-specific patterns further illustrate the complexity of DHT implementation. Human and social dynamics dominated across stakeholder groups, especially among users of health services, whose barriers and facilitators frequently centred on digital literacy, usability, and acceptance, reflecting their direct interaction with technologies [29, 46]. Healthcare professionals and health system managers showed a more balanced distribution across all dimensions, emphasising both organisational readiness and human-centred considerations. In contrast, health data service providers, with their predominantly technical orientation, reported fewer behavioural challenges but emphasised organisational and infrastructural considerations.

These stakeholder-specific differences reinforce the importance of distinguishing between adoption and implementation, terms often used interchangeably yet representing distinct stages of digital transformation. Adoption typically refers to the initial uptake of a technology, influenced primarily by user perceptions and attitudes [24, 25]. Implementation, by contrast, involves embedding technologies into established care processes, requiring alignment with clinical workflows, regulatory structures, and sustained engagement from healthcare professionals and patients [47].

Normalisation Process Theory (NPT) [41] provides a useful lens for understanding why many technologies fail to move beyond pilot phases. NPT conceptualises implementation as a continuous social process shaped by four mechanisms: coherence (how stakeholders make sense of a technology), cognitive participation (the work required to enrol and engage users), collective action (the operational work of integrating the technology into routines), and reflexive monitoring (the appraisal and adjustment of the technology over time, for instance in terms of benefits and costs) [41]. Several barriers identified in the Human and Social Dynamics and Organisational Structure and Management dimensions—such as resistance to change, limited literacy and training, workflow misalignment, and scepticism about effectiveness—reflect shortcomings across these mechanisms. While NPT helps explain micro- and meso-level dynamics, many barriers and facilitators identified in this review, such as insufficient funding, shortages of skilled workforce, and issues related to usability, interoperability, and data security, point to structural and systemic determinants that extend beyond the scope of NPT. To account for these influences, the findings could also be situated within the NASSS (Non-adoption, Abandonment, Scale-up, Spread, and Sustainability) framework, which conceptualises digital health implementation as shaped by complexity across seven interdependent domains: the condition or illness, the technology, the value proposition, the adopter system, the organisation, the wider institutional and societal context, and the interaction and mutual adaptation between these domains over time. This review’s three dimensions can be broadly related to these domains: Human and Social Dynamics overlap with the condition, value proposition, and adopter system domains; Organisational Structure and Management reflect the organisational and wider system domains; and Infrastructure and Data Security correspond primarily to the technology domain.

Connecting the findings of this review to established implementation concepts aligns the inductively derived dimensions with prior theoretical work and illustrates how the identified barriers and facilitators reflect recognised determinant patterns. NPT helps explain why many frequently reported human and social factors—such as limited engagement, difficulties integrating technologies into routines, and uncertainties regarding their value—pose challenges during early implementation. With its focus on interaction and mutual adaptation, the NASSS framework highlights the underlying complexity across these domains, consistent with the interrelated nature of the dimensions identified in this review.

The predominance of human and social factors in the literature raises the question of whether their perceived importance reflects actual implementation experiences or a publication bias. Research indicates that studies with positive outcomes are more likely to be published, while those with neutral or negative results are underrepresented, potentially skewing the available evidence [42]. This suggests that particular challenges, including organisational and infrastructural barriers, may be underreported. It is likely that all three dimensions—human, organisational, and infrastructural—interact dynamically, with their relevance varying by context and stage. Organisational and technical barriers may be equally influential but less frequently reported, particularly in user-centred or early-stage studies. This further supports the need to differentiate between adoption and implementation clearly. While individual-level dynamics shape adoption, implementation depends on system-wide alignment, including regulatory compliance, resourcing, and digital infrastructure. Addressing these dimensions in isolation risks superficial uptake without long-term integration. While technical and regulatory enablers are essential for implementation, sustained adoption appears to depend more strongly on human and social dynamics.

The findings have several implications for policy and practice. Effective digital transformation requires not only financial and technical support but also cultural change, engagement, and leadership-driven alignment between digital strategies and clinical priorities [48]. Top-down approaches can offer regulatory guidance, infrastructure, and incentives, but must be complemented by bottom-up engagement to ensure that technologies meet the needs of frontline users and local care contexts. A cultural shift away from the traditional “lonely doctor hero” model toward collaborative, team-based care supported by digital tools can strengthen acceptance and integration [49]. Alignment across different levels of the health system is essential. This includes coordination among policymakers, healthcare providers, and technology developers, as well as distributed responsibility for implementation across clinical and administrative roles. Leaders must navigate internal and system-level challenges, mediate resistance, and ensure compliance, while creating an environment conducive to digital transformation [50]. Consequently, effective implementation hinges on robust governance, proactive management [51], and recognition that cultural change—driven by both user engagement and institutional commitment—remains vital for sustained success [52].

### Strengths and limitations

A key strength of this review lies in its comprehensive and integrative approach. By synthesising findings across diverse study designs, stakeholder groups, and digital health technologies, it offers a broad overview of the most frequently reported barriers and facilitators in high-income healthcare settings. Methodological rigour was ensured through piloted extraction, dual coding, and structured classification of implementation factors. The categorisation of digital technologies also enabled a more nuanced understanding of variation across intervention types. Several limitations should be acknowledged. Limiting the review to English- and German-language publications may have excluded relevant evidence. Furthermore, the findings cannot be generalised to low- and middle-income settings, as they are based solely on upper-middle and high-income countries. As a result, important barriers and facilitators specific to LMICs may not be reflected in this review. Excluding certain publication types, such as conference abstracts and protocols, may have led to the omission of emerging research. In addition, the search was limited to two major databases and did not include grey literature, which may mean that some policy reports, evaluation studies, or institutional publications reporting additional barriers and facilitators were not captured. Some screening and extraction steps were conducted by a single reviewer, introducing potential bias. Lastly, as a scoping review, the analysis did not include a formal quality assessment, limiting conclusions about the strength of the underlying evidence.

### Future research

Despite its broad scope, this review highlights several gaps in the existing evidence base. The findings confirm that human factors such as motivation, communication, and trust are central to the adoption of digital health technologies. While much of the literature focuses on user-level barriers, future research should give greater attention to organisational structures, leadership practices, and governance mechanisms to better understand the conditions that support sustainable implementation. Existing frameworks such as UTAUT and CFIR [22, 52] provide useful conceptual guidance, yet their practical applicability remains limited when balancing centralised directives with local stakeholder involvement. In parallel, research on emerging technologies—including artificial intelligence and less mature digital health solutions—should examine not only technical performance but also ethical, operational, and regulatory implications. The growing number of AI-related implementation studies reflects the field's increasing relevance. Recent work, such as Nair et al. [53], which identifies critical organisational activities for the successful implementation of AI in healthcare, illustrates the need for process- and governance-oriented approaches to support the integration of increasingly complex digital technologies. Governance strategies also require further exploration, particularly with respect to aligning high-level policy objectives with the capacities and realities of frontline care. To support implementation in practice, future studies should also contribute to the development of concrete indicators that enable the operationalisation and monitoring of digital transformation efforts across contexts.

## Conclusions

This review highlights the multifaceted nature of digital health implementation, showing that success depends on more than technological readiness. Human and social dynamics, organisational capacity, and technical infrastructure each play a critical role, and their interaction determines whether digital innovations are meaningfully integrated into care delivery. While much of the literature focuses on user-level adoption, sustainable implementation also requires strategic leadership, adequate resources, supportive governance, and alignment across the health system. Advancing digital transformation will depend on interdisciplinary collaboration, user-centred design, and addressing infrastructural and regulatory challenges. Bridging the gap between policy ambitions and clinical practice remains a key challenge, but also a crucial opportunity to ensure that digital health technologies contribute to more equitable, efficient, and responsive healthcare systems.

## Supplementary Information

Additional file 1.

Additional file 2.

Additional file 3.

## Data Availability

All data generated or analysed during this study are included in this published article and its supplementary information files.

## Abbreviations

AI: Artificial Intelligence
CFIR: Consolidated Framework for Implementation Research
COVID-19: Coronavirus Disease 2019
DiHA: Digital Health Applications
DHT(s): Digital Health Technology / Technologies
DOI: Diffusion of Innovation Theory
GDHP: Global Digital Health Partnership
GDPR: General Data Protection Regulation
HIPAA: Health Insurance Portability and Accountability Act
HMIC(s): High-Income and Upper-Middle-Income Country / Countries
LMIC(s): Low- and Middle-Income Country / Countries
mHealth: Mobile Health
NASSS: Non-adoption, Abandonment, Scale-up, Spread and Sustainability
NPT: Normalisation Process Theory
OECD: Organisation for Economic Co-operation and Development
OSF: Open Science Framework
PRISMA-ScR: Preferred Reporting Items for Systematic Reviews and Meta-Analyses extension for Scoping Reviews
UTAUT: Unified Theory of Acceptance and Use of Technology
WHO: World Health Organization
