# Supplementary material for: Navigating the complexities of digital health technology implementation: a scoping review of barriers and facilitators
Source: Implement Sci Commun. 2026 Mar 4;7:69. doi: 10.1186/s43058-026-00892-4 (PMC13064259; doi:10.1186/s43058-026-00892-4)
Supplement: Supplementary file 2 — Additional file 2. [file 43058_2026_892_MOESM2_ESM.pdf]

Additional file 2 - Search string

PubMed

Date of search: 27.02.2024

("telemedicine"[MeSH Terms] OR "digital technology"[MeSH Terms] OR "Mobile Applications"[MeSH Terms] OR "Digital health"[Title/Abstract] OR "mHealth"[Title/Abstract] OR "m-health"[Title/Abstract] OR "Telehealth"[Title/Abstract] OR "Telecare"[Title/Abstract] OR "Digital health application"[Title/Abstract] OR "DiHA"[Title/Abstract] OR "web based intervention*"[Title/Abstract] OR "web-based intervention*"[Title/Abstract] OR "internet based intervention*"[Title/Abstract] OR "internet-based intervention*"[Title/Abstract] OR "artificial intelligence"[Title/Abstract] OR "medical artificial intelligence"[Title/Abstract] OR "medical AI"[Title/Abstract])
AND (("facilitator*"[Title/Abstract] OR "barrier*"[Title/Abstract] OR "enabler*"[Title/Abstract] OR "driver*"[Title/Abstract] OR "obstacle*"[Title/Abstract] OR "hurdle*"[Title/Abstract] OR (("facilitat*"[Title/Abstract] OR "enabl*"[Title/Abstract] OR "challeng*"[Title/Abstract] OR "encourag*"[Title/Abstract] OR "support*"[Title/Abstract] OR "enhanc*"[Title/Abstract] OR "promot*"[Title/Abstract] OR "hinder*"[Title/Abstract] OR "imped*"[Title/Abstract])
AND ("factor*"[Title/Abstract] OR "determinant*"[Title/Abstract])))
AND ("implement*"[Title/Abstract] OR "integrat*"[Title/Abstract] OR "diffus*"[Title/Abstract] OR "scale up"[Title/Abstract] OR "scale-up"[Title/Abstract] OR "roll out"[Title/Abstract] OR "roll-out"[Title/Abstract] OR "adopt*"[Title/Abstract])))
AND (2019:2024[pdat])

Scopus

Date of search: 27.02.2024

(TITLE-ABS(eHealth OR telemedicine OR "digital technology" OR "Mobile Applications" OR "Digital health" OR mHealth OR m-health OR Telehealth OR Telecare OR "Digital health application" OR DiHA OR "web-based intervention*" OR "web based intervention*" OR "internet-based intervention*" OR "internet based intervention*" OR "artificial intelligence" OR "medical artificial intelligence" OR "medical AI"))
AND ((TITLE-ABS("facilitator*" OR "barrier*" OR "enabler*" OR "driver*" OR "obstacle*" OR "hurdle*")) OR ((TITLE-ABS("facilitat*" OR "enabl*" OR "challeng*" OR "encourag*" OR "support*" OR "enhanc*" OR "promot*" OR "hinder*" OR "imped*")) AND (TITLE-ABS("factor*" OR "determinant*"))))
AND (TITLE-ABS(“implement*” OR “integrat*” OR “diffus*” OR "scale-up" OR "scale up" OR "roll-out" OR "roll out" OR adopt*))
AND PUBYEAR > 2018 AND PUBYEAR < 2025
