# Supplementary material for: Navigating the complexities of digital health technology implementation: a scoping review of barriers and facilitators
Source: Implement Sci Commun. 2026 Mar 4;7:69. doi: 10.1186/s43058-026-00892-4 (PMC13064259; doi:10.1186/s43058-026-00892-4)
Supplement: Supplementary file 3 — Additional file 3. [file 43058_2026_892_MOESM3_ESM.pdf]

Additional file Table 1: Technology groups considered in this scoping review as defined by the authors

Technology group	Description
Health Data Infrastructure	Systems and technologies designed to manage, store, and enable the use of electronic health data, such as electronic health records (EHRs), and large-scale health data integration platforms. These technologies prioritise data accessibility, interoperability, and security, providing a foundation also for advanced healthcare applications.
Telehealth	Broad range of technologies that facilitated the remote delivery of healthcare, including diagnosis, treatment, and monitoring. It encompassed, for instance, telemedical services such as video consultations, telemonitoring, and telerehabilitation (remote physical therapy and rehabilitation programs), to name a few.
Digital Health Applications (DiHA)	Specific digital tools, such as mHealth apps and platforms, designed primarily for patient use to support health-related tasks. DiHA focus on managing chronic diseases, patient self-management, and medication adherence. While primarily patient-centered, some DiHA may also provide features for healthcare providers, such as data sharing or reports to assist in clinical decision-making.
Artificial Intelligence (AI)	Use of AI algorithms, machine learning, and IoT-based systems in healthcare to enhance diagnostics, treatment planning, clinical decision support tools, and process automation. These technologies leverage predictive analytics, natural language processing, and advanced data modelling.
General DHT	Digital technologies and innovations that were not further defined and that did not fit neatly into other categories. These may involve platforms for eHealth, digital health policy, or interventions targeting digital equity and inclusion.
Conceptual or Niche DHT	Emerging or less-defined areas of healthcare technology, such as conceptual frameworks (e.g., Health 4.0), novel digital strategies, or niche innovations like augmented reality or specialised wearables. These technologies often explore new paradigms without widespread adoption.

Additional file Table 2: Study populations considered in this scoping review

Stakeholder group	Description
Users of Health Services	"The taxonomy in this group describes the capabilities of digital technology that can be implemented to achieve objectives that are targeted toward persons. Persons include members of the public who are potential or current users of health services and caregivers of individuals receiving health services." (World Health Organisation 2023, p.10)<br>Examples of data services identified in the reviewed studies include healthcare recipients, such as patients, pregnant women, the elderly, and general citizens.
Healthcare Providers	"The taxonomy in this group describes the capabilities of digital technology that can be implemented to achieve objectives that are targeted toward healthcare providers. Healthcare providers are members of the health workforce who deliver health interventions." (World Health Organisation 2023, p.17)<br>Examples of Healthcare Providers identified in the reviewed studies include hospitals, rehabilitation centres, nursing homes, and the health workforce, including physicians, nurses, therapists, and pharmacists.
Health System Managers	"The taxonomy in this group describes the capabilities of digital technology that can be implemented to achieve objectives that are targeted toward health management and support personnel. Health management and support personnel are involved in the administration and oversight of health systems." (World Health Organisation 2023, p.27)<br><br>Examples of Health System Managers identified in the reviewed studies include stakeholders and structures such as the general population, governance, health insurance, hospital leaders, and research and education entities.
Health Data Infrastructure	Examples of Data Services identified in the reviewed studies include software developers, start-ups, medical device manufacturers, and pharmaceutical companies.

Additional file Figure 1: Flow diagram of the selection process

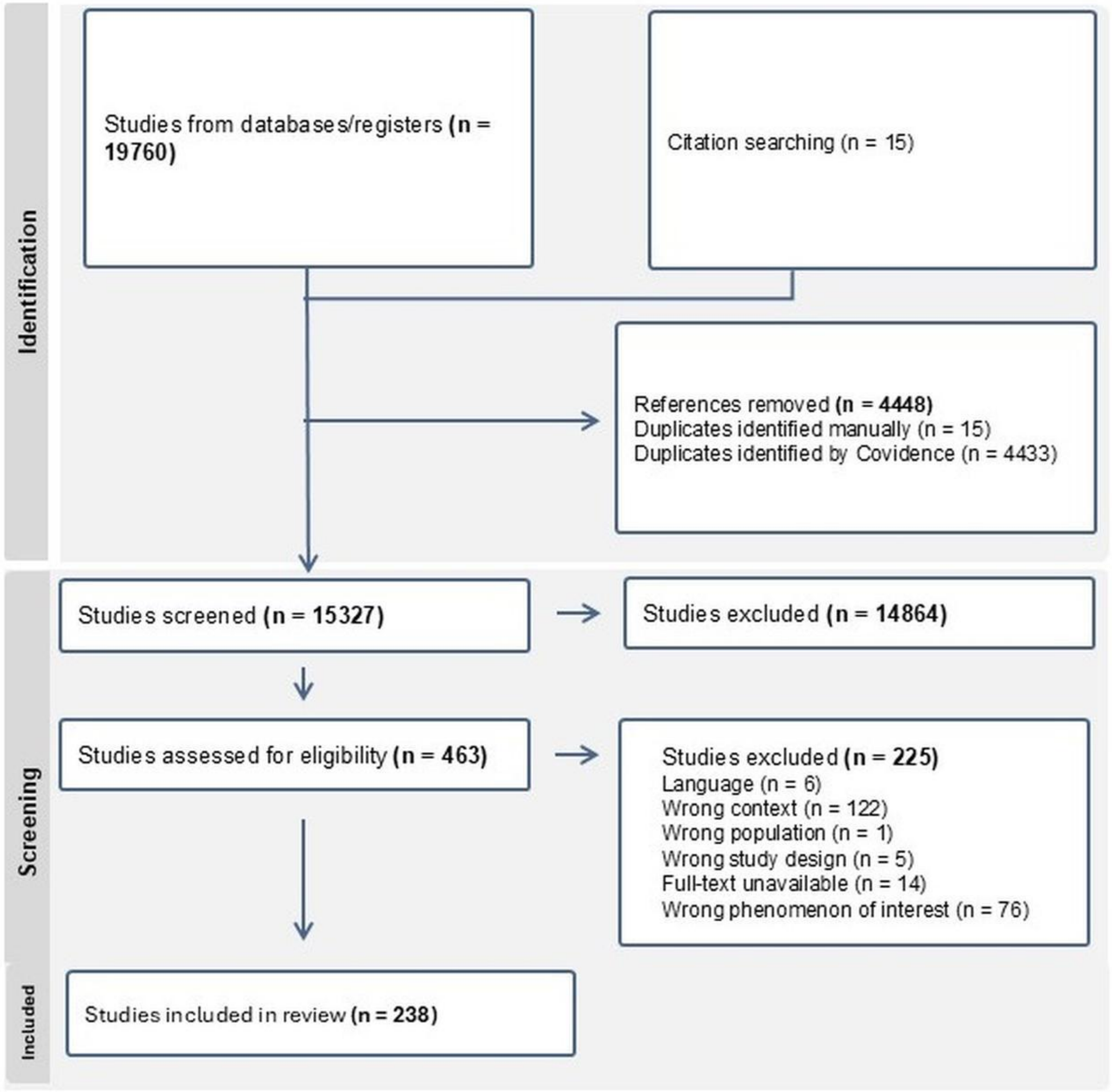

Additional file Table 3: List of excluded publications and reasons

Study	Notes
Aalami 2023	Wrong phenomenon of interest
Aamir 2024	Wrong phenomenon of interest
Abdella 2023	Wrong context
Acosta-Gomez 2023	Wrong phenomenon of interest
Adams 2021	Wrong phenomenon of interest
Addotey-Delove 2023	Wrong context
Adedeji 2023	Wrong context
Afrizal 2020	Wrong context
Ahmed 2023	Wrong context
Ajmera 2019	Wrong context
Akwaowo 2022	Wrong context
AlMeslamani 2022	Wrong phenomenon of interest
Alanezi 2021	Wrong phenomenon of interest
Al-Anezi 2020	Wrong phenomenon of interest
Albaghdadi 2023	Wrong phenomenon of interest
Aldughayfiq 2021	Wrong context
Aloini 2023	Wrong phenomenon of interest
Al-Otaibi 2022	Wrong context
Al-Qerem 2023	Wrong context
Al-Sharhan 2019	Wrong phenomenon of interest
Alsheiabni 2019	Wrong study design
Alviani 2023	Wrong context
Ames 2019	Wrong phenomenon of interest
Asah 2022	Wrong context
Assaye 2022	Wrong context
Asthana 2023	Wrong context
Badran 2019	Wrong context
Bajrić 2023	Wrong phenomenon of interest
Baker 2022	Full-text unavailable
Balch 2023	Wrong phenomenon of interest
Balla 2023	Wrong phenomenon of interest
Baltaxe 2019	Wrong phenomenon of interest
Baxter 2020	Wrong phenomenon of interest
Bekyieriya 2023	Wrong context
Bentahar 2021	Language; original in french
Berihun 2020	Wrong context
Bilal 2022	Wrong context
Bilal 2022	Wrong context
Blumenberg 2022	Wrong phenomenon of interest
Bogomiagkova 2022	Language; original in russian
Bonnechère 2023	Wrong context
Borscheva 2021	Wrong phenomenon of interest
Cady 2023	Language; original in french
Cascini 2023	Wrong phenomenon of interest
CernadasRamos 2020	Language; article in spanish
CernadasRamos 2022	Wrong phenomenon of interest
Chaibi 2022	Wrong context
Chetthamrongchai 2019	Wrong context
Chirambo 2019	Wrong context
Chowdhury 2021	Wrong context
Ciecierski-Holmes 2022	Wrong context
Cobelli 2020	Wrong phenomenon of interest
Cook 2023	Wrong phenomenon of interest
Cortelyou-Ward 2020	Wrong context
Cremers 2021	Wrong phenomenon of interest
Dang 2020	Wrong context
Dang 2021	Wrong context
Dansharif 2021	Wrong context
Das 2022	Wrong context
deArteche 2020	Language; original in Spanish
Dehart 2022	Full-text unavailable

Demeke 2023	Wrong context
Deniz-Garcia 2023	Wrong phenomenon of interest
Dhaliwal 2022	Full-text unavailable
Dharmayat 2019	Wrong context
Dhyani 2023	Wrong context
Dodoo 2022	Wrong context
Dohrn 2023	Full-text unavailable
Donida 2021	Wrong phenomenon of interest
Dykes 2021	Full-text unavailable
Eckhoff 2022	Wrong phenomenon of interest
Eddison 2022	Wrong phenomenon of interest
El-Halabi 2021	Wrong context
Fanta 2023	Wrong phenomenon of interest
Faujdar 2021	Wrong context
Feroz 2021	Wrong context
Ferreira-Brito 2024	Wrong phenomenon of interest
Fleßa 2021	Wrong phenomenon of interest
Fry 2020	Wrong context
Garber 2022	Wrong phenomenon of interest
Gochhait 2020	Wrong phenomenon of interest
Gu 2023	Wrong phenomenon of interest
Halwani 2021	Wrong context
Hameed 2023	Wrong phenomenon of interest
Hampshire 2021	Wrong context
Han 2019	Wrong context
Harris 2021	Full-text unavailable
Hawa 2023	Wrong phenomenon of interest
Haynes 2021	Wrong phenomenon of interest
Hengst 2023	Wrong context
Hilty 2021	Wrong phenomenon of interest
Hong 2019	Wrong context
Huang 2019	Wrong context
Huynh 2023	Wrong phenomenon of interest
Ikwunne 2023	Wrong context
Iliyasu 2023	Wrong context
Islami 2022	Wrong context
Jabarethina 2023	Wrong context
Jain 2020	Wrong context
Jangle 2023	Wrong context
Kaboré 2022	Wrong context
Kamulegeya 2021	Wrong context
Kanavos 2022	Wrong phenomenon of interest
Karaca 2022	Language; original in turkish
Karachay 2021	Wrong phenomenon of interest
Karlyn 2020	Wrong context
Kazi 2020	Wrong context
Khoda 2022	Wrong context
King 2023	Wrong phenomenon of interest
Kirk 2021	Wrong context
Kirkland 2021	Wrong phenomenon of interest
Koumpouros 2020	Wrong phenomenon of interest
Kowatsch 2019	Wrong phenomenon of interest
Kruse 2019	Wrong context
Kučera 2020	Full-text unavailable
Kumar 2022	Wrong context
Kumar 2024	Wrong context
Labrague 2023	Wrong context
Lacroze 2023	Wrong context
Laing 2021	Wrong context
Layer 2023	Wrong context
Le 2023	Wrong phenomenon of interest
Leader 2023	Wrong phenomenon of interest
Li 2021	Wrong phenomenon of interest
Lima 2021	Wrong context

Luciano 2020	Wrong context
Madanian 2019	Wrong context
Maini 2020	Wrong context
Maliwichi 2022	Wrong context
Maliwichi 2021	Wrong context
Mamuye 2023	Wrong context
Manyati 2021	Wrong context
Maroju 2023	Wrong context
Mather 2019	Wrong phenomenon of interest
Mbunge 2022	Wrong context
Medaglia 2022	Wrong context
Mehregany 2020	Full-text unavailable
Melchiorre 2020	Wrong phenomenon of interest
Meng 2019	Wrong phenomenon of interest
Mengiste 2022	Wrong context
Mensah 2023	Wrong context
Metzger 2021	Wrong phenomenon of interest
Mishra 2023	Wrong phenomenon of interest
Mohammadzadeh 2022	Full-text unavailable
Muller 2020	Wrong context
Musa 2023	Wrong context
Nabukenya 2023	Wrong context
Namatovu 2023	Wrong context
Namatovu 2022	Wrong context
Ncube 2023	Wrong context
Ng 2023	Wrong phenomenon of interest
Ngongo 2019	Wrong context
Nittas 2024	Wrong phenomenon of interest
Nkwanyana 2022	Wrong study design
Octavius 2021	Wrong context
Oduor 2021	Wrong context
Oduoye 2024	Wrong context
Ogundaini 2021	Wrong context
Ohia 2021	Wrong context
Olaza-Maguiña 2022	Wrong context
Onsongo 2023	Wrong context
Osei 2021	Wrong context
Osman 2023	Wrong context
Øvretveit 2019	Wrong phenomenon of interest
Owolabi 2022	Wrong context
Paliwal 2023	Wrong context
Pan 2019	Wrong phenomenon of interest
Parajuli 2022	Wrong context
Patel 2022	Wrong phenomenon of interest
Peer 2022	Wrong phenomenon of interest
Poulsen 2024	Wrong context
Rachmawati 2022	Wrong phenomenon of interest
Rajkumar 2023	Wrong context
Ramachandran 2021	Wrong context
Ramaswamy 2022	Wrong context
Ross 2019	Full-text unavailable
Russkikh 2023	Full-text unavailable
Rydenfält 2019	Full-text unavailable
Sagaro 2020	Wrong context
Said 2023	Wrong phenomenon of interest
Saini 2023	Wrong phenomenon of interest
Saiso 2021	Wrong context
Schierhout 2021	Wrong context
Schlieter 2022	Wrong study design
Schroeder 2024	Wrong phenomenon of interest
Schuetze 2023	Wrong context
Semple 2019	Wrong phenomenon of interest
Shardha 2024	Wrong context
Sharma 2019	Wrong context

Shiferaw 2020	Wrong context
Sikarwar 2022	Wrong context
Sides 2023	Wrong study design
Sritart 2021	Wrong context
Sticca 2020	Full-text unavailable
Swain 2024	Wrong context
Sy 2020	Wrong context
Těšinová 2022	Full-text unavailable
Tolera 2022	Wrong context
Triplett 2023	Wrong context
Truong 2023	Wrong context
Tucci 2022	Wrong phenomenon of interest
Tuvesson 2020	Wrong phenomenon of interest
Upadhyay 2023	Wrong context
Upshaw 2023	Wrong phenomenon of interest
Valente 2022	Wrong population
vanHaeften 2021	Wrong study design
vanKessel 2023	Wrong phenomenon of interest
vanOlmen 2020	Wrong context
Venkataraghavan 2021	Wrong context
Venkataraman 2024	Wrong context
Versluis 2020	Wrong phenomenon of interest
vonKalckreuth 2023	Wrong phenomenon of interest
Wagaba 2023	Wrong context
Weißenfeld 2021	Wrong phenomenon of interest
Wen 2022	Full-text unavailable
Wong 2023	Wrong phenomenon of interest
Wu 2024	Wrong phenomenon of interest
Wubante 2022	Wrong context
Yu-tong 2022	Wrong phenomenon of interest
Zehra 2021	Wrong phenomenon of interest
Zemplényi 2023	Wrong phenomenon of interest
Zhen 2023	Wrong phenomenon of interest
Zobair 2019	Wrong context
Zobair 2020	Wrong context
Zobair 2020	Wrong context

Additional file Table 4: Overview of publications included and their characteristics

PUBLICATION	TITLE	DOCUMENT TYPE	TOTAL NUMBER OF PARTICIPANTS/ INCLUDED STUDIES	SEARCH PERIOD/ STUDY PERIOD	COUNTRY	REGION	STUDY POPULATION	TECHNOLOGY
AbdRazak 2020	Investigating the Barriers of Health Information System implementation in Malaysian Public Hospitals	Qualitative	4	not reported	Malaysia	East Asia and Pacific	Healthcare Professionals; Health Data Service Providers	Health Data Infrastructure
Abell 2023	Identifying barriers and facilitators to successful implementation of computerized clinical decision support systems in hospitals: a NASSS framework-informed scoping review.	Review	44	04/2022	N/A	N/A	Healthcare Professionals	Health Data Infrastructure
Abels 2019	Computational pathology definitions, best practices, and recommendations for regulatory guidance: a white paper from the Digital Pathology Association.	Qualitative	not relevant	not reported	N/A	N/A	Health System Managers	Artificial intelligence
AbHamid 2023	Utilisation of Health Information Technology among Dietitians in the workplace: A qualitative study	Qualitative	15	09/2020 - 12/2020	Malaysia	East Asia and Pacific	Healthcare Professionals	General Digital Health Technology
Aggarwal 2022	Defining the Enablers and Barriers to the Implementation of Large-scale, Health Care-Related Mobile Technology: Qualitative Case Study in a Tertiary Hospital Setting.	Qualitative	14	not reported	United Kingdom	Europe and Central Asia	Healthcare Professionals; Health System Managers; Health Data Service Providers	DiHA
Ahmed 2023	A Systematic Review of the Barriers to the Implementation of	Review	59	12/2021	United Kingdom	Europe and Central	Health System Managers	Artificial intelligence

PUBLICATION	TITLE	DOCUMENT TYPE	TOTAL NUMBER OF PARTICIPANTS/ INCLUDED STUDIES	SEARCH PERIOD/ STUDY PERIOD	COUNTRY	REGION	STUDY POPULATION	TECHNOLOGY
	Artificial Intelligence in Healthcare.					Asia		
Alanazi 2023	Clinicians' Views on Using Artificial Intelligence in Healthcare: Opportunities, Challenges, and Beyond.	Qualitative	26	not reported	Saudi Arabia	Middle East and North Africa	Healthcare Professionals	Artificial intelligence
Alanzi 2023	Barriers and Facilitators of Artificial Intelligence in Family Medicine: An Empirical Study With Physicians in Saudi Arabia.	Quantitative	157	not reported	Saudi Arabia	Middle East and North Africa	Healthcare Professionals	Artificial intelligence
AlBadi 2022	Challenges of AI Adoption in the UAE Healthcare	Qualitative	27	not reported	UAE	Middle East and North Africa	Healthcare Professionals	Artificial intelligence
Al-Dhahir 2023	An overview of facilitators and barriers in the development of eHealth interventions for people of low socioeconomic position: A Delphi study	Mixed-methods	27 (first round), 19 (second round)	04/2020 - 04/2021	Netherlands	Europe and Central Asia	Healthcare Professionals; Health System Managers ; Health Data Service Providers	General Digital Health Technology
Aljohani 2019	Adoption of M-Health Applications: The Saudi Arabian Healthcare Perspectives	Qualitative	N/A	not reported	Saudi Arabia	Middle East and North Africa	Health System Managers	DiHA
Alkureishi 2021	Clinician perspectives on telemedicine: Observational cross-sectional study	Qualitative	400	07/2020	United States	North America	NA	Telehealth
Almathami 2020	Barriers and facilitators that influence telemedicine-based, real-time, online consultation at patients' homes:	Review	45	2008 - 2018	N/A	N/A	Users of Health Services	Telehealth

PUBLICATION	TITLE	DOCUMENT TYPE	TOTAL NUMBER OF PARTICIPANTS/ INCLUDED STUDIES	SEARCH PERIOD/ STUDY PERIOD	COUNTRY	REGION	STUDY POPULATION	TECHNOLOGY
	Systematic literature review							
Aloyuni 2020	Knowledge, attitude, and barriers to telerehabilitation-based physical therapy practice in Saudi Arabia	Quantitative	347	not reported	Saudi Arabia	Middle East and North Africa	Healthcare Professionals	Telehealth
Alpert 2023	Identifying barriers and facilitators for using a smartwatch to monitor health among older adults	Qualitative	22	not reported	United States	North America	Health System Managers	DiHA
Alqurashi 2023	The Perception of Health Care Practitioners Regarding Telemedicine During COVID-19 in Saudi Arabia: Mixed Methods Study	Mixed-methods	81	not reported	Saudi Arabia	Middle East and North Africa	Healthcare Professionals	Telehealth
Al-rawashdeh 2022	IoT Adoption and Application for Smart Healthcare: A Systematic Review	Review	22	2015 - 2021	N/A	N/A	Users of Health Services; Healthcare Professionals	Artificial intelligence
Al-Samarraie 2020	Telemedicine in Middle Eastern countries: Progress, barriers, and policy recommendations	Review	43	not reported	multiple countries	Multiple countries	Health System Managers	Telehealth
Alshahrani 2019	A systematic review of the adoption and acceptance of eHealth in Saudi Arabia: Views of multiple stakeholders	Review	15	1993 - 2017	Saudi Arabia	Middle East and North Africa	Healthcare Professionals	General Digital Health Technology
Alsobhi 2022	Facilitators and Barriers of Artificial Intelligence Applications in Rehabilitation: A Mixed-Method Approach	Mixed-methods	236	05/2021	N/A	N/A	Healthcare Professionals	Artificial intelligence
Alsswey 2021	mHealth technology utilization in the Arab world : a systematic	Review	31	01/2008 - 05/2018	UAE	Middle East and North	Health System Managers	DiHA

PUBLICATION	TITLE	DOCUMENT TYPE	TOTAL NUMBER OF PARTICIPANTS/ INCLUDED STUDIES	SEARCH PERIOD/ STUDY PERIOD	COUNTRY	REGION	STUDY POPULATION	TECHNOLOGY
	review of systems, usage, and challenges					Africa		
Alzghaibi 2023	An examination of large-scale electronic health records implementation in Primary Healthcare Centers in Saudi Arabia: a qualitative study	Qualitative	17	not reported	Saudi Arabia	Middle East and North Africa	Healthcare Professionals	Health Data Infrastructure
Antonacci 2023	Healthcare professional and manager perceptions on drivers, benefits, and challenges of telemedicine: results from a cross-sectional survey in the Italian NHS	Quantitative	124	06/2021 - 09/2021	Italy	Europe and Central Asia	Healthcare Professionals	Telehealth
Aranha 2021	Exploring the Barriers and Facilitators which Influence mHealth Adoption among Older Adults: A Literature Review	Review	23	01/2010 - 12/2020	N/A	N/A	Users of Health Services	DiHA
Ashtarian 2023	Popular diffusion as an instrument for overcoming barriers to digital health in Iran: the critical role of the pandemic	Qualitative	15	08/2022 - 10/2022	Iran	Middle East and North Africa	Health System Managers; Health Data Service Providers	General Digital Health Technology
Babaei 2023	A scoping review of virtual care in the health system: infrastructures, barriers, and facilitators	Review	20	2017 - 2022	N/A	N/A	Health System Managers	Telehealth
Baines 2021	Meaningful patient and public involvement in digital health innovation, implementation and evaluation: A systematic review	Review	433	2010 - 07/2020	multiple countries	N/A	Users of Health Services	General Digital Health Technology
Balasubramanian	Applying artificial	Qualitative	58 review, 12 case	06/2022	UAE	Middle	Health System	Artificial

PUBLICATION	TITLE	DOCUMENT TYPE	TOTAL NUMBER OF PARTICIPANTS/ INCLUDED STUDIES	SEARCH PERIOD/ STUDY PERIOD	COUNTRY	REGION	STUDY POPULATION	TECHNOLOGY
2023	intelligence in healthcare: lessons from the COVID-19 pandemic		study			East and North Africa	Managers	intelligence
Bally 2020	Toward Integration of mHealth in Primary Care in the Netherlands: A Qualitative Analysis of Stakeholder Perspectives	Qualitative	23	05/2017 - 09/2018	Netherlands	Europe and Central Asia	Users of Health Services ;Healthcare Professionals; Health System Managers	DiHA
Baradwan 2023	Perceived Knowledge, Attitudes, and Barriers Toward the Adoption of Telemedicine Services in the Kingdom of Saudi Arabia: Cross-Sectional Study	Qualitative	1024	06/2022 - 07/2022	Saudi Arabia	Middle East and North Africa	Health System Managers	Telehealth
Bele 2021	Using the Theoretical Domains Framework to Identify Barriers and Enablers to Implementing a Virtual Tertiary-Regional Telemedicine Rounding and Consultation for Kids (TRaC-K) Model: Qualitative Study	Review	42	11/2017 - 08/2018	Canada	North America	Users of Health Services; Healthcare Professionals	Telehealth
Bertolazzi 2024	Barriers and facilitators to health technology adoption by older adults with chronic diseases: an integrative systematic review	Review	29	01/2012 - 04/2022	N/A	N/A	Users of Health Services	General Digital Health Technology
Blandi 2023	The potential of digital health records for public health research, policy, and practice: the case of the Lombardy region data warehouse	Qualitative	not relevant	not relevant	Italy	Europe and Central Asia	Health System Managers	Health Data Infrastructure
Blondino 2024	The use and potential	Quantitative	1,141	11/2022 - 05/2023	multiple	Multiple	Healthcare	General Digital

PUBLICATION	TITLE	DOCUMENT TYPE	TOTAL NUMBER OF PARTICIPANTS/ INCLUDED STUDIES	SEARCH PERIOD/ STUDY PERIOD	COUNTRY	REGION	STUDY POPULATION	TECHNOLOGY
	impact of digital health tools at the community level: results from a multi-country survey of community health workers.				countries	countries	Professionals	Health Technology
BorgesdoNascimento 2023	Barriers and facilitators to utilizing digital health technologies by healthcare professionals	Review	108	inception to 03/2023	N/A	N/A	Healthcare Professionals	General Digital Health Technology
Butt 2023	5G and IoT for Intelligent Healthcare: AI and Machine Learning Approaches—A Review	Review	7	not reported	N/A	N/A	Health System Managers	Artificial intelligence
Byambasuren 2019	Current knowledge and adoption of mobile health apps among Australian general practitioners: Survey study	Quantitative	1,014	10/2017 - 12/2017	Australia	East Asia and Pacific	Healthcare Professionals	DiHA
Byambasuren 2020	Barriers to and facilitators of the prescription of mhealth apps in Australian general practice: Qualitative study	Review	35	07/2017 - 12/2017	Australia	East Asia and Pacific	Users of Health Services ;Healthcare Professionals	DiHA
Calleja 2021	Telehealth use in rural and remote health practitioner education: an integrative review	Review	60	2007 - now	multiple countries	Multiple countries	Healthcare Professionals; Health System Managers	Telehealth
Cannavacciuolo 2022	Digital innovation and organizational changes in the healthcare sector: Multiple case studies of telemedicine project implementation	Qualitative	4	not reported	Italy	Europe and Central Asia	Healthcare Professionals	Telehealth
Carlqvist 2021	Health care professionals' experiences of how an eHealth application can	Qualitative	13	03/2020 - 11/2020	Sweden	Europe and Central	Healthcare Professionals	DiHA

PUBLICATION	TITLE	DOCUMENT TYPE	TOTAL NUMBER OF PARTICIPANTS/ INCLUDED STUDIES	SEARCH PERIOD/ STUDY PERIOD	COUNTRY	REGION	STUDY POPULATION	TECHNOLOGY
	function as a value-creating resource - a qualitative interview study					Asia		
Cascini 2023	Strengthening and promoting digital health practice: results from a Global Digital Health Partnership's survey	Quantitative	29 countries	03/2021 - 04/2021	multiple countries	Multiple countries	Health System Managers	General Digital Health Technology
Chan-Nguyen 2022	Patient and caregiver perspectives on virtual care: a patient-oriented qualitative study.	Review	18	11/2020 - 12/2020	Canada	North America	Users of Health Services; Healthcare Professionals	Telehealth
Chibuike 2024	Overcoming Challenges for Improved Patient-Centric Care: A Scoping Review of Platform Ecosystems in Healthcare	Review	20	2013 - 2023	N/A	N/A	Health System Managers	General Digital Health Technology
Chomutare 2022	Artificial Intelligence Implementation in Healthcare: A Theory-Based Scoping Review of Barriers and Facilitators	Review	19	2015 - 2021	multiple countries	Multiple countries	Health System Managers	Artificial intelligence
Choukou 2021	Digital health technology for Indigenous older adults: A scoping review	Review	26	no limitation	N/A	N/A	Users of Health Services	General Digital Health Technology
Chua 2023	Facilitators and barriers to implementation of telemedicine in nursing homes: A qualitative systematic review and meta-aggregation	Review	10	not reported	N/A	N/A	Healthcare Professionals	Telehealth
Chueke 2023	Persisting Barriers to the Adoption of Telemedicine in Latin America After the COVID-19 Pandemic	Qualitative	not relevant	not reported	Brazil, Chile, Colombia, Mexico	Latin America and the Caribbean	Health System Managers	Telehealth
Clark 2021	Understanding the Needs	Quantitative	17	2020	United	North	Healthcare	Telehealth

PUBLICATION	TITLE	DOCUMENT TYPE	TOTAL NUMBER OF PARTICIPANTS/ INCLUDED STUDIES	SEARCH PERIOD/ STUDY PERIOD	COUNTRY	REGION	STUDY POPULATION	TECHNOLOGY
	and Values of Rehabilitation Therapists in Designing and Implementing Telehealth Solutions				States	America	Professionals	
Cole 2019	Report on the use of telehealth in early intervention in Colorado: Strengths and challenges with telehealth as a service delivery method	Qualitative	161	not reported	United States	North America	Healthcare Professionals	Telehealth
Curtis 2020	Digital health technology: Factors affecting implementation in nursing homes	Qualitative	20	2018	United Kingdom	Europe and Central Asia	Users of Health Services; Healthcare Professionals	General Digital Health Technology
Dahlhausen 2021	Physicians' attitudes toward prescribable mHealth apps and implications for adoption in Germany: Mixed methods study	Mixed-methods	18 interviews, 1,308 survey responses	12/2020 - 01/2021	Germany	Europe and Central Asia	Healthcare Professionals	DiHA
Darcel 2023	Implementing artificial intelligence in Canadian primary care: Barriers and strategies identified through a national deliberative dialogue	Qualitative	49	09/2020 - 10/2020	Canada	North America	Users of Health Services; Healthcare Professionals; Health System Managers	Artificial intelligence
DeHart 2022	Benefits and Challenges of Telehealth Use during COVID-19: Perspectives of Patients and Providers in the Rural South	Qualitative	31	2021	United States	North America	Users of Health Services	Telehealth
Denecke 2023	Digital health as an enabler for hospital@home: A rising trend or just a vision?	Review	42	2013 - 2022	N/A	N/A	Healthcare Professionals	Telehealth
Depuccio 2021	Making It Work: Physicians' Perspectives	Qualitative	20	07/2020 - 08/2020	United States	North America	Healthcare professionals	Telehealth

PUBLICATION	TITLE	DOCUMENT TYPE	TOTAL NUMBER OF PARTICIPANTS/ INCLUDED STUDIES	SEARCH PERIOD/ STUDY PERIOD	COUNTRY	REGION	STUDY POPULATION	TECHNOLOGY
	on the Rapid Transition to Telemedicine							
Desveaux 2019	Identifying and overcoming policy-level barriers to the implementation of digital health innovation: Qualitative study	Review	10	not reported	Canada	North America	Health System Managers	General Digital Health Technology
Dimitrova 2023	Digital health in Bulgaria: Imagination or possible reality?	Mixed-methods	380	not reported	Bulgaria	Europe and Central Asia	Users of Health Services; Healthcare Professionals; Health System Managers; Health Data Service Providers	General Digital Health Technology
Dinh 2023	Perceptions About Augmented Reality in Remote Medical Care: Interview Study of Emergency Telemedicine Providers	Qualitative	21	not reported	United States	North America	Healthcare Professionals	Artificial intelligence
Dwivedi 2021	Potential of Internet of Medical Things (IoMT) applications in building a smart healthcare system: A systematic review	Review	165	2005 - 2020	N/A	N/A	Health System Managers	Artificial intelligence
Early 2019	Use of Mobile Health (mHealth) Technologies and Interventions Among Community Health Workers Globally: A Scoping Review	Review	64	2007 - 2018	multiple countries	Multiple countries	Healthcare Professionals	DiHA
Eigner 2019	Success factors for national eHealth strategies: A comparative analysis of the Australian and German eHealth system	Review	not relevant	not reported	Germany; Australia	East Asia and Pacific	Health System Managers	General Digital Health Technology
Eisapareh 2022	Facilitators and Barriers	Qualitative	17	until 12/2020	N/A	N/A	Users of Health	DiHA

PUBLICATION	TITLE	DOCUMENT TYPE	TOTAL NUMBER OF PARTICIPANTS/ INCLUDED STUDIES	SEARCH PERIOD/ STUDY PERIOD	COUNTRY	REGION	STUDY POPULATION	TECHNOLOGY
	to Using mHealth from Users’ Attitudes: A Qualitative Meta-synthesis						Services	
Esmaeilzadeh 2021	Patients' perceptions toward human-artificial intelligence interaction in health care: Experimental study	Quantitative	634	not reported	N/A	N/A	Users of Health Services	Artificial intelligence
Eze 2020	Telemedicine in the OECD: An umbrella review of clinical and cost-effectiveness, patient experience and implementation	Review	98	2014 - 2019	multiple countries	Multiple countries	Health System Managers	Telehealth
Ezezika 2021	The implementation of a maternal mHealth project in South Africa: Lessons for taking mHealth innovations to scale	Qualitative	interviews: 17	11/2019 - 02/2020	South Africa	Sub Saharan Africa	Users of Health Services ; Health System Managers	DiHA
Fazakarley 2023	Experiences of using artificial intelligence in healthcare: A qualitative study of UK clinician and key stakeholder perspectives	Qualitative	13	12/2021 - 09/2022	United Kingdom	Europe and Central Asia	Health System Managers	Artificial intelligence
FernándezCoves 2022	Teleconsultation adoption since COVID-19: Comparison of barriers and facilitators in primary care settings in Hong Kong and the Netherlands	Mixed-methods	45 papers, 13 participants	2011 - 2021	Netherlands, Hongkong	East Asia and Pacific	Healthcare professionals; Health System Managers	Telehealth
Fortuna 2020	Certified Peer Specialists’ Perspective of the Barriers and Facilitators to Mobile Health Engagement	Quantitative	267	02-2018 - 04/2018	United States	North America	Users of Health Services	DiHA
Frank 2021	Drivers and social	Quantitative	1,068	04/2020 - 05/2020	multiple	Multiple	Users of Health	Artificial

PUBLICATION	TITLE	DOCUMENT TYPE	TOTAL NUMBER OF PARTICIPANTS/ INCLUDED STUDIES	SEARCH PERIOD/ STUDY PERIOD	COUNTRY	REGION	STUDY POPULATION	TECHNOLOGY
	implications of Artificial Intelligence adoption in healthcare during the COVID-19 pandemic				countries	countries	Services	intelligence
Frey 2022	Acceptance of digital health applications in non-pharmacological therapies in German statutory healthcare system: Results of an online survey	Quantitative	150	03/2021 - 06/2021	Germany	Europe and Central Asia	**Healthcare Professionals**	DiHA
Frishammar 2023	Digital health platforms for the elderly? Key adoption and usage barriers and ways to address them	Mixed-methods	22	2019 - 2020	Sweden	Europe and Central Asia	**Users of Health Services**	General Digital Health Technology
Furlepa 2022	Recommendations for the Development of Telemedicine in Poland Based on the Analysis of Barriers and Selected Telemedicine Solutions	Review	59	2011 - 2021	multiple countries	Multiple countries	Health System Managers	Telehealth
Gajarawala 2020	Telehealth Benefits and Barriers	Qualitative	not reported	not reported	N/A	N/A	Health System Managers	Telehealth
Ganapathi 2023	Exploring the experiences and views of doctors working with Artificial Intelligence in English healthcare; a qualitative study	Qualitative	11	03/2020 - 04/2020	United Kingdom	Europe and Central Asia	Healthcare Professionals	Artificial intelligence
Giebel 2023	Problems and Barriers Related to the Use of Digital Health Applications: Scoping Review	Review	1,479	2015 - 2021	N/A	N/A	Health System Managers	DiHA
Giesbrecht 2023	Telerehabilitation Delivery in Canada and the Netherlands: Results	Quantitative	723	11/2021 - 03/2022	multiple countries	Multiple countries	Healthcare Professionals	Telehealth

PUBLICATION	TITLE	DOCUMENT TYPE	TOTAL NUMBER OF PARTICIPANTS/ INCLUDED STUDIES	SEARCH PERIOD/ STUDY PERIOD	COUNTRY	REGION	STUDY POPULATION	TECHNOLOGY
	of a Survey Study							
Gray 2022	Artificial Intelligence Education for the Health Workforce: Expert Survey of Approaches and Needs	Review	39	10/2020 - 12/2020	Australia	East Asia and Pacific	Healthcare Professionals; Health System Managers	Artificial intelligence
HallDykgraaf 2021	“A decade's worth of work in a matter of days”: The journey to telehealth for the whole population in Australia	Review	not reported	not reported	Australia	East Asia and Pacific	Health System Managers	Telehealth
Haneef 2020	Innovative use of data sources: A cross-sectional study of data linkage and artificial intelligence practices across European countries	Qualitative	141 (literature search), 29 (countries)	04/2019 - 05/2019	multiple countries	Multiple countries	Health System Managers	Artificial intelligence
Harst 2020	Identifying barriers in telemedicine-supported integrated care research: scoping reviews and qualitative content analysis	Review	118	2007 - now	N/A	N/A	Health System Managers	Telehealth
Hawley 2023	Connecting the disconnected: Leveraging an in-home team member for video visits for older adults.	Review	20	08/2020 - 07/2021	United States	North America	Users of Health Services	Telehealth
Heeres 2023	Drivers and Barriers to Implementing the Internet of Things in the Health Care Supply Chain: Mixed Methods Multicase Study.	Mixed-methods	38	04/2022 - 01/2023	Netherlands	Europe and Central Asia	Health System Managers	Health Data Infrastructure
Hobeck 2021	Overcoming diffusion barriers of digital health innovations: Conception of an assessment method	Review	21	up to 2018	Germany	Europe and Central Asia	Health System Managers; Health Data Service Providers	DiHA

PUBLICATION	TITLE	DOCUMENT TYPE	TOTAL NUMBER OF PARTICIPANTS/ INCLUDED STUDIES	SEARCH PERIOD/ STUDY PERIOD	COUNTRY	REGION	STUDY POPULATION	TECHNOLOGY
Horwood 2023	Challenges of using e-health technologies to support clinical care in rural Africa: a longitudinal mixed methods study exploring primary health care nurses' experiences of using an electronic clinical decision support system (CDSS) in South Africa.	Qualitative	9	10/2020 - 05/2021	South Africa	Sub Saharan Africa	Healthcare Professionals	Health Data Infrastructure
Hosseini 2024	Challenges and solutions for implementing telemedicine in Iran from health policymakers' perspective	Qualitative	19	04/2022 - 07/2022	Iran	Middle East and North Africa	Health System Managers	Telehealth
Hulter 2020	Adopting patient portals in hospitals: Qualitative study	Qualitative	122	2018	multiple countries	Multiple countries	Users of Health Services; Health System Managers	Health Data Infrastructure
Ilali 2023	Telemedicine in the primary care of older adults: A systematic mixed studies review	Review	20	not reported	N/A	N/A	Users of Health Services	Telehealth
Jacob 2020	Social, organizational, and technological factors impacting clinicians' adoption of mobile health tools: Systematic literature review	Review	171	2008 - 2018	N/A	N/A	Healthcare Professionals	DiHA
James 2021	Spread, Scale-up, and Sustainability of Video Consulting in Health Care: Systematic Review and Synthesis Guided by the NASSS Framework	Review	13	2010 onwards	N/A	N/A	Health System Managers	Telehealth
Javaid 2022	Internet of Things in the global healthcare sector:	Qualitative	not relevant	not reported	multiple countries	Multiple countries	Health System Managers	Artificial intelligence

PUBLICATION	TITLE	DOCUMENT TYPE	TOTAL NUMBER OF PARTICIPANTS/ INCLUDED STUDIES	SEARCH PERIOD/ STUDY PERIOD	COUNTRY	REGION	STUDY POPULATION	TECHNOLOGY
	Significance, applications, and barriers							
Johnston 2022	Foundation Level Barriers to the Widespread Adoption of Digital Solutions by Care Homes: Insights from Three Scottish Studies.	Qualitative	not relevant	not reported	United Kingdom, Scotland	Europe and Central Asia	Healthcare Professionals	General Digital Health Technology
Jonasdottir 2022	Health professionals? Perspective towards challenges and opportunities of telehealth service provision: A scoping review	Review	22	2010 - 2020	N/A	N/A	Healthcare Professionals	Telehealth
Jonnagaddala 2021	From telehealth to virtual primary care in Australia? A Rapid scoping review	Review	29	01/20 - 12/20	Australia	East Asia and Pacific	Health System Managers	Telehealth
Kaihlanen 2022	Towards digital health equity - a qualitative study of the challenges experienced by vulnerable groups in using digital health services in the COVID-19 era	Qualitative	74	10/2020 - 05/2021	Finland	Europe and Central Asia	Users of Health Services	General Digital Health Technology
Kalicki 2021	Barriers to telehealth access among homebound older adults	Quantitative	16	2020/04 - 2020/06	United States	North America	Users of Health Services	Telehealth
Kester 2022	Telepharmacy services in acute care: Diverse needs within a large health system.	Qualitative	not reported	not reported	United States	North America	Healthcare Professionals	Telehealth
Khalifa 2021	Utilizing Health Analytics in Improving the Performance of Hospitals and Healthcare Services: Promises and	Review	60	2010 to 2020	N/A	N/A	Health System Managers	Artificial intelligence

PUBLICATION	TITLE	DOCUMENT TYPE	TOTAL NUMBER OF PARTICIPANTS/ INCLUDED STUDIES	SEARCH PERIOD/ STUDY PERIOD	COUNTRY	REGION	STUDY POPULATION	TECHNOLOGY
	Challenges							
Khodadad-Saryazdi 2021	Exploring the telemedicine implementation challenges through the process innovation approach: A case study research in the French healthcare sector	Qualitative	2	not reported	France	Europe and Central Asia	Healthcare Professionals; Health System Managers	Telehealth
Kilova 2022	Electronic Health in the practice of general practitioners in Bulgaria	Qualitative	381	2020/12 - 2021/01	Bulgaria	Europe and Central Asia	Healthcare Professionals	Health Data Infrastructure
Klaver 2021	Relationship between perceived risks of using mhealth applications and the intention to use them among older adults in the Netherlands: Cross-sectional study	Quantitative	481	02/2020 - 06/2020	Netherlands	Europe and Central Asia	Users of Health Services	DiHA
Kosari 2020	Pharmacists' Perspectives on the Use of My Health Record.	Quantitative	63	09/2018 - 03/2019	Australia	East Asia and Pacific	Healthcare Professionals	Health Data Infrastructure
Kruse 2020	Utilization barriers and medical outcomes commensurate with the use of telehealth among older adults: Systematic review	Review	57	02/2016 - 02/2020	N/A	N/A	Users of Health Services	Telehealth
Kruse 2021	Telemedicine and health policy: A systematic review	Review	48	01/2015 - 06/2020	N/A	N/A	Health System Managers	Telehealth
Kruse 2023	Analysing the Effect of Telemedicine on Domains of Quality through Facilitators and Barriers to Adoption: Systematic Review	Review	33	up to date 2022	N/A	N/A	Health System Managers	Telehealth

PUBLICATION	TITLE	DOCUMENT TYPE	TOTAL NUMBER OF PARTICIPANTS/ INCLUDED STUDIES	SEARCH PERIOD/ STUDY PERIOD	COUNTRY	REGION	STUDY POPULATION	TECHNOLOGY
Kushniruk 2021	The Human Factors of AI in Healthcare: Recurrent Issues, Future Challenges and Ways Forward	Qualitative	not relevant	not reported	N/A	N/A	Health System Managers	Artificial intelligence
Lavallee 2020	mHealth and patient generated health data: stakeholder perspectives on opportunities and barriers for transforming healthcare	Qualitative	41	not reported	United States	North America	Users of Health Services; Healthcare Professionals; Health System Managers	DiHA
Leonard 2020	Investigating the barriers and facilitators to implementing an eHealth innovation into a resource-constrained setting: A South African case study	Review	not reported	not reported	South Africa	Sub Saharan Africa	Health System Managers	General Digital Health Technology
Lew 2023	Perceptions and Attitudes of Patients and Health Care Stakeholders on Implementing a Telehealth Service for Preoperative Evaluation: A Qualitative Analysis	Qualitative	25	07/2021 - 11/2021	Singapore	East Asia and Pacific	Users of Health Services;Healthcare Professionals	Telehealth
Li 2020	Patients' Perceptions of Barriers and Facilitators to the Adoption of E-Hospitals: Cross-Sectional Study in Western China.	Qualitative	1,032	06/2019 - 08/2019	China	East Asia and Pacific	Users of Health Services	General Digital Health Technology
Li 2022	Readiness of healthcare providers for e-hospitals: a cross-sectional analysis in China before the COVID-19 period	Review	2,298	06/2019 - 09/2019	China	East Asia and Pacific	Healthcare Professionals	General Digital Health Technology
Li 2023	Technical/Algorithm, Stakeholder, and Society (TASS) barriers to the	Review	306	01/2001 - 01/2023	N/A	N/A	Health System Managers	Artificial intelligence

PUBLICATION	TITLE	DOCUMENT TYPE	TOTAL NUMBER OF PARTICIPANTS/ INCLUDED STUDIES	SEARCH PERIOD/ STUDY PERIOD	COUNTRY	REGION	STUDY POPULATION	TECHNOLOGY
	application of artificial intelligence in medicine: A systematic review							
Liljeroos 2023	Implementation of Telemonitoring in Health Care: Facilitators and Barriers for Using eHealth for Older Adults with Chronic Conditions	Qualitative	14	not reported	Sweden	Europe and Central Asia	Healthcare Professionals	Telehealth
Lin 2020	Seniors and mobiles: A qualitative inquiry of mHealth adoption among Singapore seniors	Qualitative	35	03/2014 - 04/2014	Singapore	East Asia and Pacific	Users of Health Services	DiHA
Lindenfeld 2023	Synchronous Home-Based Telemedicine for Primary Care: A Review	Review	22	2010 - 2021	N/A	N/A	Health System Managers	Telehealth
Lingg 2020	Health system stakeholders' perspective on the role of mobile health and its adoption in the Swiss health system: Qualitative study	Qualitative	50	07/2019 - 10/2019	Switzerland	Europe and Central Asia	Healthcare Professionals	DiHA
Lokshina 2019	Evaluation of IoT-driven eHealth: Knowledge management, business models and opportunities, deployment and evolution	Review	not relevant	not relevant	N/A	N/A	Health System Managers	Artificial intelligence
Lori 2020	Experiences of Medicaid Programs and Health Centers in Implementing Telehealth.	Qualitative	26	06/2018 - 08/2018	United States	North America	Healthcare Professionals;Health System Managers	Telehealth
Lundereng 2023	Health Care Professionals' Experiences and Perspectives on Using Telehealth for Home-based Palliative Care: Scoping Review	Review	29	01/2000 - 08/2022	N/A	N/A	Healthcare Professionals	Telehealth

PUBLICATION	TITLE	DOCUMENT TYPE	TOTAL NUMBER OF PARTICIPANTS/ INCLUDED STUDIES	SEARCH PERIOD/ STUDY PERIOD	COUNTRY	REGION	STUDY POPULATION	TECHNOLOGY
Ma 2022	Usage and perceptions of telemedicine among health care professionals in China	Qualitative	1,349	08/2019 - 10/2019	China	East Asia and Pacific	Healthcare Professionals	Telehealth
Macias 2022	Utilizing big data from electronic health records in paediatric clinical care	Qualitative	not reported	not reported	N/A	N/A	Users of Health Services	Health Data Infrastructure
Madanian 2023	Patients' perspectives on digital health tools	Review	71	01/2010 - 11/2021	N/A	N/A	Users of Health Services	General Digital Health Technology
Mahapatra 2023	Translational Challenges of Implementing AI in Healthcare: Solutions and Opportunities	Qualitative	not reported	not reported	multiple countries	Multiple countries	Users of Health Services	Artificial intelligence
Malavasi 2024	Validation of an IoT-based home system for integrated care services: a qualitative investigation involving older adults with multiple chronic health conditions	Review	15	not reported	Italy	Europe and Central Asia	Users of Health Services	Artificial intelligence
Marco-Ruiz 2024	A multinational study on artificial intelligence adoption: Clinical implementers' perspectives	Qualitative	37	not reported	multiple countries	Multiple countries	Healthcare Professionals	Artificial intelligence
Mathur 2023	Barriers and Solutions to Adoption of AI in Healthcare	Qualitative	not reported	not reported	multiple countries	Multiple countries	Healthcare Professionals	Artificial intelligence
May-21	Challenges in current nursing home care in rural Germany and how they can be reduced by telehealth - an exploratory qualitative pre-post study	Review	13	09/2020 - 12/2020	Germany	Europe and Central Asia	Healthcare Professionals	Telehealth
Mbunge 2021	Sensors and healthcare 5.0: transformative shift	Qualitative	not relevant	not reported	N/A	N/A	Health System Managers	Artificial intelligence

PUBLICATION	TITLE	DOCUMENT TYPE	TOTAL NUMBER OF PARTICIPANTS/ INCLUDED STUDIES	SEARCH PERIOD/ STUDY PERIOD	COUNTRY	REGION	STUDY POPULATION	TECHNOLOGY
	in virtual care through emerging digital health technologies							
Moll 2023	“It depends on the people!” – A qualitative analysis of contextual factors, prior to the implementation of digital health innovations for chronic condition management, in a German integrated care network	Review	18	11/2020 - 02/2021	Germany	Europe and Central Asia	Healthcare Professionals	General Digital Health Technology
Mosch 2022	Creation of an Evidence-Based Implementation Framework for Digital Health Technology in the Intensive Care Unit: Qualitative Study	Qualitative	7	05/2018 - 03/2020	Germany	Europe and Central Asia	Healthcare Professionals	General Digital Health Technology
Mouloudj 2023	Adopting artificial intelligence in healthcare: A narrative review	Review	50	2018 - 2023	N/A	N/A	Health System Managers	Artificial intelligence
Muller 2023	Needs, expectations, facilitators, and barriers among insurance physicians related to the use of eHealth in their work: results of a survey	Quantitative	315	07/2020	Netherlands	Europe and Central Asia	Healthcare Professionals	General Digital Health Technology
Nataliansyah 2022	Managing innovation: a qualitative study on the implementation of telehealth services in rural emergency departments	Review	18	2016 - 2018	United States	North America	Health Data Service Providers	Telehealth
Natsiavas 2019	Citizen perspectives on cross-border eHealth data exchange: A European survey	Quantitative	437	not reported	multiple countries	Multiple countries	Users of Health Services ; Health System Managers; Health Data Service	Health Data Infrastructure

PUBLICATION	TITLE	DOCUMENT TYPE	TOTAL NUMBER OF PARTICIPANTS/ INCLUDED STUDIES	SEARCH PERIOD/ STUDY PERIOD	COUNTRY	REGION	STUDY POPULATION	TECHNOLOGY
							Providers	
NavarroMartínez 2023	Nurses' view of benefits, enablers and constraints to the use of digital health tools with patients: A cross-sectional study	Quantitative	848	12/2020 - 12/2020	Spain	Europe and Central Asia	Healthcare Professionals	General Digital Health Technology
Neher 2022	Perspectives of Policy Makers and Service Users concerning the Implementation of eHealth in Sweden: Interview Study	Qualitative	13	2018	Sweden	Europe and Central Asia	Users of Health Services; Health System Managers	DiHA
Nene 2023	Personalized Telehealth: Redesigning Complex Care Delivery for the 65+ during the COVID Pandemic: a Survey of Patients, Caregivers, and Health-care Providers	Quantitative	39 health-care providers, 40 patients, and 22 care givers	02/2021-03/2021	Canada	North America	Users of Health Services; Healthcare Professionals	Telehealth
Nezamdoust 2022	Adopting mobile health applications by nurses: a scoping review	Review	25	01/2000 - 03/2019	multiple countries	Multiple countries	Healthcare Professionals	DiHA
Ng 2022	Perception and Attitude of Malaysian Community Pharmacists Towards the Implementation of Telepharmacy	Quantitative	217	09/2020 - 11/2020	Malaysia	East Asia and Pacific	Healthcare Professionals	Telehealth
Odendaal 2020	Health workers' perceptions and experiences of using mHealth technologies to deliver primary healthcare services: a qualitative evidence synthesis.	Review	43	12/2015 AND 01/2018	N/A	N/A	Healthcare Professionals	DiHA
Offermann 2023	Telemedicine in nursing homes: Insights on the social acceptance and	Qualitative	14	03/2022 - 04/2022	Germany	Europe and Central	Healthcare Professionals	Telehealth

PUBLICATION	TITLE	DOCUMENT TYPE	TOTAL NUMBER OF PARTICIPANTS/ INCLUDED STUDIES	SEARCH PERIOD/ STUDY PERIOD	COUNTRY	REGION	STUDY POPULATION	TECHNOLOGY
	ethical acceptability of telemedical consultations					Asia		
Olawade 2023	Using artificial intelligence to improve public health: a narrative review	Review	not reported	not reported	N/A	N/A	Health System Managers	Artificial intelligence
Olaye 2023	The Gap Between AI and Bedside: Participatory Workshop on the Barriers to the Integration, Translation, and Adoption of Digital Health Care and AI Startup Technology Into Clinical Practice	Qualitative	10	not reported	United States	North America	Healthcare Professionals; Health Data Service Providers	Artificial intelligence
Olesen 2023	Usefulness of a Digitally Assisted Person-Centered Care Intervention: Qualitative Study of Patients' and Nurses' Experiences in a Long-term Perspective	Qualitative	27	2018 - 2022	Denmark	Europe and Central Asia	Users of Health Services; Healthcare Professionals	DiHA
Osman 2019	Barriers and facilitators for implementation of electronic consultations (eConsult) to enhance access to specialist care: A scoping review	Review	130	09/2017 - 12/2018	multiple countries	Multiple countries	Users of Health Services; Healthcare Professionals	Telehealth
Otto 2019	Investigating barriers for the implementation of telemedicine initiatives: A systematic review of reviews	Review	8	2012 - 2016	N/A	N/A	Users of Health Services; Healthcare Professionals; Health System Managers	Telehealth
Palombini 2023	Building a Framework for a More Inclusive Healthcare System	Qualitative	11	not reported	multiple countries	Multiple countries	Healthcare Professionals; Health System Managers; Health Data Service Providers	Telehealth

PUBLICATION	TITLE	DOCUMENT TYPE	TOTAL NUMBER OF PARTICIPANTS/ INCLUDED STUDIES	SEARCH PERIOD/ STUDY PERIOD	COUNTRY	REGION	STUDY POPULATION	TECHNOLOGY
Pan 2021	Perception and initial adoption of mobile health services of older adults in London: Mixed methods investigation	Mixed-methods	42 (Study 1= 30, Study 2= 12)	Study 1= 01/2017 - 02/2017, Study 2= 03/2017	United Kingdom	Europe and Central Asia	Users of Health Services	DiHA
Panda 2021	Perceptions of Mobile Health Technology in Elective Surgery: A Qualitative Study of North American Surgeons	Review	30	not reported	United States; Canada	North America	Healthcare Professionals	other
Paranjape 2021	The Value of Artificial Intelligence in Laboratory Medicine	Quantitative	128	08/2019	Netherlands	Europe and Central Asia	Healthcare Professionals	Artificial intelligence
Patterson 2022	Virtual care and the influence of a pandemic: Necessary policy shifts to drive digital innovation in healthcare	Review	28	not reported	Canada	North America	Users of Health Services; Healthcare Professionals	General Digital Health Technology
Payán 2022	Telemedicine implementation and use in community health centers during COVID-19: Clinic personnel and patient perspectives	Qualitative	24	10/2020 - 04/2021	United States	North America	Users of Health Services; Healthcare Professionals; Health System Managers	Telehealth
Pechtor 2023	Unravelling the processes and challenges of Artificial Intelligence Implementation in the Swiss Public Sector : A Toe Framework Analysis	Review	6	01/2023 - 04/2023	Switzerland	Europe and Central Asia	Health System Managers; Health Data Service Providers	Artificial intelligence
Pohlmann 2020	Digitalizing health services by implementing a personal electronic health record in Germany: Qualitative analysis of fundamental	Review	33	HEALTHCARE PROVIDER = 09/2015 - 03/2016, HEALTH SYSTEM	Germany	Europe and Central Asia	Healthcare Professionals; Health System Managers	Health Data Infrastructure

PUBLICATION	TITLE	DOCUMENT TYPE	TOTAL NUMBER OF PARTICIPANTS/ INCLUDED STUDIES	SEARCH PERIOD/ STUDY PERIOD	COUNTRY	REGION	STUDY POPULATION	TECHNOLOGY
	prerequisites from the perspective of selected experts			MANAGERS = 06/2016 - 10/2016				
Pomales-Ramos 2023	A mixed-methods examination of clinicians' perceived barriers to telehealth delivered applied behavior analysis	Mixed-methods	388	07/2020 - 09/2020	United States	North America	Healthcare Professionals	Telehealth
Poon 2022	A qualitative research study of primary care physicians' views of telehealth in delivering postnatal care to women	Review	29	02/2020 - 10/2020	Singapore	East Asia and Pacific	Healthcare Professionals	Telehealth
Prendergast 2019	The barriers and facilitators for nurse educators using telehealth for education	Mixed-methods	19	2016	New Zealand	East Asia and Pacific	Healthcare Professionals; Health System Managers	Telehealth
Rabanifar 2022	Exploring Barriers to Implementing Telerehabilitation from experiences of managers, policymakers, and providers of rehabilitation services in Iran: A Qualitative Study	Review	26	02/2021 - 11/2021	Iran	Middle East and North Africa	Healthcare Professionals; Health System Managers	Telehealth
Rabinowitz 2023	The telemedicine experience in primary care practices in the united states: Insights from practice leaders	Qualitative	25	04/2021 - 09/2021	United States	North America	Healthcare Professionals; Health System Managers	Telehealth
Raghavan 2021	Public health innovation through cloud adoption: A comparative analysis of drivers and barriers in Japan, South Korea, and Singapore	Qualitative	not relevant	not reported	multiple countries	Multiple countries	Healthcare Professionals	Health Data Infrastructure
Ramachandran 2023	Identifying Challenges and Barriers in Wearable	Review	58	not reported	N/A	N/A	Users of Health Services	other

PUBLICATION	TITLE	DOCUMENT TYPE	TOTAL NUMBER OF PARTICIPANTS/ INCLUDED STUDIES	SEARCH PERIOD/ STUDY PERIOD	COUNTRY	REGION	STUDY POPULATION	TECHNOLOGY
	Medical Devices Adoption through Text Mining							
Ramdani 2020	Exploring the determinants of mobile health adoption by hospitals in China: Empirical study	Quantitative	87	not reported	China	East Asia and Pacific	Healthcare Professionals	DiHA
Ramos 2020	Ehealth in Spain: Evolution, current status and future prospects	Quantitative	1.695	05/ 2018 - 06/2018	Spain	Europe and Central Asia	Users of Health Services	Health Data Infrastructure
Rauwerdink 2021	Successes of and lessons from the first joint ehealth program of the Dutch university hospitals: Evaluation study	Mixed-methods	not reported	not reported	Netherlands	Europe and Central Asia	Health System Managers	Health Data Infrastructure
Reinhardt 2021	Non-use of telemedicine: A scoping review	Review	73	2004 - 2020	multiple countries	Multiple countries	Users of Health Services	Telehealth
Rodrigues 2024	Barriers and facilitators of health professionals in adopting digital health-related tools for medication appropriateness: A systematic review	Review	15	01/2000 - 10/2022	multiple countries	Multiple countries	Healthcare Professionals	DiHA
Rohowsky 2023	Everybody hurts sometimes: perceptions of benefits and barriers in telemedical consultations	Mixed-methods	217	not reported	Germany	Europe and Central Asia	Users of Health Services; Healthcare Professionals	Telehealth
Roppelt 2023	Artificial intelligence in healthcare institutions: A systematic literature review on influencing factors	Review	130	08/2022 - 10/2022	N/A	N/A	Healthcare Professionals	Artificial intelligence
Saxena 2022	Advancing digital technologies in healthcare	Qualitative	not relevant	not reported	N/A	N/A	Users of Health Services	General Digital Health

PUBLICATION	TITLE	DOCUMENT TYPE	TOTAL NUMBER OF PARTICIPANTS/ INCLUDED STUDIES	SEARCH PERIOD/ STUDY PERIOD	COUNTRY	REGION	STUDY POPULATION	TECHNOLOGY
								Technology
Scheibner 2021	Benefits, challenges, and contributors to success for national eHealth systems implementation: A scoping review	Review	86	01/2000 - 2020	multiple countries	Multiple countries	Users of Health Services; Healthcare Professionals; Health System Managers	Health Data Infrastructure
Schouten 2022	Implementing artificial intelligence in clinical practice: a mixed-method study of barriers and facilitators	Mixed-methods	121	08/2021 - 02/2022	Netherlands	Europe and Central Asia	Healthcare Professionals	Artificial intelligence
Schreiweis 2019	Barriers and facilitators to the implementation of eHealth services: Systematic literature analysis	Mixed-methods	expert discussion: 23, literature analysis: 38	literature analysis: 05/2018	multiple countries	Multiple countries	Health System Managers	General Digital Health Technology
Schroeder 2023	Enablers and inhibitors to the adoption of mHealth apps by patients – A qualitative analysis of German doctors' perspectives	Qualitative	28	not reported	Germany	Europe and Central Asia	Healthcare Professionals	DiHA
Schroeder 2024	What would it take to improve the uptake and utilisation of mHealth applications among older Australians? A qualitative study	Qualitative	21	07/2022 - 09/2022	Australia	East Asia and Pacific	Users of Health Services	DiHA
Serrano 2018	Analysis of Barriers to the Deployment of Health Information Systems: a Stakeholder Perspective	Mixed-methods	Review: 76, 33	not reported	Spain	Europe and Central Asia	Users of Health Services; Healthcare Professionals; Health System Managers; Health Data Service Providers	Health Data Infrastructure
Seto 2019	Opportunities and challenges of telehealth in remote communities: Case study of the Yukon	Mixed-methods	36	04/2016 - 08/2016	Canada	North America	Users of Health Services; Healthcare Professionals; Health Data Service	Telehealth

PUBLICATION	TITLE	DOCUMENT TYPE	TOTAL NUMBER OF PARTICIPANTS/ INCLUDED STUDIES	SEARCH PERIOD/ STUDY PERIOD	COUNTRY	REGION	STUDY POPULATION	TECHNOLOGY
	telehealth system						Providers	
Shabir 2022	The Barriers and Facilitators to the Use of Lifestyle Apps: A Systematic Review of Qualitative Studies.	Review	25	2016 - 2020	N/A	N/A	Users of Health Services	DiHA
Shah 2021	Governing health data across changing contexts: A focus group study of citizen's views in England, Iceland, and Sweden	Review	71	not reported	multiple countries	Multiple countries	Users of Health Services	Health Data Infrastructure
Sharma 2023	Addressing the challenges of AI-based telemedicine: Best practices and lessons learned	Review	not reported	not reported	multiple countries	Multiple countries	Health System Managers	Telehealth
Shear 2023	Experts' Perspectives on Use of Fast Healthcare Interoperable Resources for Computerized Clinical Decision Support	Qualitative	5	10/2021 - 11/2021	United States	North America	Health Data Service Providers	Health Data Infrastructure
Shinners 2023	Healthcare professionals' experiences and perceptions of artificial intelligence in regional and rural health districts in Australia	Quantitative	173	03/2021 - 07/2021	Australia	East Asia and Pacific	Healthcare Professionals	Artificial intelligence
Shull 2019	Digital health and the state of interoperable electronic health records	Qualitative	not relevant	not reported	Spain	Europe and Central Asia	NA	Health Data Infrastructure
Singh 2020	Current challenges and barriers to real-world artificial intelligence adoption for the healthcare system, provider, and the patient	Review	not relevant	not reported	United States	North America	Users of Health Services; Healthcare Professionals; Health System Managers	Artificial intelligence

PUBLICATION	TITLE	DOCUMENT TYPE	TOTAL NUMBER OF PARTICIPANTS/ INCLUDED STUDIES	SEARCH PERIOD/ STUDY PERIOD	COUNTRY	REGION	STUDY POPULATION	TECHNOLOGY
Singh 2021	Exploring the perspectives of primary care providers on use of the electronic Patient Reported Outcomes tool to support goal-oriented care: a qualitative study	Qualitative	13	not reported	Canada	North America	Healthcare Professionals	DiHA
Singh 2023	Technological paradoxes and artificial intelligence implementation in healthcare. An application of paradox theory	Review	62	not reported	N/A	N/A	Health System Managers	Artificial intelligence
SinhaGregory 2023	The feasibility, acceptability, and usability of telehealth visits	Qualitative	79 hcp, 240 patients	10/2020 - 04/2021	United States	North America	Users of Health Services; Healthcare Professionals	Telehealth
Smirnova 2021	Adoption and use of health-related mobile applications: A qualitative study with experienced users	Qualitative	10	not reported	N/A	N/A	Users of Health Services	DiHA
SolbergCarlsson 2023	Rapid implementation of remote digital primary care in Stockholm and implications for further system-wide implementation: practitioner's and manager's experience of the Always Open mobile application.	Qualitative	99	03/2020-11/2021	Sweden	Europe and Central Asia	Healthcare Professionals	DiHA
Sonawane 2023	The application of artificial intelligence: perceptions from healthcare professionals	Qualitative	21	not reported	United States	North America	Healthcare Professionals ; Health System Managers	Artificial intelligence
Sony 2023	Critical Success Factors for Successful	Review	89	not reported	multiple countries	Multiple countries	Health System Managers	General Digital Health

PUBLICATION	TITLE	DOCUMENT TYPE	TOTAL NUMBER OF PARTICIPANTS/ INCLUDED STUDIES	SEARCH PERIOD/ STUDY PERIOD	COUNTRY	REGION	STUDY POPULATION	TECHNOLOGY
	Implementation of Healthcare 4.0: A Literature Review and Future Research Agenda							Technology
Sousa 2019	Digital and innovation policies in the health sector	Mixed-methods	103	2015	multiple countries	Multiple countries	Health System Managers	General Digital Health Technology
Stasevych 2023	Innovative Robotic Technologies and Artificial Intelligence in Pharmacy and Medicine: Paving the Way for the Future of Health Care—A Review	Review	not relevant	not reported	N/A	N/A	Health System Managers	Artificial intelligence
Steinhauser 2020	The Relative Role of Digital Complementary Assets and Regulation in Discontinuous Telemedicine Innovation in European Hospitals	Quantitative	1753 acute care hospitals	10/2012-02/2013	multiple countries	Multiple countries	Healthcare Professionals	Telehealth
Støme 2021	Enabling guidelines for the adoption of eHealth solutions: Scoping review	Review	27	04/2019	multiple countries	Multiple countries	Users of Health Services	General Digital Health Technology
Sumner 2023	Artificial intelligence in physical rehabilitation: A systematic review	Review	29	07/2020 - 10/2021	multiple countries	Multiple countries	Users of Health Services; Healthcare Professionals	Artificial intelligence
Sundstrom 2019	"People are struggling in this area:" a qualitative study of women's perspectives of telehealth in rural South Carolina	Review	52	06/2015-08/2015	United States	North America	Users of Health Services; Healthcare Professionals	Telehealth
Tabaeeian 2022	A systematic review of telemedicine systems use barriers: primary health care providers' perspective	Review	37	2009 - 2019	multiple countries	Multiple countries	Healthcare Professionals	Telehealth
Taboada 2021	Implementing Goal Mama: Barriers and	Mixed-methods	42	not reported (6-month study	United States	North America	Healthcare Professionals; Health	DiHA

PUBLICATION	TITLE	DOCUMENT TYPE	TOTAL NUMBER OF PARTICIPANTS/ INCLUDED STUDIES	SEARCH PERIOD/ STUDY PERIOD	COUNTRY	REGION	STUDY POPULATION	TECHNOLOGY
	Facilitators to Introducing Mobile Health Technology in a Public Health Nurse Home-Visiting Program.			period)			System Managers	
Temsah 2023	ChatGPT and the Future of Digital Health: A Study on Healthcare Workers' Perceptions and Expectations	Quantitative	1057	02/2023-06/2023	Saudi Arabia	Middle East and North Africa	Healthcare Professionals; Health System Managers; Health Data Service Providers	Artificial intelligence
Temsah 2023	Healthcare's New Horizon With ChatGPT's Voice and Vision Capabilities: A Leap Beyond Text.	Qualitative	not reported	not reported	Saudi Arabia	Middle East and North Africa	Healthcare Professionals; Health System Managers	Artificial intelligence
Teng 2022	Virtual Care Adoption - Challenges and Opportunities From the Lens of Academic Primary Care Practitioners	Review	not relevant	not reported	United States	North America	Users of Health Services ; Healthcare Professionals	Telehealth
Terry 2022	Is primary health care ready for artificial intelligence? Stakeholder perspectives: Worth the risk as long as you do it well	Qualitative	14	09/2020 - 02/2021	Canada	North America	Health System Managers	Artificial intelligence
Těšinová 2023	Development of telemedicine in the Czech Republic from patients' and other key stakeholders' perspective	Qualitative	32	2021-2022	Czech Republic	Europe and Central Asia	Users of Health Services ; Healthcare Professionals; Health System Managers	Telehealth
ThomassenEEK 2023	Willingness, perceived facilitators and barriers to use remote care among healthcare professionals - a cross-sectional study.	Quantitative	130	04/2022	Norway	Europe and Central Asia	Healthcare Professionals	Telehealth
Tierney 2023	Telemedicine	Review	45	06/2021 - 12/2021	United	North	Users of Health	Telehealth

PUBLICATION	TITLE	DOCUMENT TYPE	TOTAL NUMBER OF PARTICIPANTS/ INCLUDED STUDIES	SEARCH PERIOD/ STUDY PERIOD	COUNTRY	REGION	STUDY POPULATION	TECHNOLOGY
	Implementation for Safety Net Populations: A Systematic Review				States	America	Services	
Tomasella 2021	“Sometimes I don’t have a pulse … and I’m still alive!” Interviews with healthcare professionals to explore their experiences of and views on population-based digital health technologies	Qualitative	9	05/2019-07/2019	United Kingdom	Europe and Central Asia	Users of Health Services ; Healthcare Professionals; Health Data Service Providers	General Digital Health Technology
Tossaint-Schoenmakers 2021	The Challenge of Integrating eHealth Into Health Care: Systematic Literature Review of the Donabedian Model of Structure, Process, and Outcome	Review	11	up to 12/2019	N/A	N/A	Health System Managers	General Digital Health Technology
Totten 2022	Telehealth-guided provider-to-provider communication to improve rural health: A systematic review	Review	166	01/2021 - 10/2021	N/A	N/A	Healthcare Professionals	Telehealth
Traube 2021	Strategies for implementation of virtual home visitation in the United States	Review	15	01/2017-01/2019	United States	North America	Users of Health Services; Healthcare Professionals	Telehealth
Tully 2021	Barriers and Facilitators for Implementing Paediatric Telemedicine: Rapid Review of User Perspectives	Review	27	not reported	multiple countries	Multiple countries	Users of Health Services; Healthcare Professionals	Telehealth
Twamley 2022	Exploring the perceptions of former ICU patients and clinical staff on barriers and facilitators to the implementation of	Review	13	not reported	United Kingdom	Europe and Central Asia	Healthcare Professionals	Other

PUBLICATION	TITLE	DOCUMENT TYPE	TOTAL NUMBER OF PARTICIPANTS/ INCLUDED STUDIES	SEARCH PERIOD/ STUDY PERIOD	COUNTRY	REGION	STUDY POPULATION	TECHNOLOGY
	virtual reality exposure therapy: A qualitative study							
Valeur 2021	Patient rationales against the use of patient-accessible electronic health records: Qualitative study	Qualitative	40	2018 - 2019	Norway	Europe and Central Asia	Users of Health Services	Health Data Infrastructure
vanAcker 2023	Older Adults' User Engagement With Mobile Health: A Systematic Review of Qualitative and Mixed-Methods Studies.	Review	32	2014 - 2021	multiple countries	Multiple countries	Users of Health Services	DiHA
vanVelthoven 2019	Digitization of healthcare organizations: The digital health landscape and information theory	Qualitative	12	08/2017	Switzerland	Europe and Central Asia	Healthcare Professionals; Health System Managers	General Digital Health Technology
vanVelthoven 2021	Sustainable adoption of digital health innovations: Perspectives from a stakeholder workshop	Qualitative	12	08/2017	Switzerland	Europe and Central Asia	Users of Health Services ; Healthcare Professionals ; Health System Managers ; Health Data Service Providers	General Digital Health Technology
Vecchia 2022	Willingness of French General Practitioners to Prescribe mHealth Apps and Devices: Quantitative Study	Quantitative	226	06/2019 - 10/2019	France	Europe and Central Asia	Healthcare Professionals	DiHA
Vo 2023	Multi-stakeholder preferences for the use of artificial intelligence in healthcare: A systematic review and thematic analysis	Review	105	01/2001 - 08/2021	multiple countries	Multiple countries	Users of Health Services ; Healthcare Professionals	Artificial intelligence
Wali 2022	Primary Care Physician's Perception and Satisfaction With	Quantitative	53	2022	Saudi Arabia	Middle East and North	Healthcare professionals; Health System Managers	Telehealth

PUBLICATION	TITLE	DOCUMENT TYPE	TOTAL NUMBER OF PARTICIPANTS/ INCLUDED STUDIES	SEARCH PERIOD/ STUDY PERIOD	COUNTRY	REGION	STUDY POPULATION	TECHNOLOGY
	Telehealth in the National Guard Primary Healthcare Centers in Jeddah, Saudi Arabia in 2022.					Africa		
Wardlow 2022	Development of telehealth principles and guidelines for older adults: A modified Delphi approach	Mixed-methods	not reported	09/2021-01/2022	United States	North America	Users of Health Services	Telehealth
Waschkau 2020	Evaluation of attitudes towards telemedicine as a basis for successful implementation: A cross-sectional survey among postgraduate trainees in family medicine in Germany	Quantitative	388	07/2016-10/2016	Germany	Europe and Central Asia	Healthcare Professionals	Telehealth
Weichelt 2019	A model for assessing necessary conditions for rural health care's mobile health readiness: Qualitative assessment of clinician-perceived barriers	Qualitative	13	02/2016-04/2016	United States	North America	Healthcare Professionals	DiHA
Weik 2024	Understanding inherent influencing factors to digital health adoption in general practices through a mixed-methods analysis.	Mixed-methods	not reported	not reported	Germany	Europe and Central Asia	Healthcare Professionals; Health System Managers	General Digital Health Technology
Weinert 2022	Perspective of Information Technology Decision Makers on Factors Influencing Adoption and Implementation of Artificial Intelligence	Quantitative	40	10/2020- 02/2021	Germany	Europe and Central Asia	Healthcare Professionals	Artificial intelligence

PUBLICATION	TITLE	DOCUMENT TYPE	TOTAL NUMBER OF PARTICIPANTS/ INCLUDED STUDIES	SEARCH PERIOD/ STUDY PERIOD	COUNTRY	REGION	STUDY POPULATION	TECHNOLOGY
	Technologies in 40 German Hospitals: Descriptive Analysis							
Weltin 2021	The role of telemedicine in gynecologic healthcare: A narrative review	Review	33	2015 - 08/2020	N/A	N/A	Healthcare Professionals	Telehealth
Wilson 2021	Barriers and facilitators to the use of e-health by older adults: a scoping review.	Review	14	08/2020	multiple countries	Multiple countries	Users of Health Services	General Digital Health Technology
Wolff 2021	Success Factors of Artificial Intelligence Implementation in Healthcare	Review	50	2015 - 2020	N/A	N/A	Health System Managers	Artificial intelligence
Wubineh 2023	Exploring the opportunities and challenges of implementing artificial intelligence in healthcare: A systematic literature review	Review	33	2015 - 2022	N/A	N/A	Health System Managers	Artificial intelligence
Yang 2022	Artificial intelligence healthcare service resources adoption by medical institutions based on TOE framework	Qualitative	not relevant	not reported	China	East Asia and Pacific	Healthcare Professionals	Artificial intelligence
Ye 2019	How resource scarcity and accessibility affect patients' usage of mobile health in China: Resource competition perspective	Qualitative	229,516	06/2016-05/2017	China	East Asia and Pacific	Users of Health Services; Healthcare professionals	DiHA
Ye 2023	Implications for implementation and adoption of telehealth in developing countries: a systematic review of China’s practices and	Review	32	until the end of 06/2022	China	East Asia and Pacific	Users of Health Services; Healthcare Professionals; Health System Managers	Telehealth

PUBLICATION	TITLE	DOCUMENT TYPE	TOTAL NUMBER OF PARTICIPANTS/ INCLUDED STUDIES	SEARCH PERIOD/ STUDY PERIOD	COUNTRY	REGION	STUDY POPULATION	TECHNOLOGY
	experiences							
Young 2020	A mobile app to capture EPA assessment data: Utilizing the consolidated framework for implementation research to identify enablers and barriers to engagement.	Qualitative	8 faculty and 10 residents	02/2019 - 03/2019	N/A	N/A	Users of Health Services	Health Data Infrastructure
Yuen 2023	The determinants of users' intention to adopt telehealth: Health belief, perceived value and self-determination perspectives	Qualitative	500	04/2021 - 04/2021	Singapore	East Asia and Pacific	Users of Health Services	Telehealth
Zachrison 2019	Understanding Barriers to Telemedicine Implementation in Rural Emergency Departments	Quantitative	not reported	2016 (3-month period)	United States	North America	Healthcare Professionals;Health System Managers	Telehealth
Zakerabasali 2021	Mobile health technology and healthcare providers: Systemic barriers to adoption	Review	18	01/2015 - 12/2019	multiple countries	Multiple countries	Healthcare Professionals	DiHA
Zarei 2023	Application of artificial intelligence in medical education: A review of benefits, challenges, and solutions	Review	not reported	2023	N/A	N/A	Health System Managers	Artificial intelligence
Zhao 2023	Does the transcultural problem really matter? An integrated approach to analyze barriers to eHealth SMEs' development	Qualitative	20	01/2022 - 04/2022	United Kingdom	Europe and Central Asia	Health Data Service Providers	General Digital Health Technology
Zharima 2023	Exploring the barriers and facilitators to implementing electronic health records in a middle-income country: a	Qualitative	26	11/2021-06/2022	South Africa	Sub Saharan Africa	Healthcare professionals;Health System Managers	Health Data Infrastructure

PUBLICATION	TITLE	DOCUMENT TYPE	TOTAL NUMBER OF PARTICIPANTS/ INCLUDED STUDIES	SEARCH PERIOD/ STUDY PERIOD	COUNTRY	REGION	STUDY POPULATION	TECHNOLOGY
	qualitative study from South Africa							
Zhou 2019	Barriers to and facilitators of the use of mobile health apps from a security perspective: Mixed-methods study	Mixed-methods	117	not reported	United Kingdom	Europe and Central Asia	Users of Health Services	DiHA

N/A – Not available, DiHA- Digital Health Applications

Additional file Table 5: Overview of Included Publications and Identified Barriers & Facilitators

PUBLICATION	TITLE	BARRIERS	FACILITATORS
AbdRazak 2020	Investigating the Barriers of Health Information System implementation in Malaysian Public Hospitals	- Support<br>- Human<br>- Technology & Infrastructure<br>- Software limitation	- human skills, experience, expertise, satisfaction and quality of information; Human context in Malaysian Public Hospitals had the highest effect of HIS adoption.<br><br>To ensure the successful integration of the system, organizations need the necessary financial and technological resources. However, in developing countries, poor maintenance or lack of personnel in charge of these resources is exceptionally common, and that causes information systems projects to fail significantly.
Abell 2023	Identifying barriers and facilitators to successful implementation of computerized clinical decision support systems in hospitals: a NASSS framework-informed scoping review.	1. Condition (contextual factors and characteristics of the healthcare condition or problem for which a particular intervention or technology is being implemented)<br>2. Technology (material features, knowledge inputs and outputs of the CDSS, its procurement model, and the knowledge and skill required to use it)<br>3. Value proposition (proposition or value intervention offers to users, providers, organizations, and the wider healthcare system<br>4. Adopter system (refers to the individuals, groups, or organizations that are involved in the adoption, implementation, and use of a particular innovation or intervention within the healthcare setting)<br>5. Organisation (considered organizational capacity and readiness to implement CDSS, funding and costs of the new technology, implementation processes, and changes to team interactions and routines)<br>6. Wider system (considers factors such as the political, economic, and social contexts within which the healthcare system operates)	extent of change needed to organizational routines to implement a new CDSS (minimal workflow changes, interruptions, or unnecessary duplication of activities); early, ongoing, and supported implementation plan (local champions and super-users that promoted and supported use of the CDSS; benchmarking, audit, and feedback to drive change; provision of technical training and support; and early engagement and involvement of users in the development and implementation of the CDSS); good overall capacity to innovate (positive institutional culture, clear innovation strategy, organisational support for change, and previous successful technological deployment)
Abels 2019	Computational pathology definitions, best practices, and recommendations for regulatory guidance: a white paper from the Digital Pathology Association.	- Infrastructure: large size of images may present a problem for evaluation, storage, and inventory; processor speed and memory requirements of local workstations, data storage requirements, and limitations of the network management; number of intended users, flexibility of the server or cloud configuration to accommodate new algorithms or case-loads, cyber-security, and associated costs<br><br>- Processor speed and sources: may be necessary to purchase a more powerful GPU	- Infrastructure:<br>Safeguards ensure images are fully loaded and analysis algorithms operate smoothly without disruption from bandwidth, processing power, or memory issues.<br><br>- Processor Speed and Sources:<br>Vendors provide server or cloud-based

PUBLICATION	TITLE	BARRIERS	FACILITATORS
		designed for deep learning; these are generally more expensive and tend to generate more heat<br><br>- Network limitations: The large size of whole slide images presents a potential hurdle for efficient processing in environments that lack sufficient bandwidth<br><br>- Acquiring training data: Obtaining clinical ground truth data suitable for algorithm development is often time-consuming and challenging (It usually takes a long time to generate enough survival data from clinical patients, and clinical data are generally locked into an unstructured format within one or more disparate electronic medical records); Obtaining adequately annotated datasets for deep learning by a trained expert can be difficult due to the amount of time required, associated expenses, and the tedious nature of the task<br><br>- Data variability: in surgical pathology, there is currently no accepted global standard for tissue processing, staining, and slide preparation. Even digital acquisition may introduce variability<br><br>- Public sources: currently only limited publicly available datasets with annotated images and associated non-image patient data that are required for CPATH.<br><br>- Active learning: active learning<br>offers a solution to the problem of limited data annotations in pathology by having the pathologist engage actively with the algorithm, which evolves through continuous learning<br><br>- Quality control and reliability of the algorithm: difficult to establish strict quality control steps for deep learning algorithms, especially in segmentation problems (‘gold-standard paradox’, arises from histopathological assessments by<br>the pathologist being considered the gold standard, but the algorithm data may in fact be more reproducible than human assessment); local regulations apply to legally market any clinical-grade software solution<br><br>- Understanding algorithms: it is very difficult to understand some of the features and neural pathways used to make decisions; Artificial neural networks have accordingly been described as a ‘black box’. This has led to several concerns: difficulty in correcting an underperforming algorithm; lack of transparency, explainability, and provability for humans who may not trust how an algorithm generates reliable results; and regulatory<br>concerns because, unlike traditional image analysis, in<br>deep learning the image features are abstracted in a way that is very difficult for a human to understand	image analysis, offering more resources and broadening access to deep learning capabilities.<br><br>- Acquiring Training Data:<br>Streamlined workflows and a common, intuitive annotation tool ease the creation and sharing of regional annotations. Web-based tools facilitate sharing between research groups, reducing the need for multiple software installations. Annotations from experts, like pathologists, may not always be necessary, balancing quantity with accuracy.<br><br>- Crowdsourcing:<br>Crowdsourced image annotation achieves scientific goals more quickly and cheaply than traditional methods. While it may introduce noise, this can be mitigated with a large training dataset and multiple annotators to reach consensus.<br><br>- Quality Control and Reliability of the Algorithm:<br>Comparing algorithm data to patient outcomes can address the "gold-standard paradox", potentially improving predictive capabilities over manual pathology assessments.<br><br>- Understanding Algorithms:<br>Efforts are underway to make deep learning algorithms more interpretable by clarifying the relationship between inputs and outputs.<br><br>- Ethics:<br>Transparency in the data used for algorithm development promotes interpretability, openness, and trust.

PUBLICATION	TITLE	BARRIERS	FACILITATORS
		- Ethics: Whether to publicly release data that went into creating an algorithm is a complex ethical problem: not exposing the data used by a deep learning model allows companies to create proprietary models that may not be validated or challenged in the public space. On the other hand, exposing private data of a patient can present ethical concerns that violate privacy and as a result may prompt restrictive governance policies and security models. Companies leveraging patient data in order to commercialize artificial intelligence tools and services raises ethical and legal concerns regarding data ownership and intellectual property rights<br>- Cyber security: concerns primarily stem from storing large amounts of medical data in cloud-based systems that can be accessed via the internet.	Robust data governance controls access to various data types and levels.<br><br>- Cyber Security:<br>Decoupling digital images from patient data minimizes data breach risks. Many cloud service providers now offer HIPAA-compliant solutions for enhanced security.
AbHamid 2023	Utilisation of Health Information Technology among Dieticians in the workplace : A qualitative study	Lack of Disease-Specific Tools – Limited apps tailored for conditions like chronic kidney disease.<br><br>Not Suitable for All Age Groups – Elderly patients struggle with digital tools.<br><br>Inaccurate Information – Concerns about misleading or non-evidence-based content.<br><br>Inconvenience & Practicality Issues – Some tools are difficult to use in clinical settings.<br><br>Requires Paid Versions – Limited functionality in free apps deters usage.<br><br>Information Overload – Too much data can overwhelm users.	Self-motivation – Dietitians actively seek out and use health IT for learning and patient management.<br><br>Advertisement – Exposure to health IT tools through social media ads influences adoption.<br><br>Demonstration & Training – Attending courses, events, and webinars increases confidence in using health IT.<br><br>Peer Influence – Colleagues encourage the use of certain applications and tools.<br><br>Patients' Influence – Patients introduce new health IT tools to dietitians.<br><br>Content Quality – Reliable, evidence-based content encourages use.<br><br>Ease of Use – Simple, user-friendly, and practical tools are preferred.<br><br>Developer Credibility – Tools created by government agencies or professional bodies are more trusted.<br><br>Functionality – Multi-device connectivity and comprehensive features improve

PUBLICATION	TITLE	BARRIERS	FACILITATORS
			usability.
Aggarwal 2022	Defining the Enablers and Barriers to the Implementation of Large-scale, Health Care-Related Mobile Technology: Qualitative Case Study in a Tertiary Hospital Setting.	Leadership and Management:<br>-Shifting prioritization<br>-Unclear strategy and road map<br>- Risk aversion<br><br>User Engagement:<br>-Adoption challenge<br>- Not device-agnostic<br>- Functionality and iterative development<br>- COVID-19<br><br>Information Governance: - Difficulties in data extraction<br>- (BYOD) bring-your-own-device policy and (MDM) mobile device management<br>- Regulation<br><br>Partnerships:<br>- Siloed working<br>- Partnership model<br><br>Resourcing and Skills:<br>- Personnel<br>- Investment<br>- Expertise<br>- Training<br>- Support	Leadership and Management:<br>- The involvement of senior leadership in the implementation process helped with decision-making, highlighted the importance<br>of the project within the organization, and helped motivate other key stakeholders in the process<br>- Steering Group<br>- Clinical involvement at leadership level<br>- Motivation and champions for change<br><br>User Engagement:<br>- Communication and engagement with end users<br>- Testing and feedback<br><br>Information Governance:<br>- Data processing and information sharing<br>- Patient engagement and transparency<br><br>Partnerships:<br>- technical, implementation, user engagement, and clinical collaborative working groups<br>- Defined roles and responsibilities between partners<br><br>Resourcing and Skills:<br>- Dedicated project manager
Ahmed 2023	A Systematic Review of the Barriers to the Implementation of Artificial Intelligence in Healthcare.	- ethical barriers (39 articles)<br>- technological barriers (55 articles)<br>- liability and regulatory barriers (37 articles)<br>- workforce barriers (35 articles)<br>- patient safety barriers (24 articles)<br>- and social barriers (18 articles)	
Alanazi 2023	Clinicians' Views on Using Artificial Intelligence in Healthcare: Opportunities, Challenges, and Beyond.	Challenges:<br>Short term:<br>- Data quality concerns<br>- Ethical and privacy concerns<br>- Lack of trust and transparency bias	

PUBLICATION	TITLE	BARRIERS	FACILITATORS
		- Technical limitations<br>- Workforce displacement<br><br>Long term:<br>- Ethical and philosophical concerns<br>- Dependence on AI<br>- Cybersecurity risks<br>- Cost and resource allocation<br>- Unintended consequences of AI<br><br>Threats:<br>Short term:<br>- Lack of efficiency and productivity<br>- Missed diagnoses and treatment opportunities<br>- Inability to provide personalized treatments<br>- Increases in healthcare costs<br>- Limitations of medical research<br><br>Long term<br>- Increased healthcare costs<br>- Delayed access to cutting-edge technology<br>- Missed precision medicine opportunities<br>- Loss of research capabilities<br>- Limited healthcare access and outcomes	
Alanzi 2023	Barriers and Facilitators of Artificial Intelligence in Family Medicine: An Empirical Study With Physicians in Saudi Arabia.	- Performance expectancy (PE)<br>- Effort expectancy (EE)<br>- Social influence (SI)<br>- Facilitating conditions (FC)<br>- Behavioural intention (BI)<br>- Trust<br>- Perceived privacy risk (PPR)<br>- Personalized innovativeness (PI)<br>- Ethical concerns (EC)<br>- Facilitators	- Access to data<br>- Advancements in machine learning<br>- Increased computing power<br>- Digital health technologies<br>- Regulatory support<br>- Interoperability standards<br>- Collaboration and education<br>- Patient engagement<br>- Cost-efficiency<br>- Clinical decision support<br>- Telemedicine<br>- Research and development
AlBadi 2022	Challenges of AI Adoption in the UAE Healthcare	-Accuracy<br>-Privacy & Security<br>-Ethical Barriers<br>-Interpretability<br>-Control	
Al-Dhahir 2023	An overview of facilitators	Development	

PUBLICATION	TITLE	BARRIERS	FACILITATORS
	and barriers in the development of eHealth interventions for people of low socioeconomic position: A Delphi study	-Involving people with a low SEP<br>-Involving professionals<br>-Everyday life of people with a low SEP<br>-Knowledge of professionals<br>-Rewards<br>-Time and financial resources<br><br>Reach<br>-Communication<br>-Engagement<br>-Involving professionals<br>-Everyday life<br>-Finding people with low-SEP<br>-Reach strategies<br>-Knowledge of professionals<br>-Rewards<br>-Social environment<br>-Time and financial resources<br>-Usability<br><br>Adherence<br>-Communication<br>-Engagement<br>-Involving low SEP groups<br>-Everyday life<br>-Modes of delivery<br>-Social environment<br>-Knowledge of people with low-SEP<br>-Motivation of people with low-SEP<br>-Rewards<br>-Usability<br><br>Evaluation<br>-Engagement<br>-Evaluation methods<br>-Everyday life<br>-Planning evaluation<br>-Time and financial resources<br><br>Implementation<br>-Effect of the intervention<br>-Implementation planning	

PUBLICATION	TITLE	BARRIERS	FACILITATORS
		-Time and financial resources<br>-Involving professionals<br>-Privacy	
Aljohani 2019	Adoption of M-Health Applications: The Saudi Arabian Healthcare Perspectives	- Individual Perceptions<br>- Technical Complexities<br>- Social Influences and Trends<br>- Organizational Reliability and Readiness	
Alkureishi 2021	Clinician perspectives on telemedicine: Observational cross-sectional study	The top three most commonly cited barriers were not clinician-specific barriers but rather patient related,<br>including<br>- patient lack of technical knowledge (n=139, 70%)<br>- lack of patient access to necessary technology for a video visit (n=132, 66%)<br>- patient reluctance to have a video visit (n=75, 38%)<br>- inadequate staff support both during (n=70, 35%) and when scheduling visits (n=68, 34%)	
Almathami 2020	Barriers and facilitators that influence telemedicine-based, real-time, online consultation at patients' homes: Systematic literature review	Slow internet speed (n=14)<br>Poor audio quality (n=11)<br>Poor video quality (n=11)<br>Privacy concerns (n=10)<br>High cost (n=10)<br>Security concerns (n=9)<br>Technical issues (n=9)<br>Limited access to technology (n=9)<br>Resistance to change (n=8)<br>Lack of training for patients (n=8)<br>Lack of training for healthcare professionals (n=7)<br>Legal and regulatory barriers (n=7)<br>Concerns about the quality of care (n=6)<br>Cultural and language barriers (n=6)<br>Lack of digital literacy (n=5)<br>Reimbursement issues (n=5)<br>Lack of infrastructure (n=4)<br>Interoperability issues (n=4)<br>Healthcare professionals' acceptance (n=4)<br>Lack of patient acceptance (n=3)<br>Data management issues (n=3)<br>Concerns about technology reliability (n=3)<br>Inadequate resources (n=2)<br>Socioeconomic factors (n=2)<br>Lack of funding (n=1)	Easy to use (n=22)<br>Saving costs (n=21)<br>Patients’ training (n=20)<br>Reducing travel time (n=15)<br>Internet or phone availability (n=15)<br>Reducing waiting time (n=9)<br>Improved accessibility to care (n=9)<br>Motivation and engagement (n=8)<br>Involvement of family (n=7)<br>Better management (n=7)<br>Familiarity with the system (n=6)<br>System approach to improve patients’ compliance (n=5)<br>Security (n=10)<br>Convenience (n=11)<br>Providing support: emotional, technical, and organizational (n=4, 5, and 2)<br>Privacy (n=12)<br>Clinicians’ training and skills (n=13 and 4)<br>Patients’ familiarity with staff and past experience (n=3 and 1)<br>Fast Internet speed (n=3)<br>Enabled body language (n=2)<br>Trust in technology (n=1)

PUBLICATION	TITLE	BARRIERS	FACILITATORS
		Concerns about patient safety (n=1)<br>Issues with technology adoption (n=1)	Patients' ages (n=1)<br>Insurance coverage (n=1)<br>Developed with expert (n=1)
Aloyuni 2020	Knowledge, attitude, and barriers to telerehabilitation-based physical therapy practice in Saudi Arabia	main barriers to implementation of TR in physical therapy settings were<br>- technical issues<br>(24%)<br>- staff skill issues (23%)<br>- high cost (22%) - provider's willingness (20%)<br>- location of the<br>health care institute (10%)	In addition to these limitations, respondents named the attitudes of policymakers, whereas very few participants thought that the lack of skilled personnel and patient compliance factors hinder the use of telerehabilitation services
Alpert 2023	Identifying barriers and facilitators for using a smartwatch to monitor health among older adults	four barriers were found that reduced and discouraged usage of the device:<br>1) little interest in health information, 2) insufficient device functionality, 3) technical difficulties, and 4) insufficient education and training	- Familiarity: the general opinion was that the device was 'easy to use.'<br>- Confidence: using electronics was very high<br>- The Appeal of Tracking Health: The ability of the device to collect health information was perceived as a way to enhance participants' lives.<br>- Doctor's Endorsement: The device compelled participants to think more about their<br>health, and they were inclined to share new knowledge with their physicians.
Alqurashi 2023	The Perception of Health Care Practitioners Regarding Telemedicine During COVID-19 in Saudi Arabia: Mixed Methods Study	- Concerns about patient privacy and confidentiality (agree: 54%, neutral: 27%, disagree: 18%)<br>- High cost of equipment (agree: 36%, neutral: 19%, disagree: 46%)<br>- Negative attitudes of staff involved (agree: 25%, neutral: 24%, disagree: 52%)<br>- Lack of user-friendly software (agree: 33%, neutral: 28%, disagree: 38%)<br>- Lack of suitable training in the use of equipment (agree: 35%, neutral: 32%, disagree: 33%)<br>- perceived increase in workload (agree: 31%, neutral: 44%, disagree: 25%)<br>- Lack of perceived clinical usefulness (agree: 32%, neutral: 40%, disagree: 28%)	National standards are essential for telemedicine technology implementation in Saudi Arabia (agree: 72%, neutral: 17%, disagree: 11%)
Al-rawashdeh 2022	IoT Adoption and Application for Smart Healthcare: A Systematic Review		Main perceived adoption factors of IoT applications in healthcare at the individual level: social influence, attitude, and personal<br>inattentiveness.<br>- Technology level: perceived usefulness, perceived ease of use, performance expectancy, and effort expectations.<br>- Security level: perceived privacy risk<br>- Health level: perceived

PUBLICATION	TITLE	BARRIERS	FACILITATORS
			severity and perceived health risk<br>- Environmental financial cost, and facilitating conditions
Al-Samarraie 2020	Telemedicine in Middle Eastern countries: Progress, barriers, and policy recommendations	- Cultural Barriers:<br><br>Social and Religious Restrictions: These include resistance to being recorded/filmed, and fear of data breaches.<br>Resistance to Change: Both doctors and patients may resist new technologies.<br>Traditional Beliefs: These may hinder acceptance and use of telemedicine.<br>Literacy Levels: Lower literacy can impact understanding and usage.<br>Linguistic Distinctions: Diverse languages make communication challenging.<br><br>- Legal and Regulatory Barriers<br>Lack of Policies and Regulations: Absence of supportive laws for telemedicine.<br>Privacy and Security Concerns: Fears regarding the safety of patient information.<br>Judicial Acceptance: Courts may not accept electronic evidence.<br>Ethical Issues: Concerns about confidentiality and liability.<br><br>- Financial Barriers:<br>High Costs: Implementation and maintenance of telemedicine are costly.<br>Lack of Funding: Insufficient financial support from governments and organizations.<br>Economic Constraints: Especially significant in countries facing recession.<br>Insurance and Reimbursement Issues: Inconsistent policies across regions.<br><br>- Individual Barriers<br>Lack of Awareness and Knowledge: Both healthcare providers and patients may not understand telemedicine.<br>Trust Issues: Patients may not trust the technology or the security of their data.<br>Motivation and Satisfaction: Low motivation and satisfaction levels among users.<br><br>- Organizational Barriers:<br>Infrastructure Deficiencies: Lack of necessary infrastructure like electricity and internet.<br>Training and Expertise: Insufficient training programs for healthcare professionals.<br>Strategic Planning: Poor planning and management within healthcare organizations.<br>Media Representation: Inadequate promotion of telemedicine benefits.<br><br>- Technological Barriers:<br>System Quality: Issues with the quality of telemedicine systems.<br>Technical Support: Lack of efficient and cost-effective technical support.<br>Internet Connectivity: Problems with connectivity, speed, and costs.<br>Data Management: Challenges in developing and maintaining a centralized patient	

PUBLICATION	TITLE	BARRIERS	FACILITATORS
		database.	
Alshahrani 2019	A systematic review of the adoption and acceptance of eHealth in Saudi Arabia: Views of multiple stakeholders		From the 15 studies included, 39 factors were identified as influences affecting the adoption and acceptance of eHealth in Saudi Arabia from the perspective of multiple stakeholders.<br><br>- Organizational Factors: These relate to the healthcare organizations and facilities, influenced by bureaucracy and policy clarity.<br><br>- Technical Factors: These involve IT infrastructure, eHealth applications, and information security, requiring significant budget for operations and maintenance.<br><br>- Financial Factors: Costs associated with eHealth implementation, including start-up, ongoing, and sustainability funding.<br><br>- Individual Factors: Perceptions, awareness, and acceptance by healthcare professionals and patients.<br><br>- Cultural Factors: Social norms, values, and beliefs affecting eHealth acceptance.<br><br>- Legal Factors: Regulations and policies governing eHealth practices.
Alsobhi 2022	Facilitators and Barriers of Artificial Intelligence Applications in Rehabilitation: A Mixed-Method Approach	- Cost and Available Resources of AI in Clinical Settings:<br>Many respondents identified the high cost of AI implementation as a significant barrier. This includes the cost of AI equipment, treatment fees, development of AI software, and the expense of training both healthcare providers and patients.<br><br>- Compliance and Adoption of AI among Patients and Therapists: Acceptance and adoption of AI: technologies by patients and therapists were seen as major barriers. Factors affecting this include the willingness of patients to trust and use AI systems and the readiness of therapists to integrate AI into their practice.<br><br>- Lack of Knowledge and Proficiency:<br>The lack of adequate knowledge and proficiency among therapists regarding AI	

PUBLICATION	TITLE	BARRIERS	FACILITATORS
		applications was highlighted. This includes the need for more education and training to effectively utilize AI in rehabilitation.<br><br>- Technology Trust in Clinical Settings:<br>Trust in AI technology was another concern. Some therapists expressed scepticism about the reliability and safety of AI systems, which could impact their willingness to adopt these technologies in clinical practice.<br><br>- Infrastructure and Technical Support: The adequacy of infrastructure and technical support required for AI implementation was questioned. This includes the availability of necessary hardware, software, and ongoing technical assistance to maintain AI systems.<br><br>- Legal and Ethical Issues:<br>Legal and ethical concerns related to AI usage were noted. This includes issues around data privacy, patient confidentiality, and the potential legal implications of using AI in clinical settings.<br><br>- Inability of AI to Manage All Patients' Health Conditions or Impairments:<br>A few participants were concerned about AI applications not being customizable enough to cater to the variety of patients' conditions and clinical scenarios. Examples include concerns over AI's ability to handle complex clinical situations and diverse patient needs.	
Alsswey 2021	mHealth technology utilization in the Arab world : a systematic review of systems, usage, and challenges	- User interface:<br>Designing a smart and flexible user interface to make mHealth accessible for patients, particularly considering<br>elderly patients who may encounter physical limitation such as " fat finger" and poor vision<br><br>- Cloud Storage:<br>Integration data transport with cloud storage enables easy responsibility of the transporters for data<br><br>- Platforms:<br>Designing mHealth applications for different properties of phones platforms such as SMS-based system<br><br>- Quality of Service (QoS): The massive number of available smartphones in the world can be possible entry points into the mHealth field. Therefore, achieving the required Quality of Service (QoS) is one of a most secret factor to successful mHealth care applications. Managing bandwidth for massive of smartphones	

PUBLICATION	TITLE	BARRIERS	FACILITATORS
		- Security: The security and privacy should be taken into account such as local communication between the sensors and the smartphone, data transmission between the remote server and local system and the server that stores and processes the health data<br><br>- Data Acquisition: Developing pervasive sensors for the body currently, the sensors are relatively conspicuous and troublesome	
Alzghaibi 2023	An examination of large-scale electronic health records implementation in Primary Healthcare Centres in Saudi Arabia: a qualitative study	- End-User Resistance:<br>Many end-users showed reluctance to use EHRs due to lack of computer literacy and familiarity with the new system. Older users and those without computer experience were particularly resistant. This resistance stemmed from a fear of job security and a preference for paper-based systems.<br><br>- Connectivity Issues:<br>Linking PHCs through the internet was challenging, with many centres facing connectivity problems. This lack of connectivity hindered the implementation process and contributed to the failure of previous EHR projects.<br><br>- Technical Support:<br>Previous implementations suffered from inadequate technical support. Complaints were frequent, and the need for robust, ongoing support from within the country, rather than relying on external vendors, was highlighted.<br>Infrastructure<br><br>- Readiness:<br>The technological infrastructure, including hardware and internet connectivity, was often inadequate. Developing countries like Saudi Arabia face significant challenges in this area compared to developed nations.<br><br>- Management and Leadership:<br>Changes at the administrative level, including ministers and senior managers, disrupted the continuity and focus on EHR projects, leading to termination or delays.<br><br>- Lack of User Training:<br>Insufficient training and awareness campaigns contributed to resistance and low readiness among end-users. There was a need for comprehensive training programs to enhance user competence and acceptance.	- Consultations and Planning:<br>Conducting multi-stage consultations and leveraging the experience of other countries and experts were seen as crucial for avoiding costly mistakes and enhancing the readiness for EHR implementation.<br><br>- Strategic Planning:<br>A well-designed strategic plan, informed by consultations and addressing the specific needs of PHCs, was essential. Such planning included restructuring business processes and ensuring management and administrative support.<br><br>- Training and Support:<br>Implementing training programs, including the "train the trainers" concept and using vendor-provided training, were effective in preparing end-users. Distributing guidance materials also helped in educating users.<br><br>- Technical and Security Measures:<br>Ensuring the security, privacy, and confidentiality of patient data through strict policies, laws, and secure systems was a priority. Monitoring and auditing by PHC directors, along with limited access privileges based on user roles, were key strategies.<br><br>- End-User Involvement:<br>Involving end-users and stakeholders

PUBLICATION	TITLE	BARRIERS	FACILITATORS
			throughout the project, from planning to post-implementation, helped in reducing resistance and ensuring the system met user needs. Representatives from various departments and levels participated in decision-making processes.<br><br>- Project Team Selection and Communication:<br>Selecting a well-qualified project team and ensuring effective communication through committees, workshops, and regular meetings were critical. This included using various media for communication and involving end-users in these processes.
Antonacci 2023	Healthcare professional and manager perceptions on drivers, benefits, and challenges of telemedicine: results from a cross-sectional survey in the Italian NHS	- Technological Challenges:<br>Poor quality of internet connection and other technologies.<br>Difficulty in using technology and technological literacy issues among patients and healthcare professionals.<br>Limited access to necessary equipment such as cameras, smartphones, and internet connectivity, especially in rural areas.<br><br>- Socio-Cultural and Linguistic Barriers:<br>Socio-cultural factors, including resistance to change among certain patient groups.<br>Language barriers and lack of caregivers to assist patients, particularly the elderly and people with disabilities.<br><br>- Regulatory and Organizational Challenges:<br>Fragmented legislation and inconsistent reimbursement schemes across different regions.<br>Complexity in technology purchasing processes and lack of clear data governance and privacy guidelines.<br>Poor integration between telemedicine and traditional care processes leading to duplication of activities and additional burdens on staff.<br><br>- Digital Literacy and Training:<br>Poor digital literacy among both patients and healthcare providers.<br>Lack of adequate training for healthcare providers on telemedicine technologies and their use in clinical practice.	- Improved Access to Care:<br>Telemedicine facilitates access to healthcare services for patients in remote and underserved areas, reducing the need for travel and associated costs.<br>It helps in maintaining continuity of care, especially during emergencies like the COVID-19 pandemic.<br><br>- Operational and Organizational Benefits:<br>Positive impact on operational efficiency by reducing patient wait times and missed appointments.<br>Enhanced flexibility for healthcare providers in managing their schedules and patient availability.<br><br>- Positive Attitude and Satisfaction:<br>Both patients and healthcare providers generally have a positive attitude towards telemedicine, acknowledging its potential to improve care delivery and patient outcomes.<br><br>- Regulatory Support and Financial

PUBLICATION	TITLE	BARRIERS	FACILITATORS
			Incentives:<br>Easing of regulatory barriers and provision of financial incentives during the pandemic to support the rapid adoption of telemedicine.<br>Government and regional funding to develop telemedicine infrastructure and services.
Aranha 2021	Exploring the Barriers and Facilitators which Influence mHealth Adoption among Older Adults: A Literature Review	- Dispositional Barriers:<br>Low Self-Efficacy: Many older adults lack confidence in their ability to use mHealth technologies, leading to anxiety and reluctance to adopt these tools.<br><br>- Mistrust of Technology: Concerns about privacy, security, and potential misuse of their personal information deter many older adults from using mHealth applications.<br><br>- Age-Related Physical and Cognitive Impairments: Declining physical abilities, such as impaired vision and dexterity, as well as cognitive decline, make it challenging for older adults to use mHealth tools effectively.<br><br>-Usability Features:<br>Complex Interfaces: Many mHealth applications have complicated interfaces that are not intuitive for older users, making them difficult to navigate and use.<br><br>- Small Device Size: The small size of mobile devices can be difficult for older adults with fine motor skill issues and visual impairments.<br><br>- Social Influence:<br>Lack of Support: Older adults often rely on family and healthcare providers for support in adopting new technologies. A lack of encouragement and assistance from these sources can hinder mHealth adoption.<br><br>- Negative Past Experiences: Previous negative experiences with technology, such as difficulties with early personal computing, contribute to a general aversion to new technological tools	- Social Support:<br>Family and Caregiver Support: Encouragement and technical support from family members and caregivers can significantly enhance the willingness of older adults to adopt mHealth technologies.<br><br>- Healthcare Provider Recommendations: Trust in healthcare providers means that their endorsement of mHealth tools can positively influence older adults' adoption of these technologies.<br><br>- Perceived Usefulness:<br>Health Benefits: Older adults are more likely to adopt mHealth technologies if they perceive clear benefits, such as improved health monitoring, better management of chronic conditions, and enhanced overall health outcomes.<br><br>- Tailored Applications: Applications that address specific needs of older adults, such as medication reminders for those with memory impairments, are more likely to be adopted
Ashtarian 2023	Popular diffusion as an instrument for overcoming barriers to digital health in Iran: the critical role of the pandemic	Government Incapacity for Digital Health Governance: The lack of a clear strategy, regulations, and knowledge regarding digital health led to opposition from governmental bodies.<br><br>Conflict of Interest: The presence of conflicting interests between public officials and digital health startups hindered collaboration.	Popular Diffusion and Passive Government Attitude: The widespread adoption of digital health applications, especially during the COVID-19 pandemic, forced the government to accommodate these services due to public demand.

PUBLICATION	TITLE	BARRIERS	FACILITATORS
		Professional Obligations for Information Transparency: Lack of transparency in health data and the inability to verify the credentials of healthcare providers on digital platforms caused trust issues.<br><br>Protection of Patients' Rights: Inadequate mechanisms to protect patient rights, such as ensuring the digital identity of healthcare providers, raised concerns about service authenticity.<br><br>Data Security and Privacy: Concerns about data privacy and the potential for patient information to be exposed or misused by digital platforms.	Lower Cost: The affordability of digital health services compared to in-person consultations, particularly during the pandemic, encouraged greater use of telemedicine.
Babaei 2023	A scoping review of virtual care in the health system: infrastructures, barriers, and facilitators	- Technological Barriers:<br>Lack of IT facilities and equipment: Many organizations struggle with insufficient IT infrastructure, lack of high-speed internet, and inadequate access to the necessary technology and equipment.<br>Technological literacy: Both patients and healthcare providers often face challenges in using new technologies effectively, which includes difficulties with installing applications, using devices like smartphones and tablets, and navigating software platforms.<br>Poor internet connectivity: Especially in rural areas, limited access to reliable internet services hampers the effective implementation of virtual care.<br><br>- Social and Cultural Barriers:<br>Digital divide: Inequities in access to technology are pronounced among older adults, low-income families, and people with disabilities, affecting their ability to benefit from virtual care services.<br>Privacy and security concerns: Fear of data breaches and concerns over how personal health information is stored and used can discourage the adoption of virtual care.<br>Socio-cultural factors: Socio-cultural and linguistic challenges, as well as the absence of caregivers, significantly impact the adoption of telemedicine services.<br><br>- Organizational Barriers:<br>Lack of legal and regulatory frameworks: The absence of clear regulations and policies for e-health, telemedicine, and the use of virtual care platforms poses a significant barrier.<br>Workflow integration: Integrating telemedicine into existing clinical workflows can be complex, leading to additional burdens on healthcare providers and potential duplication of tasks.	- Technological Facilitators:<br>Development of robust IT infrastructure: Enhancing technological infrastructures, such as ensuring high-speed internet access and providing the necessary hardware and software, is crucial for the effective implementation of virtual care.<br>User-friendly platforms: Designing intuitive and accessible digital health platforms that cater to the needs of both healthcare providers and patients can facilitate the adoption of virtual care.<br><br>- Educational and Training Facilitators:<br>Training programs: Providing comprehensive training for healthcare providers, patients, and their families on how to use virtual care technologies effectively can mitigate many of the challenges related to technological literacy.<br>Patient education: Creating patient-facing materials that assist in preparing for and navigating virtual sessions can improve the user experience and increase adoption rates.<br><br>- Organizational Support:<br>Policy and regulatory support: Developing clear regulations and guidelines for virtual care, along with ensuring compliance with privacy and

PUBLICATION	TITLE	BARRIERS	FACILITATORS
			data security laws, can foster trust and encourage widespread adoption.<br>Financial support: Allocating budgets for the purchase of necessary technology and the maintenance of virtual care platforms is essential for sustained implementation.<br><br>- Social and Cultural Facilitators:<br>Involving caregivers: Engaging caregivers in the virtual care process, especially for elderly and disabled patients, can help overcome many socio-cultural and logistical barriers.<br>Building trust: Ensuring transparency in how data is stored and used, and addressing ethical and privacy concerns can build trust among users.
Baines 2021	Meaningful patient and public involvement in digital health innovation, implementation and evaluation: A systematic review	- Time and Financial Constraints:<br>PPI (Patient and Public Involvement) in digital health innovation is time-consuming and resource-intensive, often limiting how much patients can be involved in the process.<br><br>- Technological Limitations:<br>Issues such as low-quality internet connection, poor technological infrastructure, and lack of IT facilities and equipment hinder the implementation of digital health solutions<br><br>- Disconnect Between Developers and End Users:<br>There is often a disconnect between the design process and the actual needs of the users, leading to products that may not be well-suited to the market when launched.<br><br>- Regulatory and Ethical Issues:<br>Lack of clear legal frameworks, privacy concerns, and ethical issues pose significant barriers to the adoption of virtual care and digital health solutions.<br><br>- Lack of Digital Literacy:<br>Both healthcare providers and patients often lack the necessary skills to effectively use digital health technologies, which limits their adoption and effective utilization.<br><br>- Socio-Cultural Barriers:<br>Socio-cultural factors, including language barriers and the absence of caregivers, impact the use of telemedicine, especially among elderly and disabled populations.	- Technological Advancements<br>The development and deployment of robust IT infrastructure and digital health technologies can significantly enhance the delivery of virtual care.<br><br>- Organizational Support:<br>Organizational backing, including budget allocation and policy support, is crucial for the successful implementation of digital health initiatives.<br><br>- Training and Education: Providing: comprehensive training for healthcare providers and patients on using digital health technologies can facilitate their adoption and effective use.<br><br>- Patient and Public Involvement (PPI):<br>Early and meaningful involvement of patients in the design and implementation of digital health solutions can ensure that the products meet their needs and are more likely to be accepted and used.

PUBLICATION	TITLE	BARRIERS	FACILITATORS
		- Equity of Access<br>There are significant disparities in access to digital health services, particularly in rural areas and among low-income populations, which exacerbates existing health inequalities.	- Flexible and Adaptive Systems: Developing flexible and adaptive digital health systems that can be customized to meet the varying needs of different patient groups can enhance the effectiveness of virtual care.<br><br>- Regulatory Support: Streamlining regulations and providing clear legal frameworks for the use of telemedicine and other digital health services can facilitate their broader adoption
Balasubramanian 2023	Applying artificial intelligence in healthcare: lessons from the COVID-19 pandemic	- Technological Limitations:<br>Data Quality and Availability: AI models require large, high-quality datasets to function accurately. Limited access to comprehensive, standardized datasets can hinder AI model training and performance.<br>Infrastructure Requirements: The adoption of AI necessitates significant technological infrastructure, including powerful computational hardware, advanced software, and high-speed internet. Many healthcare facilities, especially in low-income regions, lack these resources.<br><br>- Regulatory and Ethical Concerns:<br>Privacy and Security: Handling sensitive health data raises significant privacy and security concerns. There is a need for robust legal frameworks to protect patient data and ensure ethical use of AI in healthcare.<br>Lack of Standardized Regulations: Inconsistent regulatory environments across different regions can create barriers to the widespread adoption of AI technologies in healthcare.<br><br>- Algorithmic Bias and Generalizability:<br>Bias in AI Models: AI models can exhibit biases based on the data they are trained on, which may lead to unfair or inaccurate outcomes in different populations.<br>Generalizability Issues: AI models developed in one context may not perform well in another due to differences in patient demographics, clinical practices, and available data.<br><br>- Stakeholder Resistance:<br>Resistance to Change: Healthcare providers and patients may be resistant to adopting new AI technologies due to a lack of understanding, fear of job displacement, or preference for traditional methods.<br>Training and Acceptance: There is often insufficient training for healthcare professionals on how to effectively use AI tools, leading to reluctance or misuse of	- Technological Advancements:<br>High-Quality Data and Advanced Algorithms: The development of sophisticated AI algorithms and the availability of comprehensive, high-quality datasets can significantly enhance AI applications in healthcare.<br>Improved Computational Power: Advances in computational power and infrastructure facilitate the deployment of complex AI models capable of handling large datasets and providing accurate predictions.<br><br>- Regulatory and Policy Support:<br>Supportive Legal Frameworks: The establishment of clear, robust legal and regulatory frameworks can facilitate the ethical and secure adoption of AI technologies in healthcare

PUBLICATION	TITLE	BARRIERS	FACILITATORS
		these technologies.	
Bally 2020	Toward Integration of mHealth in Primary Care in the Netherlands: A Qualitative Analysis of Stakeholder Perspectives	- Inadequate Set of Core Standards:<br>Lack of specific regulation on mHealth to ensure privacy and data security standards are met.<br>Difficulty in ensuring health information from mHealth applications complies with privacy standards.<br>Need for universal standards for enabling health information exchange .<br><br>- Difficulties in Informing End-Users on mHealth:<br>Obstacles in informing healthcare consumers and professionals about the possibilities of mHealth.<br>The challenge of validating and measuring the effectiveness of numerous health applications using traditional clinical trial methods .<br><br>- Financing Issues:<br>Lack of time and resources to establish financial flows for mHealth implementation. Difficulties in obtaining funding for mHealth projects. Insurers' uncertainty about return on investment leading to reluctance in providing funding .<br><br>- Risk of Losing Personal Contact:<br>Concerns among GPs about losing the benefits of face-to-face consultations, which can help in picking up non-verbal cues relevant to treatment. Preference among older patients for personal contact with their GP .<br><br>- Risk of Misinterpreting Health Data:<br>Concerns about the quality and validity of self-measured data by patients. Difficulties healthcare providers face in making sense of the data collected by patients, leading to potential misinterpretations .<br><br>- Lack of Knowledge and Digital Skills:<br>GPs and healthcare providers are poorly informed about the possibilities of mHealth. Insufficient knowledge on how to adopt and use health technologies.<br>Digital skills gap, especially among older generations, making it difficult to use mHealth technologies effectively .<br><br>- Negative Attitudes of Stakeholders:<br>Attitudes of stakeholders, particularly GP information system developers, towards the adoption of mHealth are often negative, hindering integration efforts.	- Encourage Reflexive Learning:<br>Using reflexive learning approaches that include multiple stakeholders and gathering evidence to support mHealth integration .<br><br>- Good Working mHealth Solutions:<br>Development of high-quality mHealth solutions tailored to the needs of end-users.<br>Focus on usability and user-experience, ensuring applications are easy to use and align with daily life situations.<br>Harmonizing the use of validated apps by aligning high-quality applications in one easily accessible portal .<br><br>- Education and Support:<br>Education on the possibilities of mHealth and operational support on how to implement mHealth services.<br>Providing knowledge and resources to healthcare providers to support the adoption of mHealth technologies .<br><br>- Collaboration and Incentives:<br>Collaboration between various stakeholders, including policymakers, healthcare providers, and mHealth developers.<br>Providing incentives for pioneers and early adopters to encourage the integration of mHealth.<br><br>- Gathering Evidence:<br>Demonstrating the efficacy and cost-effectiveness of mHealth services through practical evaluation methodologies, including clinical, patient-reported, and economic outcomes .
Baradwan 2023	Perceived Knowledge,	- Resistance:	

PUBLICATION	TITLE	BARRIERS	FACILITATORS
	Attitudes, and Barriers Toward the Adoption of Telemedicine Services in the Kingdom of Saudi Arabia: Cross-Sectional Study	45.31% (464/1024) - physician resistance is a barrier to the practice of telemedicine, 43.55% (446/1024) patient resistance is a barrier to the practice of telemedicine, 39.65% (406/1024) diagnostic reliability is a barrier to the practice of telemedicine<br><br>- Limitations<br>to the adoption of telemedicine pertaining to culture 37.3% (382/1024) and technology 42.87%<br>(439/1024)	
Bele 2021	Using the Theoretical Domains Framework to Identify Barriers and Enablers to Implementing a Virtual Tertiary-Regional Telemedicine Rounding and Consultation for Kids (TRaC-K) Model: Qualitative Study	awareness of telemedicine:<br>limited awareness about the use of telemedicine in paediatric clinical care.<br><br>- skills to provide virtual clinical care:<br>lack of skills to communicate over the screen. lack of clinical assessment skills to provide care over the screen. lack of technical skills.<br><br>- processes and resources to support trac-k:<br>unclear processes as a potential source of harm. challenges in clinical circumstances.<br><br>physical environment issues. absence of dedicated personnel.<br><br>difficulties in scheduling. paucity of professional guidelines.<br><br>- provider roles and responsibilities:<br>concerns about clear roles and responsibilities. lack of workflow integration. increased workload and competing priorities.	- Motivation to Provide Care Closer to Home:<br>Desire to provide care closer to home.<br>Confidence in TRaC-K.<br>High importance placed on the model.<br>Excitement about the new approach.<br><br>- System Resource Stewardship:<br>Balancing provincial resources.<br>Ability to provide tertiary-level care at regional sites.<br>Redistribution of patient load and resources; care closer to homes.<br><br>- Site and Practice Compatibility:<br>Compatibility with current practice.<br>Motivation to strengthen tertiary–regional relationships.
Bertolazzi 2024	Barriers and facilitators to health technology adoption by older adults with chronic diseases: an integrative systematic review	- Demographic and Socioeconomic Factors:<br>Older age, low education, limited/fixed income, cost, limited space at home for the system.<br><br>- Health-related Factors:<br>Poor learning skills, cognitive impairments, sensory deficits, severe anxiety about the illness, complexity of health conditions, type of medical condition.<br><br>- Dispositional Factors:<br>Scepticism about the accuracy of the results, judging oneself or being judged negatively by physicians when one does not monitor data, aversion to/difficulty learning how to operate new technology, fear of using new technology, lack of confidence and technical skills/unfamiliarity with the technology/computer anxiety, previous negative experiences with the technology, lack of need for technology, lack of motivation, interest, or personal inertia, established routines and a conservative mentality.	- Demographic and Socioeconomic Factors:<br>Higher level of education, cost-effectiveness.<br><br>- Health-related Factors:<br>Awareness and a better understanding of the illness, adoption of technology in the early stages of the disease, being monitored continuously by healthcare providers and receiving timely care, improving the self-management of chronic diseases, perceiving an improvement in medical condition, lifestyle, and quality of life.

PUBLICATION	TITLE	BARRIERS	FACILITATORS
		- Technical Factors:<br>Technical issues, privacy and safety concerns, lack of technical support, poorly designed interface, overload/complicated information, not having the required equipment, a lack of interactive design features.<br><br>- Social Factors:<br>Fear of a weakening of social relations with relatives and healthcare providers, needing others to use the technology, social stigma about aging and reliance on technology.	- Dispositional Factors:<br>Trustworthiness and reliable information, willingness to learn, technological self-efficacy, previous experience with the technology, motivation/interest to use the technology, personal enjoyment, competitive characteristics, perceived usefulness, higher level of health literacy.<br><br>- Technical Factors:<br>Perceived ease of use, privacy is not a concern, comfortability of wearable technology, patient involvement in the design process, self-tracking functions, adequate training, likeable appearance, clarity in the presentation and organisation of information, natural speech and eye contact with the robot, technical helpdesk.<br><br>- Social Factors: Connectedness to healthcare providers, connectedness to other people, support from partners and relatives in the use of technology, patient’s significant others’ and physician’s recommendations for using technology.
Blandi 2023	The potential of digital health records for public health research, policy, and practice: the case of the Lombardy region data warehouse	- Inaccuracy:<br>Data might be inaccurate, resulting in a loss of predictive power. The extent and bias of the noise could require different methods for data analysis.<br><br>- Incompleteness:<br>Incomplete data can impede comprehensive analysis.<br><br>- Biases:<br>There are inherent biases in the healthcare recording process that need to be managed.<br><br>- Lack of Data Quality Analysis:<br>No thorough data quality analysis has been undertaken on DWH data, which may lead to uncertainties in research findings.<br><br>- Compliance with Legislation: Health data integration must adhere to national and	- Use of Validated Algorithms:<br>Researchers are encouraged to use validated algorithms from national and international literature or from good practices to control potential errors and biases.<br><br>- Anonymisation:<br>Personal data is anonymized before reuse for public interest purposes, protecting individual privacy while allowing data usage for research.<br><br>- Ethical Considerations:<br>Ethical considerations are emphasized,

PUBLICATION	TITLE	BARRIERS	FACILITATORS
		European privacy laws, which sometimes limit the potential of data integration.<br><br>Privacy Impact Assessments:<br>High-risk assessments must be conducted to ensure the protection of individual rights, which can delay or complicate data usage.<br><br>- Regulatory Limitations:<br>Current laws do not permit the implementation of advanced tools like Artificial Intelligence in predictive medicine, limiting scientific progress.	ensuring that the reuse of data is compliant with the rights and fundamental liberties of individuals.<br><br>Structured Process:<br>- Lombardy Region conducts a privacy impact assessment before processing personal data, ensuring compliance with privacy regulations.
Blondino 2024	The use and potential impact of digital health tools at the community level: results from a multi-country survey of community health workers.	- Limited or no internet connectivity: 689 (60.4%)<br>- Cost of internet services: 600 (52.6%)<br>- Cost of mobile phone services: 546 (47.9%)<br>- Cost of phone/device: 457 (40.1%)<br>- Limited experience with technology/not knowing how to use technology: 396 (34.7%)<br>- Prefer traditional face-to-face interaction: 294 (25.8%)<br>- Limited or no electricity/power source: 274 (24.0%)<br>- Distrust in technology: 95 (8.3%)<br>- No particular barriers: 34 (3.0%)	
BorgesdoNascimento 2023	Barriers and facilitators to utilizing digital health technologies by healthcare professionals	Infrastructure and Technical Barriers: Limited network, insufficient technology, device shortages, workflow compatibility, connectivity speed, and standardization issues, especially in rural areas. Recommendations: Ensure high-speed connectivity, reliable local networks, and involve healthcare professionals in tech development.<br><br>Personal and Psychological Barriers: Resistance to change, technophobia, low literacy, and fear of using technology. Recommendations: Enhance perception of usefulness and willingness to use digital health technologies.<br><br>Fear of Increased Working Hours and Workload: Concerns about increased workload hinder adoption.<br><br>Legal and Ethical Features: Legal and ethical issues interfere with technology adoption.<br><br>Lack of Training and Educational Programs: Negative experiences due to insufficient training decrease usage.<br><br>Healthcare System Structure and Financial Constraints: Structural and financial issues limit technology use.<br><br>Interoperability and Data Incompatibility: Issues with data sharing and compatibility hinder adoption. Infrastructure and technical barriers: (6.4% [95% CI 2.9–14.1])	- Offering training and educational activities: (3.8% [95% CI 1.6–9.0])<br>- Perceived usefulness and willingness to use DHTs: (3.8% [95% CI 1.8–7.9])<br>- Government and multisector incentives: (3.0% [95% CI 1.4–6.6])<br>- Adherence promotion campaigns: (2.2% [95% CI 1.1–4.3])<br>- Involvement in the development and implementation of DHTs: (2.0% [95% CI 0.8–4.9])<br>- Easy-to-use and intuitive navigation systems: (1.9% [95% CI 0.7–5.2]) ;<br>Offering Training and Educational Activities: Improves user experience and adoption of digital health technologies.<br><br>Perceived Usefulness and Willingness to Use: Positive perception and willingness to use are crucial facilitators.<br><br>Government and Multisector Incentives: Incentives promote adoption.

PUBLICATION	TITLE	BARRIERS	FACILITATORS
		- Psychological and personal issues: (5.3% [95% CI 2.2–12.7])<br>- Fear of increased working hours and workload: (3.9% [95% CI 1.5–10.1])<br>- Legal and ethical features: (3.6% [95% CI 1.3–9.6])<br>- Lack of training and educational programs: (3.4% [95% CI 1.3–8.9])<br>- Healthcare system structure and lack of financial support: (2.9% [95% CI 1.0–8.3])<br>- Interoperability and data incompatibility: (2.2% [95% CI 1.0–5.0])	Adherence Promotion Campaigns: Campaigns enhance adherence and use of technologies.<br><br>Involvement in Development and Implementation: Involvement ensures technologies meet user needs.<br><br>Intuitive Navigation Systems: Easy-to-use systems facilitate adoption and continued use.
Butt 2023	5G and IoT for Intelligent Healthcare: AI and Machine Learning Approaches - A Review	- Data Security and Privacy:<br>Patient data is generated in large quantities due to the interconnected nature of 5G, IoT, AI, and ML in healthcare, which raises concerns about data security and privacy. Maintaining patient trust and meeting legal standards necessitate the implementation of stringent data security and privacy protection procedures. Protecting sensitive patient data requires the use of cutting-edge encryption methods, safe data storage, and rigorous authentication procedures.<br><br>- Standardization and Interoperability:<br>Data formats, communication protocols, and interfaces need to be standardized to allow for the seamless integration of different technologies. It is crucial to facilitate efficient data interchange, cooperation, and integration across diverse healthcare devices, systems, and platforms by achieving seamless interoperability. The efficient sharing of data between healthcare facilities is dependent on the development of standardized protocols that allow for interoperability between those facilities.<br><br>- Ethical Considerations:<br>Concerns about algorithm bias, transparency, and responsibility arise when using AI and Machine Learning algorithms. If we don't want to see unfair healthcare inequities and biased decisions, we need to make sure that AI algorithms are fair, transparent, and interpretable. The proper application of AI in healthcare calls for the establishment of ethical frameworks, norms, and regulatory frameworks.<br><br>- Infrastructure Investment:<br>To integrate 5G, IoT, AI, and ML in healthcare, a substantial investment in infrastructure is required. This includes things like 5G network coverage, dependable connectivity, and enough computing resources. There may be monetary difficulties associated with investing in new healthcare technology and upgrading old facilities. To guarantee widespread adoption and accessibility, these technologies need to have their infrastructure and costs addressed.	

PUBLICATION	TITLE	BARRIERS	FACILITATORS
		- Training and Education:<br>Integration of cutting-edge healthcare technologies calls for a trained staff with the requisite expertise. Effective use of AI and data analytics in clinical practice requires training for healthcare personnel. The full potential of these technologies can only be realized if healthcare practitioners' skill gaps are closed through training programs and educational activities.	
Byambasuren 2019	Current knowledge and adoption of mobile health apps among Australian general practitioners: Survey study	- Lack of Knowledge of Effective Apps: The most significant barrier identified by GPs, with 372 (60%) citing this as the main issue.<br><br>- Lack of Trustworthy Sources: Another major barrier, with 96 (15%) GPs indicating difficulty in finding reliable sources to access effective apps.<br><br>- Lack of patient interest (33% main barrier)<br><br>- Consultation Time Constraint: Additional barrier noted by 24 (28%) GPs, highlighting the time required to discuss and integrate apps during consultations.<br><br>- Uncertain Benefits and Interest: Reported by 19 (21%) GPs as a barrier due to unclear advantages and varying levels of interest in health apps.<br><br>- Cost: Considered a minor barrier, mentioned by only 3 (3%) GPs.	What Would Help GPs Recommend Health Apps More Often<br><br>- More Awareness, Knowledge, or Training: 243 responses (30%)<br>- List of Approved or Vetted Apps: 224 responses (28%)<br>- Evidence of Benefits or Quality: 92 responses (12%)<br>- Nothing: 44 responses (5%)<br>- More Time: 39 responses (5%)<br>- Practice Incentives: 29 responses (4%)<br>- Integration with Practice Software: 26 responses (3%)
Byambasuren 2020	Barriers to and facilitators of the prescription of mhealth apps in Australian general practice: Qualitative study	General Practitioners' Perceived Barriers:<br><br>- Generational Differences: There is a digital divide between older and younger providers and patients.<br>- Lack of Knowledge: GPs are often unaware of prescribable apps and trustworthy sources to access them.<br>- Time Commitment: Both providers and patients need significant time to learn and use the apps.<br>- Concerns: Issues regarding the privacy, safety, and trustworthiness of health apps are major concerns.<br><br>Patients' Perceived Barriers_<br>- Older Age: Older patients find it difficult to use mHealth apps.<br>- Usability Issues: Apps may not be user-friendly for all patients.	General Practitioners' Perceived Facilitators:<br><br>- Trustworthy Sources: Access to reliable sources for prescribable apps and information.<br>- Younger Generation: Younger patients are more receptive to using health apps. Smartphone<br>- Ownership: The widespread ownership of smartphones supports app usage.<br><br>Patients' Perceived Facilitators:<br>- Smartphone Ubiquity: The widespread use of smartphones and apps, especially among the younger generation.<br>- Doctor Recommendations: Patients are more likely to use health apps if recommended by their doctors.<br>- Lack of Privacy Concerns: Unlike GPs,

PUBLICATION	TITLE	BARRIERS	FACILITATORS
			patients are generally not concerned about privacy and data safety issues with health apps.
Calleja 2021	Telehealth use in rural and remote health practitioner education: an integrative review	- Technical issues include poor audio and video quality, overloaded servers, and difficulty accessing education on mobile devices.<br><br>- User complexity presents challenges for end users to complete the education.<br><br>- Session availability is limited by the availability of session times and the infrastructure and training required for clinicians to use the equipment.<br><br>- Logistical challenges encompass being released from clinical work to attend education, session lengths that conflict with clinical needs, lack of time to use resources identified in the education, and scheduling clashes.	- Improved Practice Change:<br>Focused on the changed practices of clinicians as a result of engaging in the education provided.<br>Linked to improved patient outcomes, clinician confidence, knowledge, and self-efficacy.<br><br>- Increased Clinical Competency:<br>Related to tested clinical competence, whether tested or self-reported.<br>Sometimes extended the scope of practice of the clinician.<br><br>- Sense of Community and Interaction:<br>Decreases isolation by enabling collaboration with peers and access to support.<br>Results in feeling more connected and reduced stress.<br><br>- Improved Patient Outcomes:<br>Related to measurable patient clinical outcomes.<br>Involves being able to access healthcare in rural or remote environments for a broader scope of care.<br><br>- Satisfaction, Activity, and Reach:<br>Related to the acceptability and feasibility of the education provided.<br>Would benefit from more robust outcomes-based research.<br><br>- Successful VC Education:<br>Focused on the relevance of the education to the target audience, being interactive, providing a mixture of educational approaches, and being tailorable to local

PUBLICATION	TITLE	BARRIERS	FACILITATORS
			needs.<br>Included specific educator skills and characteristics, such as being knowledgeable, approachable, comfortable with equipment, and creating a confidential, safe, and comfortable learning environment.<br>Also included training for educators on adapting teaching style to VC sessions, technological skills, accessibility for questions, and consistency of session information.<br><br>- VC Education Acceptability and Feasibility:<br>Reported satisfaction of participants.<br>Enables attendance at education sessions, decreases time and money required to access education.<br>Offers additional benefits by providing frequent VC education versus sporadic face-to-face education.<br><br>- Technology's Role in Education:<br>Can support or hinder education in rural and remote communities.<br>Depends on the availability of support and infrastructure to troubleshoot issues and the quality of the initial setup of easy-to-use systems.
Cannavacciuolo 2022	Digital innovation and organizational changes in the healthcare sector: Multiple case studies of telemedicine project implementation	- Technology Support (Case 3):<br>Internal Provider Issues: Inadequate support from the corporate ICT department caused slowdowns in project development. Lack of timely collaboration led to reliance on the internal project team’s IT knowledge, which hindered the full utilization of the platform and delayed implementation.<br><br>- Acceptance (General):<br>Patient Resistance: Low number of patient bookings when the telemedicine service went live online, attributed to preconceptions about remote healthcare provision. Patients preferred in-person interactions for greater reassurance and direct communication with healthcare personnel.	- Technology Support (Case 1):<br>External Provider Collaboration:<br>Effective partnership with an external technology provider who analysed requirements, provided a suitable platform, and supervised training.<br>Continuous support positively impacted usability, training, and the quality of the information infrastructure.<br><br>Acceptance (General):<br>Previous Experience and Dissemination:

PUBLICATION	TITLE	BARRIERS	FACILITATORS
		- Organization (Case 3):<br>Coordination Issues: Lack of coordination and support from the internal ICT provider affected the interconnection of different departments (e.g., pharmacy), leading to implementation slowdowns.<br><br>- Policy and Legislation:<br>Neutral Impact: Delays in regional actions to promote telemedicine and the absence of formal top-down procedures or standard tariffs at the operational phase posed potential future hindrances.	Participation in previous telemedicine projects and internal dissemination of successful practices enhanced acceptance and trust among project team members and healthcare personnel.<br>Strong Leadership: Project managers played a crucial role in encouraging and supporting teams, promoting telemedicine projects, and maintaining good relationships with stakeholders.<br><br>- Organization (General):<br>Procedural Clarity and Coordination: Development of organizational procedures and clear data/information flow between different areas facilitated collaboration and the digitalization of patient treatment.<br><br>- Financing:<br>Sufficient Initial Funding: Affordable technology costs allowed projects to be financed with internal resources or public funds, ensuring smooth project initiation without significant financial barriers
Carlqvist 2021	Health care professionals' experiences of how an eHealth application can function as a value-creating resource - a qualitative interview study	Technical Issues:<br>A common barrier was technical difficulties with telemonitoring devices. Some patients struggled with the technology, which caused frustration and interrupted data transmission. Issues like incorrect measurements or failure of devices to sync created additional work for healthcare professionals.<br><br>Workload Increase:<br>Healthcare professionals, especially nurses, reported that telemonitoring added to their workload. Monitoring patients' data daily took extra time, and the system was not fully integrated into their workflow, creating inefficiencies.<br><br>Limited Patient Capability:<br>Not all patients were able to use the technology independently. Those with limited digital literacy or cognitive impairments needed assistance from relatives or healthcare providers. This dependency increased the workload on the healthcare system and limited the reach of telemonitoring.	Improved Communication:<br>Telemonitoring applications facilitated better communication between healthcare professionals and patients. Video meetings and chats allowed for more personalized care, making it easier for healthcare providers to track patients' health remotely and offer timely advice.<br><br>Proactive Care:<br>The ability to monitor patients' vital signs in real time helped healthcare professionals take proactive steps to manage chronic conditions, reducing the need for emergency visits and hospitalizations. This was especially beneficial for patients with heart failure,

PUBLICATION	TITLE	BARRIERS	FACILITATORS
		Lack of System Integration:<br>A major barrier was the lack of integration between the telemonitoring system and the electronic health record (EHR) system. This caused inefficiencies and added extra steps for healthcare professionals, making the process more time-consuming.<br><br>Alerts and Notifications:<br>Some applications lacked important features like alerts for abnormal measurements, which reduced their usefulness. On the other hand, systems that sent too many notifications overwhelmed healthcare professionals, making it hard to prioritize important alerts.	diabetes, and hypertension.<br><br>Patient Engagement:<br>Telemonitoring encouraged patients to take an active role in managing their health. By tracking their own vital signs, patients became more involved in their care, which improved adherence to treatment and increased their knowledge about their conditions.<br><br>Enhanced Safety and Trust:<br>Both healthcare professionals and patients felt that telemonitoring increased safety. Patients felt reassured knowing their health was being monitored remotely, and healthcare providers could intervene early when signs of deterioration appeared, fostering a sense of trust in the system.<br><br>Flexibility:<br>Telemonitoring applications gave patients the flexibility to manage their health at home, making it easier for those in rural or remote areas to access healthcare. This increased continuity of care and reduced the need for frequent in-person visits.
Cascini 2023	Strengthening and promoting digital health practice: results from a Global Digital Health Partnership's survey	Cumulative scores on barriers to implement digital health use (from highest to lowest)<br>- accessibility of the population<br>- scepticism of clinicians<br>- lack of organisation<br>- lack of economic resources<br>- lack of infrastructure<br>- lack of technological equipment<br>- limited sills of the population<br>- lack of political will<br>- others	
Chan-Nguyen 2022	Patient and caregiver perspectives on virtual care: a patient-oriented qualitative study.	- Major theme: Access to Technology and Internet are Barriers<br>Minor Themes: No or limited access to technology or Internet, rapid technology changes challenging and overwhelming, digital literacy can affect virtual care access, inability to afford technology or Internet is a barrier	

PUBLICATION	TITLE	BARRIERS	FACILITATORS
		- Major Theme 2: Language Barriers and Cultural Differences Can Affect Virtual Care<br>Minor Themes: Inability to express concerns, cultural barriers could affect knowledge of virtual care, traditional and digital literacy important to virtual care<br><br>- Major Theme 3: Caregiver and Family Involvement<br>Minor Themes: Caregiver support is crucial to virtual care access, heavy burden on caregiver to provide care and support virtual care access, nuances of caregiving must be considered<br><br>- Major Theme 4: Privacy, Consent, and Confidentiality Considerations<br>Minor Themes: Appointments can include sensitive information that patients want to discuss privately, tension could arise between patients and caregivers because of lack of understanding or agreement over confidentiality, caregiver opinion should not be taken at greater value<br><br>- Major Theme 5: Different Patient–Physician Relationship<br>Minor Themes: Patients feel they did not have a personal connection anymore, mechanical interaction with health care provider virtually<br><br>Major Theme 6: Future Research Directions or Calls to Action<br>Minor Themes: Technology and Internet must be universally accessible, integration of virtual care modalities will improve patient care (e.g., consolidated patient portal)	
Chibuike 2024	Overcoming Challenges for Improved Patient-Centric Care: A Scoping Review of Platform Ecosystems in Healthcare	- Privacy and Security Concerns: The sensitive nature of health data raises substantial concerns about unauthorized access and breaches, posing threats to the effectiveness and viability of digital platforms.<br><br>- Interoperability Issues: The need for seamless data exchange among diverse healthcare technologies necessitates clear standards and system compatibility, which is often lacking.<br><br>- User Resistance and Adoption Barriers: Technological resistance and patient adoption issues persist, especially in developing countries, despite increased adoption during the COVID-19 pandemic. Overcoming resistance to change and fostering user engagement remain challenging.<br><br>- Data Quality and Management: Accurate diagnostics and therapeutics rely on high-quality data, but misinterpretation and unreliable data can compromise patient safety. Ensuring data accuracy and effective management is critical.<br><br>- Regulation: The stringent regulatory environment in healthcare requires digital platforms to navigate complex privacy and data protection laws while fostering	- Data Governance and Standards: Implementing robust data governance policies and adhering to data protection laws and standards can address privacy and security concerns, promoting ethical information exchange.<br><br>- Adaptive Interoperability Frameworks: Developing adaptive frameworks for digital platforms can facilitate interoperability, enabling seamless data exchange and integration across healthcare systems.<br><br>- Education and Training: Continuous education and training for patients and healthcare providers are essential to foster engagement and effective utilization of digital platforms. This is especially

PUBLICATION	TITLE	BARRIERS	FACILITATORS
		innovation and maintaining user-friendly experiences.	important in low-resource environments where technological adoption can be challenging.<br><br>- Data Validation and Verification: Rigorous validation and verification of collected data across institutions are necessary to ensure precision and accuracy, thereby enhancing the overall quality of healthcare delivery.<br><br>- Stakeholder Collaboration in Regulatory Development: Active collaboration among stakeholders, including government entities, hospitals, healthcare providers, non-profits, and patients, is crucial in developing regulatory policies that prevent barriers to innovation and optimize user experiences. This holistic approach ensures the seamless integration of all pertinent systems in healthcare.
Chomutare 2022	Artificial Intelligence Implementation in Healthcare: A Theory-Based Scoping Review of Barriers and Facilitators	Study: Lee [31]: Regulation and law<br><br>Study: Cho [49]: Evaluation and testing, Interpretability, Model interoperability<br><br>Study: Cruz [37]: Data availability<br><br>Study: Davis [45]: Evaluation and testing, Trust<br><br>Study: McCoy [32]: Disruptiveness (alert fatigue)<br><br>Study: Moon [33]: Model Interoperability, Data quality<br><br>Study: Murphree [48]: Generalizability<br><br>Study: Petitgand [46]: Data interoperability, Usability, Documentation and presentation of results, Trust<br><br>Study: Romero-Brufau [43]: Usability, Data quality, Data availability, Generalizability, Evaluation and testing<br><br>Study: Scheinker [44]: Trust, Complexity, Disruptiveness	Study: Lee [31]: Healthcare demand, Evaluation and testing, Generalizability, Data availability, Available Resources, Trialability, Motivation<br><br>Study: Betriana [47]: Healthcare demand, Planning, Education of workforce, Involvement, Evaluation and testing<br><br>Study: Cho [49]: Generalizability, Evaluation and testing<br><br>Study: Cruz [37]: Evaluation and testing, Integration, Leadership, Usability<br><br>Study: Davis [45]: Integration, Usability<br><br>Study: Gonçalves [39]: Motivation, Experiences and prior knowledge<br><br>Study: Joerin [38]: Involvement,

PUBLICATION	TITLE	BARRIERS	FACILITATORS
		Study: Schuh [35]: Data quality, Experiences and prior knowledge, Cost, Regulation and law, Data interoperability<br><br>Study: Sendak [40]: Cost, Trust, Available Resources, Generalizability, Prior evidence, Integration<br><br>Study: Xu [42]: Data availability, Integration	Evaluation and testing, Patient needs, Adaptability<br><br>Study: McCoy [32]: Healthcare demand, Communication, Feedback incorporation, Education of workforce<br><br>Study: Murphree [48]: Involvement, Communication<br><br>Study: Petitgand [46]: Involvement, Organizational policy and culture<br><br>Study: Romero-Brufau [43]: Planning, Involvement, Education of workforce, Adaptability<br><br>Study: Scheinker [44]: Prior evidence, Involvement, Planning, Evaluation and testing<br><br>Study: Sendak [40]: Involvement, Planning, External collaboration, Leadership, Integration, Interpretability, Evaluation and testing, Champions, Education of workforce<br><br>Study: Xu [42]: Education of workforce, Evaluation and testing, Innovation, Usability, Integration
Choukou 2021	Digital health technology for Indigenous older adults: A scoping review	- Acceptance by Indigenous OAs: Indigenous OAs showed varying levels of acceptance towards telehealth solutions, which could hinder implementation<br><br>- Culture: Cultural differences and considerations posed significant barriers to the successful adoption of telehealth in Indigenous communities (CMAJ Open).<br><br>- Healthcare Providers' Professional Skills: The professional skills of healthcare providers, particularly in using telehealth technologies, were reported as a barrier. This includes both the technical know-how and the adaptation to telehealth methods .	- Institutionalization of Relevant Work Practices: Developing and institutionalizing work practices that support telehealth, such as training for technologists and education for referring physicians .<br><br>- Enhanced Health Centre Capacity: Improving the capacity of health centres to handle telehealth services through careful attention to image transmission and clearly defined methods of

PUBLICATION	TITLE	BARRIERS	FACILITATORS
			communication with patients and referring providers .<br><br>- Designating Indigenous Health Organizations as Lead Agencies: Empowering Indigenous health organizations to take lead roles in telehealth initiatives and integrating telehealth into the publicly funded healthcare system .<br><br>- Conducting Research Based on Initial Benchmark Reports: Using telehealth as a tool to conduct research on health conditions prevalent in Indigenous communities, such as diabetic retinopathy and diabetic macular edema .
Chua 2023	Facilitators and barriers to implementation of telemedicine in nursing homes: A qualitative systematic review and meta-aggregation	Outer Setting: Resource Constraints: Limited access to funding and resources. Policy and Regulation: Regulatory and reimbursement challenges. Stakeholder Engagement: Lack of stakeholder engagement and support.<br><br>Inner Setting: Organizational Readiness: Low organizational readiness for change. Infrastructure: Poor infrastructure and technical support. Workflow Integration: Difficulty in integrating telehealth into existing workflows.<br><br>Individuals: Skill Levels: Limited technical skills among healthcare providers. Resistance to Change: Resistance to change from staff and patients.<br><br>Implementation Process: Training: Insufficient training and support for telehealth use. Communication: Inadequate communication among team members.	Outer Setting: Policy Support: Supportive policies and regulations. Funding: Availability of funding and financial incentives. Stakeholder Engagement: Strong stakeholder engagement and collaboration.<br><br>Inner Setting: Leadership: Effective leadership and management support. Organizational Culture: Positive organizational culture towards innovation. Technical Infrastructure: Robust technical infrastructure and support.<br><br>Individuals: Skills and Competence: High levels of technical competence among healthcare providers. Positive Attitudes: Positive attitudes towards telehealth.<br><br>Implementation Process: Training Programs: Comprehensive training programs for telehealth. Feedback Mechanisms: Effective feedback mechanisms to monitor progress.
Chueke 2023	Persisting Barriers to the	Limited Connectivity Infrastructure: In rural or remote areas, lack of reliable internet	Lessons Learned

PUBLICATION	TITLE	BARRIERS	FACILITATORS
	Adoption of Telemedicine in Latin America After the COVID-19 Pandemic	hinders telemedicine services.<br><br>Digital Divide and Unequal Access: Inequality in access to technology and knowledge affects telemedicine adoption. Limited access to devices in high-poverty areas and difficulty adapting to new technologies by the elderly and low-income individuals are significant barriers.<br><br>Regulatory Challenges: Inconsistent or vague telemedicine regulations in some Latin American countries create uncertainty, hindering adoption. An IDB study emphasizes the need for clear regulatory frameworks for EMR systems, data protection, and interoperability.<br><br>Resistance to Change: Preference for traditional, in-person care and lack of awareness or trust in telemedicine's quality pose barriers to adoption.<br><br>Data Privacy and Security: Concerns about the privacy and security of medical data deter individuals from using telemedicine platforms.<br><br>Reimbursement Issues: Health systems and insurance in some Latin American countries may not adequately cover or reimburse telemedicine services, discouraging healthcare providers from adopting telemedicine practices.	Plenty of Experiences Implemented: Documented experiences provide a foundation for sustainable, cost-effective processes. Knowledge acquisition is a collaborative effort.<br><br>Open-source Solutions: Increasing use of open-source, cloud-based solutions reduces costs and promotes interoperability. These solutions offer adaptability and community support, though implementation remains complex and costly.<br><br>Greater Cooperation and Collaboration: The pandemic emphasized teamwork and institutional cooperation, making knowledge exchange a priority supported by laws and regulations.<br><br>Communication of Medical Knowledge: Technology facilitates second opinions, remote training, and continuous medical education, enhancing institutional improvements and networking.<br><br>Patients More Active in Health Management: Patients play a crucial role in their health management, avoiding disruptions and enhancing communication and resource evaluation, fostering patient empowerment.<br><br>Formation of Interdisciplinary Teams: Permanent spaces for exchange influence decisions among health institutions, professional associations, and civil society.<br><br>Advances in Regulatory Frameworks: Telemedicine regulations vary, with only 13 Latin American countries having

PUBLICATION	TITLE	BARRIERS	FACILITATORS
			specific laws. Uruguay and Panama have notable legislation.
Clark 2021	Understanding the Needs and Values of Rehabilitation Therapists in Designing and Implementing Telehealth Solutions	Documentation Burden: Therapists face significant pressure to complete documentation during or after patient sessions, often encroaching on personal time.<br><br>Insurance Constraints: Therapists must frequently justify their services to insurance companies, which adds to their workload and affects their focus on patient care.<br><br>Productivity Pressures: Therapists are required to maintain high productivity standards, sometimes at the expense of patient care quality.<br><br>Limited Telehealth Experience: Most therapists in the study had little to no experience with telehealth systems, indicating a need for training and adaptation.<br><br>Ethical Work Challenges: The conflict between providing quality care and meeting administrative demands creates ethical dilemmas for therapists.<br><br>Time Constraints: Therapy sessions are often limited by insurance policies, reducing the time available for thorough patient care.<br><br>Systemic Challenges: Scaling technology-assisted rehabilitation in home settings faces significant challenges, including replicating therapist functionality and ensuring acceptance by patients and caregivers.<br><br>Technological Reliability: Ensuring that technology is robust and low-maintenance is crucial to avoid additional burdens on therapists.<br><br>Work-Life Balance: The documentation and administrative tasks often lead to poor work-life balance for therapists.	Patient-Therapist Relationship: Establishing trust and rapport with patients is crucial for effective rehabilitation, emphasizing the need for systems that support personal interactions.<br><br>Caregiver Involvement: Caregivers play a vital role in patient recovery, providing information and support, making them key partners in the rehabilitation process.<br><br>Technology Integration: Use of AI and data mining techniques to pre-fill documentation templates can save time and improve efficiency.<br><br>Customizable Systems: Providing therapists with easily customizable system templates and AI-generated narrative prose can help maintain focus on patient care.<br><br>Home-Based Rehabilitation: Technology-assisted rehabilitation at home can provide evidence-based customization of therapy, enhancing functional outcomes and reducing costs.<br><br>Educational Tools: Indexed and searchable videos for caregivers can assist in patient care and reduce the burden of remembering therapy instructions.<br><br>Quantitative and Qualitative Proof: Systems that document patient activities and provide regular summaries can support therapists in demonstrating the need for continued therapy to insurance companies.

PUBLICATION	TITLE	BARRIERS	FACILITATORS
			Low-Cost and Accessible Technologies: Leveraging widely available consumer products like smartphones and wearable activity trackers can decrease development costs and improve accessibility.
Cole 2019	Report on the use of telehealth in early intervention in Colorado: Strengths and challenges with telehealth as a service delivery method	Access to High-Speed Internet: Access to reliable, high-speed internet remains a significant barrier, particularly in rural areas, limiting the ability to conduct telehealth sessions effectively.<br><br>Perceptions of Telehealth's Efficacy: There are concerns among providers and families about the efficacy of telehealth compared to in-person services, which may hinder its adoption.<br><br>Technological Challenges: Issues related to the technology needed for telehealth, such as the setup of secure connections and the availability of appropriate hardware and software, present significant obstacles.<br><br>Provider Training and Familiarity: There is a noted need for more training among providers in telehealth-specific practices, particularly in engaging families through remote platforms.<br><br>Resistance to Change: Some service providers and families prefer traditional in-person visits, viewing telehealth as less personal or less effective.	Flexibility: Telehealth offers flexibility in scheduling and conducting sessions, which can be especially beneficial for families with complex schedules or those living in remote areas.<br><br>Expanded Access to Specialists: Telehealth enables families, particularly those in underserved or rural areas, to access specialists and providers they might not otherwise have available.<br><br>Support for Family Coaching Practices: Telehealth encourages active parental involvement during sessions, which aligns with best practices in family engagement and empowerment.<br><br>Potential for Increased Service Delivery: Reduced travel time for providers could potentially increase the number of families they can serve.<br><br>Continuity of Care During Adverse Conditions: Telehealth allows for the continuation of services during circumstances like bad weather or minor illnesses that would otherwise lead to cancellations of in-person visits.
Curtis 2020	Digital health technology: Factors affecting implementation in nursing homes		The thematic analysis of interview data identified three broad themes common to all<br>participant groups (managers, residents and relatives, and nurses):<br>- Improving communication<br>- Engaging with DHT and retaining

PUBLICATION	TITLE	BARRIERS	FACILITATORS
			humanised<br>care. Humanised care aims to place the patient at the centre of care and includes elements such as compassion; it is opposed to task-oriented care, which focuses on the<br>completion of tasks<br>- Introducing DHT and protecting data security ; '- Managers:<br>Improved Communication: Managers see DHT as key to better communication, utilizing digital tools for risk management and alerts.<br>Efficiency Gains: Reducing paperwork allows staff to spend more quality time with residents.<br>Enhanced Care Monitoring: Digital algorithms aid in monitoring health risks and improving care.<br>Family Involvement: Opportunities for relatives to contribute to the resident's health information digitally.<br>Data Security Management: Emphasis on the careful introduction of DHT with robust planning to ensure data security and gradual transition from paper to digital.<br><br>- Residents and Relatives:<br>Keeping Up with Technology: Positive views on adopting modern communication tools like tablets for video calls and information sharing.<br>Enhanced Communication: Faster, more accurate sharing of health updates among staff, residents, and relatives.<br>Humanized Care Concerns: Awareness of the need to balance technology use with personal interactions.<br>Data Security: Acknowledgement of risks but confidence in resolving them to support DHT use.

PUBLICATION	TITLE	BARRIERS	FACILITATORS
			- Nurses<br>Improved Work Efficiency: Nurses highlighted how DHT reduces time spent on paperwork, enhancing care quality.<br>Better Health Monitoring: Use of DHT for tracking health indicators and reducing errors.<br>Human Interaction: Ensuring DHT supports rather than replaces personal care.<br>Training and Support: Importance of adequate training and technical support from DHT providers.<br>Data Security and Management: Emphasis on secure, traceable access to digital records to maintain confidentiality.
Dahlhausen 2021	Physicians' attitudes toward prescribable mHealth apps and implications for adoption in Germany: Mixed methods study	Professionals<br>are still facing substantial barriers, such as<br>- insufficient information (1135/1295, 87.6%)<br>- reimbursement for DiGA-related medical services (716/1299, 55.1%)<br>- medical evidence (712/1298, 54.9%)<br>- legal uncertainties (680/1299, 52.3%)<br><br>- technological<br>uncertainties (658/1299, 50.7%)	- Additional information about DiGA (1104/1297, 85.1%),<br>- recommendations by medical associations (1041/1297, 80.3%),<br>- positive experience reports about DiGA from medical colleagues<br>(1024/1297, 79.0%), - opportunities to test apps (1010/1297,<br>77.9%)<br>- increased reimbursement for medical services<br>related to DiGA (932/1297, 71.9%) have the potential to support<br>health care professionals in the adoption of DiGA.<br><br>When<br>approached by patients, health care professionals also believed<br>they would be more likely to engage with the topic and,<br>thereafter, potentially prescribe DiGA (821/1297, 63.3%)
Darcel 2023	Implementing artificial intelligence in Canadian	(1) system and data readiness<br>(2) the potential for bias and inequity	Enhanced Interoperability: Developing AI tools that can effectively integrate and

PUBLICATION	TITLE	BARRIERS	FACILITATORS
	primary care: Barriers and strategies identified through a national deliberative dialogue	(3) the regulation of AI and big data, and<br>(4) the importance of people as technology enablers technological Readiness: Many clinics still use paper records and have fragmented data systems, complicating the integration of AI technologies.<br><br>Potential Bias and Inequity: Concerns that AI algorithms may perpetuate existing biases or create new ones, particularly in settings with limited resources.<br><br>Data and System Fragmentation: Lack of standardization in data entry across clinics creates challenges in implementing AI tools that require uniform data inputs.<br><br>Scepticism from Past Experiences: Previous investments in electronic health records (EHRs) did not deliver promised quality improvements, leading to hesitancy towards new technologies like AI.	operate with existing health information technologies across diverse settings.<br><br>Stakeholder Engagement: Gaining buy-in from clinic owners and healthcare providers by involving them in the development and implementation process, ensuring the tools meet their needs and fit seamlessly into existing workflows.<br><br>Regulatory Oversight and Standards: Establishing robust regulatory frameworks to ensure AI tools are safe, effective, and free from biases, enhancing trust among users.<br><br>Clear Benefits Demonstration: Clinics need to see clear, immediate benefits from AI adoption to overcome scepticism and justify investment.
DeHart 2022	Benefits and Challenges of Telehealth Use during COVID-19: Perspectives of Patients and Providers in the Rural South	Quality of Care: Both providers and patients express concerns about telehealth compromising care, especially for children, teens, and elderly patients.<br><br>Patient Comfort and Access: Connectivity and technology issues, such as limited broadband and hardware, create barriers. Patients face challenges with setup and navigation, while some prefer in-person visits.<br><br>Policy Issues: Providers face reimbursement hurdles and policy limitations regarding telehealth services.<br><br>Impersonal Nature: Telehealth is often seen as less personal, reducing engagement between patients and providers.	
Denecke 2023	Digital health as an enabler for hospital@home: A rising trend or just a vision?	Technological and Data Readiness: Many clinics still rely on paper records, making the shift to digital tools challenging.<br><br>Acceptance Issues: Concerns about the effectiveness and intrusiveness of in-home care deter acceptance from insurance, caregivers, and patients.<br><br>Privacy Concerns: Data privacy issues with mobile health applications raise patient concerns.<br><br>Patient Selection and Safety: Ensuring the right patient selection and home safety is	Interdisciplinary Collaboration: Effective communication and collaboration among all stakeholders, including family members and various health providers.<br><br>Educational Support: Adequate training for both formal caregivers and family members in care techniques and digital tools.

PUBLICATION	TITLE	BARRIERS	FACILITATORS
		crucial to minimize risks.<br><br>Economic and Training Challenges: Need for financial incentives, cost-effectiveness demonstrations, and proper training for care providers.<br><br>Infrastructure Variability: Differences in home environments versus standard hospital settings complicate care delivery.<br><br>Caregiver Availability: Increasing numbers of individuals living alone poses challenges in providing necessary care.	Technology Integration: Acceptance and integration of technology like sensors and machine learning to assist in patient monitoring and care.<br><br>Regulatory and Pathway Adjustments: Development of adapted clinical pathways and integration with existing health records systems to ensure seamless care transitions.
Depuccio 2021	Making It Work: Physicians' Perspectives on the Rapid Transition to Telemedicine		- Flexibility in the face of change<br>- Upgrading work spaces at home<br>- peer-to-peer collaboration and knowledge sharing<br>- Frequent leadership communication<br>- Rapid dissemination of telemedicine training
Desveaux 2019	Identifying and overcoming policy-level barriers to the implementation of digital health innovation: Qualitative study		Unified Definition of Innovation: Establish a system-level definition of innovation that is consistent across all stakeholders to ensure alignment and understanding. This definition should reflect the entire sector, from policy to practical solutions, to promote the generation, testing, and adoption of new technologies.<br><br>Clear Mission and Drivers: Set a clear mission to guide innovation efforts, focusing on what drives innovation such as patient experience, cost reduction, or efficiency improvements. Understanding the tension between system needs and patient benefits is crucial.<br><br>Engagement and Change Management: Innovations should involve grassroots or

PUBLICATION	TITLE	BARRIERS	FACILITATORS
			frontline approaches that include end-users like clinicians and patients early in the decision-making process. Overcoming resistance from clinicians requires effective change management strategies, emphasizing shifting from technology focus to managing organizational and cultural change.<br><br>Role Definition and Accountability: Define clear roles and responsibilities within the organizational structure to prevent fragmentation. Effective governance and accountability are essential to drive the agenda and ensure clear ownership and leadership in innovation initiatives.<br><br>Standardization and Interoperability: Develop and implement standardized processes across organizations to ensure interoperability of technologies. This includes harmonizing procurement processes and technological platforms to facilitate easier adoption and integration.<br><br>Funding and Sustainability: Evolve funding mechanisms to support the nature of innovation, ensuring that funding is not only available but also sustainable over time. Address the siloed nature of funding that can hinder comprehensive adoption and scaling of innovative solutions.<br><br>Focus on Comprehensive Strategies: Beyond just implementing new technologies or payment models, comprehensive strategies that include change management and adoption plans are necessary. These strategies should align with the overall mission and facilitate a shift in culture towards

PUBLICATION	TITLE	BARRIERS	FACILITATORS
			embracing innovation.
Dimitrova 2023	Digital health in Bulgaria: Imagination or possible reality?	Regulatory Barriers:<br>The existing regulatory framework does not adapt quickly to technological progress and lacks concrete regulations such as telemedicine frameworks and data usage guidelines.<br><br>Lack of Infrastructure:<br>Insufficient internet connectivity, especially in rural areas, and outdated systems.<br><br>Funding and Investment Constraints:<br>Budget limitations restrict the allocation of funds necessary for digital health initiatives.<br><br>Data Privacy and Security Concerns:<br>Strict data privacy and security regulations can slow the adoption of digital health technologies.<br><br>Cultural and Organizational Factors:<br>Resistance to change is significant, compounded by low levels of digital skills and data literacy.<br><br>Organizational Barriers:<br>The healthcare delivery system is fragmented, and there is a lack of standardized practices across institutions, affecting coordination and interoperability.	
Dinh 2023	Perceptions About Augmented Reality in Remote Medical Care: Interview Study of Emergency Telemedicine Providers	- AR may increase existing disparities: Providers expressed concern that AR-enabled telemedicine may worsen known disparities in wealth, resources, literacy, and so on<br><br>- Providers need clinical value and support in adoption: Providers are hesitant to adopt new technologies without evidence of benefit and support at both the institutional and infrastructural level.<br><br>- Providers anticipate consumer preferences to affect acceptability: Providers felt that public adoption and awareness will influence when they and their patients adopt AR.	
Dwivedi 2021	Potential of Internet of Medical Things (IoMT) applications in building a smart healthcare system: A systematic review	- Privacy and Security of Data:<br>Ensuring cyber safety within healthcare monitoring systems is critical as sensitive health data of patients is transferred across systems. Innovations such as lightweight block encryption methods, smartcard-based security systems, and blockchain technology are being developed to enhance privacy and security in IoMT environments.<br><br>- Data Management:	

PUBLICATION	TITLE	BARRIERS	FACILITATORS
		Managing the flow of data involves accessing, integrating, controlling, and managing information. Techniques like data anonymization, data integration, and data synchronization are crucial to ensure that only usable information is provided for applications while hiding other details.<br><br>- Scalability, Upgradation, and Regulations:<br>IoMT devices must be scalable to adapt to environmental changes and maintain efficiency with available resources. Regular updates and validation of devices to meet communication protocols and data standards are essential. However, compliance with regulations like HITECH, HIPAA, and GDPR poses challenges for rapid and widespread adoption.<br><br>- Interoperability:<br>Differences in standards and the heterogeneity of devices and data sources impede interoperability. Developing standard interfaces for cross-system operations is crucial to manage the extensive data volumes and ensure that devices can interact seamlessly within an interoperable framework.<br><br>- Cost Efficacy:<br>Financial constraints, exacerbated during times of economic stress like the COVID-19 pandemic, limit the adoption of IoMT. It is important to focus on reducing the costs of development, installation, and maintenance of IoMT systems to make them more accessible.<br><br>- Power Consumption:<br>IoMT devices, often battery-operated, face challenges with power consumption. There is a need for devices that are either self-powering or can integrate with renewable energy systems to address both operational efficiency and environmental sustainability.<br><br>- Environmental Impact:<br>The manufacturing of IoMT sensors, which often include semiconductors and potentially hazardous chemicals, has environmental implications. Efforts are needed to design and produce sensors using biodegradable materials to minimize ecological damage.	
Early 2019	Use of Mobile Health (mHealth) Technologies and Interventions Among Community Health Workers Globally: A Scoping Review	Limited funding available for mHealth development<br>• Few government policies focused on the implementation and provision of mHealth programs<br>• mHealth programs are often short-term research projects rather than a part of mainstream health care provision<br>• Few clinical trials of mHealth interventions involving CHWs<br>• Limited education of staff about the use of mHealth tools and technology	

PUBLICATION	TITLE	BARRIERS	FACILITATORS
		• Design of tailored technologies and applications can be time intensive<br>• Lack of evaluation, experimental, and longitudinal studies<br>• Most mHealth tools and apps developed without cultural relevance (norms and practices)<br>• Lack of access to, and knowledge of, mobile technologies within communities<br>• Need for improved communication between, and among, health professionals working with CHWs<br>• Programs may be developed without the ongoing involvement of the intended community	
Eigner 2019	Success factors for national eHealth strategies: A comparative analysis of the Australian and German eHealth system	Incomplete Documentation (T1)<br><br>Legal Changes (T2)<br><br>Bureaucracy Implications (T3)<br><br>User Acceptance (T4)<br><br>Data Privacy, Confidentiality, Liability, and Data Protection (T5)<br><br>Lack of Skilled Professionals (T6)T3:<br>Increase national governmental cooperation.<br>Encourage close cooperation between government and healthcare providers to shorten discussion paths and streamline processes.<br><br>T4:<br>Engage consumers in participating in voluntary eHealth services by providing comprehensible personal insights.<br>Leverage high mobile penetration and broadband coverage to implement mobile applications and IT solutions that link healthcare consumers.<br><br>T5:<br>Appoint a single institution to store, manage, and secure healthcare data in a structured and reliable way.<br>Ensure secure and stable networks and regulate data access according to different stakeholders; maintain that data authority lies with the consumer.<br><br>T6:<br>Offer training and raise transparency	T1:<br>Provide more binding regulations to participate in the nationwide network.<br>Develop easy and secure methods to share and exchange data.<br>Create structured overviews/templates for patients including treatments, medications, and personal data as a single source of truth.<br>Aggregate collected data on platforms like My Health Record to provide a structured history for each patient.<br><br>T2:<br>Introduce legal regulations for eHealth on a national level.<br>Systematically monitor issues in eHealth development to enable fast reactions for necessary changes.<br>Opportunities<br><br>Lower Adoption Barriers (O1):<br>Strategy: Engage consumers by providing comprehensible personal insights to encourage participation in voluntary eHealth services.<br><br>Increasing User Acceptance and IT Literacy (O2):<br>Strategy: Leverage high mobile penetration and broadband coverage to

PUBLICATION	TITLE	BARRIERS	FACILITATORS
			implement mobile applications and IT solutions that connect healthcare consumers and providers, enhancing user acceptance and IT literacy.<br><br>Reuse of Knowledge and Applications (O3):<br>Strategy:<br>Extend national platforms to share experiences in eHealth service development.<br>Develop open IT standards and initiate a national open-source platform for eHealth development.<br><br>Increased Scalability of eHealth Solutions (O4): Strategy:<br>Integrate currently isolated solutions into the national infrastructure.<br>Use a two-sided approach to adapt infrastructure and aggregate collected data on platforms like My Health Record to provide a structured history for each patient.<br><br>Better Information Exchange (O5):<br>Strategy: Provide easy and secure methods to share and exchange data among healthcare providers and patients.<br><br>Integrated Healthcare Data and Applications (O6):<br>Strategy:<br>Integrate healthcare information from all linked partners, including providers and patients, into a mandatory linked system to gain holistic insights over larger patient cohorts.<br>Support infrastructural development with funding projects to enhance data integration.

PUBLICATION	TITLE	BARRIERS	FACILITATORS
			Increased Data Sharing (O7):<br>Strategy:<br>Implement a secure messaging system and uphold data standards already in place to enable effective communication and coordination between patients and healthcare providers.<br><br>Cross-linking of Healthcare Providers (O8):<br>Germany (GER):<br>Provide more binding regulations to ensure participation in the nationwide network.<br>Support and monitor collaboration through government actions, acknowledging existing legal mandates and ongoing field studies.<br>Australia (AUS):<br>Foster closer collaboration with healthcare providers and consumers to quickly adapt to individual needs.<br>Modify legal regulations to be more adaptable to ensure quick responses and faster rollout of new requirements; involve stakeholders to address individual needs.
Eisapareh 2022	Facilitators and Barriers to Using mHealth from Users' Attitudes: A Qualitative Meta-synthesis	Barriers included difficulty in use, inaccessibility, uselessness or inapplicability, lack of adequate skills, communication barriers, and security concerns	facilitating<br>factors included motivational factors, documentation, degree of ease, provider credibility and source of information, perceived usability, social- cultural appropriateness, and perceived benefits
Esmaeilzadeh 2021	Patients' perceptions toward human-artificial intelligence interaction in health care: Experimental study	Communication Barriers:<br>Issue: AI applications can cause significant communication barriers between customers (patients) and service providers (healthcare professionals).<br>Specific Concerns: AI may reduce physician-patient interactions and conversations. Consumers may reject AI applications due to a need for human social interaction. Traditional physician-patient communications are fundamentally changed by AI, leading to potential losses in face-to-face cues and personal interactions.<br><br>Privacy Concerns:	Enhancing Communication:<br>Facilitator: Implement more sophisticated technologies within AI applications to improve human-computer interactions and streamline information flow.<br><br>Improving Privacy Protections:<br>Facilitator: Stronger and clearer regulations on data privacy to protect

PUBLICATION	TITLE	BARRIERS	FACILITATORS
		Issue: There are heightened privacy concerns, especially when dealing with AI under physician control.<br>Specific Concerns: People fear that anonymized data might be reidentified through AI models, increasing the risk of privacy invasion and unauthorized data breaches. There is also concern about the extensive need for data by AI systems, leading to potential misuse of health information.<br><br>Trust Issues:<br>Issue: Trust in AI clinical applications is lacking, particularly when no physician interactions are included.<br>Specific Concerns: AI's lack of transparency and the complexity of its decision-making models contribute to reduced trust among patients, who prefer more traditional, direct interactions with physicians for managing their health conditions.<br><br>Accountability and Liability Concerns:<br>Issue: There are significant concerns regarding who is held accountable for errors made by AI applications, especially in urgent care scenarios.<br>Specific Concerns: The complexity of determining liability when AI systems make errors or when their correct recommendations are dismissed. Regulatory and legal clarity is lacking, adding to the anxiety of using AI in healthcare.<br><br>Regulatory Transparency:<br>Issue: The regulatory framework governing AI applications in healthcare lacks transparency and is not well understood.<br>Specific Concerns: Patients and healthcare providers are unsure of the standards and regulations AI applications are held to, leading to hesitancy in adoption. The ongoing evolution of AI capabilities necessitates continual updates to regulatory standards, which are not keeping pace with technological advances.	against reidentification and unauthorized data usage.<br><br>Building Trust:<br>Facilitator: Increase transparency of AI decision-making processes and ensure physician oversight is incorporated into AI healthcare delivery to foster patient trust.<br><br>Addressing Accountability:<br>Facilitator: Establish clear policies and guidelines that define the roles and responsibilities of all stakeholders involved in AI healthcare, including developers, healthcare providers, and regulatory bodies.<br><br>Clarifying Regulatory Standards:<br>Facilitator: Regulatory bodies should develop and communicate clear guidelines and standards for the evaluation and use of AI in healthcare to ensure safety and efficacy. Regular audits and monitoring should be conducted to continuously assess the impact and performance of AI applications
Eze 2020	Telemedicine in the OECD: An umbrella review of clinical and cost-effectiveness, patient experience and implementation	Technological and Interface Issues:<br>Poorly designed interfaces, manual data input requirements, and delays in feedback transmission hinder effective use in diabetes management.<br>Scalability limitations and technological illiteracy contribute to operational inefficiencies and high dropout rates due to errors such as recall bias.<br><br>Community and Resource Limitations:<br>In indigenous communities, the lack of technical skills and a shortage of local staff present significant obstacles to the adoption of telediabetes solutions.<br><br>Financial Constraints:<br>High startup and ongoing costs, along with potential revenue losses, pose financial barriers to the broader implementation of telemedicine.	Targeted and Streamlined Technology:<br>Interventions should be specifically targeted and systems designed to address clinical or behavioral issues prioritized by patients.<br>Telemedicine technology should be automated, streamlined, mobile, and low-cost to reduce usage burdens and maximize clinical utility.<br><br>Tailoring to Patient Needs:<br>Home-based remote monitoring interventions should be tailored to patient characteristics and needs.

PUBLICATION	TITLE	BARRIERS	FACILITATORS
		Regulatory and Policy Deficits:<br>Inadequate or absent legislation, policies, and liability coverage are major barriers at the organizational and health professional levels.<br><br>System Incompatibility:<br>Mismatches between telemedicine technologies and existing health systems or work practices disrupt daily clinical workflows and care delivery.<br><br>Lack of Strategic Planning:<br>Poor strategic planning and insufficient engagement of key stakeholders in the development and adoption of eHealth systems impede successful implementation.	Strong relationships and effective communication between patients and healthcare professionals, along with high usability and quality of technology, are crucial.<br>Proper organization of home health care services enhances the success of telemedicine.<br><br>Involvement of Indigenous Health Workers:<br>For indigenous populations, involving indigenous health workers is vital in delivering telediabetes interventions. Their role in local language communication and helping clinicians understand and integrate into the local community is instrumental.
Ezezika 2021	The implementation of a maternal mHealth project in South Africa: Lessons for taking mHealth innovations to scale	High cost of data - 9 key informants<br><br>Unreliability of USSD - 6 key informants<br><br>Inadequate training - 5 key informants<br><br>Gaps in funding - 5 key informants<br><br>Stakeholder mismanagement - 5 key informants<br><br>Insufficient government buy-in - 4 key informants<br><br>Unequal access to information - 4 key informants<br><br>Inadequate internet connectivity - 3 key informants<br><br>Timeline pressure from stakeholders - 3 key informants<br><br>Inadequate language translation - 3 key informants<br><br>Incompatibility with health system - 2 key informants<br><br>Uncontrollable market fluctuations - 2 key informants	Easy and accessible technology - 11 key informants<br><br>Strong political will - 11 key informants<br><br>Strong stakeholder champions - 7 key informants<br><br>Effective two-way communication - 5 key informants<br><br>Cost-effective messaging system - 5 key informants<br><br>Comprehension of user needs - 4 key informants<br><br>Strong partner relations - 4 key informants<br><br>Positive user feedback - 3 key informants<br><br>Smooth integration process - 3 key informants

PUBLICATION	TITLE	BARRIERS	FACILITATORS
		Inadequate privacy features - 2 key informants<br><br>Inability to test intervention - 2 key informants<br><br>Insufficient evidence to scale - 2 key informants<br><br>Pushback from focal people - 2 key informants<br><br>Insufficient support for individuals with HIV - 1 key informant<br><br>Gap in technical leadership - 1 key informant<br><br>Formally appointed internal implementation leaders - 1 key informant<br><br>Unequal mobile access - 1 key informant<br><br>Insufficient baseline data - 1 key informant	Effective project promotion - 3 key informants<br><br>Cost-efficient technology - 2 key informants<br><br>Effective guidelines from MAMA - 2 key informants<br><br>Effective immediate scaling design - 2 key informants<br><br>High literacy rates - 2 key informants<br><br>Effective content adaptation - 1 key informant ; based on the analysis, the key components that supported the implementation of the MomConnect project included<br>(1)<br>strategic partnership and coordination across partner levels, (2) cost-effective technology and sustainable funding measures<br>(3) adequate adaptation of the innovation to local and national settings, and (4) guiding mHealth policy and legislation frameworks
Fazakarley 2023	Experiences of using artificial intelligence in healthcare: A qualitative study of UK clinician and key stakeholder perspectives	Limited Resources: There is a general scarcity of resources available to support the deployment and maintenance of AI tools within the NHS. This scarcity affects both the speed and the scope of AI implementation.<br><br>Data Protection and Regulatory Hurdles: The process of navigating data protection regulations is described as lengthy and cumbersome. Although these regulations are crucial for safeguarding patient data, they involve extensive documentation and approvals, leading to delays in setting up AI tools.<br><br>Access to Data: Accessing necessary data for AI applications in healthcare is challenging due to strict regulations. The process, despite being fairly streamlined, requires comprehensive consent protocols and thorough briefing on information governance, which is time-consuming.	

PUBLICATION	TITLE	BARRIERS	FACILITATORS
		Inconsistent IT Infrastructure: There is considerable inconsistency in IT services across different NHS Trusts, which impacts the ability to stay updated with technological advancements. Many existing IT systems are incompatible, failing to communicate or transfer information seamlessly between different departments such as radiology, cardiology, and laboratory systems.<br><br>System Compatibility Issues: The existing computer systems within the NHS are often not compatible with new AI tools, predicting future delays in AI integration and related research projects.<br><br>Capacity Constraints of IT Teams: Many NHS IT teams are overwhelmed with work, which limits their ability to undertake necessary updates and integrations of new technologies like AI. This varies significantly between Trusts, with some having better capacity than others.	
FernándezCoves 2022	Teleconsultation adoption since COVID-19: Comparison of barriers and facilitators in primary care settings in Hong Kong and the Netherlands	- Policy and legislation:<br>Restrictive legislative framework,<br>Poor/Improvable guidelines<br>Lack of awareness on specific guidelines<br><br>- Incentives:<br>Lack of financial incentives<br>Lack of non-financial incentives<br><br>- Public Awareness:<br>Patient perception of teleconsultation utility / Limited knowledge/affordability of the elderly<br><br>- Dominant Paradigm:<br>Low prevalence of teleconsultation in the area before COVID-19<br>Current low prevalence of teleconsultation in the area<br><br>- Access to care:<br>Good access to care<br><br>Stakeholder Buy-In:<br>Conflict between GPs and health insurers<br>Infrastructure	- Policy and Legislation:<br>Restrictive legislative framework,<br>Presence of guidelines,<br>Fit with national agenda,<br>Government promotion of telehealth,<br>Presences of supportive policies for ICT use<br><br>-Incentives:<br>Subsidies for equipment acquisition,<br>External legal training available,<br>Governmental training on teleconsultation uptake and use,<br>Funding for research on telehealth promotion, uptake, use, and training<br><br>Public Awareness:<br>Patient fear of COVID-19 infection,<br>Patients preferences<br><br>Dominant Paradigm:<br>Worldwide trend of teleconsultation uptake,<br>Neighbor country has extended usage of teleconsultation,<br>Current high prevalence of teleconsultation in the area

PUBLICATION	TITLE	BARRIERS	FACILITATORS
			-Access to Care:<br>Limited access to specialists<br><br>- Stakeholder Buy-In:<br>Good collaboration between GPs and health insurers,<br>Government collaboration with research centers, universities, hospitals, and public and private organizations,<br>Participation on international eHealth projects<br><br>- Infrastructure:<br>Good internet connection and digital infrastructure,<br>Plans on improving internet connection<br><br>- Economic Climate and Governmental Financing:<br>Increased funding on healthcare and health research,<br>Teleconsultation as a tool for improving sustainability
Fortuna 2020	Certified Peer Specialists' Perspective of the Barriers and Facilitators to Mobile Health Engagement	Characteristics of Individuals:<br>- Physical Barriers: Issues such as difficulty navigating and seeing the phone.<br>- Psychological Barriers: Problems like hearing voices or fears of data breaches driven by symptoms.<br>- Beliefs and Preferences:<br>Change Aversion such as preferring flip phones. Preference for tablets over smartphones.<br>- Potential for Social Isolation: Concerns that mHealth interventions might increase social isolation if not designed to promote social interaction.<br><br>Intervention<br>Characteristics<br>- Affordability: The high cost of smartphones and data plans as a deterrent to mHealth engagement.<br>- Formal Training: Recommendations for education on basic skills such as setting up an email address and using email and texts.<br>- Connectivity: Internet connectivity issues, especially in certain locations like rural areas or basements, as barriers to engagement.	

PUBLICATION	TITLE	BARRIERS	FACILITATORS
		- Peer Support (Emerging): Inclusion of certified peer specialists to foster human connection and engagement in mHealth interventions.<br>Ideas for incorporating peer support through text messages for individuals with mental and physical health issues.	
Frank 2021	Drivers and social implications of Artificial Intelligence adoption in healthcare during the COVID-19 pandemic	- Mistrust in Medical AI:<br>People who do not trust medical AI are less likely to adopt it. This mistrust can stem from concerns about the technology's accuracy, reliability, and potential biases.<br><br>- Perceived Uniqueness Neglect from Human Physicians: The perception that human physicians overlook the unique aspects of individual patients' needs can hinder medical AI adoption. This neglect might make patients feel undervalued and lead them to distrust the integration of AI in their care.<br><br>- Lack of Social Belonging: A sense of social disconnection or isolation can be a barrier to adopting medical AI. When individuals feel that they do not belong or are not part of a supportive community, they may resist new technologies, including medical AI.	- Trust in Medical AI: Trust is a crucial predictor of medical AI adoption. When people believe that medical AI is trustworthy, accurate, and beneficial, they are more likely to accept and use it.<br>- Open-mindedness:<br>Individuals with the trait of open-mindedness are more willing to embrace new technologies and innovations, including medical AI. Being open to new ideas and approaches can facilitate the adoption of AI in healthcare settings.<br>- Experience of Mistrust and Perceived Neglect by Human Physicians:<br>experiencing mistrust or feeling neglected by human physicians can increase the likelihood of adopting medical AI. When patients feel let down by human healthcare providers, they may turn to AI solutions as a potentially more reliable or unbiased alternative.
Frey 2022	Acceptance of digital health applications in non-pharmacological therapies in German statutory healthcare system: Results of an online survey	- Perception of Errors During Patient Training: Therapists unfamiliar with DiGAs are more likely to believe that errors occur during patient training with DiGAs.<br>- Uncertainty About Risks and Liability:<br>Both proponents and opponents of DiGAs express uncertainty regarding the risks and liabilities associated with using DiGAs.<br><br>- Technical Problems: belief that using DiGAs might lead to technical problems, although this did not significantly differ between proponents and opponents.<br><br>- Lack of Technical Equipment:<br>Concerns about the unavailability of required technical equipment for implementing DiGAs, though this did not show a significant difference between groups.<br><br>- Insufficient Patients' Affinity for Technology: Some therapists believe that patients' lack of affinity for technology could be a barrier, although this did not differ significantly between proponents and opponents.	

PUBLICATION	TITLE	BARRIERS	FACILITATORS
		- Knowledge of DiGAs: Therapists knowledgeable about DiGAs are significantly less likely to believe that errors occur during patient training with DiGAs.<br><br>- Positive Behavioral Intention: A majority of the sample (60.7%) indicated a positive behavioral intention towards using DiGAs, with an average score of 3.5 out of 5.<br><br>- Effort Expectancy: The effort required to implement DiGAs into daily therapy is considered feasible, with an average rating of 4.3 points.<br><br>- Performance Expectancy: The benefits derived from using DiGAs are perceived positively, with an average score of 4.1 points for performance expectancy.<br><br>- Technology Competence: High levels of technology competence among therapists, with an average score of 17.2 out of a possible 20.<br><br>- Reaching New Patients: Both proponents and opponents agree that DiGAs can reach new patients, indicating a potential facilitator for adoption.<br><br>- Higher Technology Acceptance Among Proponents:<br>Therapists with a positive intention to use DiGAs or who have DiGA knowledge show significantly higher technology acceptance compared to those who reject or are unaware of DiGAs.	
Frishammar 2023	Digital health platforms for the elderly? Key adoption and usage barriers and ways to address them	We find that the elderly perceives two key barriers to<br>initial adoption of DHPs:<br>i) negative attitudes and technology anxiety and<br>ii) one key barrier affecting both<br>adoption and usage – lack of trust- Negative Attitudes and Technology Anxiety<br>Disbelief in Potential of Digital Care: Many elderly individuals have a general scepticism towards the effectiveness of digital healthcare compared to physical healthcare, feeling uncomfortable with the idea of seeking care digitally.<br><br>- Political Concerns: Concerns about the impact of DHPs on public healthcare resources and the political debates surrounding privatization of healthcare services can deter adoption.<br><br>- Disinterest in Digitalization: Lack of interest in digital technologies in general among the elderly, stemming from satisfaction with existing physical healthcare arrangements.<br><br>- Low Digital Maturity: Many elderly people lack experience with digital technologies, making it difficult for them to navigate and use DHPs effectively.	- General Education for Potential Users:<br>Marketing Activities: Creating awareness about the existence and benefits of DHPs among the elderly through targeted marketing campaigns.<br>Educational Initiatives: Providing step-by-step instructions, testing opportunities, and personal guidance to help elderly users become comfortable with using DHPs.<br>Depoliticizing Marketing: Addressing political concerns by emphasizing the neutrality of DHPs and their benefits, independent of political debates.<br><br>- Technological Interface Design Improvements:<br>User-Friendly Design, Developing intuitive and easy-to-use interfaces tailored to the needs of elderly users with

PUBLICATION	TITLE	BARRIERS	FACILITATORS
		- Fear of Making Mistakes: Anxiety about using digital devices incorrectly or causing technical problems can prevent adoption and usage.<br><br>- Data Security Risks: Concerns about data privacy and the security of personal information when using DHPs can hinder adoption.<br>Personal Integrity Worries: Some elderly individuals worry about maintaining their anonymity and privacy when using DHPs.<br><br>- Inaccuracy of Diagnosis: Fear that digital interactions may not provide accurate diagnoses compared to physical examinations by doctors.	lower digital maturity.<br>Test Panels: Involving elderly users in the development process to ensure the platform meets their needs and preferences.<br><br>- Service Design Improvements<br>Fixed Point of Contact: Offering a consistent healthcare provider for ongoing medical interactions to build trust and ease anxiety about unfamiliar faces.<br>Omnichannel Approach: Combining online and offline healthcare services to provide a comprehensive care solution that leverages the strengths of both digital and physical interactions.<br><br>- Clear Pricing Models: Transparent Costs: Offering clear and understandable pricing models for different DHP services, which can accommodate varying levels of willingness to pay among the elderly ; suggestions for application development<br>and tailored education activities
Furlepa 2022	Recommendations for the Development of Telemedicine in Poland Based on the Analysis of Barriers and Selected Telemedicine Solutions	Financial Barriers<br>- High Costs: Belief that the costs of telemedicine are too high for implementation.<br>- Lack of Research: Insufficient research documenting the economic benefits and profitability of telemedicine applications, which deters investment.<br><br>Legal Barriers:<br>- Privacy and Confidentiality: Concerns over patient data privacy and confidentiality compared to traditional methods.<br>- Unclear Regulations: Imprecise legal acts governing virtual health services, causing data security concerns and unclear liability standards.<br><br>Awareness-Related Barriers:<br>- User Acceptance: Lack of acceptance and satisfaction from users, which is essential for effective implementation.<br>- Opposition to Non-Traditional Models: Resistance from patients and healthcare providers to adopt new models over traditional healthcare practices.	Financial Support:<br>- Subsidies and Financing: Programs providing subsidies and financing to support telemedicine adoption.<br>- Cost Reimbursement: Insurance coverage for telemedicine services, similar to traditional treatments, reducing financial burden on patients.<br><br>Technological Advancements:<br>- Broadband Expansion,<br>- Mobile Health Evolution: Support for remote patient monitoring and mobile health applications through reliable internet connections.<br><br>Awareness and Training Programs: -

PUBLICATION	TITLE	BARRIERS	FACILITATORS
		Reliability Concerns:<br>- Doubts about the reliability of remote medical examinations, especially among the elderly and those with limited access to technology.<br><br>ICT Technology Barriers:<br>- Digital Divide: Difficulty for elderly and digitally excluded individuals to use telemedicine systems without proper training.<br>- Complexity: Complexity of telemedicine systems leading to potential handling errors and incorrect diagnostic readings.<br>- Internet Access: Lack of access to the internet, particularly broadband, which is necessary for effective telemedicine.	Training: Technological training for the elderly and individuals with difficulties operating telemedicine devices.<br><br>Legal and Policy Support<br>- Authorizations and Privileges: Nationwide authorizations and privileges for healthcare professionals to practice telemedicine, simplifying the process.<br><br>Clear Guidelines:<br>- Development of clear guidelines and regulations for telemedicine services to ensure quality and security.<br><br>- Successful Implementation Examples (e.g. "Teladoc" from the US, Virtual Tumour Boards
Gajarawala 2020	Telehealth Benefits and Barriers	Technological Barriers<br>- Internet Bandwidth: Limitations in internet bandwidth, particularly in rural or underserved areas, affecting the quality of telehealth services.<br>- Technical Difficulties: Potential for technical issues during telehealth interactions, which can hinder effective communication and service delivery.<br>- Data Accuracy: Variability in internet bandwidth can affect the accuracy of data transmission, leading to potential misdiagnosis or inappropriate clinical decisions.<br><br>Legal and Regulatory Barriers<br>- Regulatory Variations: Significant variations in telehealth regulations, guidelines, and rules across states, causing confusion and hindering widespread adoption.<br>- Lack of Multistate Licensure: Providers must obtain licensure in multiple states to practice telehealth, posing a financial and administrative burden.<br>- Privacy and Security Concerns: Vulnerability to privacy and security breaches, despite encryption and compliance with standards like HIPAA.<br>- Medical Liability: Unclear standards of care and liability insurance coverage for telehealth services, increasing the risk of malpractice issues.<br><br>Reimbursement Barriers<br>- Inconsistent Reimbursement: Lack of significant reimbursement, and commercial insurance plans, discouraging providers from adopting telehealth.<br><br>Provider-Patient Relationship Barriers<br>- Impersonal Interactions: Concerns that telehealth may compromise the continuity of	Technological Facilitators<br>- Advancements in IT: Improvements in healthcare IT and telecommunications technologies that facilitate better access and quality of telehealth services.<br>- Mobile Health Applications: Use of smartphones, tablets, laptops, and other devices to provide convenient and efficient telehealth services.<br><br>Regulatory and Policy Facilitators<br>- Legislative Initiatives: Recent legislative changes, like those in the Bipartisan Budget Act, which improve Medicare telehealth policy and include coverage for remote patient monitoring.<br>- Interstate Medical Licensure Compact: Facilitates the portability of licensure and practice of telehealth across states for physicians and physician assistants.<br><br>Reimbursement Facilitators<br>- Medicaid and Medicare Coverage: Expansions in telehealth coverage by

PUBLICATION	TITLE	BARRIERS	FACILITATORS
		care and the provider-patient relationship due to the lack of face-to-face interaction.<br>- Prescribing Limitations: Restrictions on prescribing controlled substances via telehealth, as per laws like the Ryan Haight Online Pharmacy Consumer Protection Act.<br><br>Awareness and Acceptance Barriers<br>- Provider and Patient Adaptation: Resistance from both patients and providers to adapt to telehealth models due to unfamiliarity and preference for traditional face-to-face interactions.	Medicare and Medicaid, especially for remote patient monitoring and chronic condition management.<br><br>Quality and Efficiency Facilitators<br>- Cost-Effective Care: Telehealth reduces healthcare spending by minimizing unnecessary emergency department visits and prolonged hospitalizations.<br><br>- Increased Patient Satisfaction: Telehealth offers better access to care, convenience, reduced travel and wait times, and improved patient satisfaction.<br><br>Awareness and Education Facilitators<br>- Patient and Provider Training: Increased familiarity and comfort with telehealth technology through training and education.<br><br>Public Health Initiatives:<br>- Programs and initiatives that promote telehealth awareness and benefits, encouraging adoption among patients and providers.
Ganapathi 2023	Exploring the experiences and views of doctors working with Artificial Intelligence in English healthcare; a qualitative study	Lack of Awareness and Engagement<br>- Low Awareness: Limited knowledge and awareness of AI among frontline doctors.<br>- Hype and Misinformation: The hype surrounding AI leads to misconceptions, such as the belief that AI will replace doctors.<br>- Lack of Protected Time: Doctors do not have enough protected time to learn about and engage with AI technologies.<br><br>System-Level Barriers<br>- Unstructured Pathway: There is no structured pathway for doctors to enter the field of AI.<br>- Regulatory Challenges: Lack of regulatory standards and difficulties in accessing patient data.<br>- NHS IT Infrastructure: Issues with NHS interoperability and legacy IT infrastructure hinder AI implementation.<br>- Limited Capacity of Tech Companies: Small and medium-sized enterprises (SMEs) struggle to establish partnerships with health providers due to limited capacity to	Education and Training<br>- Medical Curriculum Integration: Incorporating AI and digital health into the medical undergraduate curriculum.<br>- Flexible Opportunities: Providing flexible opportunities for doctors to gain experience in AI, such as internships, electives, and fellowships.<br><br>Organizational and System Support<br>- Protected Time: Allocating protected time for doctors to engage with AI technologies and related educational resources.<br>- Partnerships with Industry: Forming partnerships between the NHS and

PUBLICATION	TITLE	BARRIERS	FACILITATORS
		provide evidence.<br><br>Cultural and Professional Barriers<br>- Risk Aversion and Resistance to Change: Doctors are often risk-averse and resistant to change, preferring established methods over new technologies.<br>- Lack of Collaboration: Doctors working in AI face challenges in collaboration, with AI professionals often working in silos and being reluctant to share commercially valuable information.<br>- Professional Tensions: Differences between the technological mindset of "move fast and break things" and the medical mindset of "going slow and safe" create friction.<br>- Perceived Threats to Clinical Autonomy: Fears that AI might compromise their clinical autonomy and professional judgment.<br><br>Job Stability and Work-Life Balance<br>- Less Job Stability: Moving to AI roles, especially in startups, involves less job stability compared to NHS roles.<br>- Increased Workload: Transitioning to AI roles can involve increased workload and the need for greater comfort with uncertainty.<br><br>Ethical and Medico-Legal Issues:<br>- Unclear Responsibilities: Uncertainty about whose responsibility it is to innovate within the NHS.<br>- Ethical Concerns: Ethical and medico-legal concerns regarding the use of AI in healthcare.	industry to provide opportunities for doctors to work with AI.<br><br>Cultural and Professional Development<br>- Promoting a Collaborative Culture: Fostering a culture of openness and collaboration where clinical values are prioritized.<br>- Building Trust: Encouraging trust-building activities between doctors and tech professionals, recognizing and respecting different expertise.<br>- Clinical Involvement: Ensuring doctors are involved in the development and deployment of AI solutions to ensure clinical relevance and safety.<br><br>Job Satisfaction and Career Opportunities<br>- Improved Work-Life Balance: AI roles can offer better work-life balance and greater autonomy compared to traditional clinical roles.<br>- Retaining Talent: Providing flexible career options to retain NHS talent and prevent doctors from leaving for full-time industry roles.<br><br>Policy and Regulatory Support<br>- Clear Guidelines: Establishing clear guidelines for the development and use of AI in healthcare.<br>- Regulatory Standards: Developing regulatory standards to ensure safe and effective use of AI technologies.
Giebel 2023	Problems and Barriers Related to the Use of Digital Health Applications: Scoping Review	Use and Adherence (n=22)<br>Usability (n=19)<br>Technology (n=18)<br>Validity (n=16)<br>Patient-Physician Relationship (n=13)<br>Data Privacy and Security (n=12)<br>Implementation (n=11)<br>Knowledge and Skills (n=10)	

PUBLICATION	TITLE	BARRIERS	FACILITATORS
		Individuality (n=9)<br>Costs (n=8)	
Giesbrecht 2023	Telerehabilitation Delivery in Canada and the Netherlands: Results of a Survey Study	- Technology Issues (therapist or patient) - 89% (463/520)<br>- Lack of Physical Touch Required to Deliver Services - 62.3% (324/520)<br>- Poor Technology Self-Efficacy - 37.3% (194/520)<br>- Safety Concerns - 27.5% (143/520)<br>- Privacy - 22.3% (116/520)<br>- Lack of Appropriate Training Opportunities for Therapists - 22.1% (115/520)<br>- Patients with Acute Conditions - 19% (99/520)<br>- Online Platforms Not Designed for Telerehabilitation - 18.7% (97/520)<br>- Poor Fit within Workflow as Therapist - 18.1% (94/520)<br>- Lack of Reimbursement by Insurer for Appropriate Technology - 13.5% (70/520)<br>- Regulatory Body Policies - 8.1% (42/520)<br>- Inability to Consult/Collaborate with Other Professionals - 5.9% (31/520)<br>- I Don't Know - 1.3% (7/520)<br>- None - 0.2% (1/520)<br>- Other - 19.4% (101/520)	- Patients' Electronic Resources (e.g., access to internet, devices) - 85% (442/520)<br>- Good Technology Self-Efficacy - 75.9% (395/520)<br>- Technology Setup Support - 61.3% (319/520)<br>- Educational Material about the Issue or Condition - 36.9% (192/520)<br>- Use of Online Written Information, or Booklets - 35.8% (186/520)<br>- Good Fit within Workflow - 35.2% (183/520)<br>- Apps for a Smart Phone or Tablet - 32.1% (167/520)<br>- Videos - 28.1% (146/520)<br>- Patient Must Have a Chronic Condition - 2.7% (14/520)<br>- I Don't Know - 1.9% (10/520)<br>- Other - 15.6% (81/520)<br>- having an<br>appropriate physical space (17/81, 21%) and - access to<br>appropriate technology for both provider and patient (10/81,<br>12%).
Gray 2022	Artificial Intelligence Education for the Health Workforce: Expert Survey of Approaches and Needs	Lack of Governance Structures and Processes<br>- Ambiguity about roles of workforce organizations and government departments.<br>- Lack of clarity around processes for investment in AI education.<br><br>Resource Constraints<br>- Human Resource Challenges: Lack of a dedicated workforce.<br>Limitations in expertise in technology within healthcare.<br><br>Time Issues:<br>Competing priorities.<br>Over-burdened curriculum.<br><br>Funding Constraints:<br>Limited university resources. Poor external support from government.	Mobilization of Expertise<br>- Accessing academic expertise on AI and its applications.<br>- Establishing links with and participating in networks of experts.<br>- Involvement in consultative forums.<br><br>Influential Leadership<br>- Dynamic thinkers in senior roles within organizations.<br>- Instrumental healthcare and academic stakeholders advocating for AI education.<br><br>Leveraging Collaborations

PUBLICATION	TITLE	BARRIERS	FACILITATORS
		Insufficient funding for meaningful research and ongoing education.<br>Inadequate funding to access and deploy technological solutions.<br><br>Cultural Unreadiness<br>- Bridging the gap between early and late adopters.<br>- Resistance to new technologies and lack of knowledge.<br>- Complexity of implementing AI in an already crowded curriculum.<br>- Compartmentalization of the educational program.	- Cross-sector collaborations.<br>- Support from medical colleges for AI education.<br>- Investments in AI hubs.<br>- Expanding Continuing Professional Development<br><br>Continuing professional development initiatives to upskill the workforce.<br>Targeting both clinical and supervisory roles.<br>Higher Education Planning and Programming<br><br>Using university resources and investing in academic teaching.<br>Integrating AI applications in instructional delivery.<br>Comprehensive course reviews to implement AI education.<br>Government Drivers<br><br>Policy development, endorsement, and support from government.<br>Desire from health departments as policy makers, funders, and implementers.<br>Health Service Improvements<br><br>Advocating for infrastructure and quality improvements.<br>Enhancing patient experiences and outcomes.<br>Increasing productivity and economic benefits.
HallDykgraaf 2021	“A decade's worth of work in a matter of days”: The journey to telehealth for the whole population in Australia	Variation in Effectiveness: Advantages of telehealth are not universal and vary with disease severity and relative need.<br><br>Rapid Implementation Issues: Speedy telehealth implementation in response to COVID-19 may inadequately address unforeseen problems or challenges.<br><br>Practical and Operational Issues: Technical reliability, poor interoperability with appointment and clinical systems, and lack of access to stable telecommunications	

PUBLICATION	TITLE	BARRIERS	FACILITATORS
		infrastructure.<br><br>Digital Proficiency and Technology Access: Limited digital proficiency, internet affordability, lack of access to suitable technology, and lack of appropriate personal spaces for patients to receive calls.<br><br>Provider and Patient-End Barriers: Lack of necessary equipment, limits in expertise and capability, and technical disruptions.<br><br>Sustaining Telehealth Adoption: Need for normalization of implementation behaviors to ensure future capability and readiness.<br><br>Clinical Process Re-engineering: Re-engineering of clinical processes, development of novel components outside currently established health service delivery models, and examination of optimal roles for telehealth modalities in balanced portfolios of healthcare delivery modes.<br><br>Integrated Electronic Solutions: Development of integrated electronic solutions for clinical support services (e.g., e-prescribing, ordering pathology, and imaging investigations).<br><br>Role of Consumers and Clinicians: Defining applications, embedding workflows, clarifying information provision, and developing communities of practice.	
Haneef 2020	Innovative use of data sources: A cross-sectional study of data linkage and artificial intelligence practices across European countries	1. Legal: Complex laws and data protection regulations block linkage between different data sources with a deterministic approach.<br><br>2. Technical: Lack of human resources and capacities/skills within national institutes of public health and health information statistics.<br><br>3. Data Governance: Lack of governance of health information.<br><br>4. Organizational and Structural: Limited resources to support the health information infrastructure.	A. Legal Aspects:<br>Encouraging more flexible data governance frameworks to support data linkage of different data sources.<br>Ensuring specific mandates for data availability/access/capture and safe storage as part of a national/regional health information system.<br>Harmonizing differences in the implementation and interpretation of the EU-GDPR and additional national regulations across EU member states.<br>B. Technical Aspects:<br>4. Encouraging more collaborations and partnerships to build capacities for using new health information-related technologies, sharing new methods, skills, experiences, and data for comparative research studies among EU national

PUBLICATION	TITLE	BARRIERS	FACILITATORS
			institutes of public health, health information, and statistics.<br><br>C. Data Governance:<br>5. Strengthening national health information infrastructure through various initiatives.<br><br>D. Organizational and Structural Aspects:<br>6. Ministries of health and research from European countries providing support (financial and political) for the development of integrated national health data hubs/data platforms to strengthen national health information infrastructure.
Harst 2020	Identifying barriers in telemedicine-supported integrated care research: scoping reviews and qualitative content analysis	Technical Barriers<br>- Inadequate equipment and ICT infrastructure.<br>- Lack of interoperability and standardization.<br>- Poor integration with existing systems.<br>- High technical complexity and insufficient user involvement.<br><br>Behavioural Barriers<br>- Resistance to change and lack of user acceptance.<br>- Concerns about data security and privacy.<br>- Limited usability and practicality of telemedicine solutions.<br>- Lack of personalized features and educational tools.<br><br>Economical Barriers<br>- Lack of funding and financial resources.<br>- High costs of technology implementation and maintenance.<br>- Insufficient reimbursement and financial incentives.<br><br>Organizational Barriers<br>- Insufficient data for evaluation and quality management.<br>- Lack of holistic understanding and acceptance of integrated care.<br>- Inadequate involvement of professionals and regional disparities.<br>- Poorly designed care pathways and organizational structures.	
Hawley 2023	Connecting the disconnected: Leveraging an in-home team member for video visits for older adults.	- Participant's own device unexpectedly unable to be used for visit; used in-home team member's VA device (3, 15%)<br>- Uncomfortable using technology (9, 45%)<br>- Participant's usual technology support (e.g., family member) unavailable (7, 35%)<br>- Poor connection during visit (5, 25%)	- Participant owns a device (13, 65%)<br>- Comfortable using technology (11, 55%)<br>- Pharmacist attempted to troubleshoot (6, 30%)

PUBLICATION	TITLE	BARRIERS	FACILITATORS
			- Technology help from in-home team member allowed for successful connection (10, 50%)
Heeres 2023	Drivers and Barriers to Implementing the Internet of Things in the Health Care Supply Chain: Mixed Methods Multicase Study.	Barriers (ranked by order of importance)<br><br>1. Implementation costs are too high<br>2. Lack of knowledge and skills<br>3. Lack of organization-wide coordination<br>4. Difficult to integrate into existing processes<br>5. Technology immaturity<br>6. Trouble building a valid business case<br>7. Resistance to change<br>8. Security constraints<br>9. Afraid innovation will fail and cause problems<br>10. Lack of cooperation with suppliers<br>11. Lack of urgency to innovate supply chain processes	Facilitators (Drivers) (ranked by order of importance)<br><br>1. Creation of valuable data<br>2. Operational efficiency<br>3. Improved inventory management<br>4. Increased traceability<br>5. Increased transparency<br>6. Cost savings<br>7. Operations automation<br>8. Improved quality of care<br>9. Higher employee productivity<br>10. Increased job satisfaction<br>11. Collaboration with suppliers<br>12. Reduce health care expenses<br>13. Higher agility<br>14. Regulatory requirements<br>15. Shift in focus
Hobeck 2021	Overcoming diffusion barriers of digital health innovations: Conception of an assessment method	Digital health innovations (DHIs), especially cross-sectoral and intersectoral reimbursements, is a major barrier. High initial costs and infrastructure costs also contribute to financial difficulties in implementing these technologies.<br><br>Regulations and Guidelines: Complex regulatory frameworks, including strict data protection laws and medical product certifications, can delay or hinder the implementation of digital health solutions.<br><br>Technical Barriers: Limited technical interoperability and the lack of communication standards are major obstacles. Infrastructural issues and concerns regarding the reliability and security of medical data further compound these challenges.<br><br>Proof of Medical Effectiveness: There is a lack of clinical trials and certifications to confirm the medical effectiveness of DHIs, which is necessary to gain acceptance in healthcare systems.<br><br>User Acceptance: Resistance from healthcare providers, including concerns about changing established practices and low technical literacy, contributes to user adoption challenges. The conservative attitude of some physicians, coupled with fears of job loss, exacerbates these barriers.	Regulatory Support: The introduction of the Digital Healthcare Act (DVG) in Germany, which supports the inclusion of digital health applications alongside traditional medications, acts as a facilitator.<br><br>Governmental and Institutional Support: Support from health insurance companies and governmental initiatives, such as funding and incentives, encourages the development and adoption of DHIs.<br><br>User Awareness and Knowledge: Increased efforts in educating healthcare professionals and patients on the benefits and functionalities of DHIs could act as a facilitator.
Horwood 2023	Challenges of using e-health	- Lack of Computer Skills: Minimal computer experience and skills among nurses	

PUBLICATION	TITLE	BARRIERS	FACILITATORS
	technologies to support clinical care in rural Africa: a longitudinal mixed methods study exploring primary health care nurses' experiences of using an electronic clinical decision support system (CDSS) in South Africa.	before starting eIMCI training, leading to longer consultations and frequent technical challenges.<br><br>- Technical Challenges: Issues such as difficulty logging into eIMCI, inability to find previously entered patient information, and frequent technical disruptions.<br><br>- Poor Integration into Consultation: Nurses not using eIMCI as intended, mandatory questions causing delays, and incomplete printouts undermining confidence.<br><br>- Lack of Health System Support: Staff shortages, frequent staff rotations, and poor integration with other clinic programs, leading to increased administrative work and reliance on pIMCI.	
Hosseini 2024	Challenges and solutions for implementing telemedicine in Iran from health policymakers' perspective	Regulatory Challenges: The absence of specific telemedicine laws and legal frameworks was identified as a major barrier, making it difficult for policymakers to govern and regulate telemedicine practices.<br><br>Infrastructure Limitations: Limited internet access, especially in rural areas, and inadequate technological infrastructure were significant obstacles to telemedicine implementation.<br><br>Financial Constraints: Insufficient budget allocation for telemedicine programs and the high cost of telemedicine equipment made it challenging to expand these services.<br><br>Resistance to Change: Healthcare providers and physicians exhibited resistance to adopting telemedicine, primarily due to concerns over the quality of care, lack of familiarity with telemedicine technologies, and fear of job insecurity.<br><br>Data Privacy Concerns: Issues regarding the protection of patient data and the potential for security breaches hindered the full adoption of telemedicine.	Government Support: The Iranian government's growing interest in expanding telemedicine services, along with initiatives to integrate telemedicine into national health policies, was a key facilitator.<br><br>Technological Advancements: The increasing availability of digital platforms and improvements in communication technologies helped facilitate the adoption of telemedicine.<br><br>Cost Efficiency: Telemedicine was recognized as a cost-effective method for providing healthcare services, especially in remote and underserved areas, leading to positive support from policymakers.<br><br>Pandemic-Driven Adoption: The COVID-19 pandemic accelerated the use of telemedicine as an essential tool for delivering healthcare services when in-person visits were not possible, driving further interest in expanding telemedicine capabilities.<br><br>Educational Campaigns: Efforts to educate healthcare providers and patients about the benefits of telemedicine helped

PUBLICATION	TITLE	BARRIERS	FACILITATORS
			reduce resistance and promote its adoption.
Hulter 2020	Adopting patient portals in hospitals: Qualitative study	Perceived threat: A fear of contracting COVID-19 motivated individuals to adopt telehealth. However, concerns over the lack of personal connection with healthcare providers, potential misdiagnoses, and limited internet access, particularly among the elderly, remain significant barriers .<br><br>Self-efficacy: While self-efficacy was generally associated with telehealth adoption, the data revealed that individuals with lower self-confidence in their ability to use digital technologies found it harder to engage in telehealth services .<br><br>User Awareness and Experience: Lack of awareness of telehealth, concerns about data privacy, and difficulty in adjusting to new technology remain key challenges. Healthcare providers, especially in high-pressure environments, may feel overwhelmed by telehealth demands .	Perceived Value: Functional value (e.g., time savings, risk reduction, efficiency) and social value (the ability to maintain contact with professionals) facilitated a positive adoption of telehealth .<br><br>Cues to Action: Recommendations and encouragement from friends, family, and healthcare providers positively influenced the perceived value and intention to adopt telehealth .<br><br>Perceived Relatedness: The ability to maintain a close and communicative relationship with professionals via digital health technologies enhanced the perceived value and emotional response, further facilitating adoption .
Ilali 2023	Telemedicine in the primary care of older adults: a systematic mixed studies review	- Comfort with Patient Communication: Many older adults expressed concerns about their needs being assessed over the phone, doubting the ability of doctors to make accurate diagnoses in these circumstances. (5 articles)<br><br>- Technology Skills and Knowledge:<br>Older people believed they were not able to accomplish certain technological tasks but discovered they could do so after a small suggestion on how to proceed. (7 articles)<br><br>- User Habits/Preferences:<br>Older adults preferred in-person consultations and did not feel that telemedicine felt real to them. (6 articles)<br><br>- Technology Equipment:<br>Lack of access to necessary telecommunications devices for telemedicine consultations. (6 articles)<br><br>- Medical Cost (Out-of-Pocket):<br>Reliance on paid caregivers often meant using the aides' own phone and data plan, raising questions about reimbursement and accessibility. (2 articles)<br><br>- Patient Awareness/Support:	Location/Travel Time: E-consultations break down geographical barriers, potentially reducing the time and effort needed to access healthcare. (7 articles)<br><br>- Comfort with Provider Interaction Opportunities for doctor-nurse communication often extended beyond individual cases. (2 articles)<br><br>- Technology Skills and Knowledge:<br>Older adults discovered they could perform technological tasks with small suggestions, indicating potential for increased self-efficacy with support. (2 articles

PUBLICATION	TITLE	BARRIERS	FACILITATORS
		Difficulty with internet access and the need for help from family members to use telemedicine services. (1 article)<br>)	
Jacob 2020	Social, organizational, and technological factors impacting clinicians' adoption of mobile health tools: Systematic literature review	Social Factors:<br>- Resistance to change: Clinicians who are resistant to change may be reluctant to adopt mobile health (mHealth) tools.<br>- Lack of familiarity with technology: Clinicians unfamiliar with mobile technologies may resist using mHealth tools.<br>- Professional image: Using mobile phones at work may be seen as unprofessional by some clinicians.<br>- Privacy concerns: Concerns about privacy and data security may hinder adoption.<br><br>Organizational Factors:<br>- Lack of organizational support: Lack of support from the organization can slow down adoption.<br>Insufficient training: Nonexistent, inadequate, or insufficient training programs can be significant barriers.<br>- Increased workload: mHealth tools can be perceived as increasing workload due to issues like double data entry and adjustment to new responsibilities.<br>- Poor integration with workflow: Lack of proper integration with clinical workflows can hinder adoption.<br><br>Technological Factors:<br>- Technical issues: Common technical difficulties, such as system errors, poor output quality, and connectivity issues, can hinder adoption.<br>- Lack of technical support: Inadequate technical support, especially during off-hours, can create difficulties.<br>- System reliability: Concerns about system failures and malfunctions may deter clinicians from using mHealth tools.<br>- Poor interoperability: Issues with integrating mHealth tools with existing systems can be a significant barrier.	
James 2021	Spread, Scale-up, and Sustainability of Video Consulting in Health Care: Systematic Review and Synthesis Guided by the NASSS Framework	Barriers (Challenges)<br><br>Lack of Technical Telehealth-Specific Support for Clinical Staff (6 articles)<br><br>Need for Redevelopment of Workflows and Organizational Routines (6 articles)<br><br>Financial Pressures (5 articles)<br><br>Lack of Training (5 articles)	Facilitators (Opportunities)<br>Availability of Clinical and/or Nonclinical Telehealth Champions or Coordinators (8 articles)<br>Provider Acceptance (4 articles)<br>Absence of Billing or Licensure Restrictions (3 articles)<br>Adequate Funding (4 articles)<br>Strong Interorganizational Communications (4 articles)
Javaid 2022	Internet of Things in the	Privacy Concerns	

PUBLICATION	TITLE	BARRIERS	FACILITATORS
	global healthcare sector: Significance, applications, and barriers	Ensuring the complete privacy of individuals' health data remains challenging despite ongoing efforts.<br>Lack of Training<br><br>Insufficient training for healthcare workers, with reports indicating only 1 in 4 workers receiving training in cybersecurity and digital tools.<br><br>Inadequate Infrastructure<br>Lack of adequate infrastructure to support IoT implementation.<br>The need for regular updates to infrastructure, as older systems pose security risks.<br>Budget constraints for introducing or updating existing infrastructure.<br><br>Cybersecurity Risks<br>Healthcare organizations are frequently targeted in cybersecurity battles, and older devices connected to outdated systems are particularly vulnerable.<br><br>Availability of Devices<br>Challenges in ensuring the availability of necessary devices for IoT implementations, particularly in specialized areas like heart health care.	
Johnston 2022	Foundation Level Barriers to the Widespread Adoption of Digital Solutions by Care Homes: Insights from Three Scottish Studies.	- Connectivity of Care Homes:<br>Poor internet connectivity in care homes. Reliable internet is essential for digital solutions, yet many care homes in SE Scotland suffer from subpar connectivity.<br><br>- Capabilities for Digital Data Collection:<br>Limited use of digital data collection systems. Most care homes still rely on paper-based systems, with only 35% using electronic care management and 43% using electronic medication management. The main reasons include the high cost of digital systems and timing issues. Additionally, there is a lack of standardization in data formats and assessment tools, leading to non-comparable data and limited data linking and interoperability.<br><br>- Access to Data to Inform and Drive Digital Solutions:<br>Complicated governance and ethical frameworks for data access. The governance of care home data is fragmented, involving multiple bodies and processes, making it difficult to navigate. Unlike NHS data, social care data governance is less developed, hindering research and innovation due to the lack of a streamlined process for obtaining permissions and accessing data.<br><br>- The Need for Trust in the Use of Care Home Data:<br>Lack of trust and confidence in data management systems. Public and professional trust is crucial for data system success. Concerns over transparency and data sharing advantages exist, particularly among private sector providers worried about	

PUBLICATION	TITLE	BARRIERS	FACILITATORS
		commercial sensitivity. Regional data safe havens and trusted third-party intermediaries can help build trust by securely managing and sharing data.<br><br>- The Need for Certainty and Coordination: Lack of coordination among stakeholders. Multiple stakeholders are working towards digital transformation in health and social care. However, without tight coordination, efforts may be duplicated or incompatible, hindering progress. Effective communication and coordinated efforts are needed to avoid these issues and ensure successful implementation of digital solutions.	
Jonasdottir 2022	Health professionals? Perspective towards challenges and opportunities of telehealth service provision: A scoping review	The findings of the review led to three categories: (1) study demographics, (2) challenges for telehealth,<br>and (3) opportunities for telehealth. The most frequently reported challenges were issues related to communications, inadequate technology, or support, and need for training and knowledge to use the technology. Issues related to communication (n = 11): Miscommunication; Impersonal communication; Need for seeing the patient; Hard to build rapport; Written communication may not be read; Not always possible to see the client; Disturbance to personal life; Messages during non-working hours; Ineffective communications; Lack of presence; Hard to develop therapeutic rapport; Indirect communications.<br><br>Technology and support (n = 11): Concerns over accuracy and reliability of technology; Barriers related to hardware, software, or networking; Inadequate technological infrastructure and support; Technological problems; Need for immediate technological support.<br><br>Technological knowledge and training (n = 10): Technology literacy barriers; Knowledge and experience in using technological tools and applications; Need for technological training for both providers and clients; Training needed when technology issues.<br><br>Security barriers (n = 8): Privacy and trust in using the technology; Concerns over perceived risk associated with not being able to assist the client if needed; Perceived risk regarding others being able to see sensitive information on the platform; Violation of personal data protection; Internet fraud; Concerns regarding confidentiality; Decreases trust; Clients concerns for security and privacy; Concerns over liability and licensing; Need to deal with complex ethical issues; Concerns over external interference during sessions.<br><br>Limitations to service provision (n = 8): Limited to "simpler" issues; Not suitable for all clients; Loss of personalized care; Limitations to online services in physiotherapy; Works better as a follow-up; Reduces quality of therapeutic work; Concerns regarding improvement in clients' outcomes or clinical efficiency; Client might feel online service is not real service.	The most<br>frequent categories of opportunities related to improved access to services, benefits related to sharing of information sharing and experience and training of using technology ; Accessibility (n = 11): Better access for people with physical health issues; Flexibility for both clients and therapist regarding location; Useful when clients can otherwise not access therapy; No need for traveling; No worries about weather; Clients can receive service without leaving their home; Potential to expand services and provide better access to services in rural locations; Useful when physical access to healthcare is limited; Enables remote monitoring of clients and their environment; Enables multiple participants to access the appointment; Less commute.<br><br>Information sharing (n = 11): HCP can facilitate data exchange; Possible to send photos; Some people like to send information through an app; Provides timely access to information; Enhances opportunities to initiate contact after discharge; Possibility to look back at written information; Facilitates integrated care and co-operation between healthcare professionals; Gives clients or family members possibility to inform about emergency and for HCP to respond to it;

PUBLICATION	TITLE	BARRIERS	FACILITATORS
		Limitations to assessment (n = 8): Lack of face-to-face feedback as otherwise some information might get lost such as regarding body language, emotions, scent, etc.; HCPs don't get all the clues they would get if they were going to the client's home; Practitioners don't know if clients read written information.<br><br>Healthcare providers' stance (n = 8): Barriers related to attitudes, willingness, and beliefs of HCP to use this form of service; HCP consider it a lesser form of therapy; HCP concerns over quality of service; HCP hesitate to use new technology; Fear of losing face-to-face contact with clients; HCP expectations about client's skills or choices.<br><br>Administrative barriers (n = 5): Barriers related to organizational and management policies; Jurisdictional and organizational boundaries; Lack of legal guidelines or supporting policies; Lack of organizational support, incentives, and operating procedures.<br><br>Workload (n = 5): Using online services might increase workload; Difficulties fitting the online service into HCP already busy schedule; Hassle and time commitment for HCPs to adapt; Large patient load.<br><br>Financial barriers in the workplace (n = 5): Budgeting barriers or limited funding; Expensive technology; Cost vs. reimbursement (verifiable return on investment); Gap between private and public sectors.<br><br>Clients' context (n = 4): Some clients can't afford internet connection or technology; Limited access to internet services; Poverty.<br><br>Clients' stance (n = 4): Clients' disengagement; Clients' expectations to service; Clients not showing up or forget appointments.	Promotes the HCP-client relationship and communication; Can also facilitate information sharing between HCP; Enables real time communication with clients; Enables clients self-reporting of outcome data; Relatives can join the appointment; Written communication gives both HCP and clients the chance to reflect on their answer before they send it.<br><br>Experience, training, and attitude (n = 8): Technology knowledge, training, and experience with technology; Positive attitudes toward online work; Perceived controllability over the technology; HCP with experience of providing online services are more comfortable providing such service.<br><br>Technology and support (n = 6): Technology support is important; Good audio and visual clarity of the digital system encourages greater uptake; Access to good reliable technology and programs important; Regular technology meetings to get questions answered are helpful; Basic and user-friendly technology; Helpful if users recognize the technology used; Need for having technology where privacy and confidentiality is ensured.<br><br>Beneficial outcomes for clients (n = 6): Has proven clients' gains; Emotional support; Supports clients functioning at home and in the community; Supports clients to adhere to prescribed exercises/activities; Supports clinical needs of clients; Alleviates treatment barriers such as fears of stigma; Online services can facilitate improvement in the quality of care; Patient satisfaction; Enables monitoring; Client-centered care.

PUBLICATION	TITLE	BARRIERS	FACILITATORS
			Efficiency (n = 6): Possible to reach more clients through phone; Reduces workload; Potential to improve efficiency in emergency situations; Saves time because of less commute, less phone calls to relatives if they join the appointment, and short appointments.<br><br>Cost effectiveness (n = 5): Reduces cost because of no travel and possibility of serving more clients.<br><br>Structure (n = 4): Important to have visual contact with client; Regular meeting with tech support; Closed sessions that have boundaries; Rigorous screening of clients as it does not fit everyone; Tiered approach to implementation; Important to define structure.<br><br>Administrative support (n = 1): Need for sufficient number of staff; Policies and protocol; "Netiquette" policies
Jonnagaddala 2021	From telehealth to virtual primary care in Australia? A Rapid scoping review	Limited access to affordable technology: Inadequate access to necessary telehealth technology for both patients and providers.<br><br>Lack of long-term policies for funding telehealth: Absence of sustainable financial models to support the continuous use of telehealth services.<br><br>Safety and privacy concerns: Issues related to patient data protection and security during telehealth sessions.<br><br>Electronic Medical Records (EMRs) limitations: EMRs may not fully capture clinical thinking and patient experiences, potentially leading to acontextual clinical management and ethical concerns.<br><br>Need for plain language explanations and ethical oversight: Essential for ensuring clear communication and ethical governance in telehealth services.<br><br>Increased workload and reduced income for GP practices: Rapid administrative requirements and staffing adjustments required for telehealth, leading to higher	Workforce preparation: Adequate training and development of healthcare professionals for safe and appropriate usage of telehealth technologies.<br><br>Good communication among care providers: Enhanced by networking, co-location, and consolidation.<br><br>Professional bodies' guidelines: Guidelines provided by professional organizations to ensure best practices in telehealth.<br><br>Virtual simulations for training: Use of virtual simulations to develop key telehealth competencies.

PUBLICATION	TITLE	BARRIERS	FACILITATORS
		workloads and lower revenue.<br><br>Delayed prevention, screening, referral, and diagnosis of cancer: Shifting to virtual care has caused delays in critical cancer-related services.<br><br>Challenges in managing lifestyle-related cancer risk factors: Difficulties in addressing lifestyle-related factors contributing to cancer risk through virtual care.	Trained staff and validated apps: Essential for effective virtual medication reviews and other telehealth services.<br><br>Patient privacy protocols and relevant technology: Ensuring privacy and using technology suited to local contexts.<br><br>User-centric approaches: Approaches like 'co-presence-enhanced design' to reduce patient anxiety and increase confidence in managing chronic diseases.<br><br>Potential for shifting more types of care to primary care: Using telehealth to transfer care (e.g., cancer survivorship) to primary care, freeing up hospital staff.<br><br>Digital resources in remote and rural settings: Highlighting the importance of digital resources in primary care, especially in remote and rural areas.<br><br>Business model realignment: Networking, co-location, consolidation, and new funding arrangements to manage resources more efficiently and ensure safety and quality of care.
Kaihlanen 2022	Towards digital health equity - a qualitative study of the challenges experienced by vulnerable groups in using digital health services in the COVID-19 era	Access to digital resources: For most participants, access to digital health services was hampered by insufficient digital skills, language skills, or both. Additionally, a lack of support and training, poor health, and the lack of strong e-identification or suitable devices required for digital services prevented access. Regardless of age, participants felt that using digital health services required significantly higher-level digital skills compared to the skills required to use everyday digital devices and applications.<br><br>Digital skills and devices for older adults: Especially for many older participants, poor basic computer skills and the lack of devices were considerable barriers to accessing digital health services. However, there were large differences in skills, with some older participants able to use computers and smart devices fluently. Older participants felt they did not receive enough guidance on how to take advantage of digital health services. The cancellation or conversion of voluntary support and	

PUBLICATION	TITLE	BARRIERS	FACILITATORS
		computer assistance due to the COVID-19 pandemic further exacerbated this issue.<br><br>High users and mental health service users: Some high users found learning digital skills to access digital health services too demanding and time-consuming. They expressed concerns about falling out of a digitalizing society due to their current life situations not allowing for learning. High users and mental health service users also found certain health services challenging to access due to usability or language problems.<br><br>Language barriers for migrants: Inadequate local language skills were a major barrier for migrant participants in using digital health services. Even booking appointments remotely using the Finnish language proved difficult. Obtaining a strong electronic identification (e-ID), necessary for using digital public services, was not straightforward for non-EU migrants.<br><br>Health conditions: Poor health, such as memory impairment, language disorders, and visual or hearing impairments, particularly challenged the use of digital health services. These conditions made on-screen reading difficult, and participants preferred receiving health information in a letter or printing it from a digital service on paper.<br><br>Financial barriers and device issues: For some participants, such as the unemployed and older adults, the lack of suitable devices due to financial reasons or problems with devices functioning at home hindered their ability to use and benefit from digital health services. These issues sometimes forced participants to seek services elsewhere.<br><br>Communication challenges: Digital services were not seen as applicable for all situations. Participants experienced challenges related to the nature of communication and poor interaction in the digital environment. Face-to-face services were perceived as more effective for handling more demanding and complex matters, while routine tasks like booking appointments and checking health records online were more doable digitally.<br><br>Privacy and interaction: Poor interaction was a major barrier for mental health service users, who found it difficult to express their health service needs remotely. Digital mental health consultations lacked warmth and felt distant without facial expressions and tones. Using digital health services at home in the presence of others was challenging and did not allow for privacy.<br><br>Security concerns: Participants had concerns related to the security of digital health services and feared that personal data might be compromised. The rapid transition to	

PUBLICATION	TITLE	BARRIERS	FACILITATORS
		digital services during the COVID-19 pandemic sometimes resulted in using platforms perceived as insecure. Lack of sufficient security expertise worried participants, as they felt unable to protect their computers from hackers.<br><br>Trust and quality: Distrust in the quality of digital health services emerged from the interviews. Some participants had less confidence in digital health services than in face-to-face services.<br><br>Cultural preferences: Many participants preferred traditional face-to-face meetings over digital ones. Digital services were not seen to provide an experience equivalent to face-to-face encounters. Migrant participants, in particular, valued personal communication, including gestures, facial expressions, and touches.<br><br>Integration challenges: The challenges associated with integrating digital resources into health infrastructure were mainly related to the unavailability of digital alternatives as desired by participants or participants being unaware of existing digital service options and their value. Some high users hoped for better information about the possibilities of using digital health services. Migrant participants noted that only some web pages were available in their mother tongue, making relevant information difficult to find and understand.	
Kalicki 2021	Barriers to telehealth access among homebound older adults	deafness prevent 27% of patients from interacting via video.<br><br>Need for Assistance: Nearly 50% of tele-naïve patients require help to use telehealth, such as a caregiver's presence.<br><br>Lack of Caregiver Support: Among those needing assistance, 28% lack a caregiver to help facilitate the interaction.	
Kester 2022	Telepharmacy services in acute care: Diverse needs within a large health system.	Multiple State Regulations – Different licensing and compliance requirements across 10 states complicate operations.<br><br>Variation in Hospital Practices – Differences in formularies, protocols, and EHR systems create complexity for telepharmacists.<br><br>Communication Challenges – Telepharmacists rely on faxing, messaging, and phone calls, which may not always be seamless.<br><br>Multiple-Site Coverage Difficulties – Managing many small hospitals with different systems is more complex than handling one large hospital.<br><br>IT and Infrastructure Issues – Dependence on stable internet, power, and IT support is critical, with contingency plans needed.	24/7 Pharmacist Coverage – Enables round-the-clock medication order review for hospitals that lack onsite pharmacists.<br><br>Increased Efficiency – Reduces median medication order review turnaround times by 50% to 70%.<br><br>Work-from-Home Model – Helps in recruitment, retention, and reducing disruptions due to weather or travel.<br><br>Cost Sharing Among Hospitals – Smaller hospitals can afford pharmacist services by pooling resources.

PUBLICATION	TITLE	BARRIERS	FACILITATORS
		Need for Ongoing Training – Pharmacists must stay updated on site-specific rules, software, and protocols.<br><br>Scope Limitations – Telepharmacists may be restricted from handling oncology medications, neonatal care orders, and other specialized tasks.<br><br>State-Specific Work Requirements – Some states require telepharmacists to work from licensed facilities, limiting work-from-home options.	Technology Integration – Use of electronic health records (EHR) and real-time audiovisual connections enhances service delivery.<br><br>Flexibility in Staffing – Allows adjustments to coverage based on demand and hospital needs.<br><br>Regulatory Compliance Support – Helps hospitals meet state requirements for pharmacist medication order verification.<br><br>Customer Satisfaction – Regular surveys indicate positive feedback from hospitals on telepharmacy services.
Khalifa 2021	Utilizing Health Analytics in Improving the Performance of Hospitals and Healthcare Services: Promises and Challenges	- Technological Barriers:<br>Data Complexity and Integration Issues: Healthcare systems generate vast amounts of data from multiple sources (e.g., electronic health records, medical devices, and billing systems). Integrating this data into a cohesive system for analysis can be challenging, due to variations in formats, standards, and systems.<br><br>Lack of Interoperability: Different healthcare systems often use incompatible technologies, which makes it difficult to share data across institutions. This hinders the development of comprehensive analytics solutions.<br><br>Data Security and Privacy Concerns: Handling sensitive patient data while ensuring compliance with regulations like GDPR or HIPAA can pose significant challenges for analytics systems. Ensuring the privacy and security of health data is critical but complex.<br>Resource Constraints: Advanced analytics tools often require significant financial investments in technology infrastructure, software, and skilled personnel, which may not be feasible for all healthcare organizations.<br><br>- Human Barriers:<br>Resistance to Change: Healthcare professionals may be reluctant to adopt new technologies, fearing that analytics systems could replace human judgment or alter traditional workflows.<br>Skill Gaps: There is often a lack of expertise among healthcare workers in understanding and utilizing health analytics tools effectively. Many organizations lack personnel with the necessary skills to interpret complex data outputs or manage	Alignment with Organizational Goals: Ensuring that health analytics projects are aligned with the overall goals of the healthcare organization is a key facilitator for success. For example, using analytics to improve patient outcomes, reduce costs, or enhance operational efficiency makes it easier to secure support from leadership and staff.<br><br>Focus on End-User Requirements: Involving clinicians, administrators, and other stakeholders in the design and implementation of analytics systems can greatly enhance adoption. Customizing solutions to meet the specific needs of users ensures that the tools will be perceived as helpful and relevant.<br><br>Strong Data Governance Framework: Implementing a clear data governance structure can help ensure that data is accurate, reliable, and properly managed. This includes establishing protocols for data ownership, security, and privacy,

PUBLICATION	TITLE	BARRIERS	FACILITATORS
		analytics systems.<br>Trust in Data: Clinicians and staff may doubt the accuracy or relevance of the insights generated by analytics systems, particularly if they are not involved in the implementation process.<br><br>- Organizational Barriers:<br>Lack of Strategic Vision: Many healthcare organizations struggle to develop a clear and cohesive vision for how health analytics will be integrated into their operations. Without strong leadership and strategic alignment, projects can fail to gain traction.<br>Cultural Challenges: Organizational cultures that are resistant to innovation can pose significant challenges to implementing health analytics. Success often requires a cultural shift towards data-driven decision-making, which may be difficult to achieve.<br>Data Governance Issues: Ensuring the accuracy, availability, and accountability of data throughout its lifecycle is a common challenge. Effective data governance policies are often lacking, which can lead to poor data quality and hinder analytics efforts.	which are essential for building trust in the system.<br>Co-Creation and Collaboration:<br><br>Collaborating with stakeholders, including clinicians, IT professionals, and data scientists, fosters co-creation of solutions. This approach allows for the development of systems that are more likely to be embraced by end users and integrated smoothly into workflows.<br>Training and Capacity Building:<br><br>Providing training to healthcare professionals on how to use and interpret health analytics is a key facilitator. Building capacity within the organization ensures that staff are equipped with the skills needed to effectively utilize the data and insights generated by analytics tools.<br>Pilot Programs and Gradual Implementation:<br><br>Introducing health analytics through pilot programs can be an effective way to demonstrate the value of the technology without overwhelming the organization. Gradually scaling up the use of analytics after successful pilots helps to build confidence and minimize resistance.
Khodadad-Saryazdi 2021	Exploring the telemedicine implementation challenges through the process innovation approach: A case study research in the French healthcare sector	Supply Burden: The document highlights that the rapid implementation of telemedicine required a significant increase in the supply of digital devices and internet services. This was challenging due to the sudden spike in demand and limited availability, making it difficult for healthcare providers to ensure that all patients had the necessary equipment and connectivity.<br><br>Administrative Burden: The implementation of telemedicine also increased the administrative burden on healthcare providers. This included additional paperwork, new protocols for telehealth sessions, and the need for thorough documentation to meet regulatory requirements. The increase in administrative tasks often took time away from direct patient care.	Administrative Body Cooperation: The cooperation of administrative bodies is crucial in the successful implementation of telemedicine. This involves aligning policies, streamlining approval processes, and ensuring that all regulatory requirements are met. Administrative support helps in reducing bureaucratic hurdles and facilitates smoother integration of telehealth services into existing healthcare systems.

PUBLICATION	TITLE	BARRIERS	FACILITATORS
		Evaluation Burden: Evaluating the effectiveness of telemedicine services posed a challenge. Traditional metrics and evaluation methods were not always suitable for telehealth, and there was a need to develop new frameworks to assess outcomes accurately. This included evaluating patient satisfaction, clinical outcomes, and the cost-effectiveness of telemedicine services.<br><br>Work Habit Change: The shift to telemedicine required significant changes in the work habits of healthcare providers. Many providers had to adapt to new technologies and workflows, which required time and training. The transition was often met with resistance due to the disruption of established routines and the learning curve associated with new systems.<br><br>Financial Limits: Financial constraints were a significant barrier to the widespread adoption of telemedicine. The costs associated with purchasing new equipment, training staff, and maintaining telehealth systems were substantial. Additionally, reimbursement policies for telemedicine services were often inadequate, making it financially challenging for providers to sustain these services.<br><br>Staff Overloading: The implementation of telemedicine added to the workload of healthcare staff. In addition to their regular duties, staff had to manage telehealth appointments, troubleshoot technical issues, and ensure that patients were comfortable using the new systems. This often led to burnout and reduced job satisfaction among healthcare providers.<br><br>Negative Belief: There were negative beliefs and scepticism about the effectiveness of telemedicine among both healthcare providers and patients. Some providers were hesitant to adopt telehealth due to concerns about the quality of care, while some patients were wary of using technology for their healthcare needs. Overcoming these negative perceptions required significant effort in education and demonstration of the benefits of telemedicine.	Management Support Activities: Management support is essential for driving telemedicine initiatives. Activities include providing the necessary resources, offering training programs, and ensuring that staff members are well-equipped to handle new technologies. Effective leadership and clear communication from management can motivate staff and promote the adoption of telehealth services.<br><br>Health Staff Collaboration: Collaboration among healthcare staff is vital for the successful implementation of telemedicine. This includes teamwork between doctors, nurses, IT staff, and administrative personnel. Effective communication and coordination help in addressing challenges quickly and ensuring that telehealth services are delivered efficiently.<br><br>Champion: Having a champion within the organization who advocates for telemedicine can significantly impact its success. This individual, often a senior clinician or a respected leader, can drive the initiative forward, address concerns, and garner support from other staff members. Champions play a crucial role in overcoming resistance to change and promoting the benefits of telehealth.<br><br>IT Efficiency: Efficient IT systems are the backbone of telemedicine services. This includes reliable hardware, user-friendly software, and robust network infrastructure. Ensuring that IT systems are efficient and capable of handling telehealth demands is crucial for providing seamless and uninterrupted

PUBLICATION	TITLE	BARRIERS	FACILITATORS
			services.<br><br>ISD Efficiency: Information Systems Department (ISD) efficiency is critical in managing and maintaining telehealth platforms. This includes ensuring data security, integrating systems with existing healthcare infrastructure, and providing technical support to staff and patients. An efficient ISD helps in minimizing technical issues and enhancing user experience.<br><br>Process Definition: Clearly defined processes are essential for the effective implementation of telemedicine. This includes establishing protocols for telehealth consultations, setting guidelines for patient interactions, and defining roles and responsibilities. Well-defined processes ensure consistency and quality in telehealth service delivery.<br><br>Feedback: Regular feedback from both healthcare providers and patients is important for continuous improvement of telemedicine services. Gathering feedback helps in identifying areas for improvement, addressing issues promptly, and enhancing the overall quality of care. Implementing a feedback loop ensures that telehealth services evolve based on user needs and experiences.<br><br>Patient Support Activities: Providing support to patients in using telemedicine services is crucial for their success. This includes offering technical assistance, educating patients on how to use telehealth platforms, and addressing their concerns. Effective patient support activities enhance user satisfaction and

PUBLICATION	TITLE	BARRIERS	FACILITATORS
			encourage the continued use of telehealth services.<br><br>Positive Belief: Cultivating a positive belief in telemedicine among healthcare providers and patients is essential. This involves highlighting the benefits, sharing success stories, and demonstrating the effectiveness of telehealth. Building a positive perception helps in overcoming scepticism and promotes wider acceptance and adoption of telemedicine services.
Kilova 2022	Electronic Health in the practice of general practitioners in Bulgaria	What are your concerns about e-health, if you have any?,<br>there was more than one choice. Around half of the surveyed GPs feared legal regulation (n = 189; 49.6%), followed by difficulties in using technology (n = 181; 47.5%), as well as lack of technical knowledge (n = 135; 35.4%).	
Klaver 2021	Relationship between perceived risks of using mhealth applications and the intention to use them among older adults in the Netherlands: Cross-sectional study	Performance<br>risk (β=–.266; P=<.001), legal concern (β=–.125; P=.007), and privacy risk (β=–.100; P=.03) were found to be negatively<br>correlated to intention to use mHealth applications,	Privacy risk refers to the belief that personal information may be abused due to mHealth application usage, with older adults particularly concerned about data security and the potential misuse of sensitive information.<br><br>Performance risk is the doubt about the capability of mHealth applications to achieve desired health outcomes, including scepticism about technology replacing healthcare professionals and the overall quality and usability of these technologies.<br><br>Legal concern involves worries about inadequate law enforcement for mHealth applications and the potential for personal data to be improperly combined with clinical data, especially in home-use scenarios where specific legislation may not apply.<br><br>Trust is the perceived credibility of an mHealth application and the people

PUBLICATION	TITLE	BARRIERS	FACILITATORS
			behind it, with a higher level of trust being positively associated with the intention to use mHealth technologies; whereas trust (β=.352; P=<.001) was found to be positively correlated to the intention to use mHealth applications
Kosari 2020	Pharmacists' Perspectives on the Use of My Health Record.	- Privacy and Confidentiality Concerns (Patients): 81% of pharmacists reported that patients' concerns about privacy or confidentiality are likely to act as a barrier to MHR use.<br><br>- Privacy and Confidentiality Concerns (Pharmacists): 46% of pharmacists had their own concerns about privacy and confidentiality.<br><br>- Lack of IT Support: 30% of pharmacists identified a lack of IT support as a likely barrier.<br><br>- Training for Setup and Access: 41% of pharmacists reported that insufficient training to set up and access the system is a likely barrier.<br><br>Training for Confident Use: 41% of pharmacists indicated that a lack of training to enable confident use of the system is a likely barrier.<br><br>- Interference with Dispensing Processes: 21% of pharmacists felt that MHR use could interfere with existing dispensing processes, while over 40% considered this unlikely.<br><br>- Workplace Promotion of the System: Nearly 48% of pharmacists expressed that it was 'neither likely nor unlikely' that their workplace would not promote the use of the system.	Potential barriers identified by pharmacists included patients' concerns about privacy (81%),<br>pharmacists' own concern about privacy (46%), lack of training, access to and confidence in using the system. Sixty six percent of respondents had concerns about the accuracy of information contained<br>within MHR, particularly among hospital and general practice pharmacists (p = 0.016) and almost<br>half (44%) had concerns about the security of information in the system, mainly pharmacists working<br>at general practice and providing medication review services (p = 0.007)
Kruse 2020	Utilization barriers and medical outcomes commensurate with the use of telehealth among older adults: Systematic review	Reviewers identified 14 themes for barriers.<br>The most common of which were technical literacy (25/144 occurrences, 17%), lack of desire (19/144 occurrences, 13%), and cost (11/144 occurrences, 8%).Technical Literacy: Difficulty understanding and navigating technology (e.g., "I do not understand technology, I cannot navigate menus, I do not know how").<br><br>Lack of Desire: Disinterest or unwillingness to use technology (e.g., "I do not want to, I am too busy, laziness").<br><br>Cost: Financial constraints (e.g., "Too expensive, we live off a fixed income").<br><br>Lack of Technical Support: Inadequate help from friends or family, difficulty understanding interfaces (e.g., "My friends or family are not able to help me, I do not	

PUBLICATION	TITLE	BARRIERS	FACILITATORS
		understand the interface").<br><br>Visual Acuity: Issues with small fonts, icons, or poor color contrast (e.g., "Fonts or icons are too small, color contrast issues").<br><br>Social Implications: Concerns about social aspects of using technology (e.g., "I do not want to bother a first responder, I do not want a stranger coming to my house").<br><br>Ownership of Technology: Lack of access to necessary devices (e.g., "No phone, no computer, no internet access").<br><br>Privacy and Security Concerns: Worries about data security and privacy.<br><br>Medical Literacy: Difficulty understanding medical information and terminology (e.g., "I do not understand terminology, I do not understand test results").<br><br>Trust of the Internet: Distrust in using online platforms.<br><br>Mental Acuity: Cognitive challenges with technology (e.g., "Computers confuse me, the interface is too complex, I cannot focus for very long").<br><br>Hand-Eye Coordination: Difficulty using technology due to physical limitations (e.g., particularly relevant for those with Parkinson's disease).<br><br>Auditory Acuity: Hearing impairments affecting the use of technology.<br><br>Computer Anxiety: General fear or anxiety about using computers and technology.	
Kruse 2021	Telemedicine and health policy: A systematic review		The fifteen facilitators mentioned most often were increased access, increased convenience, im-<br>proved population health, care enabled through mobile technology, self-efficacy, increased patient-to-<br>provider communication, cost advantages, efficacy of modality, increased health outcomes, reaches developing countries, increased quality, a positive previous experience, and a secure means of care. The<br>twelve barriers mentioned most often were the increased cost to providers, patient privacy, technical literacy, state

PUBLICATION	TITLE	BARRIERS	FACILITATORS
			licensing, data security, socioeconomics, limited reimbursements, issues of interoperability,<br>patient safety, less personal means of care, misaligned incentives, and ethical concerns. Increased Access: Enhances the ability to reach services that would otherwise be inaccessible, particularly for vulnerable and rural populations.<br><br>Care Enables Other Resources (Educational/Technological): Utilizes mobile phones for medical education and enhanced decision-making, making it easier for patients to receive and act on health information.<br><br>Increased Convenience: Provides logistical benefits such as reduced travel, parking issues, and not needing to take time off work, improving the overall patient experience.<br><br>Improved Population Health: Leads to health improvements in specific populations, such as those with HIV, diabetes, or dementia, by providing tailored interventions and frequent monitoring.<br><br>Care Enabled Through Smart Mobile Technology: Leverages mobile devices to access health services conveniently, integrating healthcare into daily life.<br><br>Self-Efficacy: Enhances patients' understanding of their conditions, promoting self-care and positive behavioral changes.<br><br>Increased Patient-to-Provider Communication: Strengthens

PUBLICATION	TITLE	BARRIERS	FACILITATORS
			communication between patients and providers through digital platforms, ensuring continuous and effective interactions.<br><br>Cost Advantages: Achieves cost savings by avoiding higher levels of care and offsetting technology costs, making healthcare more affordable.<br><br>Efficacy of Modality: Demonstrates the clinical effectiveness of telemedicine, often comparable to traditional face-to-face care.<br><br>Increased Health Outcomes: Provides more frequent and timely interventions, leading to improved health outcomes and better disease management.<br><br>Reaches Developing Countries: Extends healthcare access to developing countries through technologies like SMS and mobile networks, where robust internet infrastructure may be lacking.<br><br>Increased Quality: Ensures higher quality care through better data quality, improved continuity of care, and more comprehensive follow-ups.<br><br>Positive Previous Experience: Builds on positive previous experiences with telemedicine, encouraging continued use and high patient satisfaction.<br><br>Preferred Modality Over Traditional Care: Some patients prefer telemedicine over traditional in-person care due to convenience and the ability to avoid crowded waiting rooms.

PUBLICATION	TITLE	BARRIERS	FACILITATORS
			Secure Means of Care: Provides a secure and private way for patients to seek care, reducing fears of judgment and ensuring confidentiality. Increased Access: 18 (16%)<br>Care Enables Other Resources (Educational/Technological): 16 (14%)<br>Increased Convenience (Time, Miles, Work, Parking, Childcare, etc.): 9 (8%)<br>Improved Population Health: 8 (7%)<br>Care Enabled Through Smart Mobile Technology: 8 (7%)<br>Self-Efficacy: 8 (7%)<br>Increased Patient-to-Provider Communication: 7 (6%)<br>Cost Advantages: 7 (6%)<br>Efficacy of Modality: 6 (5%)<br>Increased Health Outcomes: 6 (5%)<br>Reaches Developing Countries: 5 (4%)<br>Increased Quality: 5 (4%)<br>Positive Previous Experience: 4 (3%)<br>Modality Preferred Over Traditional Care: 4 (3%)<br>Secure Means of Care: 2 (2%)<br>Improved Interoperability: 1 (1%)<br>Local Telephone Triage Overcomes Impersonal Nature: 1 (1%)<br>User Experience Using Modality: 1 (1%)
Kruse 2023	Analyzing the Effect of Telemedicine on Domains of Quality through Facilitators and Barriers to Adoption: Systematic Review	Staff Training: 31 (33.3%)<br>May Not Be Preferred Modality: 30 (32.3%)<br>Cost: 29 (31.2%)<br>Low Reimbursement: 3 (3.2%)	Effective: 33 (19.9%)<br>Meets a Digital Preference: 32 (19.3%)<br>Convenience: 31 (18.7%)<br>Patients Value Technology: 30 (18.1%)<br>Savings in Time and Mileage: 30 (18.1%)<br>Education at Own Pace: 7 (4.2%)<br>Avoids Stigma: 2 (1.2%)<br>Patients Value Personal Guidance: 1 (0.6%)
Kushniruk 2021	The Human Factors of AI in Healthcare: Recurrent Issues, Future Challenges and Ways Forward	Integration into Existing Healthcare Systems:<br>AI technologies often struggle to integrate seamlessly with existing healthcare systems like electronic health records (EHRs), hospital workflows, and clinical decision-making processes, creating operational disconnects.	AI Systems for Well-Defined Tasks:<br>AI technologies that handle specific, well-defined tasks, such as diagnostic image interpretation, are more likely to gain acceptance as they focus on narrow areas

PUBLICATION	TITLE	BARRIERS	FACILITATORS
		Interoperability:<br>Healthcare organizations use a variety of systems that often lack interoperability, meaning they cannot easily share or exchange data, which complicates AI integration and data flow.<br><br>Trust and Liability Concerns:<br>Healthcare professionals may be hesitant to rely on AI systems due to concerns over transparency, trust, and legal liability, especially when it's unclear how AI reaches decisions (the "black-box" issue).<br><br>Generalizability of AI Solutions:<br>AI models are typically trained on specific datasets, making it difficult to generalize them for use in different healthcare settings, patient populations, or regions.<br><br>Transparency and Explainability:<br>Many AI systems lack the ability to provide clear explanations for their decisions, leading to hesitancy in adoption by clinicians who prefer systems they can fully understand and justify.	where they can outperform humans.<br><br>User-Centered Design and Usability:<br>AI tools designed with a strong focus on usability, involving clinicians and end-users during the design phase, enhance adoption by fitting seamlessly into existing workflows and being easier to use.<br><br>Gradual Integration into Healthcare Workflows:<br>Gradually integrating AI into healthcare processes, starting with non-critical or supportive tasks, helps to reduce resistance and allows users to familiarize themselves with the technology before scaling up.<br><br>Regulatory Approvals and Certifications:<br>AI systems that undergo rigorous testing and receive regulatory approvals from authorities like the FDA gain credibility and trust, making healthcare providers more likely to adopt them.<br><br>Collaboration and Co-Creation with Stakeholders:<br>Involving healthcare professionals, IT experts, and AI developers in the co-creation process of AI tools ensures that the final product meets the practical needs of its users and is more likely to be accepted.
Lavallee 2020	mHealth and patient generated health data: stakeholder perspectives on opportunities and barriers for transforming healthcare	Effort, Time, and Resources: Capturing and utilizing PGHD requires significant effort, time, and resources from both healthcare consumers and providers.<br><br>Diversity of Data Types and Modes: While diverse tracking methods allow customization, they also present challenges in managing data across multiple platforms. Interoperability between devices and platforms is needed to reduce complexity and facilitate sustained use.	

PUBLICATION	TITLE	BARRIERS	FACILITATORS
		Provider and Patient Dilemmas: Tailoring PGHD tracking tools to individual patient needs may reduce patient burden but increases provider burden due to the variety of data formats and measurement standards.<br><br>Lack of Standards: There is a lack of standards for how data is tracked using different technologies and for ensuring interoperability across devices and platforms. This limits the clinical utility of PGHD.<br><br>Provider Burden: If PGHD is not easy to read, interpret, and act upon, it may be ignored by healthcare providers.<br><br>System-Level Integration: Key barriers include the potential burden on providers and healthcare teams, impacts on resources and workflow within clinics, and the need for intensive training on data interpretation and use.<br><br>Clinical Context and Standardization: Carefully selecting clinical contexts for PGHD use and aligning with existing health system structures and standards is essential. Leveraging electronic medical records to standardize data reception and review is critical.<br><br>Lack of Reimbursement Mechanisms: Current payment models do not support PGHD. The lack of reimbursement mechanisms and healthcare policies supporting PGHD use hinders its advancement within the health system. Consumers:<br>Accessibility Issues<br>Burden of Tracking<br>Feasibility<br>Lack of Accountability<br>Lack of Actionability<br>Lack of Buy-In<br>Lack of Data Integration<br>Lack of Incentive<br>Lack of Standards<br>Limited Resources<br>Sustainability of Use<br>Unknown Accuracy of Data<br><br>Providers:<br>Accessibility Issues<br>Burden of Tracking<br>Feasibility<br>Lack of Actionability<br>Lack of Buy-In	

PUBLICATION	TITLE	BARRIERS	FACILITATORS
		Lack of Data Integration<br>Lack of Evidence for Use<br>Lack of Incentive<br>Lack of Standards<br>Limited Resources<br>Sustainability of Use<br>Unknown Accuracy of Data<br><br>Administrators:<br>Feasibility<br>Lack of Data Integration<br>Lack of Evidence for Use<br>Lack of Incentive<br>Lack of Standards<br>Limited Resources	
Leonard 2020	Investigating the barriers and facilitators to implementing an eHealth innovation into a resource-constrained setting: A South African case study	- Lack of Political Support: Absence of political support for the eHealth innovation, affecting its longevity and sustainability.<br><br>- Interoperability Issues: Potential barriers related to the application's language not being in the local language.<br><br>- Lack of Protocols and Policies: Absence of established protocols or policies for CHW communication, feedback, and conflict management.<br><br>- Absence of Dissemination Channels: No accessible dissemination channel for eHealth innovation companies to collaborate with the Department of Health.<br><br>- Mobility of Patients: The mobility of patients was identified as an implementation barrier.<br><br>- Limited Support for eHealth at Different Health System Levels: Barriers exist at different health system levels, emphasizing the need to consider the interrelatedness of community, health providers, district, and national health system levels.	- Effective Design and Functionality of eHealth Innovation: The design and functionality of the eHealth innovation are well-suited to resource-constrained settings, making it an effective facilitator for implementation.<br><br>- Needs-Based Innovation: The eHealth solution addresses the need for affordable, easily understandable, and accessible hearing screening in developing countries.<br><br>- Local Language Support: Including an option to change the application's language to the local language to improve interoperability with Community Health Workers (CHWs).<br><br>- Establishing Protocols and Policies: Establishing protocols or policies at the health provider level to facilitate CHW communication, feedback channels, and conflict management.<br><br>- Supportive Regulatory and Policy Environment: South Africa has a supportive regulatory and policy

PUBLICATION	TITLE	BARRIERS	FACILITATORS
			environment.<br><br>- Encouraging National Collaborations: Recommendations for the South African National Department of Health to establish dissemination channels to encourage collaboration with eHealth innovation companies.<br><br>- Improving Community Knowledge: Improving health education in primary schools and using existing dissemination channels (cell phones, television, radio) to broadcast information on hearing and vision screening.<br><br>- Understanding Cultural Context and Managing Beliefs: Emphasizing the importance of understanding cultural contexts and managing actors' beliefs.<br><br>- Partnerships for Sustainable Funding: Partnering with national actors to ensure sustainable funding sources.
Lew 2023	Perceptions and Attitudes of Patients and Health Care Stakeholders on Implementing a Telehealth Service for Preoperative Evaluation: A Qualitative Analysis	Challenges in the Use of Telehealth Platforms:<br>Patients often lack the digital literacy and equipment needed for video consultations. Concerns exist that video consults may seem less thorough, potentially compromising care. Technical issues like poor Wi-Fi and log-in problems further hinder the telehealth experience. Both patients and providers require training in videoconferencing.<br><br>Limitations of Telehealth:<br>Video consultations are unsuitable for patients needing physical examinations. This can make it difficult to accurately diagnose conditions. Patients prefer having the option between video and physical consultations, with face-to-face consults being favored for serious conditions like oncology due to the personal touch. Communication barriers, such as the ability to ask questions, are also a concern.<br><br>Low Uptake of Online Preanesthesia Health Assessment:<br>The uptake of online preanesthesia health assessments is low, impacting patient triage quality. Issues include poorly placed internet links in text messages and technical problems with digital returns. Suggestions to improve uptake include providing hard	Skills and Resources:<br>Formal training in technological and communication skills is essential for effective video consultations. The pandemic has underscored the importance of equipping healthcare professionals with these skills.<br><br>Receptiveness Toward the New Telehealth Workflow:<br>Healthcare providers and patients are open to adopting new telehealth workflows for preoperative evaluations, provided there is adequate training.

PUBLICATION	TITLE	BARRIERS	FACILITATORS
		copies, better link placement, and training patient service associates to assist patients. The reliability of patient responses is also questioned.	
Li 2020	Patients' Perceptions of Barriers and Facilitators to the Adoption of E-Hospitals: Cross-Sectional Study in Western China.	Inability to Operate Electronic Devices: 67.0% reported this as a barrier.<br><br>Preference for Face-to-Face Healthcare: 30.4% were accustomed to traditional in-person care.<br><br>Doubts About Authenticity and Reliability: 24.2% had concerns about the authenticity and reliability of e-hospitals.<br><br>Perceptions of Uselessness: 10.4% believed e-hospitals were not useful.<br><br>Concerns with Insurance Reimbursement: 7.6% were worried about insurance issues.	Convenience: 94.7% of participants considered convenience as a major facilitator for e-hospital adoption.<br><br>Improved Access to Skilled Experts: 72.2% noted this as a facilitator.<br><br>Improved Health Outcomes: 27.2% believed e-hospitals could lead to better health outcomes.<br><br>Privacy Protection: 26.6% cited privacy protection as a benefit.<br><br>Active Participation in Disease Self-Management: 21.3% saw this as an advantage. Additionally, the prominent facilitators of e-hospitals<br>were convenience (641/677, 94.7%) and accessibility to skilled medical experts (489/677, 72.2%). The most frequently perceived<br>barrier varied among age groups; seniors most often reported their inability to operate technological devices as a barrier (144/166,<br>86.7%), whereas young participants most often reported that they avoided e-hospital services because they were accustomed to face-to-face consultation (39/52, 75%)
Li 2022	Readiness of healthcare providers for e-hospitals: a cross-sectional analysis in China before the COVID-19 period	Lack of Knowledge in Operating Smart Devices: 18.5% of participants were unwilling to work at e-hospitals due to their inability to operate smart devices.<br><br>Lack of Time: 48.4% reported not having extra time to work at an e-hospital, with the highest proportion from secondary hospitals (67.5%).<br><br>Underdeveloped Licenses and Medical Dispute Policies: 43.6% were concerned about insufficient development in licensing and policies for medical disputes in e-hospitals.<br><br>Perceived Unreliability: 53.5% believed that e-hospitals were not reliable.	Healthcare providers who had positive attitudes towards e-<br>hospitals considered improved efficiency, patient satisfaction,<br>communication among physicians, increased reputation<br>and income, and alleviated workload to be advantages of<br>adoption. The participants who were unwilling to work at

PUBLICATION	TITLE	BARRIERS	FACILITATORS
		Poor Patient Reception: 18.2% expressed concerns that e-hospitals were not well-received by patients.	e-hospitals perceived lack of time, insufficient authenticity/<br>reliability and underdeveloped policies as potential barriers. Improved Convenience and Efficiency: Over 90% perceived that e-hospitals could enhance convenience and efficiency in healthcare, with 94.6% from tertiary public hospitals highlighting this.<br><br>Alleviating Workload: 66.8% of participants from tertiary public hospitals saw e-hospitals as a tool for reducing workload.<br><br>Improving Patient Satisfaction: 72.8% from tertiary public hospitals believed e-hospitals could enhance patient satisfaction.<br><br>Facilitating Physician Communication and Learning: 41.9% from private hospitals viewed e-hospitals as a means for physicians to communicate and learn from each other.<br><br>Increasing Income and Reputation: 19.6% from secondary public hospitals considered e-hospitals as a way to increase their income and reputation.
Li 2023	Technical/Algorithm, Stakeholder, and Society (TASS) barriers to the application of artificial intelligence in medicine: A systematic review	Technical/Algorithm Level:<br>-Lack of Explainability: Clinicians and patients often find AI's decision-making process too complex and opaque, leading to trust issues.<br>- Need for Validation Protocols: Current validation methods for AI models are inconsistent and often inadequate, with a tendency to publish "proofs of concept" without rigorous validation.<br>- Need for Standards Enabling Interoperability: The fragmented nature of healthcare data requires standardized protocols for data sharing and device communication.<br>- Need for Reporting Guidelines: There is significant variation in reporting AI clinical trial results, necessitating the development of specific guidelines for AI studies.<br>- Need for Standardization of Performance Metrics: Lack of consistent metrics for evaluating AI performance makes it difficult to compare models.	Technical/Algorithm Level:<br>- Performance Expectancy: AI technologies must demonstrate clear performance benefits, such as improved diagnostic accuracy and efficiency.<br>- Effort Expectancy: AI systems should be easy to use and integrate seamlessly into existing workflows.<br>- Initial Trust: Building initial trust through transparent and explainable AI models.

PUBLICATION	TITLE	BARRIERS	FACILITATORS
		- Lack of Plan for Updating Algorithms: Predictive performance of AI models deteriorates over time, requiring plans for regular updates and recalibrations.<br><br>Stakeholder Level:<br>- Provider Related Concerns:<br>Job Loss: Fear of job loss due to AI replacing human roles.<br>- Skills Loss: Concern about the erosion of clinical skills as tasks are automated.<br>- Workflow Challenges: Integration of AI into clinical workflows may increase workload and cognitive overload.<br>- Patient Related Concerns:<br>Loss of Patient Autonomy and Consent: Fear that AI may lead to a return of paternalism in healthcare, with decisions made by machines instead of human doctors.<br>- Disturbing the Patient-Clinician Relationship: AI may complicate or reduce face-to-face interactions between patients and clinicians.<br>- Lack of Trust in AI: Skepticism about AI's reliability and functionality compared to human doctors.<br>- Organization Related Concerns:<br>Logistical Challenges: Issues with obtaining appropriate data, updating infrastructure, and ensuring smooth integration into existing systems.<br>- Lack of Strategic Plan: Absence of a clear strategy for AI implementation in healthcare organizations.<br>- Lack of Cost-effectiveness Analysis and Proof of Efficacy: Insufficient evidence demonstrating the cost-effectiveness and improved outcomes of AI technologies.<br><br>Society Level:<br>- Privacy: Concerns about patient privacy due to the creation and use of large datasets.<br>Liability: Unclear legal framework regarding responsibility for AI errors leading to patient harm.<br>- Bias and Social Justice: Potential for AI to perpetuate biases and inequities due to non-representative datasets.<br>- Education: Need for better education and training for both healthcare providers and the public on the use and benefits of AI.	Stakeholder Level:<br>- Perceived Usefulness: AI systems that are seen as useful and beneficial by healthcare providers are more likely to be adopted.<br>- Top Management Support: Strong support from hospital leadership and key stakeholders is crucial for successful AI implementation.<br>- Change Management: Effective change management strategies can help address resistance to AI adoption.<br><br>Society Level<br>- Education and Training: Comprehensive training programs for healthcare providers and public education campaigns to build understanding and trust in AI.<br>- Government and Regulatory Support: Clear regulations and guidelines from government bodies to ensure safe and effective use of AI.<br>- Collaboration and Partnerships: Encouraging collaboration between AI developers, healthcare providers, and policymakers to create robust and equitable AI solutions. We developed 19 major<br>themes, which we categorized into three levels: the Technical/Algorithm, Stakeholder, and Social levels (TASS). These themes included: Lack of Explainability, Need for Validation Protocols, Need for Standards for Interop-erability, Need for Reporting Guidelines, Need for Standardization of Performance Metrics, Lack of Plan for<br>Updating Algorithm, Job Loss, Skills Loss, Workflow Challenges, Loss of Patient Autonomy and Consent, Dis-turbing the Patient-Clinician Relationship, Lack of Trust in AI, Logistical

PUBLICATION	TITLE	BARRIERS	FACILITATORS
			Challenges, Lack of strategic plan, Lack of Cost-effectiveness Analysis and Proof of Efficacy, Privacy, Liability, Bias and Social Justice, and Education.<br>Conclusion: We identified 19 major barriers to the use of AI in healthcare and categorized them into three levels:<br>the Technical/Algorithm, Stakeholder, and Social levels (TASS).
Liljeroos 2023	Implementation of Telemonitoring in Health Care: Facilitators and Barriers for Using eHealth for Older Adults with Chronic Conditions	Barriers when implementing telemonitoring:<br>Legal, logistical, or economical perspective Challenges in Digital Literacy and Equipment Use: Many patients are unable to use digital consultations without assistance due to the requirement for secure personal identification systems, which are unfamiliar to them.<br><br>Physiotherapists and occupational therapists prefer physical contact for assessments, limiting their use of digital technology.<br><br>Technical malfunctions, such as loss of network contact and patients' lack of experience with digital services, hinder effective use.<br><br>Need for Preparation: Proper preparation and involvement of all team members are crucial. There is a need for planning, safety routines, and ensuring easy access to general practitioners for emergency advice. Nurses and assistant nurses need training to handle worsening patient conditions detected through telemonitoring.<br><br>Patient Concerns: Some patients may feel lonelier with reduced in-person visits, as their primary visitors are care team members. Concerns about the ability of elderly patients to handle telemonitoring technology, as many do not use smartphones or computers regularly.<br><br>Care Team's Perspective: The care team was not fully involved in the decision-making process for implementing telemonitoring, leading to questions about its benefits and financial implications. Concerns about the balance between technology and care, ensuring that technology supports rather than replaces human care.	Facilitators when implementing telemonitoring:<br><br>- Identify healthcare professionals’ previous<br>experiences with telemonitoring.<br>- Identify key persons with positive experiences to help out at the start.<br>- Identify healthcare professionals’ knowledge gaps<br>early.<br>- Include all personnel early in the process to avoid misinterpretations.<br>- Early detection of deterioration ;<br>Previous Experience with Digital Technology: Nurses and assistant nurses have experience using smartphones for accessing care plans and medical treatments, finding it accessible and easy to use. Digital meetings are helpful for evaluating symptoms and signs of deterioration from a distance.<br><br>Importance of Preparation: Early involvement of all personnel and identification of key individuals with positive experiences can facilitate smoother implementation. Proper planning and establishing routines for when technology fails are crucial for successful telemonitoring.<br><br>Positive Expectations and Benefits:

PUBLICATION	TITLE	BARRIERS	FACILITATORS
			Telemonitoring can provide patients with more freedom and empower them to perform self-care activities. Early detection of deterioration through telemonitoring can lead to more preventive healthcare and timely home visits.<br><br>Technological Support: Telemonitoring technology, when set up properly, reduces the need for carrying equipment during home visits and provides accurate health data documentation. Access to daily measurements allows for better prioritization of patient visits.<br><br>Supportive Aid: The technology is seen as a supportive aid rather than a replacement for human care, enhancing security and accuracy in health data management. the attitudes of healthcare professionals can be a significant factor in the acceptance and efficiency of the use of telemonitoring in practice. Therefore, implementing new technology in healthcare should involve healthcare professionals at an early stage to gain common understanding
Lin 2020	Seniors and mobiles: A qualitative inquiry of mHealth adoption among Singapore seniors	- Digital Literacy and Data Plans: Many seniors lack data plans (51%) and digital literacy, limiting their ability to utilize advanced smartphone features and mHealth applications.<br><br>- Low Internet Connectivity: Only 35% can connect to the internet using 3G or 4G, restricting access to online health information and services.<br><br>- Reliance on Basic Features: Most seniors use basic mobile phone features like voice calls and text messaging for healthcare, rather than more advanced functionalities.<br><br>- Complexity of mHealth Applications: Some seniors find mHealth applications tedious or experience laziness in consistently using apps like blood pressure monitors.<br><br>- Mixed Reactions to Health Apps: Seniors' reactions to mHealth applications vary,	- High Smartphone Ownership: A significant number of seniors own smartphones (66%), with ownership higher among younger seniors (87%).<br><br>- Regular Use for Communication: Seniors frequently use their phones for voice calls, text messaging, and mobile instant messaging to stay in touch with family and coordinate daily activities, providing a foundation for potential mHealth use.<br><br>- Health Information Access: Some

PUBLICATION	TITLE	BARRIERS	FACILITATORS
		with some showing disinterest or reservations despite the benefits.	seniors use their smartphones to search for health information online, particularly those who are younger and have higher educational attainment.<br><br>- Positive Experiences with mHealth: Certain seniors enjoy using mHealth applications for fitness tracking and health monitoring, finding them beneficial for expanding their health routines and staying motivated.<br><br>- Willingness to Adopt: Many seniors are willing to use mHealth applications if the benefits are clearly explained and adequate instructions are provided.
Lindenfeld 2023	Synchronous Home-Based Telemedicine for Primary Care: A Review	Technological Barriers (13 studies):<br>Issues with technological literacy, confidence, access, and quality.<br>Lack of internet connectivity in residential settings and rural areas.<br><br>Provider-Level Barriers (6 studies):<br>Hesitancy to adopt telemedicine, change clinic workflow, insufficient training, and staffing shortages.<br>Physician licensing issues across different states.<br><br>Patient-Level Barriers (4 studies):<br>Preference for in-person visits, low levels of health literacy, and concerns over privacy.<br><br>Communication Barriers (4 studies):<br>Challenges for patients with visual or auditive impairments, non-native language preference, and lack of translators.	Technological and Skills-Based Training (12 studies):<br>Training for both patients and providers facilitates telemedicine implementation and reduces complexity.<br><br>Provision of Technological Devices (12 studies):<br>Providing devices to patients supports telemedicine implementation.<br><br>Dedicated Telehealth Workgroup (12 studies):<br>Having a dedicated team to support implementation and create structured workflows.<br><br>Reimbursement of Telemedicine Visits (5 studies):<br>Financial reimbursement for telemedicine consultations facilitates adoption.<br><br>Integration of Appointment and EHR Systems (3 studies):<br>Integrating systems to enable providers to stay present during consultations.

PUBLICATION	TITLE	BARRIERS	FACILITATORS
			Nursing Telehealth Competencies (1 study):<br>Coaching skills, clinical experience integration, communication skills, clinical knowledge, ethical awareness, and supportive attitude are critical.<br><br>Continued Reimbursement During COVID-19 (5 studies):<br>Continued financial support during the pandemic was key to adoption and increased use post-pandemic.
Lingg 2020	Health system stakeholders' perspective on the role of mobile health and its adoption in the Swiss health system: Qualitative study	- Rigid Thinking and Siloed Actions: Successful mHealth implementation requires a new mindset. However, health system actors often provide services in silos, resist digital solutions, and force reimbursement into existing structures instead of developing new financing strategies. There is also a lack of open science and data sharing due to privacy concerns and habitual data management.<br><br>- Complexity of Changing Regulations and Structures: Unresolved legal issues, complex regulations, and the need for an agile mindset hinder mHealth use. Digitally advanced countries illustrate that supportive political frameworks are crucial for digitization. Current regulations should be adapted, and interconnectivity and IT infrastructure challenges need to be addressed.<br><br>- Little understanding of mHealth Use and Clinicians' Role: The role of clinicians in mHealth adoption is critical. Despite mHealth's potential, many solutions depend on clinicians' support and patient engagement. Patients, especially those with serious conditions, prefer face-to-face contact with clinicians. Technological limitations and the risk of reduced personal responsibility for health are also concerns.<br><br>- risk of Polarization of Population Regarding mHealth Use: Diverging attitudes toward digital health, particularly among older generations, may lead to uneven mHealth adoption. This could result in health disparities, with healthier individuals benefiting more from digital health incentives while those in poorer health are disadvantaged.	- New Financing Schemes and Incentive Concepts: Adoption of mHealth is likely to depend on innovative financing models and incentives not yet established in the health system. These could include capitation models, diagnostic-related groups, and value-based systems to motivate clinicians and improve treatment efficiency.<br><br>- Comprehensive Information and Evidence Base: There is a strong demand for robust evidence and comprehensive information on mHealth use cases to build trust among potential users. Systematic clinical studies and quality labels are needed to ensure data accuracy and quality.<br><br>- Easy to Use Alternate Care Approaches: The need for user-friendly alternative care methods is increasing. Shifting public funds towards home-based care can foster novel care approaches and stabilize multimorbid patients, reducing avoidable emergencies.
Lokshina 2019	Evaluation of IoT-driven eHealth: Knowledge management, business	eHealth Entry Barriers:<br>• Functionality.– Medical relevant data and information;– Time needed to accept and develop procedures and algorithms and AI to	

PUBLICATION	TITLE	BARRIERS	FACILITATORS
	models and opportunities, deployment and evolution	handle the reduce data, obtain information and generate reliably warnings and alarms.<br>• Trust.<br>• Security and privacy.– Security and privacy concerns are major impediments to eHealth.– If they are not properly addressed, health care seekers will not feel comfortable in participating, and health care professionals will face huge liability risks.<br>• Usability and "companionship" for both users/patients and health and care providers.<br>• Market development and the required stability in value chains and business plans	
Lori 2020	Experiences of Medicaid Programs and Health Centers in Implementing Telehealth.	FQHC stakeholders identified multiple barriers beyond reimbursement<br>, including infrastructure issues (e.g., insufficient broadband)<br>, technology costs,<br>telehealth as a cost centre, billing challenges<br>, lack of buy-in among FQHC providers<br>, challenges specific to the patient population<br>(e.g., elderly patients,<br>homeless patients)<br>, complexities in adjusting clinic workflow,<br>inadequate<br>supply of specialists to provide telehealth services to FQHC patients<br>complex and time-consuming logistics around credentialing and licensing<br>,<br>and challenges in working with remote<br>providers<br>.	Stakeholders identified several facilitators that<br>supported telehealth implementation, including grant funding<br>, the presence of a clinic champion, collaboration with payers, and implementation of promising practices related to workflow
Lundereng 2023	Health Care Professionals' Experiences and Perspectives on Using Telehealth for Home-based Palliative Care: Scoping Review	Technological Issues (Mentioned in 10 instances):<br>Problems such as unreliable, slow-running, or crashing technology, issues with screen size, lack of internet connectivity, and audio or imaging issues undermine confidence in telehealth.<br><br>Resistance to Change (Mentioned in 8 instances):<br>Lack of motivation and understanding among HCPs, and resistance to adopting new technologies and responsibilities.<br><br>Lack of Desired Functionality (Mentioned in 7 instances):<br>A lack of functionalities like tailored questions, logging capabilities, upload confirmation, chat functionality, and equipment portability leads to reverting to former ways of working.<br><br>Legal and Privacy Concerns (Mentioned in 5 instances):<br>Concerns about data security, lack of privacy during video consultations, and the	Useful Patient-Reported Data (Mentioned in 11 instances):<br>Telehealth provides meaningful and timely patient-reported clinical data that support clinical assessments and decision-making.<br><br>Ease of Use (Mentioned in 10 instances):<br>Telehealth is perceived as acceptable, comfortable, interesting, and easy to learn and use by healthcare professionals (HCPs).<br><br>Added Value (Mentioned in 10 instances):<br>Telehealth improves access to patients,

PUBLICATION	TITLE	BARRIERS	FACILITATORS
		legality of digital patient assessments.<br><br>Integration with Existing Services (Mentioned in 5 instances):<br>Lack of integration with existing healthcare services negatively affects telehealth adoption.<br><br>Concerns about Missing Important Information (Mentioned in 5 instances):<br>Worries about missing important patient information and nuances in visible cues during video consultations.<br><br>Logistical and Support Challenges (Mentioned in 4 instances):<br>Lack of comprehensive technical support and complex logistics around telehealth implementation.	facilitates quick and timely responses, and enhances time efficiency, quality, coordination, and continuity of care.<br><br>Mutual Trust and Relationships (Mentioned in 10 instances):<br>Telehealth fosters a sense of control, security, and improved continuity of care, enhancing relationships with patients and families.<br><br>Remote Visual Assessment (Mentioned in 9 instances):<br>Visual features of telehealth enable HCPs to remotely notice cues of deterioration and assess patients' living situations and emotional states.<br><br>Patient and Family Acceptance (Mentioned in 8 instances):<br>Patients and families find telehealth easy to operate and useful for improving access to care and providing comfort.<br><br>Collaboration and Peer Support (Mentioned in 8 instances):<br>Telehealth facilitates collaboration and peer support among HCPs working at different levels of healthcare.<br><br>Extension of Services in Rural Areas (Mentioned in 4 instances):<br>Telehealth increases the capacity and extension of palliative care services while minimizing the need for traveling in rural areas.<br><br>Prior Experience with Technology (Mentioned in 3 instances):<br>Prior experience with technology enhances HCPs' acceptance and confidence in using telehealth.

PUBLICATION	TITLE	BARRIERS	FACILITATORS
			Proactive Support and Motivation (Mentioned in 3 instances):<br>Support and motivation from telehealth researchers or HCPs delivering telehealth encourage telehealth adoption.
Ma 2022	Usage and perceptions of telemedicine among health care professionals in China	- Patient-related Barriers (Frequency: Over 50 times):<br>Low acceptance and understanding of telemedicine.<br>Inability to participate without being physically present.<br>Insufficient medical patient information.<br><br>- Expert-related Barriers (Frequency: Over 50 times):<br>Difficulty in designating experts.<br>Limited options for experts.<br><br>- Equipment and Hardware (Frequency: More than 40 times):<br>Defective equipment needing improvement.<br><br>- Timeliness (Frequency: More than 40 times): Lack of timeliness in telemedicine services.<br><br>Cost and Charges (Frequency: More than 40 times):<br>High costs and inability to reimburse medical insurance.<br><br>Medical History and Medical Records (Frequency: More than 40 times):<br>Non-standard and incomplete medical records.<br><br>Promotion and Popularization (Frequency: More than 30 times):<br>Lack of awareness and coverage due to insufficient popularization.<br><br>Data Management (Frequency: More than 30 times):<br>Cumbersome data filling and uploading process.<br>Incomplete and irregular data.<br><br>Grassroots Medical Resources (Frequency: More than 30 times):<br>Insufficient resources at the grassroots level to implement expert recommendations or complete pre-consultation examinations.<br><br>Appointment Waiting (Frequency: 19–29 times):<br>Long waiting times for appointments.	Facilitators Influencing Satisfaction, Using Intention, and Willingness to Recommend Telemedicine<br><br>- Increase in Age of Medical Professionals (Significance: P = 0.025):Older medical professionals showed increased overall satisfaction with telemedicine.<br><br>- Service Duration (Significance: P = 0.026 for satisfaction, P = 0.017 for using intention, P = 0.040 for willingness to recommend):<br>Service durations ≤10 minutes significantly reduced overall satisfaction, using intention, and willingness to recommend.<br><br>Convenient Operation of the System (Significance: P = 0.005 for satisfaction, P = 0.003 for using intention, P = 0.001 for willingness to recommend): Very convenient system operation significantly improved attitude and evaluation of telemedicine.<br><br>- Inconvenient Operating System (Significance: P = 0.003): Inconvenient system operation reduced the willingness to participate in telemedicine.<br><br>- Punctuality of Services (Significance: P > 0.05): Improved punctuality of services increased satisfaction and willingness to recommend telemedicine.

PUBLICATION	TITLE	BARRIERS	FACILITATORS
			- Cost Subsidy Incentive Measures (Significance: P = 0.006 for satisfaction, P = 0.011 for using intention, P = 0.040 for willingness to recommend): Adoption of cost subsidy incentives positively impacted overall satisfaction, using intention, and willingness to recommend.<br><br>- Job Title Promotion (Significance: P = 0.030 for satisfaction, P = 0.001 for using intention, P = 0.004 for willingness to recommend): Job title promotion positively influenced overall satisfaction, using intention, and willingness to recommend telemedicine.<br><br>- Excellent Evaluation (Significance: P = 0.003): Positive evaluations had a significant impact on improving overall satisfaction.<br><br>- Mandatory Requirements Without Incentives (Significance: P = 0.037): Mandatory requirements without incentives reduced the willingness to recommend telemedicine to patients.
Macias 2022	Utilizing big data from electronic health records in pediatric clinical care	Themes<br>1: Ensuring data validity<br>2: Using a Hybrid of Paper-Based and EHR Orders and Documentation<br>3. Using Data to Drive Quality Improvement<br>4. Developing Clinical Decision Support Tools ; 1. Disparate data sources and confounding data definitions across entities using the data.<br><br>2. Resources necessary to fully convert to EHR.<br>Resistance to change among end-users.<br><br>3. Challenges for quality and safety teams in obtaining meaningful data.<br><br>4. Challenges in finding expertise for the development, implementation, and monitoring of CDS tools.	1. Sufficient governance structure to ensure appropriate data use, standardization of definitions, and data security.<br><br>2. Federal strategies endorsing EHR conversion.<br>Expanded uptake of EHR making conversions easier with greater knowledge of best practices.<br><br>3. Early integration of Information Technology personnel with quality improvement teams.<br>Utilization of predefined metrics to ensure

PUBLICATION	TITLE	BARRIERS	FACILITATORS
		5. Difficulty in gathering enough data for uncommon outcomes to draw insights and define best practices.<br><br>6. Slow adoption of statistically high-performing clinical prediction rules by providers.<br><br>7. Cultural acceptance issues regarding AI and machine-derived models versus human-derived models.<br><br>8. Heterogeneity in disease classes hindering the development of targeted therapies.<br>5. Analytics for Low Prevalence Outcomes<br>6. Uptake of Clinical Decision Rules<br>7. Adoption of AI- and ML-Derived CDS<br>8. Advancing Personalized Medicine	alignment in institutional priorities.<br><br>4. Engaging with EHR-specific, disease-specific national workgroups.<br>Exploring adoption in EHR decision support libraries.<br><br>5. Well-defined process and outcome measures determined a priori from reliable and valid data sources.<br>Participation in quality collaboratives where data are pooled from multiple sites.<br><br>6. Improving the utility, credibility, and usability of prediction rules within the EHR.<br><br>7. Incorporating providers and domain knowledge expertise in modeling and implementation strategies.<br>Maximizing transparency to the model's derivation methods.<br><br>8. Research focused on improving the classification of pediatric diseases.<br>Accelerating discovery through AI and machine learning.<br>Equipping hospital beds with continuous monitoring and data harvesting capabilities.
Madanian 2023	Patients' perspectives on digital health tools	Digital Literacy: Many patients, especially older adults, lack the skills and confidence to use digital health tools, worrying about device security and complexity. Education and support can help overcome this barrier.<br><br>Health Literacy: Low health literacy prevents patients from understanding medical information and effectively using patient portals. Simplifying information and providing support can mitigate this issue.<br><br>Privacy Concerns: Patients fear misuse of their health data by third parties, which deters them from using digital health tools. Ensuring robust data security and building trust can help address these concerns.	Patient Empowerment: Digital tools and patient portals enhance patient control, engagement, and participation in decision-making, improving relationships with healthcare providers and reducing frustration with technology.<br><br>Self-Management: Digital technologies help patients manage their health conditions more effectively, increasing awareness and motivation, especially when supported by good patient-clinician

PUBLICATION	TITLE	BARRIERS	FACILITATORS
			relationships and integrated online systems.<br><br>Personalisation: Tailoring digital health experiences to individual needs increases patient engagement and satisfaction. Personalized care involves patients more in their treatment decisions.<br><br>Personalisation Through Communication: Effective, personalized communication between patients and providers enhances engagement and motivation. Including patients in the design process of digital tools can improve their effectiveness.<br><br>Patient-Driven Solutions: Tools created by patients for managing health can be highly effective. Collaboration between patients, clinicians, and designers leads to better-tailored digital health solutions.
Mahapatra 2023	Translational Challenges of Implementing AI in Healthcare: Solutions and Opportunities	Lack of Trust in AI – Concerns from healthcare professionals and patients about the reliability and safety of AI.<br><br>Data Privacy and Security Issues – Regulatory constraints (e.g., GDPR, CCPA) make it difficult to access and share medical data.<br><br>Algorithmic Bias – AI models may perpetuate healthcare disparities due to biased training data.<br><br>Data Quality Issues – Healthcare data is often incomplete, inconsistent, or difficult to standardize for AI training.<br><br>Regulatory and Ethical Barriers – AI must undergo strict validation and approvals, slowing down deployment.<br><br>Resistance from Healthcare Providers – Many clinicians worry AI will replace human decision-making.<br><br>High Implementation Costs – AI solutions require significant financial and technical investment.	AI Governance – Establishing robust rules, processes, and requirements for AI development and implementation.<br><br>Training and Upskilling Healthcare Workers – Educating professionals on AI applications to enhance adoption and trust.<br><br>Integration with Existing Systems – Ensuring AI tools seamlessly work within Electronic Health Records (EHRs) and hospital workflows.<br><br>Privacy-Enhancing Technologies – Using data anonymization, federated learning, and encryption to protect patient information.<br><br>Explainable AI (XAI) – Making AI decisions transparent to build trust among

PUBLICATION	TITLE	BARRIERS	FACILITATORS
		Limited Standardization – Lack of universal AI guidelines complicates integration into hospitals.	clinicians and patients.<br><br>Collaboration Between AI Developers and Healthcare Professionals – Engaging clinicians in AI design to ensure alignment with real-world needs.<br><br>Scalability and Adaptability – Developing AI solutions that can be used across different healthcare settings.<br><br>Public Awareness and Patient Education – Informing patients about the benefits and safety of AI-driven healthcare.
Malavasi 2024	Validation of an IoT-based home system for integrated care services: a qualitative investigation involving older adults with multiple chronic health conditions		Themes:<br>Context, User, Intervention, Technology<br><br>Context: Experience with health & social services; social support & influence<br><br>User: Psychological aspects; technological expertise; autonomy<br><br>Intervention: Behavioural change strategies; social connectedness & health integration<br><br>Technology: Usability & realiability; aesthetics; expenditure; training; privacy & data sharing
Marco-Ruiz 2024	A multinational study on artificial intelligence adoption: Clinical implementers' perspectives	Technological Barriers (13 Studies):<br>Issues with technological literacy, confidence, access, and quality.<br>Lack of internet connectivity in residential and rural areas.<br><br>Provider-Level Barriers (6 Studies):<br>Hesitancy to adopt AI, change clinic workflows, insufficient training, and staffing shortages.<br>Physician licensing issues across different states.<br><br>Patient-Level Barriers (4 Studies):<br>Preference for in-person visits, low health literacy, and privacy concerns.	Promising but Immature AI Implementation (General Perception):<br>AI is seen as promising but still in early stages, with many implementations being pilot projects from academic settings.<br><br>Technological and Skills-Based Training (Multiple Studies):<br>Training for clinicians and patients is crucial for successful AI implementation.<br><br>Provision of Technological Devices

PUBLICATION	TITLE	BARRIERS	FACILITATORS
		Communication Barriers (4 Studies):<br>Challenges for patients with visual or auditory impairments and non-native language preferences.<br>Lack of integrated translators in telemedicine consultations.<br><br>Regulatory Complexity (General Perception):<br>Overlapping regulations and approval bodies create confusion and delays in AI implementation.<br><br>Data Access and Availability (General Perception):<br>Delays and challenges in accessing data due to regulatory approval processes and lack of updated, high-quality data for AI training.<br><br>Infrastructure and Resources (General Perception):<br>Outdated hospital IT infrastructure and lack of funding for testing and integrating AI solutions in clinical settings.<br>Need for more technical and human resources to develop AI beyond the prototype stage.<br><br>Legal and IP Challenges (General Perception):<br>Complex licensing structures, intellectual property rights, and data ownership issues.<br>High costs and time required for CE marking and certifying AI products.<br><br>Perception and Acceptance (General Perception):<br>Clinicians generally have a positive perception of AI, but clerical staff may see it as a threat to their jobs.<br>Patients often do not understand AI and may have misconceptions about its role in their treatment.<br><br>Human Factors and Engagement (General Perception):<br>Involving clinicians, legal experts, data scientists, and health managers in all stages of AI implementation is crucial.<br>Addressing the pressure on clinicians and ensuring AI interventions align with clinical and societal needs.	(Multiple Studies):<br>Providing necessary technological devices facilitates AI use.<br><br>Dedicated Support Teams (Multiple Studies):<br>Establishing dedicated telehealth workgroups and structured workflows supports AI implementation.<br><br>Reimbursement and Financial Support (Multiple Studies):<br>Financial incentives, such as reimbursement for telemedicine visits, are key to adoption.<br><br>Local Validation and Re-Training (General Perception):<br>AI systems must be validated and potentially re-trained with local data to ensure performance and relevance.<br><br>National Guidelines and Standard Evaluation (General Perception):<br>Unified guidelines and standard evaluation protocols are necessary for consistent and effective AI implementation.<br><br>Multicenter and Evidence-Based Evaluation (General Perception):<br>Conducting multicenter studies and evidence-based evaluations helps demonstrate AI systems' benefits across different settings.<br><br>Regulatory Framework Adaptation (General Perception):<br>Adapting regulatory frameworks to accommodate the dynamic nature of AI and provide clear guidance on data access and usage.

PUBLICATION	TITLE	BARRIERS	FACILITATORS
			National and Regional Coordination (General Perception):<br>Coordinating efforts at national and regional levels can streamline evaluation studies and centralize support.
Mathur 2023	Barriers and Solutions to Adoption of AI in Healthcare	Lack of Clinician Education – Limited understanding of AI concepts among healthcare professionals.<br><br>Trust Issues – Clinicians' skepticism due to lack of explainability, reliability, and transparency in AI models.<br><br>Reproducibility Concerns – AI models often fail to generalize across different populations and healthcare settings.<br>> (reliability, which refers to whether the AI technology can per-<br>form a task predictably and consistently, might be particularly concerning in healthcare<br>due to the changes in the reliability of AI in the presence of new data)<br><br>Unclear Value Proposition – Healthcare providers are hesitant to adopt AI without clear cost-effectiveness and clinical impact.<br><br>Human–Computer Interface (HCI) Challenges – AI systems are not seamlessly integrated into clinical workflows, making them difficult to use.<br><br>Bias and Ethical Concerns – AI models can perpetuate biases based on training data.<br><br>Regulatory and Legal Challenges – Lack of standardized guidelines for AI implementation.<br><br>Data Privacy and Security – Compliance with regulations like GDPR and HIPAA remains a major challenge.	AI Education for Clinicians – Introducing AI into medical school curricula and continuous professional development.<br><br>Building Trust in AI – Implementing explainable AI (XAI) and transparency in model decision-making.<br><br>Frameworks for Evaluation – Using DECIDE-AI and TEHAI frameworks to assess AI safety, reliability, and utility.<br><br>Demonstrating AI's Value – Conducting clinical trials and cost-effectiveness studies to prove AI's impact on healthcare outcomes.<br><br>Improving Human–Computer Interfaces – Prioritizing user-centered design and ensuring AI tools fit seamlessly into clinician workflows.<br><br>Addressing Bias and Ethical Issues – Developing fair AI models and improving dataset diversity.<br><br>Regulatory Alignment – Establishing standardized legal frameworks for AI use in healthcare.<br><br>Strengthening Data Privacy Protections – Using privacy-enhancing technologies like differential privacy and federated learning.
May 2021	Challenges in current nursing	Organizational and Administrative Efforts: The study identified that, prior to	Improved Communication: After the

PUBLICATION	TITLE	BARRIERS	FACILITATORS
	home care in rural Germany and how they can be reduced by telehealth - an exploratory qualitative pre-post study	implementation, the processes for care provision required additional organizational and administrative work. This contributed to inefficiencies in care delivery.<br><br>Interruptions in Daily Care Routine: Nurses experienced interruptions in their daily work routines, primarily due to the need for in-person consultations, which affected their workflow.<br><br>Delayed Treatments and Loss of Information: The diversity of communication methods (e.g., phone, fax) led to delays in treatment and a risk of losing patient-relevant information.<br><br>Initial Learning Curve: The implementation of video consultations required an initial effort to overcome the learning curve for using new technologies such as tablets and video software.	implementation of telehealth, communication between nursing home staff and physicians improved significantly. Video consultations reduced the risk of information loss and facilitated faster care delivery.<br><br>Time Savings: Telehealth saved time by reducing travel for physicians and documentation efforts for nurses. Physicians could conduct more consultations in the time saved from travel.<br><br>Increased Efficiency: The structured scheduling of video consultations helped create more reliable and focused appointments, improving care coordination.<br><br>Enhanced Patient Safety: Direct communication through video consultations helped avoid treatment errors and led to quicker delivery of medications and treatments.<br><br>Better Working Conditions: The study reported that telehealth improved the working conditions for healthcare providers by reducing unnecessary travel and improving coordination between staff.
Mbunge 2021	Sensors and healthcare 5.0: transformative shift in virtual care through emerging digital health technologies	- Data Heterogeneity and Fragmentation:<br>Different healthcare institutions use various database formats, making data unification and analysis difficult, impacting AI model training.<br><br>- Lack of Standardization and Interoperability:<br>Absence of standardized data formats hampers data sharing and tool integration across healthcare systems.<br><br>- Unreliable Network Connections:<br>Intermittent or non-existent Internet connections in developing countries hinder smart	

PUBLICATION	TITLE	BARRIERS	FACILITATORS
		healthcare adoption.<br><br>- Unreliable Sensor Data:<br>Lack of validation mechanisms for sensor data can lead to misdiagnosis, posing a risk to patient safety.<br><br>- Privacy and Data Security Concerns:<br>Concerns over data privacy and security breaches deter the use of cloud-based health services.<br><br>- Scalability Issues:<br>Blockchain-based tools face challenges in scaling to handle large volumes of health data.<br><br>- Sensor Health Consequences:<br>Sensitivity to misalignments and electromagnetic fields in sensors can limit their adoption in wearable robotics.<br><br>- Clinical Implementation Challenges:<br>Questions about the placement and use of biosensors, and potential device failures pose clinical adoption barriers.<br><br>- Health Hazards of Nanosensors:<br>Long-term exposure to nanodevices may alter body functions, accumulating in organs and blocking blood flow.<br><br>- Limited Computational Capabilities:<br>Nanodevices have short battery lives and limited computational power, hindering their practical use.<br><br>- Limited Memory Space:<br>Insufficient memory in nanodevices and sensors for real-time data storage poses a challenge.<br><br>- Rigid and Complex Models:<br>Rigid, complex digital models are not adaptable to changing needs and can lead to overfitting.<br><br>- Regulatory Framework Gaps:<br>Lack of regulatory frameworks for digital technologies delays approval and adoption.<br><br>- Inadequate IT Infrastructure:	

PUBLICATION	TITLE	BARRIERS	FACILITATORS
		Insufficient ICT infrastructure in developing countries hinders healthcare 5.0 adoption.<br><br>- Misalignment with E-Health Strategies:<br>Innovations not aligned with national e-health strategies face adoption challenges.<br><br>-High Implementation Costs:<br>Significant capital investment and skilled manpower requirements deter adoption in resource-constrained settings.	
Moll 2023	“It depends on the people!” – A qualitative analysis of contextual factors, prior to the implementation of digital health innovations for chronic condition management, in a German integrated care network	Technology Dimension:<br>- Lack of interoperability due to heterogeneous systems and software providers.<br>- Absence of legal requirements regarding interface standards leading to mixed media usage (paper and electronic documentation).<br>- High development costs and technical feasibility issues in creating integration interfaces.<br>- Low information quality caused by mixed media usage, affecting user satisfaction.<br>- Data protection concerns, particularly under existing regulations in Germany.<br><br>Human Dimension:<br>- Potential resistance from older colleagues still using paper-based structures.<br>- Initial reluctance to adopt new, unknown health platforms.<br>Necessity for flexibility in IT systems to accommodate different settings and tasks.<br>- Concerns over feasibility of implementing platforms under existing data protection requirements.<br><br>Organisational Dimension:<br>- Low degree of cooperation between inpatient and outpatient sectors.<br>- Varying communication channels between different healthcare sectors.<br>- Practice closures and low specialist density affecting continuity of care.<br>- Potential increase in workload and complexity in daily work routines.<br>- Risk of sectoral thinking among care providers limiting new cooperation forms.<br><br>Specific Topics in Relation to the ADLIFE Project<br>- Higher amount of work and complicated implementation processes.<br>- Complexity in using the platforms.<br>- Low willingness of patients to participate in the ADLIFE care concept.<br>- Limited digital skills among older patients possibly restricting platform use.	
Mosch 2022	Creation of an Evidence-Based Implementation Framework for Digital Health Technology in the Intensive Care Unit: Qualitative Study	Staff Involvement and Training:<br>Lack of responsibility among staff to apply the remote patient monitoring system.<br>Absence of a leading member in charge of implementation.<br>Inconsistent and insufficient training and information flow.<br>Negative peer pressure from opinion leaders.	Staff Engagement and Communication:<br>Persistent leadership engagement and nomination of responsible persons for implementation.<br>Continuous staff training, particularly in

PUBLICATION	TITLE	BARRIERS	FACILITATORS
		Additional Benefit:<br>Perceived low added value of the system due to existing monitoring solutions.<br>High staff presence in the ICU reducing the need for remote monitoring.<br>Increased workload due to frequent connecting and disconnecting of patients.<br>Impossibility of immediate reaction to alarms when away from the ward.<br><br>Intervention Features:<br>Limited number of vital parameters monitored.<br>Dependency on a stable wireless network connection.<br>Inconvenient size and usability of the tablet device.<br>Inability to monitor patients during transportation.<br><br>Attitude of Staff:<br>Satisfaction with the current monitoring system, leading to resistance to change.<br>Lack of habit and routine in using remote patient monitoring technology.<br>Fear of increased workload and reduced break times.<br>Concerns about reduced patient contact, false alarms, and patient safety.	the early stages of implementation.<br>High-quality instructions to influence staff opinion positively.<br>Feedback discussions with staff and project leaders to increase engagement.<br>Encouraging and motivating communication about the project.<br><br>Setting: Equipping all beds and staff members with portable monitoring devices.<br>Considering normal or intermediate care units for remote patient monitoring due to lower staff presence and technical facilities.<br>Monitoring patients with weaker indications for ICU admission in normal wards or IMCUs.<br>More straightforward implementation in wards with longer patient stays.<br><br>Intervention Features:<br>High intuitiveness of the technology.<br>A monitoring solution without cables to increase usability.<br>Device size that balances clear visualization and portability.<br>Software interoperability with other devices like respirators or PDMS.
Mouloudj 2023	Adopting artificial intelligence in healthcare: A narrative review	Knowledge and Expertise: Lack of digital expertise, insufficient knowledge, and poor digital leadership impede AI adoption. This is compounded by resistance to change and inflexible organizational cultures that are not conducive to digital transformation.<br><br>Funding and Costs: The high initial start-up costs, lack of funding, and quality of basic digital infrastructure present significant financial barriers. AI implementation is often seen as costly, and without adequate government financial support, these challenges are difficult to overcome.<br><br>Management Support: Lack of support from top management can hinder AI adoption. Organizations need to ensure strong backing from their leadership to drive AI initiatives effectively.	Perceived Benefit: Recognized as a strong predictor of AI adoption, particularly in fields like radiation oncology.<br><br>Evidence Strength and Design Quality: High-quality evidence and well-designed AI systems facilitate adoption, especially in decision support systems in emergency departments.<br><br>Accuracy, Privacy, and Security: Critical factors for transforming healthcare with AI, ensuring the technology is reliable

PUBLICATION	TITLE	BARRIERS	FACILITATORS
		Regulation and Legal Issues: Regulatory and legal challenges, including issues related to privacy, patient data confidentiality, and liability, pose significant obstacles. There is also a need for clear rules of accountability and comprehensive policies to guide AI integration.<br><br>Trust and Acceptance: Building trust among stakeholders, especially patients, is crucial. Concerns about privacy and information security must be addressed to reduce perceived risks. Additionally, there is a variance in acceptance and trust among direct adopters (e.g., radiologists) and indirect adopters (e.g., referring clinicians).<br><br>Technical Performance: Inconsistent technical performance of AI applications and compatibility issues with existing systems are major barriers. The performance and added value of AI for clinical practice need to be clearly demonstrated.<br><br>Data Issues: Insufficient size and quality of available data pools, as well as difficulties in data sharing, impede AI effectiveness. The opacity of AI algorithms further complicates trust and usability.<br><br>Training and Education: There is a need for specialized training for healthcare professionals to effectively use AI technologies. Without adequate training, the adoption process can be significantly slowed down.<br><br>Social and Ethical Obstacles: Societal norms and attitudes, lack of confidence in AI, and ethical concerns such as algorithmic fairness and biases, and the need for informed consent are substantial hurdles.<br><br>Organizational and Managerial Issues: Resistance to data sharing and the need for human interaction in care delivery are organizational barriers. Moreover, the integration process can be hampered by unstructured implementation and unclear visions and objectives.<br><br>Political and Policy-related Obstacles: A lack of political support and comprehensive policies that facilitate AI adoption can limit its implementation.<br><br>Performance Risk and Dependence: There are concerns about the performance risk and technology dependency associated with AI, including fears of AI replacing human work.<br><br>Cybersecurity: Protecting AI systems from cyber threats is critical. Ensuring the cybersecurity of AI applications is a legal challenge that needs addressing.<br><br>Intellectual Property: Intellectual property laws need to be clear and supportive of AI	and trustworthy.<br><br>Performance Expectancy and Effort Expectancy: These factors, along with initial trust, are important for AI adoption in healthcare.<br><br>Technological, Institutional, and Organizational Dimensions: Factors such as technology readiness, institutional support, organizational size, workflow, and training play key roles in adoption.<br><br>Management Support and Competitive Pressure: Strong support from top management and pressure from competitors can drive AI adoption.<br><br>Regulatory Compliance and Government Support: Favorable regulations and government incentives can significantly boost AI integration in healthcare.<br><br>Perceived Usefulness and Ease of Use: Positive perceptions about the usefulness and ease of using AI technologies encourage their adoption.<br><br>Innovation Strategies and Local Champions: Presence of hospital-wide innovation strategies and local champions to advocate for AI use facilitates adoption.<br><br>Education and Training: Ensuring healthcare professionals are well-trained in using AI technologies helps overcome barriers and encourages use.<br><br>Collaboration and Partnerships: Strategic partnerships and collaborations can enhance the adoption process by pooling

PUBLICATION	TITLE	BARRIERS	FACILITATORS
		innovations to protect the interests of developers and users alike	resources and expertise.<br><br>Data Transparency and Traceability: Clear and transparent data management practices are essential for building trust and confidence in AI systems.<br><br>Addressing Skills Gap: Investment in closing the skills gap among healthcare professionals through education and training is crucial.<br><br>Modernized Data and Analytic Infrastructure: Investing in updated data infrastructure and analytic procedures supports the effective use of AI.<br><br>Equity and Bias Consideration: Explicitly addressing issues of equity and bias in AI applications ensures fair and ethical use of the technology.
Muller 2023	Needs, expectations, facilitators, and barriers among insurance physicians related to the use of eHealth in their work: results of a survey	Main barriers were losing human interaction (54%) and security issues (51%). Younger IPs saw more options for using eHealth, compared to older IPs.	Main facilitators were that eHealth could make IPs' work more effectively and efficiently (61%) and<br>more future-proof (60%).
Nataliansyah 2022	Managing innovation: a qualitative study on the implementation of telehealth services in rural emergency departments	[Start-up Stage:]<br>-Strategies<br>-Capability<br>-Relationships<br>-Environment ; [Start-up Stage:]<br>-Incomplete needs assessment<br>-Limited service capacity<br>-Referral patterns limitation<br>-Politics and contextual issues<br>[Utilization Stage:]<br>-Training challenges<br>-Lack of utilization<br>-Lack of buy-in<br>-Workflow flaws<br>-Time consuming process	The domains strategies, capability, relationships, and environment played major roles in influencing implementation at all stages; [Start-up Stage:]<br>-Availability of needs assessment<br>-Experience and expertise<br>-Existing relationship<br>[Utilization Stage:]<br>-Intensive training<br>-Robust service capacity<br>-Close relationship<br>-Well-defined workflow<br>-Care coordination<br>[Sustainment Stage:]<br>-Opportunities

PUBLICATION	TITLE	BARRIERS	FACILITATORS
		-Lack of integration<br>[Sustainment Stage:]<br>-Poor fit with needed service<br>-Attrition<br>-Not a money maker<br>-Lack of evaluation measures<br>-Politics and contextual issues<br>[Utilization Stage:]<br>-Strategies<br>-Capability<br>-Relationships<br>-Protocols<br>-Service<br>Characteristics<br>-Environment<br>[Sustainment Stage:]<br>-Strategies<br>-Capability<br>-Financials<br>-Accountability<br>-Environment	-Availability of multi-specialities<br>-Referral patterns<br>-Payment model<br>-Evaluation and quality improvement ;<br>[Start-up Stage:]<br>-Strategies<br>-Capability<br>-Relationships<br>[Utilization Stage:]<br>-Strategies<br>-Capability<br>-Relationships<br>-Protocols<br>-Service Characteristics<br>-Financials<br>[Sustainment Stage:]<br>-Strategies<br>-Capability<br>-Financials<br>-Accountability<br>-Relationships
Natsiavas 2019	Citizen perspectives on cross-border eHealth data exchange: A European survey	-Lack of trust regarding the intentions of data collection<br>-Lack of suitable legislation<br>-The risks of interlinking these data with other personal information already available and traceable in the Internet (e.g. posts in social media platforms)	-A common legislation among EU Member States<br>-Better control of data management practices applied by companies<br>-More information on the processing of citizen health<br>data
NavarroMartínez 2023	Nurses’ view of benefits, enablers and constraints to the use of digital health tools with patients: A cross-sectional study	-Advanced age of patients<br>-Low cultural level of patients<br>-Institutional constraints<br>-Lack of training for professionals<br>-Patients do not have access to the Internet or devices	-Patient training<br>-User-friendliness<br>-Guidance by professionals<br>-Professional training<br>-Institutional support
Neher 2022	Perspectives of Policy Makers and Service Users concerning the Implementation of eHealth in Sweden: Interview Study	- Limitations in the capability of the service user<br>- eHealth is not always what the individual service user wants<br>- eHealth is perceived as time consuming for the service user<br>- Mismatch of technology with service user needs<br>- Perceived lack of data protection	- User-friendly design<br>- Matches skill set of service user<br>- Provides a sense of privacy<br>- Personal feedback and staff support<br>- Flexible use of time<br>- A credible sender
Nene 2023	Personalized Telehealth: Redesigning Complex Care Delivery for the 65+ during	- Patient cognitive impairment<br>- Patient sensory impairment (vision, hearing)<br>- Lack of privacy (ex: having a roommate)	- improved access to technology, including infrastructure and technological support

PUBLICATION	TITLE	BARRIERS	FACILITATORS
	the COVID Pandemic: a Survey of Patients, Caregivers, and Health-care Providers	- Patient advanced cognitive impairment<br>- Caregiver health limitations<br>- Televisits perceived as inferior to in-person visits<br>- Technology-related barriers (hardware and software)<br>- Patient not comfortable with technology<br>- Poor internet bandwidth<br><br>- technology use difficulties and lack of access to tools and guides (hardware and software)<br>- providing videoconference visits<br>- cost of technology<br>- lack of technology availability<br>- lack of administrative support<br>- Time It takes schedule an online appointment<br>- conflict with work schedule<br>- significant workload related to the process of implementing this technology	- administrative support for scheduling and set up<br>- creation of tools and guidelines to facilitate a safe and confidential process of conducting telehealth visits both for HCPs, and patients and their caregivers
Nezamdoust 2022	Adopting mobile health applications by nurses: a scoping review		Objectives of using mHealth applications:<br>1. Learning and knowledge enhancement<br>2. Treatment and improvement of the patient care process<br>3. Improve the diagnostic process<br>4. Data and patient management<br>5. Health promotion<br><br>Factors affecting the use of mHealth applications:<br>1. Ease of use<br>2. Usefulness<br>3. Security and confidentiality<br>4. Feasibility and functionality<br>5. Design and use-interface<br>6. Effectiveness<br>7. Infrastructure<br>8. Social norms and versatility
Ng 2022	Perception and Attitude of Malaysian Community Pharmacists Towards the Implementation of Telepharmacy	-Lack of coordination between different health sectors<br>-Inability to integrate telepharmacy systems with pharmacy information system<br>-Lack of awareness of telepharmacy<br>-Insufficient training of pharmacists and patients regarding use of telepharmacy<br>-Problems with telepharmacy license<br>-Complexity of telepharmacy for patients<br>-Government's lack of knowledge about telepharmacy	[Perceived Benefits towards Implementation of Telepharmacy:]<br>-Improve availability of health services<br>-Prevent unnecessary trips to access pharmacy services<br>-Improve the quality of health services<br>-Increase satisfaction of pharmacist and

PUBLICATION	TITLE	BARRIERS	FACILITATORS
		-Lack of access to IT infrastructure<br>-Complexity of health service delivery processes<br>-Lack of pharmacist-technical staff to provide telepharmacy services<br>-Privacy and confidentiality issues<br>-Time limitation and lack of time to use telepharmacy<br>-Complexity of telepharmacy for non-pharmacists<br>-Problems in payments (insurance and repayments)<br>-Patients’ resistance to use telepharmacy<br>-High cost of equipment and hardware needed for telepharmacy system<br>-High maintenance cost of telepharmacy system<br>-Complexity of telepharmacy for pharmacists<br>-Pharmacists’ resistance to use telepharmacy	patients<br>-Assist medical services and prevention of disease<br>-Improve efficient training of pharmacists<br>-Help the decision-making and detection of drug-drug interactions<br>-Reduce health service cost<br>-Help in chronic disease control<br>-Reduce medical errors ; [Attitude towards Implementation of Telepharmacy:]<br>Compatibility:<br>-Telepharmacy is completely compatible with my current situation<br>-Telepharmacy is compatible with pharmacy services available in the pharmacy<br>-I think telepharmacy fits well with the way I like to work<br>-Using telepharmacy fits well into my current work style<br><br>Trial ability:<br>-I believe to try telepharmacy applications is a great opportunity<br>-I would like to try out telepharmacy services in retail setting<br>-I believe using telepharmacy on a trial basis is enough to see what it could do<br>-I do not have to take much effort to try out telepharmacy]
Odendaal 2020	Health workers' perceptions and experiences of using mHealth technologies to deliver primary healthcare services: a qualitative evidence synthesis.	-Staff attrition<br>-Shortages<br>-Short-life batteries<br>-healthworkers' aptitude for mobile devices and their digital literacy<br>-health system arrangements<br>-high-level stewardship	-user-friendly software<br>-healthworkers' aptitude for mobile devices and their digital literacy<br>-health system arrangements<br>-high-level stewardship
Offermann 2023	Telemedicine in nursing homes: Insights on the social acceptance and ethical acceptability of telemedical consultations	General Aspects:<br>-Only one device per nursing facility<br>-Double documentation (missing connection)<br>-Double communication (after consultation)	Rapid Contact with Physicians:<br>Telemedicine allows for quicker access to physicians, reducing waiting times, which is particularly valuable during medical emergencies .

PUBLICATION	TITLE	BARRIERS	FACILITATORS
		Contact with Physicians:<br>-Uncertainty in reasons for contact (acute situation vs. emergency)<br>-Way of contacting (prefer direct instead of central)<br><br>Personnel-related Aspects:<br>-Lack of routine (need for training)<br><br>Residents-related Aspects:<br>-Distance to residents: (telemedical physicians have to get to know residents)<br>-Distance to residents with dementia (initial touches are missing)<br><br>Technical Aspects:<br>-Poor internet connection<br>-Low battery life (30 minutes)<br>-Cable tangle<br><br>Organizational Aspects<br>-Duration of the telemedical consultation too long	Increased Safety for Staff: The ability to share responsibility with telemedical physicians enhances the safety of nursing staff .<br><br>Avoiding Unnecessary Hospitalization: Telemedicine helps prevent the disorientation and health decline that can occur with hospitalization, benefiting residents .<br><br>Support of Care Personnel by Additional Staff: The involvement of non-physician practice assistants eases the workload for nursing staff .<br><br>Improved Technical Aspects: Good acoustic and visual quality of telemedicine systems support better communication with physicians .<br><br>Better Resident Care and Service: Telemedicine helps reduce waiting times for doctor visits, improving overall care quality .
Olawade 2023	Using artificial intelligence to improve public health: a narrative review	- legal concerns, e.g. data privacy and security<br>- ethical concerns, e.g. fairness and biases of algorithms<br>- absence of clear categorization and summarization of methods for predictive modeling<br>- challenges in finding high-quality data	- Data collaboration and sharing, e.g. federated learning<br>- Use of patient data from multiple centers<br>- Compliance with regulations, e.g. HIPAA (Health Insurance Portability and Accountability Act)
Olaye 2023	The Gap Between AI and Bedside: Participatory Workshop on the Barriers to the Integration, Translation, and Adoption of Digital Health Care and AI Startup Technology Into Clinical Practice	Knowledge on health care systems' technology procurement process:<br>-Lack of knowledge on health care systems' technology procurement protocols and best practices<br>-Limited access to best practices and strategies for successful technology procurement<br>-Venture funding leads more companies to sale directly to employers<br>-Lack of awareness on how to reach and educate providers on product offerings<br><br>Digital health innovations from large technology companies:<br>-Competing with large technology companies<br>-Lack of large marketing departments	Knowledge Sharing and Education: Continuing education opportunities for both digital health entrepreneurs and healthcare providers are critical for facilitating the integration of new technologies into clinical practice.<br><br>Partnerships and Networking: Facilitating interactions and relationship-building between early-stage companies, venture

PUBLICATION	TITLE	BARRIERS	FACILITATORS
		-Lack of broad network of connections in comparison to larger companies<br>-Lack of networking and financial resources in comparison to larger companies<br>-Disadvantages of early-stage digital health companies compared to large technology conglomerates<br><br>Demanding regulatory and validation requirements:<br>-Strenuous regulatory, validation, and technology evaluation evidence required from health care systems<br>-Lack of funding for randomized controlled trials<br>-Inappropriate existing study design to evaluate digital health innovations<br>-Inability to publish study results in academic journals and other peer review mediums due to proprietary concerns<br>-Lack of ability to explain AI algorithms<br><br>Success in health care systems' technology procurement:<br>-Limited information and uniformity on the health care procurement process<br>-Lengthy sales cycle<br>-Strenuous marketing and networking process<br>-Lack of transparency on who the decision maker is<br>-Lack of funding to attend conference trade shows<br>-Limited resources to support a health care pilot that demonstrates financial and clinical ROIb<br><br>Health care systems' technology procurement barriers by early-stage health care technology entrepreneurs:<br>-Challenges within the health care system technology procurement process<br>-Lack of bandwidth at health care systems to properly evaluate digital innovations	capitalists, healthcare providers, and regulators helps to address barriers in health technology procurement and implementation.<br><br>Improved Regulatory Processes: Streamlined and transparent regulatory and validation processes can support faster adoption of new digital health innovations.<br><br>Dedicated Health Technology Evaluation Roles: Creating roles within healthcare systems, such as Chief Information Officers and Chief Research Informatics Officers, to focus on evaluating digital innovations can aid in the faster and more effective adoption of new technologies.<br><br>Access to Funding: Ensuring that early-stage digital health companies have access to sufficient venture capital and other funding sources is a key facilitator for the development and integration of innovative technologies into clinical settings.
Olesen 2023	Usefulness of a Digitally Assisted Person-Centered Care Intervention: Qualitative Study of Patients’ and Nurses’ Experiences in a Long-term Perspective	Perspectives of nurses and patients:<br>-Conflicting perspectives on the ability of patients to engage with DA-GSD and how to provide it<br>-Conflicting perspectives on DA-GSD as a threat to the nurse-patient relationship<br>-Functionality of DA-GSD and available technical equipment<br>-Data security	Perspectives of nurses:<br>-Re-evaluation of the nurse-patient relationship<br>-Improved functionality of DA-GSD<br>-Supervision, experience, patient feedback, and a global pandemic
Osman 2019	Barriers and facilitators for implementation of electronic consultations (eConsult) to enhance access to specialist care: A scoping review	- Increased workload and workflow disruptions [Primary care provider (PCP)]<br>- Technical challenges to use eConsult [PCP]<br>- Loss of specialist contact [PCP]<br>- Unfamiliarity with using eConsult service [PCP]<br>- Insufficient remuneration to use eConsult [PCP]<br>- Challenges related to patient follow-up [PCP]<br>- Receiving timely responses from specialists [PCP]<br>- Increased workload [Specialist]	- PCPs receiving timely response from specialist [PCP]<br>- Building capacity and knowledge [PCP]<br>- Improved communication with PCPs [Specialist]<br>- Educational opportunities [Specialist]<br>- Improved referral efficiency [Specialist]<br>- Reduced time commitments required for

PUBLICATION	TITLE	BARRIERS	FACILITATORS
		- Concerns with liability [Specialist]<br>- Loss of patient contact [Specialist]<br>- Challenges with the quality/content of eConsult [Specialist]<br>- Challenges with the quality/content of eConsult [Specialist]<br>- Challenges with the use of technology [Specialist]<br>- Insufficient remuneration to use eConsult [Specialist]<br>- Some patients preference to see specialists face-to-face [Patient perspective]<br>- Perceived decrease in accessibility to specialist care [Patient perspective]<br>- Concerns about safety/appropriateness of eConsult [Patient perspective]<br>- eConsult system design challenges [Healthcare system]<br>- Lack of resources [Healthcare system]<br>- Variation in licensure requirements across provinces/states [Healthcare system]<br>- Privacy concerns [Healthcare system]<br>- Insufficient remuneration for providers [Cost]<br>- Provider payment structure (salaried physicians vs fee-for-servoce models) [Cost]	eConsult [Specialist]<br>- Ability to expedite face-to-face consultation if needed [Specialist]<br>- Remote residence location [Patient perspective]<br>- Timely access to specialist care [Patient perspective]<br>- Potential cost savings [Patient perspective]<br>- Acceptance of eConsult [Patient perspective]<br>- Increase provider knowledge capacity and confidence [Healthcare system]<br>- eConsult platform choice [Healthcare system]<br>- eConsult ease of use [Healthcare system]<br>- Improved access to specialist care [Healthcare system]<br>- Use of case manager to triage consultations [Healthcare system]<br>- Security measures [Healthcare measures]<br>- Improved quality of care/ "Safety net" effect [Healthcare system]<br>- Organisational commitment to implementation [Healthcare system]<br>- Clarifying providers' duty of care/role [Healthcare system]<br>- End of user engagement/consultation [Healthcare system]<br>- Providing ongoing support/training/evaluation [Healthcare system]<br>- Piloting eConsult [Healthcare system]<br>- Developing payment models and incentives for providers to use eConsult [Healthcare system]<br>- Potential cost savings for insurance payers to use eConsult [Cost]<br>- Potential cost savings for society [Cost]<br>- Potential cost savings for the healthcare

PUBLICATION	TITLE	BARRIERS	FACILITATORS
			system [Cost]
Otto 2019	Investigating barriers for the implementation of telemedicine initiatives: A systematic review of reviews	People-related barriers:<br>- Patient: individual characteristics, individual resources, patient expectations, social support, social interaction, usability of telemedicine<br>- Healthcare Provider: negative associations with technology use, social interaction, individual characteristics, usability, individual resources<br>- Culture [e.g. culturally inappropriate communication]<br>- Disease [e.g. special demands for group therapy]<br>Process-related barriers:<br>- Health sector: Integration of telemedicine into existing processes, Workforce [workforce shortage]<br>- Standards/ Guidelines: missing standradized protocols or procedures, missing guidelines<br>- Legal framework: regulatory issues, unclear responsibilities<br>- Finance: lack of funding, lack of reimbursement strategies, high costs, missing benefits<br>- Organisation: low accessibility, inadequate workforce, missing cooperation, lack of planning [e.g. strategy for scaling up]<br>- Methodology: missing clinical evaluation, missing proof of cost-effectiveness, lack of reliability<br>Object-related barriers:<br>- Technology: outcome expectations [e.g. missing functionalities], missing usability, missing interoperability, lack of human technical support, regional infrastructure [e.g. no granting of broadband access]	
Palombini 2023	Building a Framework for a More Inclusive Healthcare System	telehealth infrastructure and access:<br>-low socioeconomic status<br>-Access to Healthcare Services (rural communities, financial barriers, stable, long-distance transportation)<br>-lack of telehealth infrastructure<br>-quality of the broad band connections<br>-availability of the internet and telemedicine<br><br>digital literacy:<br>-Low digital literacy<br>-language barriers<br>-Complex user interfaces<br>-inattention to user experience<br>- different levels of education<br>-individuals challenged by physical or mental impairments that are barriers to access of virtual care health services<br>-majority of these services are designed for an educated and English-speaking audience	

PUBLICATION	TITLE	BARRIERS	FACILITATORS
		government regulations and telehealth legislation:<br>-region-specific jurisdictions that impeded the expansion of telehealth use<br>-physicians limited in their options to implement telehealth care<br>-limited monetary support<br>-Billing regulations limit the reach of telehealth benefits<br>-implementation of telehealth services is often limited to the legislation in site-specific regions	
Pan 2021	Perception and initial adoption of mobile health services of older adults in london: Mixed methods investigation	Study 1:<br>-lack of obvious advantage<br>-low reliability<br>-scary information<br>-risk of privacy leakage<br>-aging factors (generation gap)<br><br>Study 2:<br>-Access to a device<br>-Connection to internet<br>-Downloading<br>-Installing (Register)<br>-Sustained use	
Panda 2021	Perceptions of Mobile Health Technology in Elective Surgery: A Qualitative Study of North American Surgeons	-poor up-front instructions<br>-preference for face-to-face care<br>-a lack of motivation<br><br>[Supplemental Content:]<br>Challenges/Disincentives:<br>-Slow adoption of innovation<br>-Bandwidth<br>-Legal/regulatory concerns<br>-Surgeon attitude<br>-Reimbursement and finances	[Supplemental Content:]<br>-Buy-in<br>-Data support<br>-COVID-19<br>-EMR integration
Paranjape 2021	The Value of Artificial Intelligence in Laboratory Medicine		Requirements for Implementing AI:<br>- education: specific to device, AI short course training<br>- various prerequisites: support systems, certifications, evidence of benefits<br><br>Reasons why participants feel that AI will be valuable in their organisation within the next 5 years:<br>- organisational value (e.g. quicker

PUBLICATION	TITLE	BARRIERS	FACILITATORS
			results, reduced redundancy, resource management)<br>- quality of care (e.g. accessibility of care, accuracy, early recognition)<br><br>Reasons why participants feel that AI will not be valuable in their organisation within the next 5 years:<br>- prerequisites (e.g. budget and strategic plan)<br>- unsure attitude toward AI
Patterson 2022	Virtual care and the influence of a pandemic: Necessary policy shifts to drive digital innovation in healthcare	Systemic barriers:<br>-limited cellular network coverage<br>-internet bandwidth<br>-Poor interoperability between EHR systems<br>-Disturbing the status quo when adopting virtual care<br>-Patient privacy and personal health data are strongly protected<br>-remuneration for virtual care<br>-Interoperability with health information systems<br><br>Barriers for care providers:<br>-concerns about weakened therapeutic relationships and risks of fragmented care<br>-limiting the care (virtual visits did not enable them to apply a full range of diagnostic techniques during an examination)<br>-Additional time (responding to requests)<br>-concerned about protecting patient confidentiality during virtual visits<br>-possibility of legal liability in the event of security breaches<br>-Remuneration systems<br>-limited administrative and technological resources<br>-uncertainty about the quality of virtual care<br>-lack of previous experience<br><br>Barriers faced by patients:<br>-“digital divide” rooted in unequal skills and access to suitable technology<br>-concerns over data security and privacy	

PUBLICATION	TITLE	BARRIERS	FACILITATORS
		-loss of human contact during virtual visits<br>-lower income patients<br>-cognitive or physical<br>impairments	
Payán 2022	Telemedicine implementation and use in community health centers during COVID-19: Clinic personnel and patient perspectives	[Clinic-level:]<br>Personnel capacity:<br>- Negative impact of COVID-19 on operations<br><br>Professional development capacity:<br>-Lack of knowledge or uncertainty about appropriate use<br>-Reimbursement policy confusion<br><br>Technological capacity:<br>-Lack of private workspaces for personnel<br>- Limited equipment for patients in home settings<br>-Difficulty integrating a third-party language interpretation service<br><br>[Patient-level:]<br>Individual-level:<br>-Older age<br>-Limited English proficiency<br>-Limited digital literacy<br><br>Housing and the home environment:<br>- Lack of housing<br>-Lack of privacy in home settings<br><br>Technology:<br>-Lack of equipment or services<br><br>Interpersonal support and technical or language assistance:	Clinic-level:<br>-Personnel capacity<br>-Professional development capacity<br>-Technological capacity<br><br>Patient-level:<br>-Technology<br>-Interpersonal support and technical or language assistance
Pechtor 2023	Unravellling the processes and challenges of artificial intelligence implementation in the swiss public sector : A toe framework analysis	-Bureaucracy and Centralized Decisions<br>-Data Availability, Management, Capabilities and Privacy<br>-Infrastructure<br>-Lack of Strategy and Management Capabilities<br>-Resources and Funding<br>-Siloed Applications	Processes involved in AI adoption<br>-'-access to scalable infrastructure<br>Technical Factors:<br>- Case Handling Process<br>- Capability<br>- Infrastructure<br><br>Organisational Factors:

PUBLICATION	TITLE	BARRIERS	FACILITATORS
			- Top Management Support<br>- Change Management<br>- Innovation Process<br>- Data Management<br>- Talent Management<br>- Budgeting Process<br><br>Environmental Factors:<br>- Data Protection<br>- Ethics<br>- Public Opinion
Pohlmann 2020	Digitalizing health services by implementing a personal electronic health record in Germany: Qualitative analysis of fundamental prerequisites from the perspective of selected experts	Documentation standards:<br>-Highly segmented health care<br>-Mandate of the analog paper world<br>-Highly bureaucratic<br>-Strongly differentiated<br>-Various standards and terminologies<br><br>Interoperability:<br>-Federal structures<br>-Highly segmented health care<br>-Plurality of electronic systems<br>-Old business models<br>-Plurality of actors<br>-Semantic and technical interoperability<br><br>Political structure:<br>-Clear political will<br>-Incentive structure<br>-Rights and regulations<br>-Self-administration<br>-Different interests<br>-Strong advocacy groups<br>-Clear responsibilities<br>-Transparency	
Pomales-Ramos 2023	A mixed-methods examination of clinicians' perceived barriers to telehealth delivered applied behavior analysis	Technological barriers:<br>[Quantitative data:]<br>Technological barriers scale:<br>-Client's access to technology (e.g., computer, tablet, smart phone)<br>-Client's access to internet services<br>-Client's familiarity with technology or videoconferencing software<br>-Clinician's access to an encrypted computer, tablet, or smart phone	

PUBLICATION	TITLE	BARRIERS	FACILITATORS
		-Clinician's access to internet services<br>-Clinician's access to HIPAA compliant videoconferencing software<br>-Clinician comfort with technology or videoconferencing software<br>-Internet connectivity issues<br>[Qualitative data:]<br>Technological barriers theme:<br>-Internet connectivity issues<br>-Difficulty seeing or hearing child<br>-Access to technology<br><br>Client barriers:<br>[Quantitative data:]<br>Client barriers scale:<br>-Provide telehealth to clients who elope or bolt.<br>-Provide telehealth to clients who engage in challenging behaviors.<br>-Provide telehealth to clients in preverbal stage.<br>-Provide telehealth to clients who are in early stages of services<br>-Provide telehealth to clients who experience motor difficulties.<br>-Provide telehealth to clients who communicate using single words.<br>-Transition clients from in-person to telehealth services.<br>-Have a productive session over telehealth.<br>-Make session feel personal<br>-Establish rapport over telehealth.<br>[Qualitative data:]<br>Caregiver involvement theme:<br>-Distractions and competing demands<br>-Expectations and perceptions of services<br>Child Engagement theme:<br>-Difficulty maintaining attention<br>-Elope from session<br>-Age or language level<br>Implementing intervention strategies theme:<br>-Behavioral management skills<br>-Demonstrating strategies or coaching parents<br>-Prompting or reinforcing the child<br><br>Administrative barriers:<br>[Quantitative data:]<br>Administrative barriers scale:<br>-Access to research about telehealth services<br>-Completing intakes or on-boarding new clients<br>-Access to therapy or assessment materials (e.g., manuals)	

PUBLICATION	TITLE	BARRIERS	FACILITATORS
		-Remote record keeping and paperwork<br>-Privacy and confidentiality<br>-Establishing professional boundaries and self-disclosure<br>-Scheduling appointments with my clients<br>-Using an interpreter<br>-Cost of systems (e.g., software, technology)<br>-Providing families with materials (e.g., worksheets)<br>-Providing assessment reports<br>-Providing remote consenting for telehealth services<br>-Concerns about mandated reporting<br>-Reimbursement from insurance<br>-Liability insurance coverage for specific services provided<br>[Qualitative data:]<br>Administrative or logistical barriers theme:<br>-Administrative barriers related to lack of guidance<br>-Lack of resources	
Poon 2022	A qualitative research study of primary care physicians' views of telehealth in delivering postnatal care to women		Perceived Usefulness:<br>-Increased access and convenience<br>-Complementary to in-person consultation<br>-Specific advantages of telehealth<br>-Portal to provide validated and personalised medical advice<br>-Telehealth for selected group of mothers with intrinsic motivation<br>-Impetus during the Covid-19 pandemic<br><br>Perceived Ease of Use:<br>-Technology literacy and infrastructure-Virtual team-based care for post-natal mothers<br>-Balance workload and access to specialists for support<br>-Adequacy of Privacy and data security<br>-Financial sustainability in a pay-for-service primary healthcare system
Prendergast 2019	The barriers and facilitators for nurse educators using telehealth for education	Important factors when engaging in Telehealth:<br>-Negative impact on service change<br>-Negative impact on staff<br>-Low expectations of outcomes/needs<br>-Impersonal<br>-Security & confidentiality	Important factors when engaging in Telehealth:<br>-Integration into routine practice<br>-Trust in technology<br>-Collaboration<br>-Flexible & response to working practice

PUBLICATION	TITLE	BARRIERS	FACILITATORS
		-Poor change management<br>-Lack of time<br>-Not a strong practice culture<br>-Lack of training<br>-Lack of confidence in technology<br>-Concerns about user friendliness<br>-Collaboration<br>-Communication issues	-Strong leadership & local champions<br>-Training & support<br>-Easy-to-use, reliable equipment
Rabanifar 2022	Exploring Barriers to Implementing Telerehabilitation from experiences of managers, policymakers, and providers of rehabilitation services in Iran: A Qualitative Study	Insufficient infrastructure:<br>-Lack of specialized equipment<br>-Internet failure<br>-lack of trained specialized personnel<br>-insufficient facilities<br><br>Legal and moral hazards:<br>-patient information security<br>-endangering the patient's physical health<br>-social and psychological vulnerability<br>-negative family consequences<br><br>Lack of priority and insufficient determination:<br>- acceptance among beneficiaries<br>-considering TR as a complementarity approach<br>-poor cooperation between knowledge-based companies and equipment manufacturers<br>-having bigger problems, and the lack of priority for TR<br><br>Insufficient support from public and non-governmental organizations:<br>-insufficient government support<br>-lack of support from NGOs<br>-unclear legal issues<br>-weak insurance system<br>-being costly<br>-insufficient support for academic researchers<br><br>Poor knowledge of using equipment:<br>-Inability to work with devices<br>-the therapist's inability to provide appropriate treatment<br>-equipment failure and the need for ongoing support<br><br>Lack of knowledge and negative attitude:	

PUBLICATION	TITLE	BARRIERS	FACILITATORS
		-Lack of education and knowledge in benefactors<br>-different interpretation and lack of common language between patient, therapist, and technologist<br>-lack of patience and high expectations of the approach<br>-administrative bureaucracy<br>-unfamiliarity of personnel with equipment<br><br>Low capacity in comparison with face-to-face rehabilitation:<br>-Inability to accurately assess the patient<br>-reduced patient interaction and team approach<br>-unrealistic treatment environment<br>-uncertainty in the implementation process<br>-time-consuming<br>-limited to simple virtual and research methods	
Rabinowitz 2023	The telemedicine experience in primary care practices in the united states: Insights from practice leaders	The ease of telemedicine adoption depended on both patients' and providers' prior experience using virtual health platforms:<br>-scheduling volume of telemedicine visits<br>-without prior telemedicine<br><br>Regulation of telemedicine varied across states and differentially impacted the roll-out processes:<br>-growing restrictions related to visits, such as the annual preventive visit<br>-state boundaries<br><br>Unclear telemedicine visit triage rules:<br>-Uncertainty about valuable triage<br>-wasted appointments for complaints that clearly needed to be addressed in person<br>-staff availability<br>-limited physical examination	The ease of telemedicine adoption depended on both patients' and providers' prior experience using virtual health platforms:<br>-Patient training, particularly on portal use<br>-clinicians' comfort with telemedicine<br>-prior experience<br>-formal training<br><br>Unclear telemedicine visit triage rules:<br>-robust triage process
Raghavan 2021	Public health innovation through cloud adoption: A comparative analysis of drivers and barriers in Japan, South Korea, and Singapore	Contextual factors:<br>-Healthcare status<br>-Technology readiness<br><br>Organizational factors:<br>-Cost of adoption<br>-Technical expertise<br><br>Policy factors:<br>-Lack of data standards<br>-Strict data storage requirements<br>-Data privacy concerns<br>-Weak cybersecurity infrastructure	Contextual factors:<br>-Demographic and economic factors<br>-Nature of the healthcare system<br>-Technology readiness and innovation mindset<br><br>Policy factors:<br>-National policy frameworks that promote digitization of healthcare<br>-Cloud-specific policies (Data standardization, Data storage, Data privacy, Cybersecurity)<br>-Incentives for cloud adoption

PUBLICATION	TITLE	BARRIERS	FACILITATORS
			Human factors:
Ramachandran 2023	Identifying Challenges and Barriers in Wearable Medical Devices Adoption through Text Mining	Data related:<br>-Handling Big Data<br>-Risk of data loss<br>-Security and Communication of Data<br>-Interoperability of data from diverse heterogeneous devices<br>-Data Accuracy<br><br>Security and Privacy:<br>- Threat of Excessive Monitoring<br>-Security and Ownership<br>-Communication of Wireless Means<br>-Lack of Regulations<br><br>Sensor related:<br>-Customisable<br>-Embedded into Healthcare Ecosystem<br>-Placement<br>-Sensitivity and Accuracy<br><br>Individual/ Patient related:<br>-Convenience<br>-Acceptability<br>-Consent and Ethical Issues<br><br>Miscellaneous:<br>-Cost and Health Disparity<br>-Regulations<br>-Connectivity Issues<br>-Battery Life<br>-Issues related to Wireless Networks	
Ramdani 2020	Exploring the determinants of mobile health adoption by hospitals in China: Empirical study		Technological organizational and environmental determinants of mHealth adoption by hospitals:<br>-Perceived usefulness<br>-Perceived ease of use<br>-System compatibility<br>-System security<br>-Information technology infrastructure<br>-System reliability

PUBLICATION	TITLE	BARRIERS	FACILITATORS
			-Top management support<br>-Organizational readiness<br>-Hospital size<br>-Government policy<br>-External pressure
Ramos 2020	Ehealth in Spain: Evolution, current status and future prospects	"1. Digital Literacy: Older populations, who are the primary users of healthcare services, often lack the digital skills necessary to fully utilize eHealth services.<br>2.Interoperability Challenges: Differences in coding criteria, regional languages, and administrative boundaries between Spain's autonomous communities hinder the full integration of eHealth systems.<br>3.Concerns about Privacy and Security: Users and professionals still have concerns about the security of online health data, including fears of unethical or commercial misuse.<br>4.Professional Resistance: Some healthcare professionals are resistant to adopting new eHealth technologies due to increased workloads or unfamiliarity with ICT tools.<br>5.Unequal Technological Development: There is a disparity in the level of technological advancement between regions, leading to unequal access to eHealth services.<br>6.Inadequate Investment: A lack of sufficient investment in ICT and digital infrastructure limits the expansion and enhancement of eHealth services across Spain.<br>7.Complexity of Use: Some users find existing eHealth services too complex, especially when interacting with systems like Electronic Health Records or telemedicine platforms"	"1.Technological Evolution: Advances in ICT have provided increasingly efficient solutions for health management and service provision, making eHealth more accessible and effective.<br>2.Cost Reduction: eHealth services like online appointments and electronic prescriptions significantly reduce costs, such as administrative expenses, and improve efficiency in the healthcare system.<br>3.Efficiency and Effectiveness: Tools such as Electronic Health Records (EHRs) and telemedicine improve access to clinical data, reduce errors, and facilitate better management of pharmaceutical spending.<br>4.User Empowerment: eHealth promotes greater user control over health through services like online appointment booking, prescription renewals, and access to medical histories, contributing to patient empowerment.<br>5.Support from Healthcare Professionals: Professionals recognize that eHealth tools can simplify and facilitate their work, such as remote monitoring and telemedicine consultations.<br>6.Interoperability: Efforts are underway to enhance interoperability between regional healthcare systems, especially in areas such as electronic prescriptions and medical records.<br>7.Public Satisfaction: Surveys show a high level of satisfaction among users with services like telehealth and online appointment booking, indicating positive

PUBLICATION	TITLE	BARRIERS	FACILITATORS
			reception and acceptance.<br>"
Rauwerdink 2021	Successes of and lessons from the first joint ehealth program of the Dutch university hospitals: Evaluation study		[Success factors for eHealth development and implementation]<br>-Fulfill a need<br>-Outsource<br>-Communication<br>-Personnel
Reinhardt 2021	Non-use of telemedicine: A scoping review	Usability related problems:<br>- general usability problems<br>- problems with the uptake of interventions<br>- lack of support<br>- technical problems: general technical problems, problems with connectivity, synchronisation problems, lack of access, poor adaptation to interventions user's devices, other problems<br>- design-related problems: general problems, difficulties with the structure and navigation of the intervention, limited comprehensibility<br><br>Content related problems:<br>- framing-related problems: incomprehensible language, lack of personalisation<br>- problems related to conveyed information: false or inaccurate information, missing or superficial information, inadequate information frequency, annoying content<br>- missing or insufficient functions<br><br>Costs<br><br>Data security concerns<br><br>Organisational barriers:<br>- lack of time<br>- incompatibility of the intervention with everyday life<br>- forgetfulness<br><br>Lack of trust<br><br>Negative experiences:<br>- loss of contact with providers<br>- impaired communication<br>- no perceived benefit<br>- psychological burden<br>- too high effort<br>- other problems	

PUBLICATION	TITLE	BARRIERS	FACILITATORS
		Lack of information<br><br>External barriers<br><br>Personal factors:<br>- barriers due to sociodemographics: gender [both male and female], age [high age], low level of education other problems<br>- barriers due to health status: low burden of disease, high burden of disease, physical limitations [vision, hearing, mobility], lack of psychological resources, limited perceived vulnerability, other problems<br>- low perceived self-efficacy<br>- low technical literacy<br>- lack of access: internet, personal computer, other problems<br>- barriers related to the social environment<br><br>Attitudes towards telemedicine technologies:<br>- lack of motivation: general lack of motivation, no perceived health need, no interest or rejection of the intervention, rejection of behavioural change<br>- technology aversion<br>- other preferences: conventional solutions, alternative technical solutions<br>- negative outcome expectations: loss of contact with their provider, did not expect any improvement, additional burden, decrease in freedom and independence, miscommunication and wrong decision-making	
Rodrigues 2024	Barriers and facilitators of health professionals in adopting digital health-related tools for medication appropriateness: A systematic review	Technical barriers:<br>- need for training<br>- time consumed<br>- poor user interface and system design<br>- alert fatigue<br>- limited value of the alerts<br>- lack of connection with other medical software<br>- lack of customisability<br>- low specificity of the alerts<br>- lack of well-integrated functions in the system<br><br>User-related barriers:	Technical facilitators:<br>- ease of use and learning how to use<br>- support given to clinicians<br>- time saved<br>- useful reminders<br>- educational role<br>- reduction in medication errors<br>- training provided<br>- easy access to up-to-date information<br>- possibility to enhance medication safety<br>- pleasant presentation of data<br>- opportunity to update and complement knowledge

PUBLICATION	TITLE	BARRIERS	FACILITATORS
		- end-user unfamiliarity with the device and technology<br>- negative impact on the doctor–patient relationship<br>- lack of time<br>- lack of trust in the system<br>- loss of clinical autonomy<br>- reluctance to change<br>- difficulties navigating in the system<br>- disagreement with the clinical decision recommendations<br><br>Economic barriers:<br>- lack of funding and/or financial incentives<br><br>Organisational barriers:<br>- lack of internet<br>- limited access to computers and other devices<br>- medico-legal issues and liability<br><br>Patient-related barriers:<br>- willingness to change according to the medication priorities of the patient<br>- patient's cooperation in changing medication<br>- inconvenience to use the system for patients with multiple complaints	User-related facilitators:<br>- trust in the system<br>- commitment to use it<br>- opportunity for HCPs to reflect it in their practice<br>- agreement with the clinical decision recommendations<br>- familiarity with the system and the technology<br>- improvement of communication between HCPs<br>- system meeting expectations<br><br>Economic facilitators:<br>- improvement of cost-effectiveness<br>- financial incentives<br>- adequate budgeting<br><br>Organisational facilitators:<br>- possibility to accommodate practice workflow<br>- possibility of non-medical staff having access to the system<br>- willingness to invest greater resources in the future<br><br>Patient-related facilitators:<br>- improved quality of care provided to patients
Rohowsky 2023	Everybody hurts sometimes: perceptions of benefits and barriers in telemedical consultations	[Perceived barriers of telemedical consultations in acute situations:]<br>Relevant for patients:<br>-Impersonality<br>-Loss of time<br><br>Relevant for personnel:<br>-Complex usage<br>-No physical examination<br>-No overall impression of the patient’s status<br><br>General Disadvantages:	[Perceived benefits of telemedical consultations in acute situations:]<br>Relevant for patients:<br>-No waiting times<br>-Reducing stress<br>-Centered to the patient<br><br>Relevant for personnel:<br>-Better work efficiency<br>-Facilitating the work of medical personnel

PUBLICATION	TITLE	BARRIERS	FACILITATORS
		-Missing infrastructure<br>-Technical failure<br>-Missing technological understanding of personnel<br><br>[Perceived barriers of telemedical consultations with a general practitioner:]<br>Relevant for patients:<br>-Impersonality<br>-Invasion of privacy<br>-Too fast handling of the examination<br><br>Relevant for personnel:<br>-Complex usage<br>-No physical examination<br>-No overall impression of the patient's status<br><br>General Disadvantages:<br>-Missing infrastructure<br>-Technical failure<br>-Missing technological understanding of personnel	General Advantages:<br>-Time efficiency<br>-Avoiding unnecessary hospitalizations<br>-Saving resources<br>-Supplying rural areas<br><br>[Perceived benefits of telemedical consultations with a general practitioner:]<br>Relevant for patients:<br>-No waiting times<br>-Regular check-ups possible<br><br>Relevant for personnel:<br>-Flexibility of the physician<br>-No travel time for the physician<br><br>General Advantages:<br>-Low risks of infections<br>-Supplying rural areas
Roppelt 2023	Artificial intelligence in healthcare institutions: A systematic literature review on influencing factors		- macro-economic readiness: IT-infrastructure, supportive communities]<br>- technological readiness: multi-faceted value proposition, overcome algorithmic challenges, evidence-based application<br>- regulatory readiness: regulation, laws, political support<br>- organisational readiness: organisational strategy, organisational culture, characteristics of task, IT set-up<br>- user readiness: general [awareness, beliefs etc.], staff-related [training, threat to professional autonomy etc.]
Saxena 2022	Advancing digital technologies in healthcare	-High costs<br>-Interoperability of systems<br>-Coverage issues (especially in rural areas and in developing countries)<br>-Concerns about privacy and technological disruptions<br>-Inability of technology to allow the "human-ness" of provider contact to flow through the online interaction monitoring<br>-Initial costs for developing countries (investment in infrasturcture, connectivity,	

PUBLICATION	TITLE	BARRIERS	FACILITATORS
		upgrades)	
Scheibner 2021	Benefits, challenges, and contributors to success for national eHealth systems implementation: A scoping review	- Implementation challenges: conflicting stakeholder requirements, difficulty demonstrating benefits, financial issues, government, policy and political issues, broader implementation challenges<br>- legal and ethical challenges: concerns about privacy, research ethics, patient autonomy, medical liability<br>- data challenges: ensuring data availability, information quality, interoperability<br>- stakeholder engagement: lack of patient uptake, physicians refusing to use the systems [no trust, poor experiences, burnout], lack of access to benefits from using eHealth systems [geographically isolated areas and developing countries], communication issues<br>- software-related challenges: technical challenges with the systems themselves, security challenges	Benefits:<br>- improvement of the efficiency and effectiveness of healthcare coordination, processes and delivery<br>- improvement of the access and exchange of information and data<br>- improvement of the quality of care<br>- support of research and policy<br>- patient empowerment and engagement<br>- improvements to patient safety and data security<br>- reduction of costs<br>- better service monitoring<br>- generally increased ability to address challenges that emerge
Schouten 2022	Implementing artificial intelligence in clinical practice: a mixed-method study of barriers and facilitators	Intervention characteristics, relative advantage:<br>-Physicians do not believe AI has relative advantage over experienced healthcare professionals<br><br>Intervention characteristics, adaptability:<br>-adaptability is a barrier as many interviewed physicians tend to believe an algorithm would not be applicable to their patients, even when it is validated in their specific patient population<br><br>Inner setting, structural characteristics:<br>-physicians are highly conservative<br><br>Inner setting, tension for change:<br>-physicians did not feel the need for AI algorithms<br>-habit (used to doing things a certain way)<br><br>Worst case scenarios<br>-worry about adverse outcomes for patients, i.e., delayed or wrong diagnosis, suboptimal treatment, inappropriate discharge, or even death<br>-losing their job<br>lose the enjoyable aspects of the job<br>-become a ‘lazy’ physician	Intervention characteristics, evidence strength:<br>-evidence strength could facilitate AI implementation by enhancing trust (e.g. a comprehensive retrospective and prospective validation of an algorithm, a high impact study or a internationally published RCT)<br><br>Intervention characteristics, relative advantage:<br>-for the less experienced physicians, AI was thought to be beneficial (Less experienced physicians do not have the benefit of this pattern recognition due to less experience)<br><br>Intervention characteristics, trialability:<br>-trial-and-error phase would enhance trust<br><br>Inner setting, tension for change:<br>physicians did see potential value of using in AI algorithms in healthcare<br><br>Inner setting, compatibility:

PUBLICATION	TITLE	BARRIERS	FACILITATORS
			AI algorithms need to be compatible to existing workflows and decision-making and enhance/support this<br><br>Inner setting, access to knowledge and information:<br>-physicians felt that they would sooner use decision support provided by algorithms when they understand how the predictions are made
Schreiweis 2019	Barriers and facilitators to the implementation of eHealth services: Systematic literature analysis	[Barriers of eHealth usage among consumers identified in the first expert discussion at MIE 2015]:<br>Individual barriers:<br>-Cognitive barriers (Missing education for health professionals to use and promote eHealth; Capability to learn; Language, linguistic barriers; eHealth literacy; Ability to keep up with technology)<br>-Motivational barriers (Does it have value for me?; Elderly people with more time might see their doctor instead of using eHealth services; Unclear benefits)<br>-Accessibility barriers (Missing/bad information about existing eHealth services; Time to keep up with technology and learn new technology)<br>-Trust-related barriers<br><br>Environmental and organizational barriers:<br>-Financial issues (Problem with financing eHealth solutions)<br>-Political barriers (Proof of effectiveness & efficiency of eHealth?)<br>-Organizational barriers (Missing fit into organizational structures, incentives?)<br><br>Technical barriers:<br>-Unsuited services (Design does not fit to users' needs)<br>-Security concerns<br>-System language<br>-Missing support (Who to call for help?)<br>-Missing standards (for patient data & for data exchange)<br>-Missing system feedback: unclear benefits<br><br>[Barriers mentioned in the literature]:<br>-Limited exposure/knowledge of eHealth (eg, poor digital health literacy)<br>-Lack of necessary devices<br>-Problems with financing eHealth solutions<br>-Cognition<br>-Security	[Success factors for consumer-centric eHealth services identified in the second expert discussion at eHID]:<br>Individual success factors:<br>- Trust & control (Quality (serivce, data), Qualification, Transparency (provider, data))<br>-Collaboration (Multichannel access improves communication with provider)<br>-User experience (Popularity, Status (objects), Fun of using it)<br>-Facilitating research (Finding patterns in data, Visualization of data, "Broader picture", Data linkage, Finding partners)<br><br>Environmentral & organizational success factors:<br>-Flexible funding<br>-Health outcomes<br>-Policies for using generated data for research<br>-Competition<br>-Supporting laws & regulations<br><br>Technical success factors:<br>-Usability (Service easy to access, Ease of use)<br>-Standards<br>-Security<br>-Reliability of service

PUBLICATION	TITLE	BARRIERS	FACILITATORS
		-Motivation<br>-Accessibility<br>-Unsuited services, design does not fit users' needs<br>-Confidentiality<br>-Missing fit into organizational structures, incentives<br>-Added workload	[Facilitators mentioned in literature]:<br>-Ease of use<br>-Improves communication<br>-Motivation<br>-Integrated into care<br>-Involvement of all relevant stakeholders<br>-Availability of resources<br>-User-friendliness
Schroeder 2023	Enablers and inhibitors to the adoption of mHealth apps by patients – A qualitative analysis of German doctors' perspectives	Personal factors:<br>-Advocacy<br>-Personal attitudes towards technology<br>-Existing workload<br>-wondered whether the trusting relationship could be affected by less face-to-face interaction<br><br>Interoperability:<br>-concerned about their strong relationship with their patients if they would include a DiGA as treatment support	Facilitating conditions:<br>-operation and stability of DiGAs<br>-user experience<br>-benefits<br>-costs<br>-health insurers provide more intensive ; advertising and regular information to patients to reduce negative preconceptions; Impact on the doctor-patient relationship:<br>- Trust (trust of the patients towards their doctors)<br>- Motivation (doctors do not see it as their responsibility to motivate their patients)<br><br>Doctors' expectation of action outcomes from a behavioral perspective:<br>- Patient health literacy (doctors expect, that the use of mHealth can strenghten the health literacy of patients)<br>- Patients' adherence (improved medication adherence as a primary benefit of mHealth)<br><br>Interoperability:<br>-valuable addition to<br>their role as medical professionals
Schroeder 2024	What would it take to improve the uptake and utilisation of mHealth applications among older Australians? A qualitative study	-language challenges<br>-cost<br>-possible misinterpretation by the mHealth apps<br>-an app my not be able to specifically address the needs and requirements of the patient<br>-tedious if a health app would ask them every day how they are doing	[Benefits:]<br>-beneficial to use an app when there is a need, such as having a chronic condition<br>-use of an app to provide added value to their well-being<br>-reduction in the frequency of visits to the doctor (saving travel an time)

PUBLICATION	TITLE	BARRIERS	FACILITATORS
			-feel safe and well cared<br>-expect that the app provide more detailed information about the disease, symptoms, medication and contraindications ; '-Social influence through the doctor<br>- Trust<br>- Health self-efficacy<br>- Benefits
Serrano 2018	Analysis of Barriers to the Deployment of Health Information Systems: a Stakeholder Perspective	Strategy:<br>-Lack of investment in the area, funding problems<br>-High initial funding<br>-Unclear situation about who has to pay, hospital or regional authorities<br>-Lack of organisational to deal culture with these projects<br>-Healthcare sector has not been consider as an industry<br><br>Information Systems:<br>-Too many actors involved, difficulties to align and coordinate all of them<br>-A very fragmented system<br>-Lack of tradition to work in collaboration<br>Organisations are very complex<br>-Lack of integration with hospital information systems<br>-Lack of data integrity<br>-The area is still emergent<br>-Lack of robust commercial solutions<br>-Technology is still immature (Prototypes not full reliable and operative, do not reflect all the functionality needed)<br>-Healthcare professionals tend to be reluctant about technology<br><br>HC Professionals:<br>-Change Resistance in some healthcare professionals<br>-Nurses and primary doctors<br>-Healthcare professionals tend to be reluctant about technology<br>-Demands unpaid extra time for health professionals<br>-Lack of awareness about the potential use of telemedicine<br>-Increase the workload<br>-Have more active patients<br>-Involvement and motivation of healthcare professionals is low<br>-Lack of training for professionals<br>Professionals are not trained in new technologies<br>-Lack of integration with hospital information systems<br>-Too many actors involved, difficulties to align and coordinate all of them<br>-A very fragmented system	

PUBLICATION	TITLE	BARRIERS	FACILITATORS
		-Lack of tradition to work in collaboration<br>Organisations are very complex<br>-Lack of robust commercial solutions<br>-Technology is still immature (Prototypes not full reliable and operative, do not reflect all the functionality needed)<br>-The area is still emergent	
Seto 2019	Opportunities and challenges of telehealth in remote communities: Case study of the Yukon telehealth system	Specialists' Perceptions:<br>-specialists were already too busy and there was a lack of incentives to use telehealth<br>-specialists already had full schedules with wait lists for their clients<br>-difficulty of scheduling telehealth sessions between face-to-face consultations because of the timing<br>in their home location<br><br>Patients' Perceptions<br>-clients would want to use telehealth more often but were not aware that it was available	
Shabir 2022	The Barriers and Facilitators to the Use of Lifestyle Apps: A Systematic Review of Qualitative Studies.	- Non-Conducive Environment: Internal environment, external environment<br>- Poor Marketing & Branding<br>- Disengaging Content: Irrelevant content & features, demotivational content<br>- Controlling & Invasive Apps<br>- Accessibility Barriers: Structural Barriers, Barriers to access of app content	- Motivational aspects for the user: extrinsic motivation, intrinsic motivation<br>- Effective marketing and communication<br>- User-centered design & content: Personalisation, User Autonomy, Engaging Content<br>- Humanising Technology<br>- Increasing Accessibility to App & Content: Ease of use, Overcoming structural barriers
Shah 2021	Governing health data across changing contexts: A focus group study of citizen's views in England, Iceland, and Sweden	Awareness of health data crossing contextual boundaries:<br>-Lack of transparency about the purpose of the use or sharing of data<br>-The intentions of private organisations wanting to use patient data extracted from the healthcare system were a concern<br><br>Information provision and individual-level control:<br>-Not being informed was perceived negatively and affected willingness to share data<br>-lack of information facilitated mistrust and helplessness<br><br>Fairness in data use, representation and reciprocity:<br>-profiling<br>-risks posed by the potential continuous flow of their health data to commercial organisations	Awareness of health data crossing contextual boundaries:<br>-purpose of the use of data was the most prominent issue about data sharing<br><br>Moral expectations and obligations for future data sharing:<br>-medical data would be used for research and improving population health, then there was a moral duty to allow that data to be reused<br><br>Information provision and individual-level control:<br>-participants wanted to be part of the decision-making process for the reuse of

PUBLICATION	TITLE	BARRIERS	FACILITATORS
			their health data in and outside of healthcare<br>-information and clarity provided some sense of control for individuals<br>-clarity over data subject's rights<br><br>Oversight and accountability of health data re-use:<br>-data subjects should have responsibility to decide whether, and how to share data about themselves<br>-informed of future data use and profits made from health data<br>-centralised information and consenting system<br>-a system where they could consent to sharing their data for different purposes
Sharma 2023	Addressing the challenges of AI-based telemedicine: Best practices and lessons learned	[AI-enabled telemedicine:]<br>-Lack of access to technology<br>-Limited human resources<br>-Language and cultural barriers<br>-Data privacy and security concerns<br>-Limited financial resources<br><br>[Telepsychiatry and the role of AI:]<br>-privacy<br>-security concerns<br>-regulatory barriers<br>-necessity for adequate training and education for providers<br><br>[Ethical concerns with implementing AI in tele-ICU:]<br>-relying too heavily on technology and neglecting the importance of human expertise and judgment<br>-risk of bias in the AI algorithms, which could lead to disparities in care for certain patient populations	[Tele-intensive care unit and the role of AI:]<br>-robust training datasets and ongoing validation<br>-transparency and explainability in the utilization of AI<br><br>[Ethical concerns with implementing AI in tele-ICU:]<br>-involve a diverse group of experts in the development and implementation of AI algorithms<br>-regularly evaluate the algorithms for accuracy, bias, and transparency<br>-provide training and education to clinicians on the proper use and interpretation of AI outputs to ensure they are making informed decisions<br>-transparency in the development, implementation, and use of AI in tele-ICU is critical to ensuring that patients and clinicians understand how the technology is being used and can trust its capabilities
Shear 2023	Experts' Perspectives on Use of Fast Healthcare	EHR Implementation Variation – Different healthcare institutions use EHR systems differently, causing integration issues.	EHR Implementation Variation – Different healthcare institutions use EHR

PUBLICATION	TITLE	BARRIERS	FACILITATORS
	Interoperable Resources for Computerized Clinical Decision Support	Limited EHR Vendor Support – Not all EHR vendors fully support FHIR or implement the latest versions.<br><br>Ontology Variation – Differences in medical terminology standards (e.g., ICD-10, SNOMED CT) make data exchange inconsistent.<br><br>Workforce Knowledge Gap – Few professionals have expertise in both FHIR and CCDS integration.<br><br>Limited Data for Testing – Lack of high-quality clinical test datasets for evaluating AI-driven CCDS.	systems differently, causing integration issues.<br><br>Limited EHR Vendor Support – Not all EHR vendors fully support FHIR or implement the latest versions.<br><br>Ontology Variation – Differences in medical terminology standards (e.g., ICD-10, SNOMED CT) make data exchange inconsistent.<br><br>Workforce Knowledge Gap – Few professionals have expertise in both FHIR and CCDS integration.<br><br>Limited Data for Testing – Lack of high-quality clinical test datasets for evaluating AI-driven CCDS.
Shinners 2023	Healthcare professionals' experiences and perceptions of artificial intelligence in regional and rural health districts in Australia	Perceptions of implementation barriers:<br>-research funding<br>-Clinical governance<br>-Interdisciplinary collaboration<br>-Organisation support<br>-Workforce knowledge<br>-Cost to implement<br>-Interoperability<br>-Infrastructure	Understanding of AI:<br>-lack of education and clear understanding<br><br>Education needs and barriers to implementation:<br>-Workforce knowledge about AI was found to be the biggest barrier to AI implementation<br>-cost<br>-organisational support<br>-interoperability<br>-infrastructure
Shull 2019	Digital health and the state of interoperable electronic health records	[Obstacles:]<br>-Cost<br>-Main technical issue with arriving at interoperability is the huge variation in semantics and coding standards<br>-Privacy Issues<br>-Analysis of Progress	[Viewpoint on Best Practices:<br>Standards:<br>-Adopt international standards such as FHIR, LOINC, and SNOMED CT and introduce these standards starting in medical school and university informatics classes<br><br>Education and Awareness:<br>-top-down approach

PUBLICATION	TITLE	BARRIERS	FACILITATORS
			-information technology professionals should be aware of how reimbursement works<br><br>Privacy:<br>-education on privacy<br>and cybersecurity issues]
Singh 2020	Current challenges and barriers to real-world artificial intelligence adoption for the healthcare system, provider, and the patient	Challenge for Healthcare Organisations:<br>- liability<br>- identifying and assessing leading AI vendors<br>- lack of established AI suppliers [companies may have limited understanding of how to apply AI's abilities to healthcare needs]<br>- AI offerings may lack features [e.g. interoperability]<br>- narrow clinical utility of AI products [because of regulatory considerations]<br>- lack of transparency of AI algorithms ["black box"]<br>- lack of financial reimbursement models for AI<br>- high technical complexity compared to previous technical innovations<br>- lack of knowledgeable personnel/skills gap<br>- lack of resources (e.g., financial, time, expertise) for development and implementation of AI systems<br>- limited datasets [e.g., small numbers, biased demographics]<br>- regulatory limitations [patient privacy, data sharing]<br>- conflicting company culture [due to lack of understanding, unaligned business goals, questioning the value of AI]<br>- lack of clarity about what successful AI implementation looks like<br><br>Challenge for Healthcare Providers:<br>- existing struggles with other technologies (e.g. EHR)<br>- limited time and mental capacity<br>- concerns regarding bias (racial, ethnic, gender and other sociodemographic characteristics)<br>- challenges in adapting medical schools curricula [teaching approaches, time]<br><br>Challenges for Patients:<br>- lack of trust in data safety and security<br>- lack of human interaction	
Singh 2021	Exploring the perspectives of primary care providers on use of the electronic Patient Reported Outcomes tool to support goal-oriented care: a qualitative study		Influencing factors<br><br>Perceived usefulness:<br>- usage behavior aligned with providers' typical approach to care<br>- Impact and value of ePRO

PUBLICATION	TITLE	BARRIERS	FACILITATORS
			- Alignment with existing workflow may influence usage behaviour<br><br>Behavioral intention:<br>- High behavioral intention<br>- Low behavioral intention<br>- Behavioral intention changed over time<br><br>Improving usage behavior:<br>- External factors may influence usage behavior: discipline, program, organization's culture research trial, patients' perceptions<br>- Perceived ease of use may influence usage behavior: technology issues, ease to use, training provided and comfort-level, learning curve
Singh 2023	Technological paradoxes and artificial intelligence implementation in healthcare. An application of paradox theory	-Privacy and trust issues<br>-Unsuitable for critical illnesses<br>-Lack of training and education<br>-Financial constraints<br>-Behavioural resistance	- Ease of use<br>- Effectiveness of automation<br>- accurate diagnosis<br>- cost efficiency
SinhaGregory 2023	The feasibility, acceptability, and usability of telehealth visits	-Patient lack of access to needed technology, technical challenges<br>-Poor internet connectivity<br>-Poor audio/visual quality<br>-Provider had technical problems<br>-inefficient or challenging system design (e.g., patient unable to log in or connect audio)<br>-lack of reliable internet as well as comfort with technology<br>-patients with low technical skills also experienced challenges with the telehealth visits<br>-challenging for older patients	(patients and general)<br>-successful implementation of telehealth for all patient populations requires telehealth systems to be designed for ease of use for patients with low technology skills and low technology efficacy<br>-success also requires that patients have access to the internet, appropriate technology, adequate training, and technical support.<br>-user-centered design approach that involves participation in the design process from all user groups including both patients and provider<br><br>-(providers):<br><br>-broader access to patients/enhanced patient ability to access care<br>-Cost savings and convenience (e.g., no

PUBLICATION	TITLE	BARRIERS	FACILITATORS
			need for patient to take off work; reduced need to travel; ease of scheduling)<br>-Improved visit adherence<br>-Facilitates ability to stay connected with patients<br>-ease of communication, and ability to fulfil patient<br>needs and appointment goals especially for health management tasks such as reviewing labs, monitoring, and follow-up visits.
Smirnova 2021	Adoption and use of health-related mobile applications: A qualitative study with experienced users	-Perceived Risk of Personal Data<br>-Time-consumption<br>-limited understanding of Health Data<br>-adaption to New Routines	-Price Value<br>- Simplicity<br>-Personalisation<br>-Guidance and Progress based on Data<br>-Flexibility<br>-Social Encounters<br>-trigger for a healthier lifestyle<br>- relevant statistic in app for own health data (e.g. burnt calories)<br>- perceived satisfaction with first health app
SolbergCarlsson 2023	Rapid implementation of remote digital primary care in Stockholm and implications for further system-wide implementation: practitioner's and manager's experience of the Always Open mobile application.	The adopter system:<br>- no systematic implementation strategy to enable take up and use of AO<br><br>The organization:<br>-knowledge of some managers and personnel about their<br>own organization (SLSO) was too low<br>-organization had yet to decide how to triage and refer patients effectively via the app and using their HLM Online unit<br>-the digitalized way of delivering and<br>coordinating care might increase costs for units<br>- new management challenges<br>- creative teamwork is more difficult to conduct remotely<br><br>The wider system:<br>-development is hindered by legal requirements<br>- accessible and<br>equitable care for all may be hindered because AO<br>requires the person to use digital identification<br>-patients and society<br>"stuck" on the traditional idea of physically	The adopter system:<br>-standardize certain procedures in digital care pathways involving different units<br>-learn how to improve their patient-education skills for digital visits<br><br>The organization:<br>-SLSO's technological readiness for AO was generally described in the groups as high, with adequate inter-<br>net connectivity and hardware<br>-high willingness of unit personnel to work with the technology<br>-importance of organized<br>practical training, as well as practical manuals, to minimize problems<br><br>Embedding and adaptation over time:<br>- combining digital and physical visits as part of the treatment plan

PUBLICATION	TITLE	BARRIERS	FACILITATORS
		visiting one's family physician<br>-unfair competition between units	
Sonawane 2023	The application of artificial intelligence: perceptions from healthcare professionals	- technical adaption challenges<br>- different protocol & policy change<br>- loss of managerial control<br>- social impact and implication<br>- informed consent and patient autonomy<br>- data privacy and security<br>- professional responsibility and accountability<br>- limited data availability<br>- regulatory challenges<br>- bias and ethic concerns<br>- lack of transparency<br>- integration with existing system	- AI will reduce nursing work loads, facilitate more efficient patient monitoring, improve risk detection, and enhance customer services
Sony 2023	Critical Success Factors for Successful Implementation of Healthcare 4.0: A Literature Review and Future Research Agenda		- Digital Integration and Interconnectedness of the Healthcare System [platforms used by different stakeholders are digitally connected, data sharing]<br>- Human centric Automation of Healthcare Providers [automated patient-system interfaces incorporating voice, gesture etc., intelligent sensing and monitoring of patient needs and health status]<br>- Improve Patient-Centricity and the Patient Experience [patients controls the flow of information, enabling them to make decisions]<br>- Use Big Data and Analytics [IoT, medical cyber-physical systems, data analytics used in patient care]<br>- Managing Digital Healthcare Supply Chains [use of digital technologies and data analytics to optimize the flow of healthcare products and services]<br>- Strategies for Promoting H 4.0 [building a technical infrastructure, improve the highly skilled manpower in these smart healthcare systems etc.]<br>- Promote a Culture for H 4.0 [visible aspects, values and beliefs, deeper shared

PUBLICATION	TITLE	BARRIERS	FACILITATORS
			assumptions]<br>- Healthcare Leadership [ability to identify priorities, provide strategic direction to multiple actors and create commitment]<br>- Healthcare Employees' Skills [e.g. big data analytics]<br>- Adoption of New Business Models [platform-based business models]
Sousa 2019	Digital and innovation policies in the health sector	A lack of:<br>-budget availability<br>- information availability<br>- knowledge of methods<br>- qualified human resources	-budget availability<br>- information availability<br>- knowledge of methods<br>- qualified human resources<br><br>-focus on creating more measures for education on digital<br><br>-CME – Continuing Medical education<br>-Medical Guidelines<br>-Scientific articles<br>- Clinical studies<br>- News about the industry and related topics<br>- Literature citation database - Congresses calendar, agendas, and proceedings<br>- Medical education videos<br>- Clinical trial data<br>- Products specifications<br>- Peer-to-peer portals and information sharing libraries<br>- Product specifications
Stasevych 2023	Innovative Robotic Technologies and Artificial Intelligence in Pharmacy and Medicine: Paving the Way for the Future of Health Care—A Review		- collaboration among stakeholders<br>- continuous investment in research and development<br>- creating appropriate regulatory frameworks
Steinhauser 2020	The Relative Role of Digital Complementary Assets and Regulation in Discontinuous Telemedicine Innovation in	-regulatory hindrances<br>-legal barriers<br>- IT infrastructure<br>- innovations may not be conform with existing business models and hinder profit	- financial support e.g. by the government<br>-digital technologies that present complementary assets can facilitate incumbents’

PUBLICATION	TITLE	BARRIERS	FACILITATORS
	European Hospitals		adaptation to digital transformation by contributing to their organizational agility<br>-regulation can facilitate the adoption of discontinuous innovation by<br>reducing uncertainty and information asymmetries<br>-regulation can<br>promote digital innovations by facilitating knowledge deployment and by providing subsidies, standards, and an innovation directive<br>-relevant digital complementary assets encompass health<br>information technology (HIT) applications<br>-digital complementary assets facilitate the use of telemedicine, and increase the value of its output<br>-In addition, IT employees can be complementary assets as well<br>-Policy makers can facilitate the creation of digital complementary assets and infrastructure<br>by providing education and training, defining crucial complementary assets, and incentivizing their acquisition<br>-managers may stimulate the accumulation of specialized digital complementary assets for these innovations<br>- adequate IT infrastructure for the adoption<br>-enables organizations to<br>deliver state-of-the-art services and to remain competitive
Støme 2021	Enabling guidelines for the adoption of eHealth solutions: Scoping review	- technological illiteracy of patients and providers<br>- lack of knowledge of patients and providers<br>- lack of awareness of patients and providers<br>- lack of access of patients and providers to the technology	Guidelines for implementation:<br>- Data management in the Integration of eHealth [efficient data transfer, security]<br>- User Adaptations to eHealth Solutions [user involvement, usability, adherence, user training programs]<br>- Evaluation and Scaling of eHealth

PUBLICATION	TITLE	BARRIERS	FACILITATORS
			Solutions [early-stage evaluation, marketing, practice community]
Sumner 2023	Artificial intelligence in physical rehabilitation: A systematic review	Standalone app-based systems:<br>- low technology literacy<br>- inability to know if an exercise has been done<br>- demands on the battery life of personal devices<br>- not considering the context for exercise recommendations (e.g. weather conditions)<br><br>Robotics to replace functions (e.g. prosthesis):<br>- fit and choice of prosthesis material [can negatively impact electrode contact, performance and durability]<br>- technical difficulties<br>- lack of portability and independent set-up<br>- requirement of ongoing calibration<br>- low accuracy of the system<br>- fatigue from overuse<br><br>Gaming systems:<br>- latency issues (causing motion sickness)<br>- gaming fatigue<br>- unclear visuals<br>- not enough personalisation (e.g., background music, bespoke avatars)<br><br>Activity monitoring using wearables:<br>- limited types of available sensors [what can be measured]<br>- inconvenience<br>- discomfort<br>- reduced battery life<br>- issues with connectivity<br>- poor compliance with wearing the devices<br>- technical inability to capture if an exercise is performed accurately	Standalone app-based systems:<br>- accessibility of the system<br>- ease of use<br>- ability to personalise treatment trough the app<br>- integration with an already established messenger app<br>- data security (data capture on patients' phone)<br><br>Robotics to replace functions (e.g. prosthesis):<br>- functional training on use of prosthesis<br>- gradually increasing the degrees of freedom [supports the transition from a direct control prosthesis to a machine learning controlled prosthesis]<br><br>Robotics to restore function (robotic orthosis with EMG sensors, intelligent treadmill):<br>- usability: clarity of instructions, ease of use, comfort, appearance, simplicity of training, effectiveness, overall satisfaction<br>- adaptive gait-pattern<br><br>Gaming system:<br>- low cost<br>- usability<br>- availability "off the shelf"<br>- flexibility<br>- customisation<br>- ability to remotely manage patients and assess their progress [for clinicians]<br>- appropriate difficulty level<br>- engaging game-based exercises<br>- ease of use<br>- gesture and voice recognition for interacting with gaming systems

PUBLICATION	TITLE	BARRIERS	FACILITATORS
			Activity monitoring using wearables:<br>- portability<br>- convenience<br>- comfort<br>- low cost
Sundstrom 2019	"People are struggling in this area:" a qualitative study of women's perspectives of telehealth in rural South Carolina	-issues of confidentiality in a small town, community-based telehealth (afraid of seeing someone you know)<br>-discomfort with mediated communication<br>-privacy concerns with telehealth infrastructure<br>-importance<br>of relationship-centered care, including patient-provider communication<br>-approachability of health care providers<br>-health care provider credibility, trust-<br>worthiness, and respectfulness when using telehealth	- lower costs using telehealth for patients (saving on gas etc.)<br>-decreased wait time for an appointment<br>- a telehealth program would address major transportation barriers
Tabaeeian 2022	A systematic review of telemedicine systems use barriers: primary health care providers' perspective	Data quality barrier:<br>- Data accuracy problem: wrong data input; blurred images, poor picture or sounds; unreadable data, unclear information, understandable data format, Repeat checking and comparing data, Uncertain that data extent are correct<br>- Data timeline issues: Lack of updating data, Lack of timely retrieval of data<br>- Data conciseness concern: Too many information limit interpretation, Information overload, Quantity of data that would have to be<br>processed<br>- Lack of data uniqueness: Duplication of data entry, Repetitive content<br><br>System quality barrier:<br>- System complexity in terms of usability: Lack of system user-friendliness, Difficult of use, Difficulty with the system in filling form fields, System is too elaborate, Lack of access easier to data/system, Technology is not simplified, Access involved an additional log-on<br>- Lack of system reliability: Lack of faithful representation, No feedback, Lack of sharing data through software, Report technical problem in system, System impossibility of filtering unnecessary<br>appointments, Limitations in the functionality for managing<br>different list, App disruptions, Unsatisfactory of accuracy of the system, High error margins and deviations in system, System inability to provide relevant information, Ineffectiveness of telemedicine<br>- Security and privacy concern: It is unclear who has what information about a user, Concern about confidently issue, Lack of information governance, Security concerns about their routine use of<br>communications channel<br>- System integration issue: Multiple platform in use, Problem in joined up between sectors, Communication across the sectors appeared	

PUBLICATION	TITLE	BARRIERS	FACILITATORS
		incoherent, Troublesome in not directly linked to the other workstations in the centers, Lack of interoperability between system, Incoherent communication across the sectors<br>- Inflexibility of systems-in-use: System incompatibility with routine health care, The system did not fit into the existing workflow, Lack of system adaptability/rigidity, Difficult to connect to other tasks, System interrupt workflow, Could not use their full abilities via telemedicine system, Lack of systems interoperability, Changing between different programs, System is time consuming<br><br>Service quality barrier:<br>- service reliability concern: Lack of timely channels of communication, Poor broadband coverage, Network dropouts, Lack of long–term service sustainability, Poor infrastructure, Inconsistent internet access<br>- Lack of technical support: Ambiguous or incompatible policy, Lack of guidance, Poor support by the information technology staff, Lack of coordinating structure<br>- Lack of technical support: Ambiguous or incompatible policy, Lack of guidance, Poor support by the information technology staff, Lack of coordinating structure<br>- Lack of user training: Lack of awareness of the practical processes, Lack of skills, Unfamiliar with new system, User inability in using system	
Taboada 2021	Implementing Goal Mama: Barriers and Facilitators to Introducing Mobile Health Technology in a Public Health Nurse Home-Visiting Program.	-Designing for mobile needs versus desktop functionality (functionality)<br>-Limited tech and workflow integration strained PHN capacity<br>-some sites had strict firewalls, lack of access to iCloud accounts, or organizational policies restricting the downloading of apps onto work phones (technological issues)<br>-PHNs need additional support to feel confident using new technology<br>-Client reluctance and mixed perceptions about goal setting affected adoption	-Technology helps to meet clients where they are and provides new insights<br>- technology allowed for communication of topics or needs that may be uncomfortable to broach in person<br>-Features provide value, skill-building, and social support<br>-Supervisor modelling supports PHN platform use
Temsah 2023	ChatGPT and the Future of Digital Health: A Study on Healthcare Workers' Perceptions and Expectations	-concerns about credibility<br>-source of information<br>-lack of credibility/Unknown source of information of data in the AI Model<br>-Worry of harmful or wrong medical decisions recommendations<br>-not available in my setting<br>-AI Chatbots are not yet well-developed<br>-Medicolegal implications of using AI for Patients care<br>-Not knowing which AI model can be used in healthcare<br>- unfamiliarity with AI Chatbots<br>- Worry about patients confidentiality<br>-resistance to adopt AI Chatbot in medical decisions	

PUBLICATION	TITLE	BARRIERS	FACILITATORS
		-worry of AI taking over human role in healthcare practice<br>-lack of personalized care and inability to adopt to prognostic factors	
Temsah 2023	Healthcare's New Horizon With ChatGPT's Voice and Vision Capabilities: A Leap Beyond Text.	- suboptimal audio quality<br>- ensuring data security necessitate<br>cautious integration in medical practice<br>- Complex medical images may be misinterpreted<br>- Security and privacy of<br>sensitive medical images need to be ensured<br>- Users might not correctly highlight the areas, leading to misinterpretation<br>-The drawing tool's accuracy and ease of use are crucial for effective communication<br>- Limitations in understanding complex or ambiguous medical images<br>- Ensuring the model's interpretations are accurate and reliable is crucial<br>- Suboptimal audio may lead to miscommunication<br>- Security of voice data<br>and prevention of impersonation or fraud is necessary<br>-Accuracy of translation in medical terminology is vital<br>- It may not be<br>proficient in all languages or dialects, leading to potential misunderstandings<br>- the system should not inaccurately analyze or make misleading statements about images<br>- Privacy concerns regarding individuals appearing in the<br>background of images need to be addressed<br>- Users must be cautious while relying on the model for medical topics and should always seek professional verification<br>- With expanded access, managing and monitoring the "responsible" use of the technology becomes imperative	
Teng 2022	Virtual Care Adoption - Challenges and Opportunities From the Lens of Academic Primary Care Practitioners	- structural barriers of the health system<br>- clinical barriers<br>-patient-centered challenges ; '- legal challenges related to state licensure and practice laws, credentialing<br>- liability concerns<br>- telehealth service reimbursement<br>- costs of telehealth infrastructure<br><br>- development of the patient-provider relationship,<br>- concerns about standard of care<br>- informed consent,<br>privacy, and security<br><br>- access to technology<br>- reliable broadband,	

PUBLICATION	TITLE	BARRIERS	FACILITATORS
		- patients' ability to use the technology<br>- loss of<br>connectivity.<br><br>- Lose context of in-person visit<br>- more difficult to obtain vitals<br>- Loss of team-based care<br>- language barriers/hearing impaired<br>- patient think it is not at real visit<br>- difficult to assess patient privacy before conducting visit<br>-Internet/phone outages<br>-Need specific<br>equipment (iPhone<br>or iPad that is not<br>supplied)<br>-Geriatric and other<br>populations do not<br>have or know how to<br>use technology<br>-Patient lack of<br>phone/computer/<br>Internet access<br>-More difficult to obtain<br>consent<br><br>-No family members<br>included in visits to<br>give provider<br>additional information<br>-Released from jail—hard<br>to connect with<br>services<br>-Lack of housing<br>-People are chattier/<br>hard to end visit<br>-Change fatigue for<br>providers<br>-PCPs are in caregiver<br>role and have to go<br>back to work with no<br>childcare—no<br>accommodations	

PUBLICATION	TITLE	BARRIERS	FACILITATORS
		-Reimbursement for telehealth is changing<br>-Quality indicators are more difficulty to extract<br>-Providers unclear on how to bill<br>-Video visits are better for reimbursement, but many patients prefer phone	
Terry 2022	Is primary health care ready for artificial intelligence? Stakeholder perspectives: Worth the risk as long as you do it well	Mismatch between envisioned AI uses and the current reality, concerns over the impact on clinical skills, the risk of AI making mistakes,<br><br>ethical, legal, and social implications,<br><br>lack of transparency (black-box AI), and potential biases in AI algorithms.	Co-creation of AI tools with end users<br><br>availability and use of high-quality data<br><br>rigorous evaluation of AI tools, and strong evidence-based assessments.
Těšinová 2023	Development of telemedicine in the Czech Republic from patients' and other key stakeholders' perspective	- legislative environment<br>- Guidelines<br>- Technology and applications<br>- communication and data sharing<br>- organization of care and conditions of provision<br>- electronic pharmacy ;<br>- reimbursement of telemedicine solution<br>- Education of healthcare professionals<br>- Patient education and awareness<br>- Prevention and health promotion	- legislative environment<br>- Guidelines<br>- Technology and applications<br>- communication and data sharing<br>- organization of care and conditions of provision<br>- electronic pharmacy<br>- reimbursement of telemedicine solution<br>- Education of healthcare professionals<br>- Patient education and awareness<br>- Prevention and health promotion
ThomassenEEK 2023	Willingness, perceived facilitators and barriers to use remote care among healthcare professionals - a cross-sectional study.	- mistrust in the technological aspects of remote care and that healthcare<br>- professionals preferred conducting a physical examination of the patients.	[Open-ended facilitators and barriers:]<br>- technological equipment<br>- eligible patients<br>- user-friendly software<br>- adequate training ; '- effective technological solutions<br>- high level of willingness to use remote care<br>- belief that patients in remission or with low disease activity may need less hospital visits<br>-patients would prefer remote care

PUBLICATION	TITLE	BARRIERS	FACILITATORS
			- remote care is widely adopted by co-workers<br><br>- work flow<br>- integrated with electronic health record
Tierney 2023	Telemedicine Implementation for Safety Net Populations: A Systematic Review	- billing/administrative workflow disruption<br>- lack of quality broadband access<br>- low patient acceptance/preference for in-person care<br>- clinical workflow disruption<br>- lack of technical/implementation expertise<br>- lack of interpretation or translation of service<br>- lack of regulatory support<br>- low patient digital literacy<br>- clinician/staff training and resource requirements<br>- privacy concerns<br>- safety/quality of care concerns<br>- challenges due to lack of in-person communication for mental health services (sharing information, assessing a patient's physical state, establishing rapport with patients)	- efficiency gains<br>- patient acceptance<br>- enhanced patient access<br>- inexpensive/more cost-effective compared with in-person appointments<br>- availability of training for staff/clinicians<br>- reimbursement/payment<br>- reduced patient transportation and childcare needs<br>- reduced missed appointment rates (especially among pregnant and postpartum patients)
Tomasella 2021	“Sometimes I don’t have a pulse … and I’m still alive!” Interviews with healthcare professionals to explore their experiences of and views on population-based digital health technologies	- Concerns associated with overall reliability of the tech (validity, accuracy, calibration)<br>- Patients’/HCPs’ digital literacy and mindsets<br>- Costs, inequality, lack of resources and infrastructure<br>- Fast obsolescence of the tech<br>- lack of evidence of effectiveness for clinical implementation	[Suggested solutions to overcome disadvantages and barriers:]<br>- Interventions on DHTs for official and trusted<br>reliability<br>- Interventions on HCPs and patients to facilitate the<br>understanding of rationale and purpose of DHTs<br>- Data security solutions<br>- Infrastructure solutions
Tossaint-Schoenmakers 2021	The Challenge of Integrating eHealth Into Health Care: Systematic Literature Review of the Donabedian Model of Structure, Process, and Outcome	Technical Issues:<br>Technical problems disrupted workflows and made eHealth tools harder for both healthcare professionals and patients to use, leading to dropouts and non-adherence.<br><br>Inflexibility of Technology:<br>Many eHealth systems weren’t adaptable to clinical workflows, reducing efficiency and complicating integration with face-to-face care.<br><br>High Workload and Time Constraints:<br>The additional burden of eHealth tools increased workloads, with insufficient time and training discouraging professionals from fully adopting the technology.	Ease of Use:<br>Easy-to-use eHealth systems encouraged higher engagement from both providers and patients, improving adherence.<br><br>Supportive Organizational Structure:<br>Clear processes and strong organizational support made eHealth integration smoother.<br><br>Personalized Care:<br>Tailored eHealth solutions that addressed

PUBLICATION	TITLE	BARRIERS	FACILITATORS
		Lack of Feedback and Process Management:<br>Poor feedback on eHealth outcomes and unclear process guidelines left healthcare professionals uncertain about the value of eHealth tools.	patient-specific needs improved engagement and satisfaction.<br><br>Effective Communication:<br>Strong communication between providers and patients via eHealth platforms helped with smoother integration and better engagement.
Totten 2022	Telehealth-guided provider-to-provider communication to improve rural health: A systematic review	- Access to Knowledge & Information (Access to digestible information and knowledge about the innovation and how to incorporate it into work tasks)<br>- Networks & Communication (Nature and quality of webs of social networks, and the nature and quality of formal and informal communications within an organization)<br>- Engaging (Attracting and involving appropriate individuals in the implementation and use of the innovation through a combined strategy of social marketing, education, role modelling, training, and other similar activities)<br>- Available Resources (Level of resources organizational dedicated for implementation and on-going operations including physical space and time)<br>- Leadership engagement (Commitment, involvement, and accountability of leaders and managers with the implementation of the innovation)<br>- Patient Needs & Resources (Extent to which the needs of those served by the organization (e.g. patients), as well as barriers and facilitators to meet those needs, are accurately known and prioritized by the organization)<br>- Reflecting & Evaluating (quantitative and qualitative feedback about the progress and quality of implementation accompanied with regular personal and team debriefing about progress and experience)<br>- Planning (Degree to which a scheme or method of behavior and tasks for implementing an innovation are developed in advance, and the quality of those schemes or methods)<br>- Readiness for Implementation (Tangible and immediate indicators of organizational commitment to its decision to implement an innovation)<br>- Adaptability (Degree to which an innovation can be adapted, tailored, refined, or reinvented to meet local needs)<br>- Cost (Costs of the innovation and costs associated with implementing the innovation including investment, supply, and opportunity costs)<br>- External Policy & Incentives (External strategies to spread innovations including	- Formally Appointed Internal Implementation Leaders (Individuals from within the organization who have been formally appointed with responsibility for implementing an innovation as coordinator, project manager, team leader, or other similar role)<br>- Access to Knowledge & Information (Access to digestible information and knowledge about the innovation and how to incorporate it into work tasks)<br>- Networks & Communication (Nature and quality of webs of social networks, and the nature and quality of formal and informal communications within an organization)<br>- Engaging (Attracting and involving appropriate individuals in the implementation and use of the innovation through a combined strategy of social marketing, education, role modelling, training, and other similar activities)<br>- Available Resources (Level of resources organizational dedicated for implementation and on-going operations including physical space and time)<br>- Leadership engagement (Commitment, involvement, and accountability of leaders and managers with the implementation of the innovation)<br>- Patient Needs & Resources (Extent to

PUBLICATION	TITLE	BARRIERS	FACILITATORS
		policy and regulations (governmental or other central entity), external mandates, recommendations and guidelines, pay-for-performance, collaboratives, and public or benchmark reporting)<br>- Implementation Climate (Absorptive capacity for change, shared receptivity of involved individuals to an innovation, and the extent to which use of that innovation will be rewarded, supported, and expected within their organization)<br>- Compatibility (degree of tangible fit between meaning and values attached to the innovation by involved individuals, how those align with individuals' own norms, values, and perceived risks and needs, and how the innovation fits with existing workflows and systems)<br>- Executing (Carrying out or accomplishing the implementation according to plan)<br>- Relative Priority (Individuals' shared perception of the importance of the implementation within the organization)<br>- Knowledge & Beliefs about the intervention (Individuals' attitudes toward and value placed on the innovation, as well as familiarity with facts, truths, and principles related to the innovation)<br>- Complexity (Perceived difficulty of the innovation, reflected by duration, scope, radicalness, disruptiveness, centrality, and intricacy and number of steps required to implement)	which the needs of those served by the organization (e.g. patients), as well as barriers and facilitators to meet those needs, are accurately known and prioritized by the organization)<br>- Reflecting & Evaluating (quantitative and qualitative feedback about the progress and quality of implementation accompanied with regular personal and team debriefing about progress and experience)<br>- Planning (Degree to which a scheme or method of behavior and tasks for implementing an innovation are developed in advance, and the quality of those schemes or methods)<br>- Readiness for Implementation (Tangible and immediate indicators of organizational commitment to its decision to implement an innovation)<br>- Adaptability (Degree to which an innovation can be adapted, tailored, refined, or reinvented to meet local needs)<br>- Cost (Costs of the innovation and costs associated with implementing the innovation including investment, supply, and opportunity costs)<br>- External Policy & Incentives (External strategies to spread innovations including policy and regulations (governmental or other central entity), external mandates, recommendations and guidelines, pay-for-performance, collaboratives, and public or benchmark reporting)<br>- Implementation Climate (Absorptive capacity for change, shared receptivity of involved individuals to an innovation, and the extent to

PUBLICATION	TITLE	BARRIERS	FACILITATORS
			which use of that innovation will be rewarded, supported, and expected within their organization)<br>- Compatibility (degree of tangible fit between meaning and<br>values attached to the innovation by involved individuals, how those align with individuals' own norms, values, and perceived risks and<br>needs, and how the innovation fits with existing workflows and systems)<br>- Executing (Carrying out or accomplishing the implementation according to plan)<br>- Relative Priority (Individuals' shared perception of the importance of the implementation within the organization)<br>- Knowledge & Beliefs about the intervention (Individuals'<br>attitudes toward and value placed on the innovation, as well as familiarity with facts, truths, and principles related to the innovation)<br>- Complexity (Perceived difficulty of the innovation, reflected by duration, scope, radicalness, disruptiveness, centrality, and intricacy and<br>number of steps required to implement)
Traube 2021	Strategies for implementation of virtual home visitation in the United States	- busy family schedule	-telepractice services are intended to mitigate barriers<br>to service, including transportation, cost, and access to local services<br><br>[strategies to engage families to USC telehealth programme:]<br>- regular contact with families<br>- multiple forms of communication<br>-predictable schedule<br>- providing information material<br>-having a tangible metric where families could monitor growth was<br>essential

PUBLICATION	TITLE	BARRIERS	FACILITATORS
			- communication about benefit of program<br>- cost free programme<br>- active listening
Tully 2021	Barriers and Facilitators for Implementing Paediatric Telemedicine: Rapid Review of User Perspectives	Barriers to initiating the use of telemedicine:<br>- Lack of (clinical need) [Healthcare provider perspective]<br>- Billing/reimbursement issues [Healthcare provider perspective]<br>- Concerns about medico-legal ramifications [Healthcare provider perspective]<br>- Lack of trust in telemedicine accuracy [Healthcare provider perspective]<br>- Lack of direct patient contact [Healthcare provider perspective]<br>- Cost (of implementation) [Healthcare provider perspective]<br>- Time constraints [Healthcare provider perspective]<br>- Insufficient training in relevant specialty [Healthcare provider perspective]<br>- Inexperience with the equipment [Healthcare provider perspective]<br>- Patient privacy concerns [Healthcare provider perspective]<br>- Ease of access in the catheterization laboratory [Healthcare provider perspective]<br>- Image quality [Healthcare provider perspective]<br>- Trust of advisor (technology for communication with mentors) [Healthcare provider perspective]<br>- Lack of trust towards telemedicine tools [Patient/family perspective]<br>- Fear of excessive responsibilities for the family [Patient/family perspective]<br>- Child too sick to take part [Patient/family perspective]<br><br>Challenges encountered during use of telemedicine:<br>- process concerns [Healthcare provider perspective]<br>- technology concerns [Healthcare provider perspective]<br>- poor image quality [Healthcare provider perspective]<br>- Patient movement leading to blurred images [Healthcare provider perspective]<br>- Inability to perform necessary examination/treatment remotely (potential reason for incomplete visit) [Healthcare provider perspective]<br>- Billing/reimbursement issues [Healthcare provider perspective]<br>- Further test or imaging needed (potential reason for incomplete visit) [Healthcare provider perspective]<br>- Child site or parent decision prevented clinician from seeing child (potential reason for incomplete visit) [Healthcare provider perspective]<br>- Technical failure/inadequacy (potential reason for incomplete visit) [Healthcare provider perspective]<br>- Designated clinicians for tele-consultations out of office without cover (potential reason for cancelled/refused visit) [Healthcare provider perspective]<br>- Practise indicated being too busy to accommodate tele-visit (potential reason for cancelled/refused visit) [Healthcare provider perspective]<br>- Insurance did not cover telemedicine/no insurance (potential reason for	Benefits:<br>- Time savings<br>- Increased efficiency<br>- Convenience<br>- Lower cost<br>- Increased communication/familiarity/solidarity between staff/services<br>- Improved workflow/patient management/protocols<br>- Increased learning opportunities<br>- Improved enjoyment of visits for paediatric patients<br>- Reassurance (for professional or patient)<br>- Reduced stress<br>- Reduced risk of infection

PUBLICATION	TITLE	BARRIERS	FACILITATORS
		cancelled/refused visit) [Healthcare provider perspective]<br>- Visit request to late (potential reason for cancelled/refused visit) [Healthcare provider perspective]<br>- Administrative error/issue unrelated to the technology (potential reason for cancelled/refused visit) [Healthcare provider perspective]<br>- Practise unable to complete visit within available time (potential reason for cancelled/refused visit) [Healthcare provider perspective]<br>- Practise refused visit due to unpaid bill (potential reason for cancelled/refused visit) [Healthcare provider perspective]<br>- Parent picked up child before information capture was complete (potential reason for abandoned visits) [Healthcare provider perspective]<br>- Unable to acquire necessary information (potential reason for abandoned visits) [Healthcare provider perspective]<br>- Administrative problem (potential reason for abandoned visits) [Healthcare provider perspective]<br>- Technical problem (potential reason for abandoned visits) [Healthcare provider perspective]<br>- Problem was beyond capacity of model (potential reason for abandoned visits) [Healthcare provider perspective]<br>- Other (potential reason for abandoned visits) [Healthcare provider perspective]<br>- Encountered inadequate imaging to provide advice [Healthcare provider perspective]<br>- Temporary disruptions in audio and video quality requiring widening bandwidth of the internet provider [Healthcare provider perspective]<br>- Perception that telemedicine examination was insufficient [Patient/family perspective]<br>- Child distracted/bothered by screen [Patient/family perspective]<br>- sub-optimal audio/video [Patient/family perspective]<br>- Connectivity issues due to capacity of home internet service [Patient/family perspective]<br>- Not optimised for tablet PC [Patient/family perspective]<br>- Insufficient troubleshooting resources for families [Patient/family perspective]<br>- Telemedicine calendar not open early enough to find available slots [Patient/family perspective]<br>- Administrative burden [Patient/family perspective]<br>- No sign interpreter [Patient/family perspective]	
Twamley 2022	Exploring the perceptions of former ICU patients and clinical staff on barriers and facilitators to the implementation of virtual reality exposure therapy: A	'- psychological<br>- sensory<br>- environmental and resource-related<br>- staff competency and confidence ; - VR can be isolating<br>- VR side effects such as dizziness<br>- no space for VR	- interventions led by psychologist<br>- enthusiastic staff about VR<br>- staffs willingness to undertake training<br>- need for accessible support before, during and after the intervention<br>- The concept of VR as a tool for

PUBLICATION	TITLE	BARRIERS	FACILITATORS
	qualitative study	- staff felt unprepared and unqualified to deliver exposure therapy<br>- individuals competence using VR technology	exposure therapy was received ; '- staff training/education<br>- patient assessment/capacity<br>- Introduction/orientation to technology<br>- support during the intervention positively by patients, however, orientation and training were required to facilitate its implementation.
Valeur 2021	Patient rationales against the use of patient-accessible electronic health records: Qualitative study	[rationales against the use of Pasientjournal:]<br>- Unnecessary: No Need to Access the Health Records<br>- Impersonal: Prefer Dialogue to Information (information received in dialogue with health care workers was “good enough”/better)<br>- Incomprehensible: Patients Are Not the Intended Reader of the Medical Records (language was hard to understand)<br>- Misery Oriented: Too Much Focus on Disease (wanted to avoid unnecessary focus on ill health)<br>- Fear Provoking: Promoting Unnecessary Worry (concerned that reading the medical records would make them upset or worried)<br>- Energy Demanding: Unsuitable When Ill Health Drains You of Your Vigor (not enough energy to log in)<br>- Cumbersome: Screen May Not Be Better Than Paper (difficulties in navigating and engaging with new digital services)<br>- Impoverishing: Resisting the Digital Transformation of Individual and Social Life (general skepticism toward the ongoing digitalization of life worlds)	
vanAcker 2023	Older Adults' User Engagement With Mobile Health: A Systematic Review of Qualitative and Mixed-Methods Studies.		Analytical themes<br><br>Limited capabilities:<br>- physique<br>- computer literacy<br>- personal relevance<br>- mode of delivery<br>- ease of use<br>- complexity<br>- Personalisation<br>- Aesthetics/design<br><br>Prerequisite of motivation:<br>- Motivation<br>- Personal relevance<br>- Goal setting<br>- Self-monitoring

PUBLICATION	TITLE	BARRIERS	FACILITATORS
			- Feedback and goal review<br>- Social comparison<br>- Gamification<br><br>Importance of social support:<br>- Support of relatives<br>- Professional support<br>- Guidance
vanVelthoven 2019	Digitization of healthcare organizations: The digital health landscape and information theory		[distributed, connected intelligence embed in<br>devices and entities:]<br>-Security<br>- Sufficient number of users<br>- Regulatory compliance<br>- Low cost<br>- Evidence of benefits<br>- Clear target market<br>- Utility<br>- Appealing<br>- Fun<br><br>[platforms enabling peer-to-peer exchange to uncover 'private' information and assets:]<br>- Security<br>- Sufficient number of users<br>- Interoperability<br>- Dynamic consent of patients<br>- Personalised<br>- Interface neutrality<br>- Reproducibility<br><br>[original content creators:]<br>- Low cost<br>- Accessibility<br>- Purpose of the data<br>- Validation<br>- Credibility<br>- Language<br>- Relevance<br>- Accuracy of information

PUBLICATION	TITLE	BARRIERS	FACILITATORS
			[platforms, devices, and software to bundle,<br>store and deliver others' content/products:]<br>- Security<br>- Interoperability<br>- Searchability<br>- Ease of use<br>- No vendor lock-in<br>- Interface neutrality<br>- Good user-interface<br>- Capacity<br><br>[provide infrastructure ('information pipes')<br>for transmitting information:]<br>- Security<br>- Interoperability<br>- Regulatory compliance<br>- Ownership<br>- Fast<br>- Reliable (uninterrupted connectivity)
Vecchia 2022	Willingness of French General Practitioners to Prescribe mHealth Apps and Devices: Quantitative Study	-dangers linked to misuse of mHealth apps and devices by patients<br>-risks associated with<br>self-medication, dehumanization of the patient–physician relationship<br>- increase in patient anxiety because of the wealth of information available<br>-use of personal data of patients<br>-possibility of monitoring activities of GPs by health authorities<br>- devotion of additional time to mHealth apps and devices during consultations	-better access to care for patients<br>-patient empowerment<br>-better communication, quality of life, and work management for caregivers ; '-possibility of having a software aid that would automatically suggest mHealth apps and devices adapted to the needs of the patient<br>-obtaining additional information from patients (Patient-Reported Outcome Measures<br>-facilitating links between the various professionals involved in patient care<br>-an alternative to prescribing drugs<br>-the strengthening of the patient–physician

PUBLICATION	TITLE	BARRIERS	FACILITATORS
			relationship<br>- the perception of the importance of the role<br>of the physician in the transition to mHealth
Vo 2023	Multi-stakeholder preferences for the use of artificial intelligence in healthcare: A systematic review and thematic analysis	AI implementation:<br>- ambiguous understanding of how AI will be used [patients/members of general service]<br>- Lack of familiarity with AI [patients/members of general service]<br>- Lack of resources [patients/members of general service]<br>- Lack of transparency [patients/members of general service]<br>- Lack of trust [patients/members of general service]<br>- Low computer literacy [patients/members of general service]<br>- Physician disapproval of AI [patients/members of general service]<br>- Regulatory and legal uncertainties [patients/members of general service]<br>- Easily affected by cyber threat [healthcare professionals]<br>- Hindering by high costs [healthcare professionals]<br>- Inconsistent performance of AI applications [healthcare professionals]<br>- Lack of resources [healthcare professionals]<br>- Lack of trust from stakeholders [healthcare professionals]<br>- Low public engagement [healthcare professionals]<br>- Regulatory and legal uncertainties [healthcare professionals]<br>- Unstructured planning and monitoring of AI implementation [healthcare professionals]	Key factors influencing the acceptability of AI use:<br>- AI explainability<br>- Accessible costs<br>- AI accuracy<br>- Technology familiarity<br>- Trust by patients/health professionals<br>- AI safety<br><br>Perceived benefits:<br>- test accuracy<br>- medical error reduction<br>- reduced workload for health professionals<br>- lower health expenses<br>- increased healthcare access<br>- reduced waiting and traveling time for patients<br>- efficiency<br>- clinical and non-clinical workload reduction<br>- workflow efficiency improvement<br>- enhancement of medical capabilities<br>- reduce medical errors<br>- sharpen quality of clinical skills<br>- improve risk detection<br>- make better decisions<br>- enhance recommendations system<br>- improve patient-clinician relationship by enabling clinicians to spend more time providing greater care to patients<br>- helps patients better understand their health situation<br><br>Perceived AI risks:<br>- Bias<br>- Data privacy

PUBLICATION	TITLE	BARRIERS	FACILITATORS
			- Decrease efficiency<br>- Dependence on technologies<br>- Increase healthcare disparities<br>- Medical errors<br>- patient safety<br>- Reduce healthcare access<br><br>Perceived AI challenges (patients and members of general public):<br>- Care experience<br>- Suitability (lack of capability to deal with complications/to detect rare conditions, Lack of context or human experience)<br>- Technical uncertainties<br>- Unregulated standards<br><br>Perceived AI challenges (healthcare professionals):<br>- Care experience<br>- Suitability (Difficulty to apply to controversial subjects/to some health areas/to some patients/in emergencies)<br>- Technical barriers ; AI implementation:<br>- Clarify processes and roles of those who are involved in the procedure [patients/members of general service]<br>- Human doctors need to be ready and capable of handling AI [patients/members of general service]<br>- Patient awareness of AI [patients/members of general service]<br>- Reimbursement of AI medicine or devices [patients/members of general service]<br>- Clear referral pathway [healthcare professionals]<br>- Easily integrated into existing IT systems [healthcare professionals]<br>- Expect a large added value of AI [healthcare professionals]<br>- Innovations strategies from the

PUBLICATION	TITLE	BARRIERS	FACILITATORS
			leadership team [healthcare professionals]<br>- local champions [healthcare professionals]<br>- more education and training on AI [healthcare professionals]<br>- Patient rights and safety [healthcare professionals]<br>- Reimbursement of AI [healthcare professionals]
Wali 2022	Primary Care Physician's Perception and Satisfaction With Telehealth in the National Guard Primary Healthcare Centers in Jeddah, Saudi Arabia in 2022.	-concerns about patients overusing services<br>-patients limited technical knowledge<br>-patients limited access to technology/devices<br>-connectivity issues<br>-concerns about patient privacy<br>-adequate administrative support<br>-obtaining email consent<br>- adequate training/education<br>-lack of integration with current workflow or electronic medical records<br>-unable to justify the cost	[Support Offered to PHC Physicians for Conducting Virtual Visits]<br>-local colleague support<br>- In-house organizational supports<br>- change management supports<br>-technical training on using tools<br>-written information about how to integrate the tool into the workflow<br>-evidence about the effectiveness of the tool<br>-virtual care standards outlined by my profession`s college<br>- written resource on comparison of virtual visit platforms
Wardlow 2022	Development of telehealth principles and guidelines for older adults: A modified Delphi approach		-Integrated and coordinated telehealth facilitates access to older adults' health records.<br>-Integrated and coordinated telehealth facilitates safe, coordinated transitions of care.<br>-integrated and coordinated telehealth is integrated into the care continuum.<br>- Integrated and coordinated telehealth connects crucial stakeholders throughout the entire process.<br>-Integrated and coordinated telehealth supports staff working at the top of their licenses to drive efficiency<br><br>-Equitable and accessible telehealth accounts for older adults' physical and

PUBLICATION	TITLE	BARRIERS	FACILITATORS
			cognitive differences.<br>- Equitable and accessible telehealth accounts for cultural and linguistic differences of older adults and their caregivers.<br>-Equitable and accessible telehealth accounts for technology literacy and readiness of older adults and their caregivers.<br>- Equitable and accessible telehealth addresses needs across all settings, including the home, as promptly as possible.<br>-Equitable and accessible telehealth ensures that staff and providers engage in ongoing education on best practices for using telehealth with older adults.<br>- Equitable and accessible telehealth accounts for differences in access to technology and connectivity.
Waschkau 2020	Evaluation of attitudes towards telemedicine as a basis for successful implementation: A cross-sectional survey among postgraduate trainees in family medicine in Germany	-safety of data<br>-lack of user-friendly software<br>-high investment costs<br>-fear of losing the personal, physical contact with patients<br>-fear of data misuse in telemedicine<br>-lack of user friendly software<br>lack of appropriate instruction for the devices<br>- negative attitude of the employees involved	
Weichelt 2019	A model for assessing necessary conditions for rural health care's mobile health readiness: Qualitative assessment of clinician-perceived barriers	1) clinician readiness, (2) patient readiness, and (3) organizational readiness - clinician familiarity<br>- clinician time<br>- electronic medical record/data<br>- health Insurance Portability and Accountability Act/Protected Health Information<br>- Patient connectivity/technology<br>-Organizational direction<br>-Patient acceptance<br>-Patient affordability<br>-Uniformity of use<br>-Hindering patient-provider communication<br>-Technology reliance and limited patient face time<br>-Usability of the app/technology	

PUBLICATION	TITLE	BARRIERS	FACILITATORS
Weik 2024	Understanding inherent influencing factors to digital health adoption in general practices through a mixed-methods analysis.	'-technological barriers<br>-social barriers<br>-organizational barriers ; -lack of time<br>-familiarity with digital health solutions<br>-reliability<br>-usefulness<br>-technical support<br>-familiarity and ability of practice staff<br>-patients´ preferences and ability<br>- a lack of time<br>- the socio-political context<br><br>technological barriers:<br>-Design & technical concern<br>- Perceived usefulness<br>- Perceived ease of use<br>-system reliability<br>-Interoperability<br>-Infrastructure requirements<br><br>social barriers:<br>-Awareness<br>- Familiarity<br>-Attitude<br>- Interaction with patients<br>- applicability to patients<br>- Access to care<br>- Patients preference & ability<br>-Professional security<br>-Endorsement by colleagues<br><br>organizational barriers:<br>-Changes in work processes<br>-Reimbursement & costs<br>-clinical evidence<br>- lack of information<br>-Workload<br>- Training<br>- Strategic plan<br>- Socio- political context<br>- Privacy & security concerns<br>-Medicolegal issues	Development-related strategies:<br>-Involvement in development<br>-improved usefulness<br>-improved usefulness<br>-Improved ease of use<br>-Improved interoperability<br>-Integration of user feedback<br>-Adaptability & customizability<br><br>Awareness-related strategies:<br>-Overview of dh<br>-medic society recommend..<br>-peer recommend.<br>-patient requests<br><br>Knowledge-related strategies:<br>-info re functionality<br>-info re. benefits<br>-info re financing<br>-info re. patient applicability<br>-info re. privacy & security<br><br>Implementation-related strategies:<br>-Trial version<br>-technical support<br>-training material<br>-Ongoing training<br>-Patient education<br><br>Policy-related strategies:<br>-medic. training integration<br>-financing & reimbursing<br>-political guidelines<br>-data security policies

PUBLICATION	TITLE	BARRIERS	FACILITATORS
		-Time	
Weinert 2022	Perspective of Information Technology Decision Makers on Factors Influencing Adoption and Implementation of Artificial Intelligence Technologies in 40 German Hospitals: Descriptive Analysis	-technological challenges<br>-consumer and patient trust<br>in AI, regulatory acceptance, and in some cases, mandated work councils (mandated institutions of non-union employee representation<br>-lack of top managerial support<br>-need for highly skilled and trained stuff<br><br>- Lacking resources (staff, knowledge and financial)<br>-lacking compatibility or interoperability with existing IT infrastructure<br>-Quality of data<br>- availability of data<br>- ethical aspects<br>-product range on the market<br>- data protection<br>-quantity of data<br>-legal regulations<br>-consent of the work council<br>-corporate culture<br>-User (eg, physicians, nurses, and administration) acceptance)<br>- leadership acceptance<br>-patient acceptance	Perceived opportunities associated with the implementation and use of artificial intelligence:<br>-Increase in efficiency due to time-saving effects<br>-Competitive advantage<br>-Increase in quality of care<br>-Easing the workload of employees<br>-Financial savings
Weltin 2021	The role of telemedicine in gynecologic healthcare: A narrative review	- Access to adolescents limited in online services only seeing adults<br>- A lab visit may be needed for comprehensive visit<br>- Service gaps for individuals experiencing homelessness and those without internet or computer/smartphone<br>- More work is required to develop continuity of care and rapport with patients via video<br>- Provider discomfort with clinical decision-making without an exam or discomfort with sensitive exam<br>- Policies relating to billing and reimbursement, practice across state lines, malpractice all in flux<br>- Technology costs and need for training, technical support, and infrastructure maintenance	Benefits:<br>- Ease of appointment access<br>- Confidentiality<br>- Clinical outcomes equal to face-to-face care<br>- Appeals to tech-savvy populations<br>- Useful for triage in high-priority case<br>- Improves follow-up rates
Wilson 2021	Barriers and facilitators to the use of e-health by older adults: a scoping review.	[Individual:]<br>- Intrinsic: Ageing limitations, Perceived self-efficacy, Lacking confidence in e-health, Fear and dislike of technology, No interest in learning<br>- Extrinsic: Lack of experience/skills with e-health or technology, Lack of knowledge of e-health, Previous negative experience, unmet expectations, lack of need to change, fear that traditional services may perish, disbelief in efficacy in e-health, lack of external accountability, inability to incorporate into routine, required effort,	[Individual:]<br>- Intrinsic: Desire to learn, Motivation to make a lifestyle change, Altruism (wanting to contribute to scientific progress)<br>- Extrinsic: Belief that e-health services are of benefit, Convenience of e-health,

PUBLICATION	TITLE	BARRIERS	FACILITATORS
		cultural limitations such as language barriers and e-health detracting from time with family<br><br>[Technological:]<br>- Functional: Small screen and text, small icons and lack of colour contrast, complex functionality, poor functionality<br>- Content: Lack of alerts, Alert fatigue (reminders, emails, text), Condescending and impersonalised communication, inability to respond to reminders, Overwhelming and difficult to understand content, Too much content on one page<br>- Availability: Lack of access to electronic equipment, cost of electronic equipment and internet service<br><br>[Relational:]<br>-Technological Support: No training/support to learn, No one to help troubleshoot issues, Reliance on family for guidance and lack of family's patience and understanding while learning<br>- Social Support: Lack of social interaction, Absence of interpersonal communication, Communication through technology considered an "inauthentic experience"<br><br>[Environmental:]<br>- Location: Poor/unreliable internet<br><br>[Organisational:]<br>- Privacy: Health information concerns<br>- Trust: Unknown accuracy of information, Not knowing who people are communicating with, Concern over management of emergency situations, Concern over Western Medicine's priorisation of medication<br>- Data sharing: Lack of communication between health platforms	Ability to incorporate into current routine, Previous experience and skills, Previous experience with e-health and required skills, Positive experience with technology generally, Opportunity to learn new information<br><br>[Technological:]<br>- Functional: Ease of use such as audio feedback and large and clear visual feedback<br>- Content: Personalised content, use of reminders/alerts, use of images<br>- Availability: Free or low-cost electronic equipment<br><br>[Relational:]<br>-Technological support: Training/support to learn, Dedicated coach for training and continued support, peer-to-peer platform to share experiences, option for family/carer to provide support<br>- Social support: Socially inclusive and community-based information<br><br>[Environmental:]<br>- Location: Availability to rural/remote populations<br><br>[Organisational:]<br>- Trust: Recommendation from physician, Content designed by experts in the field, Access to specialists through platform, Authenticity (platform with clear credentials)<br>- Data sharing: Sharing of health information between health care providers
Wolff 2021	Success Factors of Artificial Intelligence Implementation in Healthcare	-major technological limitations<br>that constrain AI implementation<br>-major policy<br>deficiencies that inhibit AI implementation<br>-ack of clinical and economic	"key factors for AI development planning"<br>-'-Technological Implementation<br>-Application scenario differentiation<br>-data processing structure definition

PUBLICATION	TITLE	BARRIERS	FACILITATORS
		impact measurement further contributes to the low level of practical implementation	-privacy by design and product class setting<br>-indication-focused<br>-institution focused<br><br>-data access<br>-data exchange pathway<br>-data confidentiality<br><br>-AI technology implementation with a "privacy-by-design" structure<br>-compliance with medical product classification<br>-adaptability for changing AI regulatory requirements
Wubineh 2023	Exploring the opportunities and challenges of implementing artificial intelligence in healthcare: A systematic literature review	Ethical and privacy related challenge:<br>- Patient data protection<br>- Ethical boundaries of innovation<br>- Obtaining informed consent for use<br>- Ensuring data privacy<br>- Maintaining safety<br>- Promoting transparency<br><br>Healthcare Providers and Professional Liability:<br>- Impact of professional responsibility<br>- Challenges when learning to integrate and use technology<br>- Varying levels of technology literacy<br>- Limited hands-on experience using AI applications<br><br>Unreliability and trustworthiness of AI technologies:<br>- Lack of adequate focus on digital technology<br>- Lack of transparency and interoperability of AI algorithms<br>- Lack of interoperability standards to model function<br>- Inaccurate predictions, diagnoses and biases in algorithms<br><br>Lack of awareness on AI technologies:<br>- Lack of understanding<br>- Unrealistic expectations	Opportunities of AI:<br>- Teamwork and decision making<br>- Technological advancements<br>- Diagnosis and patient monitoring<br>- Drug development<br>- Virtual health assistants
Yang 2022	Artificial intelligence healthcare service resources adoption by medical institutions based on TOE	- high risk of data leakage<br>- system service complexity<br>- AI infrastructure synergy<br>-skeptical of AI Processing capabilities	- increase policy subsidies on the demand side and capitation to improve the elderly’s ability to pay.<br>-Accelerate the entry of social capital and

PUBLICATION	TITLE	BARRIERS	FACILITATORS
	framework	-lack of awarness of value and benefits of AI healthcare technology<br>- medically derived risks<br>- lack of ability to read structured medical data<br><br>-lack of management leadership support<br>-hospital size<br>-financial costs<br>-lack of excellent supplier support<br>-inability to share information<br>-lack of complex talent<br><br>-government policies<br>-competitive pressures<br>-geographical restrictions<br>-unclear ownership of hospitals<br>-lack of patient trust<br>-difficult to meet complex needs of elderly patients<br>-lack of excellent partnerships	strengthen the<br>role of financial funds to guide the<br>construction of<br>elderly care institutions.<br>- Guide “to capacity,” reasonably promote<br>mergers and<br>acquisitions.<br>-Introduce excellent AI providers with<br>high professional-<br>ism into medical institutions.<br>-Encourage cooperation between public<br>medical institutions and elderly care<br>institutions, as well as implement<br>and subsidize public and private elderly<br>care pilot<br>programs.<br>- Coordinate port compatibility issues<br>among suppliers<br>and develop technological specification<br>rules for AI<br>healthcare service
Ye 2019	How resource scarcity and accessibility affect patients' usage of mobile health in China: Resource competition perspective	age is negatively and significantly<br>related to the adoption of mHealth services, with young people<br>showing higher acceptance than the elderly<br>-	-women exhibit more positive attitudes<br>toward mHealth services<br>-experience has a significant and positive<br>impact on the adoption of mHealth<br>-resource scarcity<br>is positively and significantly related to<br>the adoption of mHealth<br>-patients have a higher mHealth<br>adoption rate when medical resource<br>accessibility is low.
Ye 2023	Implications for implementation and adoption of telehealth in developing countries: a systematic review of China’s practices and experiences	Outer setting:<br>- Unequal medical and<br>technological resource<br>distribution<br>- Lack of standardized guidelines<br>and supporting evidence<br><br>Inner setting:<br>- Lack of long-term evaluation<br>methods to establish the<br>clinical value	Outer setting:<br>- Provided care to patients in resource-<br>limited areas<br><br>Inner setting:<br>- Reduced use of medical supplies and<br>human resources<br><br>Intervention characteristics:<br>- Lowered barriers (easy to use and<br>affordable) for diverse patient

PUBLICATION	TITLE	BARRIERS	FACILITATORS
		- Insufficient human resources and inadequate incentives<br><br>Intervention characteristics:<br>- Unstable internet quality<br>- Not friendly to users with low digital literacy<br>- Different health care services require different modalities (e.g., video, audio)<br>- Privacy and security concerns<br>- Low diagnostic accuracy<br><br>Characteristics of individuals:<br>- Low acceptance and buy-in<br>- Preference for face-to-face interactions<br><br>Process:<br>- Lack of public awareness<br>- Inadequate motivation and training<br>- Additional workload for providers	populations<br>- Reduced adverse events<br>- Supported multiple functions and smooth patient-provider communication<br><br>Characteristics of individuals:<br>- Increased evidence base (especially with rigorously designed studies) to improve access or care at a reasonable cost<br><br>Process:<br>- Increased awareness of telehealth among the public<br>- The train the trainer model alleviated workload for providers who were in high demand<br>- Partnered with third-party technology companies
Young 2020	A mobile app to capture EPA assessment data: Utilizing the consolidated framework for implementation research to identify enablers and barriers to engagement.	-lack of time<br>-competing demands such as clinical workload<br>-challenges with the assessment frameworks; (the competencies and milestones used on workplace-based assessments are viewed by some as too numerous, too granular, and/or too abstract for educators to use)<br><br>[Intervention characteristics:]<br>– Residents and faculty see the value of assessment tools (such as the paper-based form also used in the clinic) which generate more comments that are more detailed, nuanced, and comprehensive<br>– The absence of a checklist, while making the app easier to use, led to less systematic observation and feedback<br>– No reinforcing comments<br>– Most faculty did not understand the entrustment scale and/or the EPA framework<br>– Faculty prefer paper-forms for discretely jotting down feedback points while observing	[Intervention characteristics:]<br>– Sufficient training prior to use<br>– Few, if any, technical challenges<br>– EPA app intuitive and easy to use, especially compared with pa-per-based assessment tools<br>– Feedback timely and frequent<br>– Feedback quality high—behaviourally specific and salient<br>– User interface forced succinct feedback with a single take home message for the resident<br>[Characteristics of individuals:]<br>-Exciting about use of app-based technology<br>-High confidence in use of the app<br>–Faculty appreciated how the interface forced synthesis and distillation of their

PUBLICATION	TITLE	BARRIERS	FACILITATORS
		[Characteristics of individuals:]<br>-Faculty worry that use of the EPA app during patient encounters may convey lack of respect and attention<br>–Residents reviewed emailed feedback briefly, then rarely referred to it again<br>– Faculty prioritized verbal feedback over app completion when short on time<br><br>[Inner setting:]<br>-Clinical demands, especially from the residents' panels of patients, often resulted in the EPA app assessment not being completed	observations into a single, concise feedback point<br>[Inner setting:]<br>-Faculty time protected for the sole purpose of directly observing the resident and giving feedback<br>– Monitoring of app utilization by the program<br>[Outer setting:]<br>-The app aligned with the organization's emphasis on innovation, especially regarding the use of measurement and technology, in clinical and educational practice
Yuen 2023	The determinants of users' intention to adopt telehealth: Health belief, perceived value and self-determination perspectives	Non-Significant Influence of Self-Efficacy: While self-efficacy is often assumed to contribute to perceived value, in this study, it was found to have a non-significant impact. This suggests that users might not feel their personal capabilities (self-efficacy) strongly influence their decision to adopt telehealth.<br><br>Functional and Environmental Value: While emotional factors play a significant role, the functional and environmental value of telehealth might not be as compelling for users, acting as a barrier for adoption in some cases.	Perceived Threat: Users are more likely to adopt telehealth services when they perceive a health crisis as a threat. This is a significant facilitator as it highlights the urgency of using telehealth to mitigate health risks.<br><br>Cues to Action: Positive cues and encouragement from external sources increase the likelihood of telehealth adoption. These cues can come from healthcare providers or public health campaigns.<br><br>Perceived Relatedness: Communication and interaction with professionals in a digital environment enhance users' perceived value of telehealth, acting as a strong facilitator.<br><br>Perceived Autonomy and Self-Efficacy: These factors positively impact users' emotions, making them more inclined to adopt telehealth.<br><br>Perceived Value: A higher perceived value of the service directly influences users' intention to adopt telehealth. It also

PUBLICATION	TITLE	BARRIERS	FACILITATORS
			positively affects users' emotional responses.<br><br>Emotion: Positive emotions linked to telehealth usage increase the intention to adopt these services.
Zachrison 2019	Understanding Barriers to Telemedicine Implementation in Rural Emergency Departments	-clinical concerns<br>-legal concerns<br>-cost concerns<br>-administrative concerns<br>-fear of losing patients<br>-not valuable for patients care<br>-technologic concerns<br>-telemedicine not necessary to meet patients needs	
Zakerabasali 2021	Mobile health technology and healthcare providers: Systemic barriers to adoption	Technical barriers:<br>- Lack of existing technology<br>- Concerns about regulation and efficacy of applications<br>- Security and privacy concerns<br>- User-friendliness<br>- Compatibility with the workflow<br>- Connectivity speed<br>- Lack of interoperability and integration with other<br><br>Individual barriers:<br>- Lack of physician support<br>- Resistance to change<br>- Difficulty understanding the technology<br>- Human appeal<br>- Knowledge and limited literacy<br><br>Healthcare system barriers:<br>- Legal barriers<br>- Reimbursement and accountable care organizations<br>- Economic and financial factors<br>- Lack of health system policies<br>- Lack of standards	
Zarei 2023	Application of artificial intelligence in medical education: A review of benefits, challenges, and solutions	- Essential infrastructure<br>- Ethical issues<br>- Complete interdisciplinary collaborations<br>- Cheating & Plagiarism<br>- Technical Malfunction<br>- Lack of an Evaluation Method for Educational Strategies	Advantages:<br>- Effective Student Assessment<br>- Clinical Setting Simulation<br>- Active Learning Strategies<br>- Cost-effectiveness<br>- Curriculum Review

PUBLICATION	TITLE	BARRIERS	FACILITATORS
			- Continuous & Distance Learning
Zhao 2023	Does the transcultural problem really matter? An integrated approach to analyze barriers to eHealth SMEs' development	'-Informational barriers<br>-Marketing barriers<br>-Environmental barriers<br>-procedural barriers<br>-tasks barriers<br>-governmental barriers ; -Lacks of links<br>-labour shortage<br>-difficulty in accessing funding<br>-limited re-innovation capability<br>-poor staff support<br>-poor leadership<br>-transcultural problems<br>-limited product scalability<br>-lack of knowledge about product promotion<br>-weak public awareness of eHealth<br>-The impacts of COVID-19<br>- Lack of specific digital skills<br>-Problems with user experience evaluation<br>-Lack of knowledge about the end user and routes to markets<br>-Problems with eHealth policy dissemination<br>-lack of adequate infrastructure	
Zharima 2023	Exploring the barriers and facilitators to implementing electronic health records in a middle-income country: a qualitative study from South Africa		[Factors affecting EHR implementation:]<br>- Leadership: Leaders that understand the scale of implementation,<br>and obtaining buy in from various stakeholders for a<br>collaborative implementation process, Leadership appointments based on merit rather than<br>political affiliations, Internal conflicts leading to uncertainty and<br>hindered progress<br>- Skills and expertise: Capacity building to address skills shortage in the<br>public health sector, collaborating with more skilled<br>sectors can be helpful, Health care workers should be involved in the implementation process, Early training programs can prepare the health care workforce for implementation

PUBLICATION	TITLE	BARRIERS	FACILITATORS
			- Resources, funding and infrastructure: Long-term investments towards infrastructural needs<br>(hardware, connectivity, etc.), Increased funding for public health facilities in remote areas, Setting up data storage centers to facilitate the<br>integration and migration of health data, Building on existing infrastructure while gradually<br>upscaling<br>- Governance: Alignment between provincial and national health departments for concerted implementation,<br>transcending the federal system, Better monitoring mechanisms for tender systems in<br>to combat corruption and improve accountability, Appropriately qualified and skilled authorities to<br>prioritize digital health, transparent and multi-disciplinary panel of<br>experts to help with planning and decision-making
Zhou 2019	Barriers to and facilitators of the use of mobile health apps from a security perspective: Mixed-methods study	- Concerns about health data security and privacy; especially when the apps are for issues associated with stigma, social isolation, or discrimination<br>such as HIV/AIDS, sexual orientation, and mental disease<br>- hard-to-use user interface or security features<br>- high cost of the app<br>- when apps use a<br>context-sensing approach to generate reminders or suggestions<br>- location tracking<br>-fear that some information collected by the<br>mobile device might get compromised during transmission and<br>storage<br>- app runs slow<br><br>[What barriers would prevent the adoption and integration of a mHealth App into your health monitoring and management?]:<br>- Price (not free, not below 5$)<br>- app sends data to a remote server without my permission	[What specific privacy policies of an mHealth app would encourage you to use the app for your own health care purpose?]<br>- data will not be shared with any unauthorized personnel<br>- data will be collected only if you give permission to the app<br>- data will be removed from the server if you request it<br>- right to terminate the permission for data collection at any time<br>- data will be collected only for health care and/or research purposes<br><br>[What security measures would give you the confidence that an mHealth app would

PUBLICATION	TITLE	BARRIERS	FACILITATORS
		- app asks to provide personal information even when I just want to determine whether the app is good for me<br>- app does not encrypt personal data<br>- app does not have a clear privacy statement<br>- app stores personal data on my mobile device and makes data easily accessible to anyone who can access my mobile device<br>- daily alerts<br>- name of the app (should not imply a certain disease)<br>- app asks to set up an account with user name and password	protect the confidentiality of patient data?]<br>- explicit encryption<br>- user authentication<br>- remote removal of my personal data on a lost mobile device<br>- access control<br>- easy-to-understand privacy policy<br>- data transmission via secure channel<br>- easily adjustable security settings for different type of data<br>- health care providers' activities are logged and can be audited<br>- one unique account for each patient and health care provider<br>- regular password update ; '- low data entry burden<br>- a clear patient protection privacy policy<br>- intuitive user interface<br>- strong but easy-to-use security features<br>- data protection by security measures<br>- adjustments to mHealth apps’ security features for different user groups in terms of marital status, sex, age, and income<br>- free MHealth apps
